# Terminator Operon Reporter: combining a transcription termination switch with reporter technology for improved gene synthesis and synthetic biology applications

**DOI:** 10.1038/srep26572

**Published:** 2016-05-25

**Authors:** Massimiliano Zampini, Luis A. J. Mur, Pauline Rees Stevens, Justin A. Pachebat, C. James Newbold, Finbarr Hayes, Alison Kingston-Smith

**Affiliations:** 1 Institute of Biological, Environmental and Rural Sciences, Edward Llwyd Building, Aberystwyth University, Aberystwyth SY23 3FG, UK; 2 Faculty of Life Sciences, University of Manchester, Manchester M13 9PL, UK

## Abstract

Synthetic biology is characterized by the development of novel and powerful DNA fabrication methods and by the application of engineering principles to biology. The current study describes Terminator Operon Reporter (TOR), a new gene assembly technology based on the conditional activation of a reporter gene in response to sequence errors occurring at the assembly stage of the synthetic element. These errors are monitored by a transcription terminator that is placed between the synthetic gene and reporter gene. Switching of this terminator between active and inactive states dictates the transcription status of the downstream reporter gene to provide a rapid and facile readout of the accuracy of synthetic assembly. Designed specifically and uniquely for the synthesis of protein coding genes in bacteria, TOR allows the rapid and cost-effective fabrication of synthetic constructs by employing oligonucleotides at the most basic purification level (desalted) and without the need for costly and time-consuming post-synthesis correction methods. Thus, TOR streamlines gene assembly approaches, which are central to the future development of synthetic biology.

Synthetic biology is an emerging discipline that aims to apply engineering principles to biology and has the potential to drive a revolution in biotechnology^1^. The discipline has progressed particularly as a result of the implementation of novel and powerful DNA construction techniques that allow for efficient assembly and manipulation of sequences both *in vitro* and *in vivo*^2^,^3^. At the most basic level among DNA fabrication strategies, gene synthesis^4^ is a key pillar supporting synthetic biology.

Despite extraordinary advances in recent years, the chemistry behind gene synthesis is not yet an error-free process. A fundamental source of sequence errors in synthetic genes derives from the presence of upstream mutations, most commonly deletions or insertions, in the oligonucleotide pool used to prepare the assembly^5^. Several methods have been developed to reduce the error rate in the final construct such as the use of highly purified oligonucleotides, the development of functional assays based on intrinsic detectable properties of the assembled gene, or the addition of a reporter tag to the synthetic element^6^,^7^. However, assays based on the detection of catalytic activities are limited to enzymes, and reporter tags expressed in fusion proteins may fold inappropriately thereby impairing activity^7^. Alternative error removal strategies involve highly controlled assembly conditions, the use of mismatch binding or mismatch cleavage enzymes, or site-directed mutagenesis approaches in the post assembly stages. All of these approaches are costly and time consuming, and sequencing of multiple samples is normally necessary to identify clones harbouring the correct sequence^8^.

Developed with the aim of overcoming current challenges in gene synthesis, Terminator Operon Reporter (TOR) described here is a novel gene synthesis platform whose principle relies on the established synchrony between transcription and translation in bacteria. Whereas in eukaryotes these two fundamental steps of protein synthesis are spatially and temporally separated, transcription and translation occur simultaneously in prokaryotes. Thus, as RNA polymerase transcribes the 3′-end of bacterial mRNA, ribosomes bind to the 5′-end permitting concurrent translation of the newly synthesized transcripts. This synchrony allows translation to interfere with transcription in a number of well-documented cases. For example, ribosomes bound to bacterial mRNAs during transcription support efficient protein synthesis by promoting mRNA stability in several ways: masking endoribonuclease sites associated with mRNA decay, inhibiting the activity of RNA hairpin dependent transcriptional pausing signals, or hindering transcript access by transcription termination factors^9^,^10^,^11^,^12^. Transcriptional attenuation is another example of a ribosome-mediated event commonly found in amino acid biosynthetic operons in bacteria^13^. This type of transcriptional control depends on the ribosome-dependent folding of regulatory RNA stem-loops structures in the 5′ untranslated region of the operon^14^,^15^. Whilst such mechanisms are part of the natural regulatory machinery in prokaryotes, Wright and Hayward demonstrated almost three decades ago how ribosomes can efficiently inhibit the activity of intrinsic terminators located within the coding sequence (CDS) in artificial genetic contexts^16^. The TOR method developed here is based upon this principle elucidated by Wright and Hayward. The strategy combines an in-frame, switchable transcription terminator and a downstream reporter gene to monitor the precision of synthetic gene assembly.

## Results

**Figure 1:**
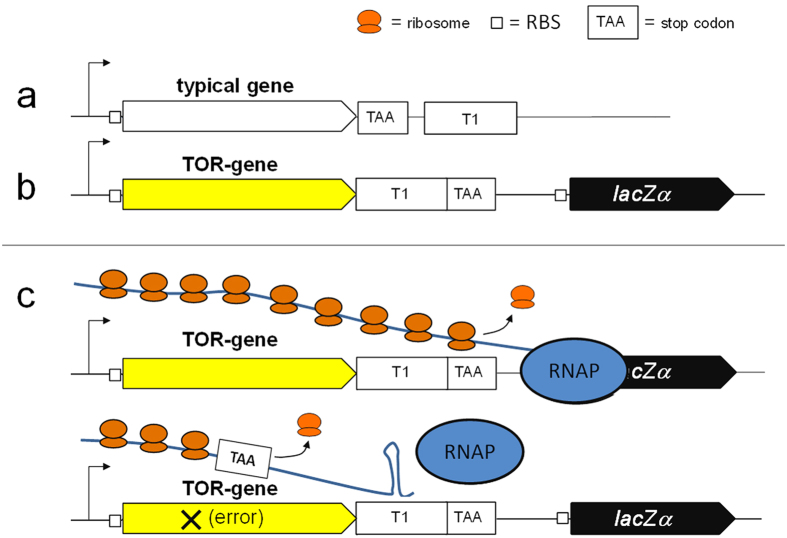
Principle of TOR. (**a**) The coding sequence in a typical protein coding gene is followed by a stop codon (TAA) and then by a transcription terminator (T1). (**b**) In the TOR platform the transcription terminator is placed upstream of the stop codon and the reporter follows as part of the same operon. (**c**, top) In TOR T1 is translated as a fusion tag and becomes inactive or is significantly impaired, thus the reporter can be expressed as the second gene of the operon. (**c**, bottom) In the presence of a premature stop codon arising after a nonsense or a frame-shift mutation, T1 will be active and the reporter is not expressed.

TOR is a gene assembly strategy in which the sequence of interest is assembled from oligonucleotides and cloned so that the strong T1 transcription terminator^17^ is positioned in-frame with the synthetic element (Fig. 1). The termination activity of T1 is abolished or significantly impaired when it is translated in *Escherichia coli*^16^ thereby allowing expression of a *lacZ*α reporter gene located downstream, as the second gene of a bicistronic operon. Thus, when the sequence of the synthetic gene is correct, read-through occurs of the T1 transcription terminator and the *lacZ*α gene is expressed to generate blue colonies on medium containing X-Gal. In contrast, frame-shift mutations lead to a stop codon shortly after the erroneous sequence. As the most common errors in synthetic oligonucleotides are deletions and insertions^5^,^18^, frame-shift mutations along the CDS are the most frequent. In constructs harbouring frame-shift mutations the T1 terminator is active and *lacZ*α is not expressed, so recombinant plasmids produce white (clear) colonies.

**Figure 2:**
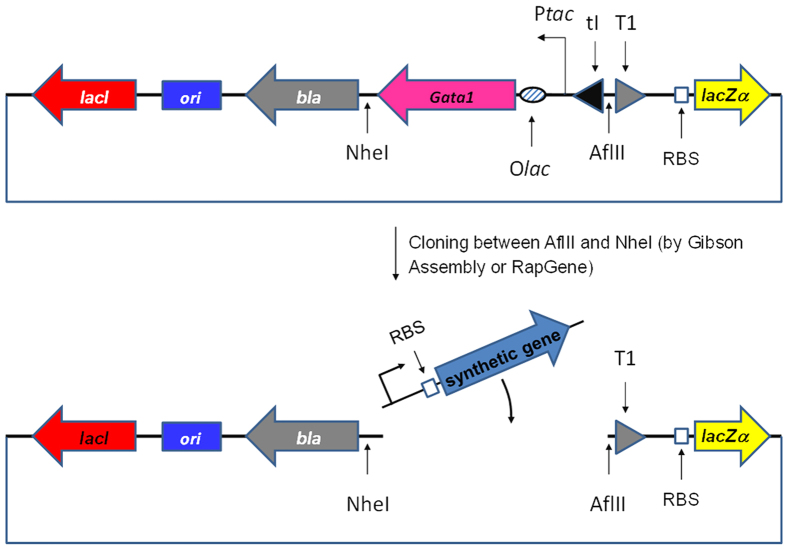
pRG2 structure (not to scale). pRG2 is the plasmid employed throughout this study. It features a wild-type *lacI* gene, the toxic and inducible *Gata-1*, the *bla* gene conferring resistance to ampicillin or carbenicillin, a pMB1 type origin of replication, the *lacZα* gene and two terminators with opposite orientations, λtI and T1. Simultaneous digestion of pRG2 with AflII, NheI and T4 DNA polymerase in the presence of dTTPs results in a plasmid having LIC-compatible ends suitable for RapGene assembly. On the contrary, ends produced by simultaneous NheI and AflII digestion of the vector in the absence of T4 DNA polymerase were used to create overlap regions in the PCA product suitable for the Gibson Assembly. The reporter gene *lacZα* is a transcriptional fusion with the synthetic element. The second terminator λtI was introduced to reduce leaky expression of *Gata-1* potentially resulting from engineering the original pGATA region upstream of the toxic element. Ori, origin of replication. NheI, restriction site for NheI. AflII, restriction site for AflII. O*lac*, *lac* operator. P*tac*, *tac* promoter.

TOR was tested with the prokaryotic *aadA1* gene (792 bp; GenBank HQ880267.1) that encodes aminoglycoside adenyltransferase which confers spectinomycin and streptomycin resistance in *E. coli*, and with the eukaryotic *gfp* gene (717 bp; GenBank L29345.1) that codes for *Aequorea victoria* green fluorescent protein^19^. These genes were selected as we have recently described their assembly by RapGene, a ligation independent approach for gene synthesis in *E. coli*^20^. The cloning reporter vector used throughout this study was pRG2 (Fig. 2). This plasmid is a positive selection vector based on the toxic gene *Gata1*^21^ and is derived from the previously described pRG1.0 vector^20^. As TOR was developed as an extension of RapGene^20^, the promoter and the ribosome binding site (RBS) were designed to be part of the pool of oligonucleotides in the assembly (Supplementary Fig. S1), as detailed in Methods. To ensure that frame-shift mutations located at the C-terminus of a synthetic gene also resulted in transcription termination at T1, two additional stop codons were embedded in the vector in reading frames two and three, respectively within an AflII site and just before the beginning of T1 (Supplementary Fig. S2).

Different *E. coli* promoters and terminators where tested for use in the TOR system. Preliminary successful results were obtained with a variant of the *E. coli* P_*recA*_ promoter, referred to here as P_recA_*, and the T1 terminator^17^. The sequence of P_recA_* differs from its wild-type sequence at one nucleotide of the −35 region (TTGATA was changed to TTGACA)^22^. In *recA*^+^ strains, P_*recA*_ is induced by RecA itself via proteolytic cleavage of the LexA repressor^22^. However the *E. coli* NEB5α strain used in this study is a *recA1*^−^ strain. This mutation has been reported to abolish completely all known RecA functions, including the protease activity towards LexA^23^. Thus, P_*recA*_ and P_recA_* are expected to be constitutively repressed in NEB5α. Two additional non-inducible promoters were also tested to assess the robustness of the TOR platform under different conditions: the *E. coli* P_*BAD*_ core promoter comprising only the sequence from −35 to −10 plus external 4 bp on both sides and no additional regulatory elements^24^, and the P_*tac*_ promoter^25^ comprising the −35 to −10 region with an additional 5 bp upstream and 4 bp downstream from the original pGATA plasmid (Supplementary Fig. S1).

**Figure 3:**
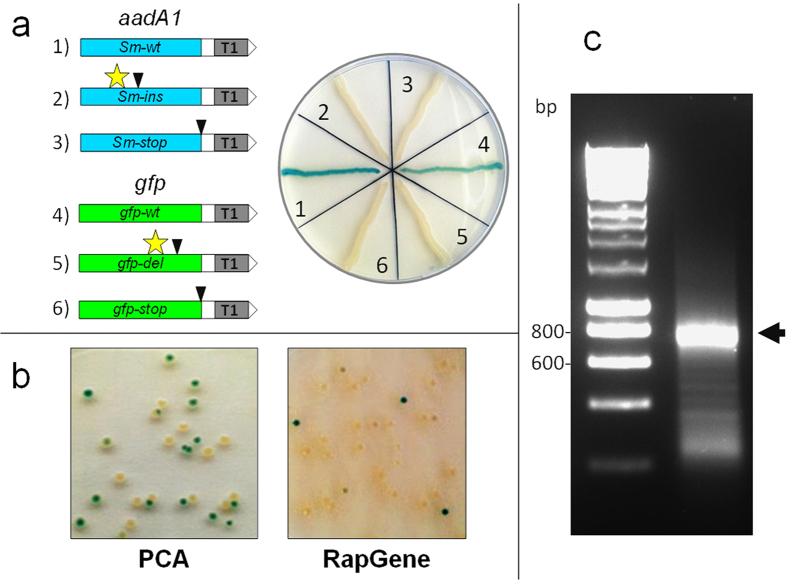
Application of TOR to *aadA1* and *gfp* genes. TOR was applied to six test constructs generated from *aadA1* and *gfp* by standard molecular biology techniques, and to *gfp* assembled by either RapGene or PCA assembly. (**a**) Preliminary assays on test samples. After approx. 36 hours incubation wild-type samples (1 and 4) conferred a dark blue colony colour on CIX, whereas the four mutants carrying a stop codon (black triangles) within or at the end of the CDS showed a white colour on the same medium. Stars correspond to frame-shift deletions or insertions that generate a premature stop codon further downstream. Grey boxes within the constructs correspond to the terminator T1. (**b**) TOR was applied to a gene synthesis assay for the assembly of *gfp* by PCA or RapGene methods starting from a pool of 24 overlapping oligonucleotides. (**c**) Electrophoresis of a typical *gfp* PCA product. The band corresponding to the insert (arrow) was gel-extracted and cloned by the Gibson assembly in pRG2.

TOR was tested initially on six genes comprising wild-type and nonsense mutants of *aadA1* and *gfp*, all expressed from P_recA_* (Fig. 3a, Supplementary Fig. S3). These genes, containing either wild-type sequences or a stop codon upstream of T1, showed that the TOR platform could easily discriminate between correct and incorrect sequences. In fact, on X-Gal-containing plates, colonies with the correct sequences appeared dark green or blue depending on the incubation time. This colony colour reflects read-through of the T1 terminator and consequent expression of the downstream *lacZ*α gene (Fig. 1). On the contrary, genes possessing a stop codon upstream of T1 resulted in white colonies on the same medium due to transcription termination at T1 and impairment of *lacZ*α expression. Colour development was assessed after a minimum of 30 hours incubation at 37 °C. (Fig. 3a).

**Figure 4:**
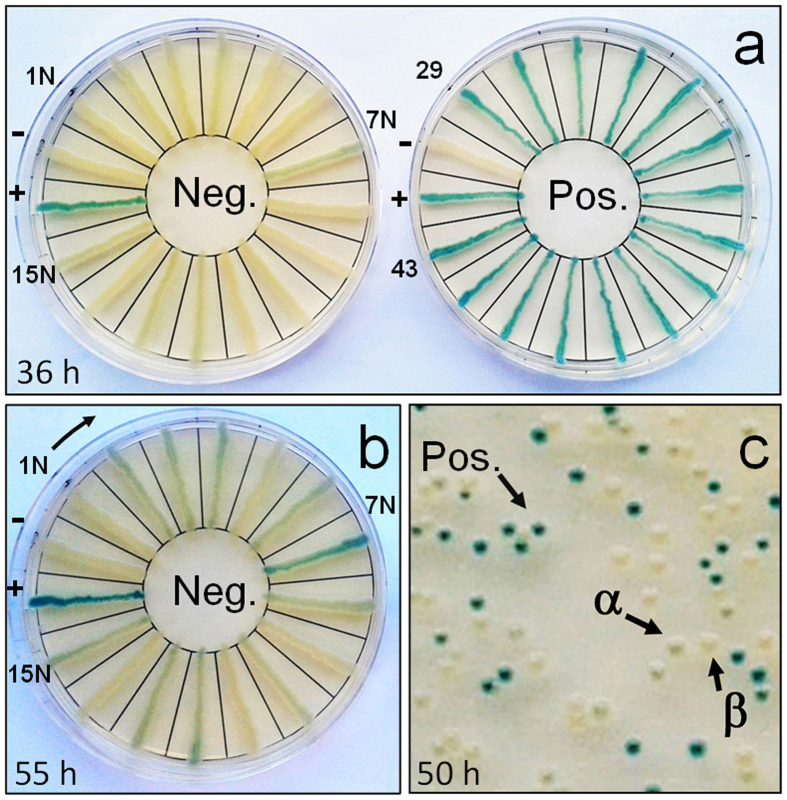
Analysis of TOR-negative samples obtained by TOR. (**a,b**) Comparison between the phenotypes of 15 PCA-positive versus 15 PCA-negative samples (both under P_recA_*). Eleven out of fifteen samples gradually darkened after prolonged incubation. A clear difference was still observable between positive and negative samples, but *gfp-ins* (sample N7) darkened more than the others. (**c**) TOR-negative samples showed two different colony colours upon cloning, green (α) and yellow (β). Neg., TOR-negative samples; Pos., TOR-positive samples. 1N, 7N, 15N, samples 1, 7, 15 from the TOR-negative samples.

In a following approach *gfp* was synthesized by either RapGene or polymerase cycling assembly^26^ (PCA) (Fig. 3b). These techniques (Supplementary Fig. S4) were used throughout the study as standard gene synthesis methods to test TOR efficiency. In a first round of TOR, with the synthetic *gfp* expressed from P_recA_*, fifteen randomly chosen TOR-positive candidates obtained by PCA assembly of *gfp* and 13 samples from those derived by RapGene were sequenced (Supplementary Data 1). Analysis of chromatograms showed that 66% of the positive samples identified by PCA and 61% of those obtained by RapGene contained the fully correct *gfp* sequence, while the remaining candidates contained either missense mutations or in-frame deletions (Table 1, Supplementary Data 1). In a second round of TOR, *gfp* was built only by PCA and placed under the control of either P_recA_*, P_*BAD*_ or P_*tac*_ promoters (Supplementary Figs. S1 and S5). Plasmids from fifteen transformants, randomly selected among the positive samples, were sequenced for each promoter. Here 93%, 86% and 73% of the positive samples contained a correct *gfp* sequence driven by the P_recA_*, P_*BAD*_ or P_*tac*_ promoters, respectively (Table 1). In the case of candidates expressed using P_recA_*, we additionally sequenced 15 TOR-negative transformants (Fig. 4, Supplementary Data 1). As expected, the latter group contained incorrectly assembled synthetic elements, featuring one or more frame-shift mutations followed by a stop codon emerging soon after the mutation site.

### Accuracy and error rates in TOR

As reported in other studies^18^,^20^ constructs harbouring full-length inserts can be used to determine the error rate. The error rate is defined as the total number of bases mutated (deleted, inserted or substituted) divided by the number of bases sequenced multiplied by 100. This value represents the statistical occurrence of a single mutation every 100 bp, and allows for comparisons between methods that use different strategies and different insert lengths. The error rate in TOR with RapGene was 0.136 from 13 samples sequenced from blue colonies on X-Gal, while the values determined for PCA assembly from a total of 60 positive samples varied between 0.009 and 0.045 (Table 1, column 6), with an average of 0.024. This latter value corresponds to 0.24 errors per kb, or equivalently to 24% sequences containing at least an error, for a 1 kb gene. Thus, with 76% correct sequences, less than two transformants per TOR cloning need to be sequenced to identify a correct synthetic gene of 1 kb.

The accuracy of gene synthesis in TOR was also determined as the ratio between the transformants harbouring a fully correct *gfp* gene divided by the total number of TOR-positive samples sequenced (Table 1, column 3). For the 714-bp *gfp* gene synthesized by PCA the accuracy was 79.5% from four separate trials revealing that values determined by both methods were similar. Thus, the efficiency and reliability of TOR synthesis for genes of ~1 kb is very competitive with market standards, as less than two TOR-positive transformants need to be sequenced to identify the correct sample (https://www.thermofisher.com/uk/en/home/life-science/cloning/gene-synthesis/gene-strings-dna-fragments.html). Extrapolating the observed error rate (0.024) to longer synthetic genes, the predicted value of 0.48 errors per 2 kb genes would allow identification of correct constructs of this length by sequencing as few as two transformants per cloning assay. Crucially, only desalted oligonucleotides were used here and no post synthesis DNA correction methods were required for the assembly method. Without TOR selection, the probability of producing an intact *gfp* gene using desalted, 60 nt oligonucleotides is expected^26^,^27^ to be 0.93^22^ = 20%, rather than the 76–79.5% observed with TOR. Here, 0.93 is the sequence accuracy estimated for the oligonucleotides used in this work (see below and Supplementary Fig. S6), and 22 is the number of oligonucleotides necessary for *gfp* assembly. This value is comparable with that determined experimentally, both in a previous study^20^ (~15%) and here: ~35% colonies per TOR cloning are blue of which 79.5% are correct resulting in an accuracy of 27%.

### False positives in TOR

The analysis of sequence data from the PCA and RapGene samples revealed that false positives were present in each of the five independent assays for *gfp* assembly (Table 1). Approximately 2% of the constructs contained a single missense mutation, and an additional 2% showed in-frame deletions or insertions within the CDS. TOR cannot eliminate these types of false positives which are however very rare. We noticed that a potential second class of false positives could be observed after prolonged growth at 37 °C. In fact, after extended incubation times several colonies among the negative samples developed a light-green colony colour (Fig. 4c). To investigate this observation further, the fifteen negative samples previously sequenced were studied in more detail (Fig. 4a,b). These defective *gfp* genes were all expressed from the P_recA_* promoter. Eleven samples showed a light-green colony colour when incubated on CIX medium (LB agar plus carbenicillin [120 μg/ml], IPTG [1 mM] and X-Gal [0.0001%]) for up to 55 hours at 37 °C. In particular, sample *gfp-ins*, which sequence analysis revealed to possess a single insertion mutation after position 183 of the coding sequence, developed a strong green colour after 48 hours of incubation at 37 °C (Supplementary Fig. S7). However, a difference between *gfp-ins* and wild-type *gfp* was still evident at this timepoint, especially when cells expressing these mutants were observed as single colonies. All of the sequenced negative samples possessed at least one stop codon within *gfp*, but colony colour development differed significantly. Therefore, we excluded that colony darkening was due to inefficiency of T1 as a transcription terminator in this genetic context. To test whether the preceding false positives expressed from the P_recA_* promoter behaved similarly when expressed from alternative promoters, a plate assay was performed using the same *aadA1* and *gfp* mutants described in Fig. 3a, but expressed under the control of P_*BAD*_ or P_*tac*_ (Supplementary Fig. S8). Only *gfp-ins* expressed from P_*tac*_ produced an indistinguishable blue colour compared to wild-type *gfp*. Therefore, although high level of expression of the reporter may produce false-positives such as *gfp-ins*, sequencing data for P_*tac*_ transformants showed that the latter is still a reliable and “fast” TOR promoter (73% accuracy; Table 1, Supplementary Data 1), with false positives from the second class being negligible (accounting for only one out of the 73 TOR-positive samples sequenced, i.e. ~1%). While we cannot currently explain the light-green colour of some of the negative samples, we suggest that translation re-initiation at cryptic ribosome binding sites within the CDS may account for this effect. Ribosome recruitment at internal sites has been recently reported to be more common than expected, (not only for heterologous gene expression), as a mechanism that can affect the rate of transcription more than the codon usage^28^.

In TOR, a cryptic RBS-ATG/GTG pair located on frame two or three could also be involved in this effect. In fact, as a result of a frame-shift, a short sequence upstream of the mutation site on either of these frames will become in-frame with the CDS.

An appropriate codon optimization of the synthetic gene - common practice in gene synthesis - could avoid the emergence of a cryptic RBS, and likely exclude the possibility of weak expression of the reporter gene in TOR-negative samples.

### Accuracy of the oligonucleotides used in this study

Long deletions were occasionally observed in constructs following PCA cloning (Supplementary Data 1). As the inserts amplified by PCA were always gel extracted, these mutations were likely the result of *in vivo* DNA rearrangement events. To quantify this effect, the NheI-AfeII fragment bearing the wild-type *gfp* sequence construct in pRG2 under the control of P_recA_* was amplified and cloned back into the same vector by the Gibson procedure (Supplementary Fig. S6). Transformants were plated both on LB plus Carbenicillin and on CIX plates. Thirty colonies that appeared white on the former were chosen randomly and screened by PCR to identify possible DNA deletions. No rearrangements were observed (Supplementary Fig. S6a). Transformants plated on CIX mostly developed into colonies with a blue colour, but 10.6% (15/141) were white (Supplementary Fig. S6b). Ten samples randomly chosen among the latter were sequenced and mutations were identified only in the sequence of the reverse oligonucleotide used for PCR amplification (Supplementary Fig. S6c). This assay confirmed that the background level of *in vivo* rearrangements observed in this study was very low, and also provides an accurate quantification of the sequence accuracy associated with the oligonucleotides used in this study (approx. 93%). Typical accuracy levels for desalted 60 nt oligonucleotides may be as low as 60%. However, a much higher percentage of oligonucleotides (93% determined here) can be considered correct for selected applications such as TOR as several types of 5′ deletions appear to be tolerated by this assembly method, as oligonucleotide gaps are filled *in vivo*.

### Colony colour development in TOR depends on codon usage

Whereas colony colour development for positive samples was a function of the incubation time in TOR, the colour conferred by the *gfp* gene on CIX plates was intrinsically less intense than that produced by *aadA1* (sample *Sm-wt*, Fig. 3a; Supplementary Fig. 8a). Wright and Hayward noted that when a terminator was in frame with the CDS, events expected to slow down the movement of ribosomes on the mRNA, e.g., binding of fusidic acid, allowed restoration of termination^16^. Based on this principle, a *gfp* mutant containing several rare codons was predicted to confer a brighter green colour on CIX plates compared to wild-type *gfp*. In contrast, the co-expression of rare tRNAs from plasmid pRARE2 (see Methods) in a strain expressing a *gfp* with rare codons, was expected to rescue the dark colony colour. These predictions were confirmed (Supplementary Figs. S9 and S10). As rare codons are expected to slow down the movement of the ribosomes on the transcript, fewer ribosomes will be able to populate the downstream mRNA region. Thus here, a weak but detectable transcription termination at T1 would be observed, and colony colour intensity would decrease even in the absence of a frame-shift mutation.

## Discussion

In this work we have described and characterized TOR, a screening strategy that employs a universal reporter assay for the quick and cost-effective identification of correctly assembled synthetic protein-coding genes. The approach relies on the conditional activation of a transcription terminator in response to sequence errors originating at the assembly step. The effect of TOR-based selection was significant as almost 80% of approx. 1 kb sequences selected by TOR were fully correct. Thus, no more than two transformants per cloning experiment need to be sequenced with this method to identify a correctly assembled 1 kb synthetic gene. Crucially, the technology allows the quick synthesis of standard length genes at affordable prices, as only desalted oligonucleotides are used to achieve the same level of accuracy of more complex industrial procedures. In addition, the predicted yields of approximately 50% for a 2 kb assembly would allow the commercially viable synthesis of even longer synthetic genes in a one-pot assay, especially when the TOR platform was coupled with current methods used in gene synthesis, e.g., highly purified oligonucleotides and enzymatic post-assembly error correction methods. Fragments of 2–3 kb are typically close to the upper limit for one-pot gene synthesis approaches, as yields for these long constructs may drop easily below values of commercial interest.

The results achieved by TOR are relevant for several reasons. First, as low-quality desalted oligonucleotides are not a limiting factor in achieving high accuracy by the TOR method, this technology is an efficient alternative option to third party gene synthesis services, especially if a simple in-house oligonucleotide synthesizer was available. Use of TOR in industrial settings will be an additional tool to improve yields and reduce gene synthesis costs by helping to overcome current limitations associated with the maximum gene length that is commercially viable. Moreover, the need for more complex techniques are avoided when accuracy levels similar to those obtained by the TOR method are sufficient.

Second, the potential use of reporter genes made up of entire metabolic pathways will further enhance the sensitivity and flexibility of the method allowing, for example, detection of very low expression levels for the reporter, and so also the synthesis of toxic genes. In fact, as TOR relies on the expression of the synthetic gene of interest, this method would not be suitable for the assembly of toxic proteins, although, as shown here for P_*BAD*_ driven expression, TOR is also effective under low expression levels that could be compatible with toxic effects. Moreover, the use of an antibiotic resistance marker as a reporter gene, will allow only positive clones to grow on appropriate antibiotic-containing plates. This is important when longer synthetic genes are assembled, as the lower yields expected with extended constructs could require too many colonies to be grown on the same plate.

Third, due to its intrinsic ability to select for full-length CDS mutants derived from random mutagenesis, the TOR platform could be explored for additional applications including directed evolution or phage-display-like approaches.

Thus, the TOR gene synthesis method described here is a fast, cost effective and accurate gene assembly platform for improved applications in synthetic biology.

## Methods

### Plasmids and strains

Plasmid pRG2 (Fig. 2) is a derivative of pRG1.0^19^. It features a wild-type *lacI* promoter and a novel reporter system which consists of two elements introduced just upstream of the toxic *Gata1* gene: (i) the efficient *E. coli* T1 transcription terminator^17^ designed to be in-frame with the synthetic gene upon cloning, followed by (ii) the *lacZ*α gene and native RBS from the cloning vector pUC19^29^. The *lacZ*α gene encodes the α-fragment derived from β-galactosidase that is employed in blue-white screening for cloning in *E. coli*^30^. Since pRG2 was designed as an extension of the RapGene assembly method^20^, the P_*sm*_ promoter located upstream of the NheI restriction site in pRG1.0 is absent in plasmid pRG2. In fact the assembly of a >600 bp synthetic element by RapGene was associated with the generation of insert-free re-ligated vectors^20^ and the presence of a plasmid-encoded promoter in pRG2 could easily produce false positive results due to the expression of the *lacZ*α from P_*sm*_. Thus, instead of P_*sm*_, variable promoters were introduced in pRG2 as part of the synthetic insert generated from several oligonucleotides. Plasmid pRG2 was produced by Gibson assembly from four DNA fragments derived from pGATA, pUC19 (*lacZ*α) and a six oligonucleotide generated PCA fragment (λtI-T1). The T1 terminator sequence^17^ was derived from the Registry of Standard Biological Parts (http://parts.igem.org/Main_Page) (part:BBa_B0010) and was placed downstream of the λ*tI* terminator^31^. This latter was oriented in the direction of *Gata1* to avoid the possibility that genetic manipulation upstream of *Gata1* (in the T1-*lacZ*α region) might introduce a promoter-like sequence inducing constitutive expression of the toxic GATA-1. *E. coli* NEB5α (New England Biolabs) was used throughout the study. Plasmid pRARE2 was purified from *E. coli* strain Rosetta™ 2(DE3) (Merck Millipore) and used subsequently in the NEB5α strain.

### TOR accuracy determination

When determining the error rate in TOR, full-length inserts were arbitrarily defined as those samples showing deletions/insertions smaller than 15 bp, i.e., 25% of the average size oligonucleotide used in the assembly.

### DNA assembly and mutagenic strategies

Recombinant DNA was generated mainly by the Gibson Assembly Cloning Kit (New England Biolabs) following the manufacturer’s instructions. For PCA assembly of *gfp* constructs, plasmid pRG2 was simultaneously digested with NheI and AflII for 1–2 hours at 37 °C. After spin column purification the vector was used directly for cloning. For RapGene assembly of *gfp* constructs, T4 DNA polymerase and 1 mM dTTPs were also added to the digestion mixture and cloning was performed as previously described^19^. PCA assays were performed with Phusion DNA polymerase (New England Biolabs) using oligonucleotides that were 60 nt in length on average. One microliter of each oligonucleotide (from a 100 μM stock) was added to 1 ml 10 mM Tris, 50 mM NaCl (pH 8.0). One microliter of this mix was used as template in a 50 μl PCR reaction containing 0.25 μM external primers, Phusion DNA polymerase (1U/50 μl), 200 μM each dNTPs and HF Phusion buffer. Amplification primers were the same external oligonucleotides used in the assembly. PCA amplification of *gfp* required up to 50 cycles of PCR at constant annealing temperature (65 °C). However, in the first round of PCA amplification (Table 1) annealing temperatures varied from 70 °C to 50 °C in steps of 5 °C per 10 cycles. The amplified PCR product was purified by gel extraction before cloning with the Gibson Assembly Cloning Kit using *E. coli* NEB5α chemically competent cells.

### CIX plates

Screening assays were performed on CIX plates (LB agar plus carbenicillin [120 μg/ml], IPTG [1 mM] and X-Gal [0.0001%]). Plates were stored at 5 °C and used within 10 days of preparation. Colour development on CIX plates (Supplementary Fig. S11) required unexpectedly the presence of IPTG, despite pRG2 lacking any *lac* operator-like sequences. The very low expression of *lacZ*α observed here – typically about 10 Miller units for the strongest constructs (Supplementary Fig. S10) - coupled with LacI overexpression could possibly account for the observed silencing effect^32^ (i.e. all site-specific DNA-binding proteins bind non-specifically although with low affinity to any DNA sequence^33^). The low levels of *lacZ*α activity may also suggest that T1 is still partly active in termination, even when fully translated.

### Oligonucleotides and sequencing

Sigma-Aldrich was the supplier for oligonucleotides (Supplementary Table S1) which were obtained at the lowest purification level (desalted), re-suspended in sterile 10 mM Tris-HCl pH 8.0, 0.1 mM EDTA, and stored at −80 °C. Sequencing reactions were performed at GATC-Biotech (Germany).

### ONPG assay

Bacteria were grown on LB agar plus Carbenicillin [120 μg/ml], IPTG [1 mM] (without X-Gal) for 48 hours. From these plates, suspensions were prepared (OD_600_ = 0.2) in 3ml NaCl 0.9%. Then 100 μl of these samples were mixed with 900 μl Z-buffer [60 mM Na_2_HPO_4_, 40 mM NaH_2_PO_4_, 10 mM KCl, 1 mM MgSO_4_, plus β-mercaptoethanol (50 mM, freshly added)] in 15 ml Falcon tubes. After adding 20 μl of CHCl_3_, 20 μl 0.1% SDS (sodium dodecyl sulphate) and 200 μl ONPG (ortho-Nitrophenyl-β-galactoside) [4 mg/ml in water], samples were vortexed and incubated at 28 °C in a waterbath for 55 hours, before stopping the reaction with 500 μl 1 M Na_2_CO_3_. OD_600_ was measured for all the samples at 420 nm and 550 nm, against a blank made of the same buffer plus 200 μl of NaCl 0.9%. The negative controls consisted of NEB5α transformed with either pRG2 or pRG2 plus pRARE2. Miller Units (MU) were determined using the formula 1000 × (OD_420_ − [1.75* OD_550_])/(t* v* OD_600_) (t = minutes, v = millilitres). MU from the controls were subtracted from MU of the samples.

## Additional Information

**How to cite this article**: Zampini, M. *et al.* Terminator Operon Reporter: combining a transcription termination switch with reporter technology for improved gene synthesis and synthetic biology applications. *Sci. Rep.* **6**, 26572; doi: 10.1038/srep26572 (2016).

## Supplementary Material

Supplementary Information

Supplementary Data 1

Supplementary Data 2

## Figures and Tables

**Table 1:** Efficiency of TOR gene synthesis for the assembly of *gfp* through PCA or RapGene methods.

1		2	3	4	5	6
		Number of samples sequenced	Number and % of samples with fully correct *gfp* CDS	Number and % of samples with deletions/ insertions in *gfp* CDS	Number and % of samples with substitutions in *gfp* CDS	Error rate
1^st^ round	1^st^ PCA assembly (P_recA_*)	15	10 (10/15 = **66%**)	2 (**13%**)	2 (**13%**)	0.022
	1^st^ RapGene assembly (P_recA_*)	13	8 (8/13 = **61%**)	3 (**23%**)	2 (**15%**)	0.136
2^nd^ round	2^nd^ PCA assembly (P_recA_*)	15	14 (**93%**)	1 (**6%**)	1 (**6%**)	0.009
	2^nd^ PCA assembly (P_*BAD*_)	15	13 (**86%**)	2 (**13%**)	0 (**0%**)	0.045
	2^nd^ PCA assembly (P_*tac*_)	15	11 (**73%**)	2 (**13%**)	3 (**20%**)	0.020

A total of 73 TOR-positive samples derived from one round of RapGene and four rounds of PCA assembly (in the latter case using either P_recA_*, P_*tac*_ or P_*BAD*_) were sequence verified.
