# Supplementary material for: Terminator Operon Reporter: combining a transcription termination switch with reporter technology for improved gene synthesis and synthetic biology applications
Source: Sci Rep. 2016 May 25;6:26572. doi: 10.1038/srep26572 (PMC4879669; doi:10.1038/srep26572)
Supplement: Supplementary Information [file srep26572-s1.pdf]

Supplementary Information

Terminator Operon Reporter: combining a transcription termination switch with reporter technology for improved gene synthesis and synthetic biology applications

Massimiliano Zampini[1*], Luis A J Mur[1], Pauline Rees Stevens[1], Justin A Pachebat[1], C James Newbold[1], Finbarr Hayes[2*] & Alison Kingston-Smith[1*]

[1] Institute of Biological, Environmental and Rural Sciences, Edward Llwyd Building, Aberystwyth University, Aberystwyth SY23 3FG, UK, [2] Faculty of Life Sciences, University of Manchester, Manchester M13 9PL, UK

*Correspondence should be addressed to M.Z. (M.Zampini@outlook.com) or F.H. (Finbarr.Hayes@manchester.ac.uk) or A. K-S. (ahk@aber.ac.uk)

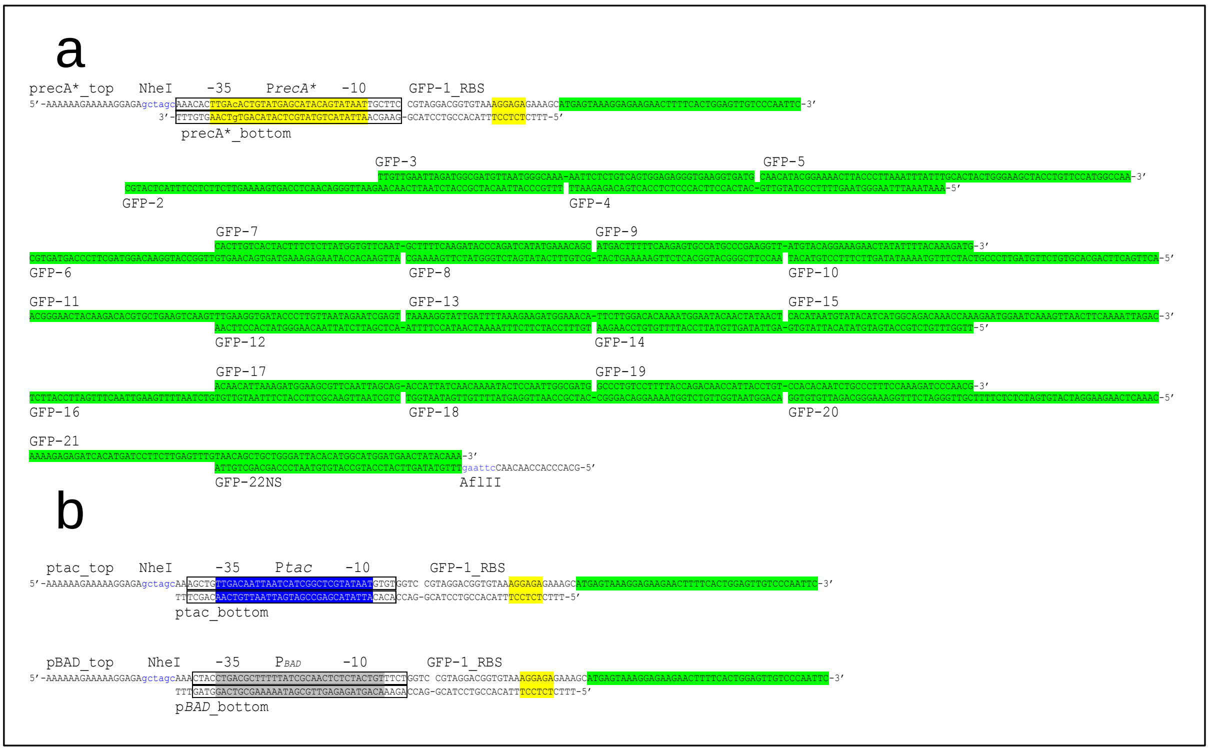

Supplementary Figure S1. Pool of oligonucleotides used to build *gfp* inserts by either RapGene or PCA. P_{recA*} promoter was used for RapGene constructs. Either P_{recA*}, P_{tac} or P_{BAD} promoters were used for PCA constructs. (a) Oligonucleotides used for the assembly of the *gfp-wt* construct including the P_{recA*} promoter region (highlighted in yellow). (b) Promoter regions for constructs generated by PCA in which *gfp* was under the expression of either P_{tac} (highlighted in blue) or P_{BAD} (highlighted in grey) core promoter. For both panels: precA*_top, precA*_bottomm, GFP-1_RBS, GFP-2 to GFP-21, ptac_top, ptac_bottom, pBAD_top, pBAD_bottom are the name of the oligonucleotides. NheI restriction site NheI. P_{recA*}, P_{recA*} promoter. P_{tac}, P_{tac} promoter. P_{BAD}, P_{BAD} promoter. The sequence highlighted in yellow downstream the promoters is the RBS.

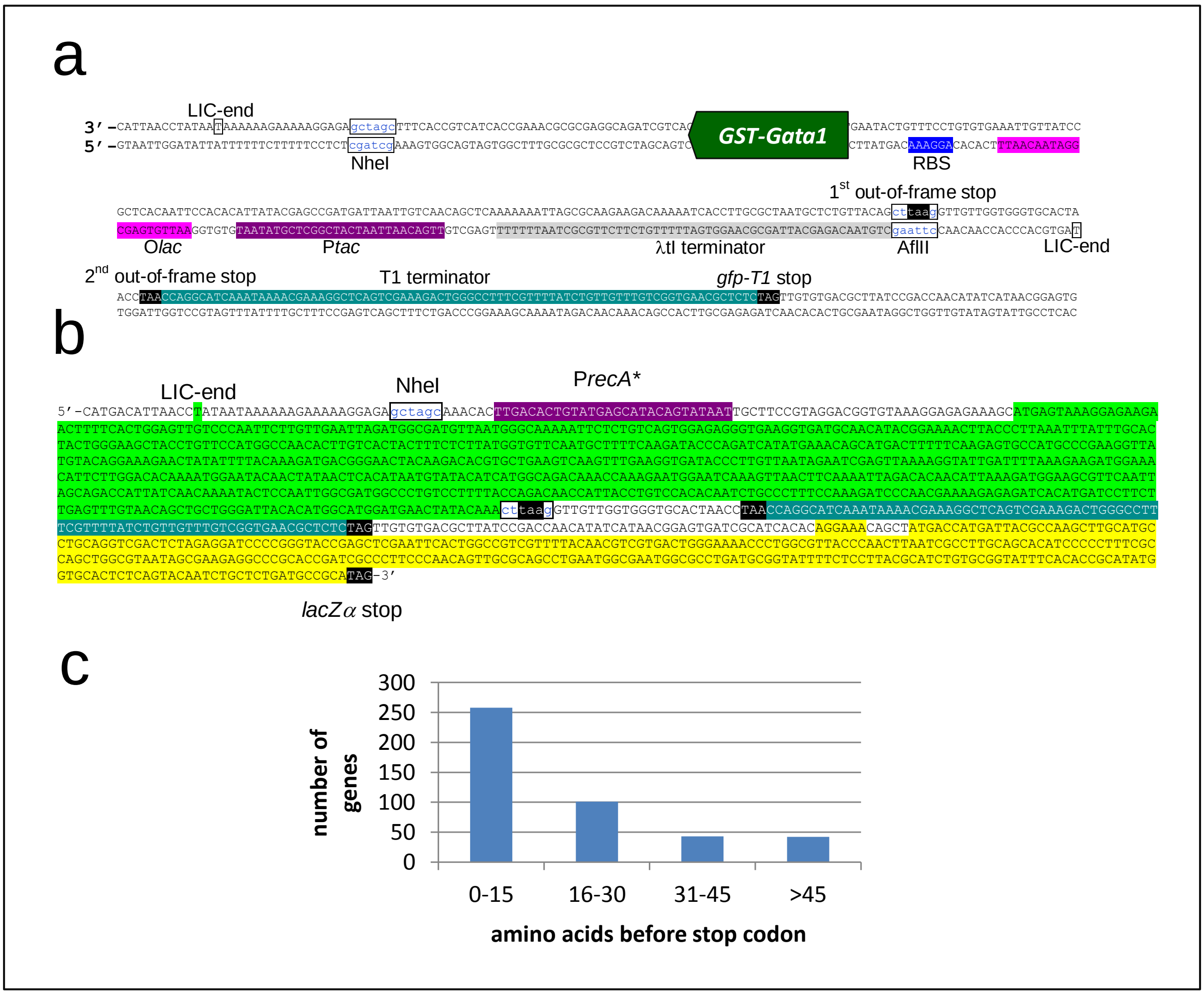

Supplementary Figure S2. (a) Sequence of pRG2 spanning *Gata1* and T1. (b). Corresponding region of pRG2-*gfp-wt* (under P_{recA*}), after cloning *gfp* and its P_{recA*} promoter between NheI and AflII sites of pRG2. (c) The insertion of a single base (adenine) after position 99 in the CDS of the first ~450 genes from *E. Coli* MG1655 genome (GenBank NC_000913.3) produces a stop codon after a variable number of corresponding amino acids (on average 18), whose distribution is reported in the histogram. For both panels: LIC-end, T base at which T4 DNA polymerase digestion stops (leaving 3'-recessed ends upstream NheI). NheI, restriction site for NheI. *GST-Gata1*, toxic gene derived from pGATA. RBS, ribosome binding site. O*lac*, lac operator. P_{tac}, tac promoter. λtI, tI terminator from lambda phage. 1st out-of-frame stop, stop codon on frame 3 relative to *gfp* wt sequence. AflII, restriction site for AflII. 2nd out-of-frame stop, stop codon on frame 2, relative to *gfp* wt sequence. T1 terminator, sequence of T1 terminator. *gfp-T1* stop, stop codon for the fusion *gfp-T1*. Sequences highlighted in green and in yellow in (b) are *gfp* wild type and *lacZα* sequences, respectively. *lacZα* stop, stop codon for *lacZα*. P_{recA*}, sequence of P_{recA*} promoter.

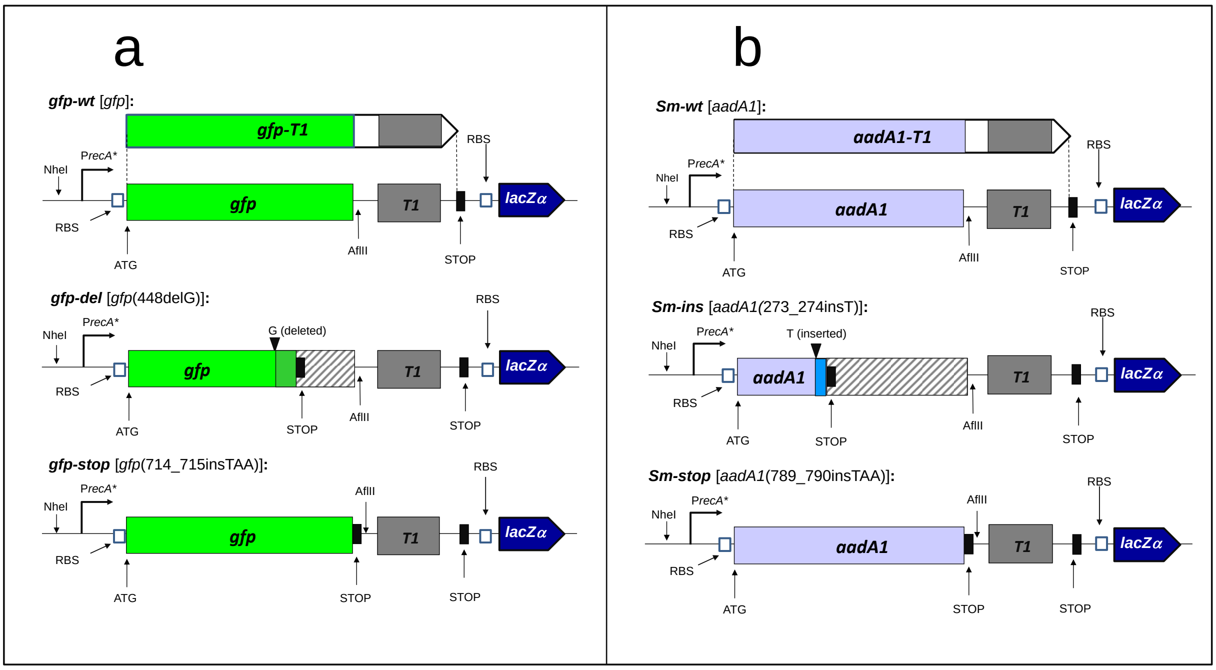

Supplementary Figure S3. Structure of the six control constructs used to test the TOR platform (not to scale): (a) *gfp* wild-type and mutants. *gfp-wt* is the wild-type *gfp* sequence, with no stop codon upstream of T1. *gfp-del* contains a single point deletion (G) after base 448. *gfp-stop* contains a stop codon (TAA) inserted after the last *gfp* codon at position 715. (b) *aadA1* wild-type and mutants. *Sm*-wt is the wild-type *aadA1* sequence, with no stop codon upstream of T1. *Sm-ins* contains a single point insertion at position 273. *Sm-stop* contains a stop codon (TAA) inserted after the last *aadA* codon at position 790. Base numbering refers to the wild-type sequence of either *gfp* or *aadA1*. These six linear fragments were cloned between the NheI and AflII sites of pRG2. RBS, ribosome binding site. *gfp*, *gfp* CDS. Light-green boxes, *gfp* CDS. Dark green boxes in *gfp-del*, extended CDS derived from the frame-shift introduced by the mutation. *aadA1*, *aadA1* CDS. T1, transcription terminator T1. *lacZα*, *lacZα* CDS. ATG, translation start. STOP, translation stop. *gfp-T1*, translational fusion *gfp-T1*. *aadA1*-T1, translational fusion *aadA1-T1*. Light-blue boxes, *aadA1* coding sequence. Dark blue boxes in *Sm-ins*, extended CDS derived from the frame-shift introduced by the mutation.

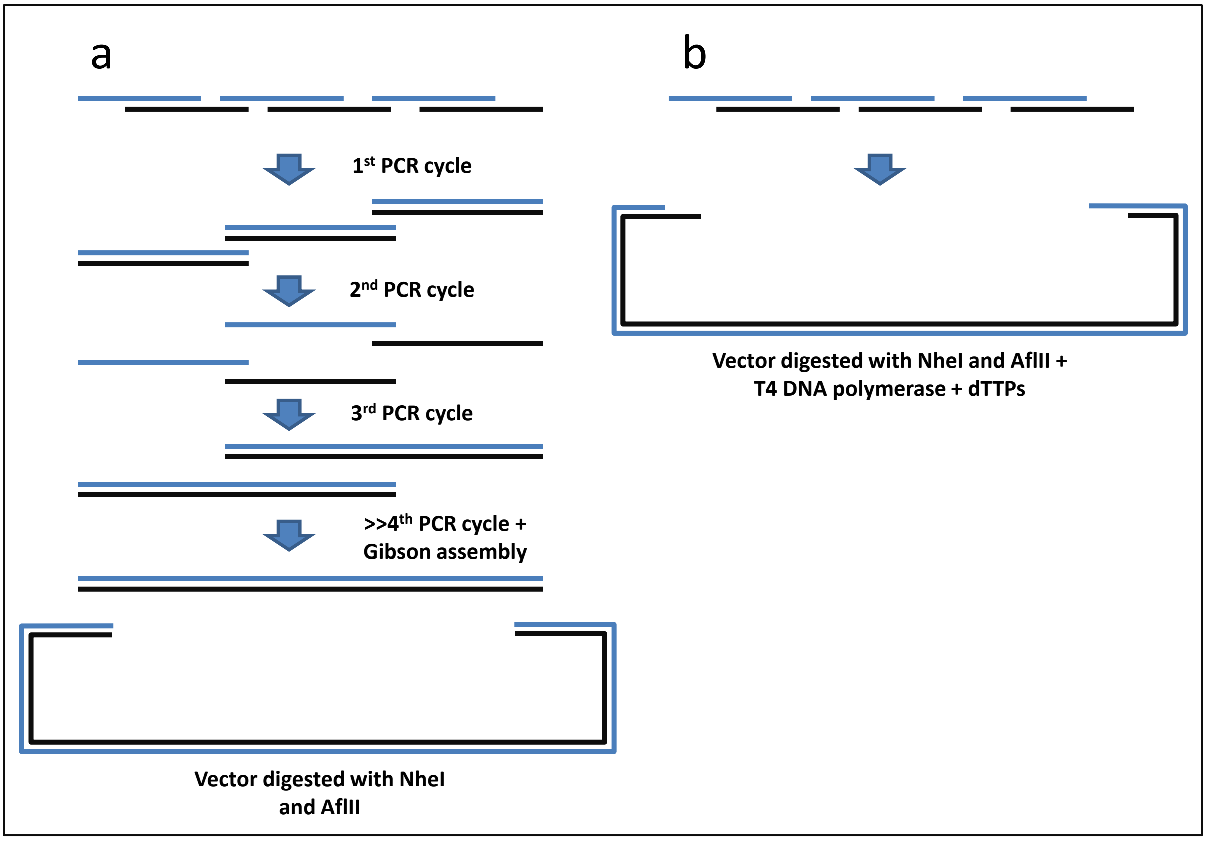

Supplementary Figure S4. Comparison between PCA and RapGene assembly methods. (a) PCA requires several steps of PCR to generate overlapping fragments that are longer at each cycle. In this study the final product *gfp* is gel extracted and cloned into the vector pRG2 digested with NheI and AflII, by the Gibson Assembly method. (b) RapGene requires an initial annealing step where oligonucleotides are annealed together without ligation, and a second step in which this non-ligated and non-gel-extracted insert is annealed to a LIC-vector (LIC= Ligation Independent Cloning), produced here by digestion of pRG2 with NheI and AflII, in the presence of T4 DNA polymerase and dTTPs.

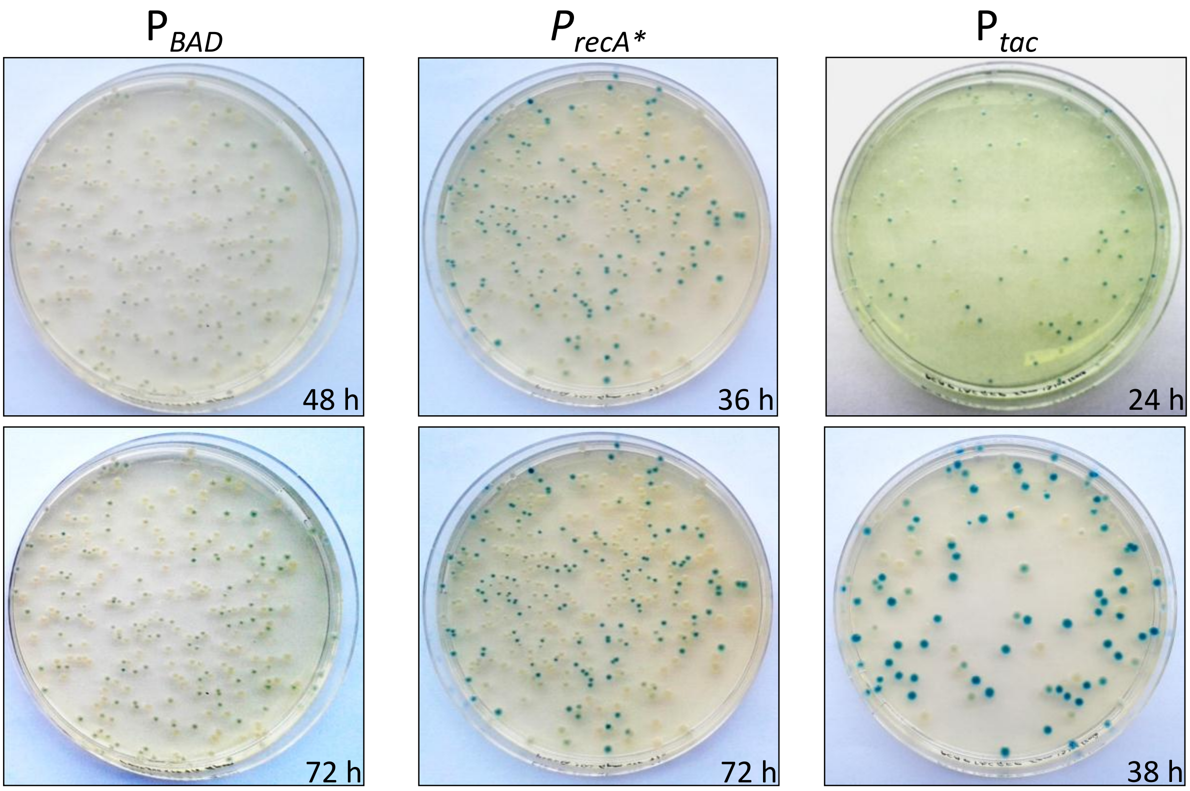

Supplementary Figure S5. PCA-cloning with TOR, using the three different promoters P_{recA*}, P_{BAD} and P_{tac}. Sequenced samples were chosen among these candidates.

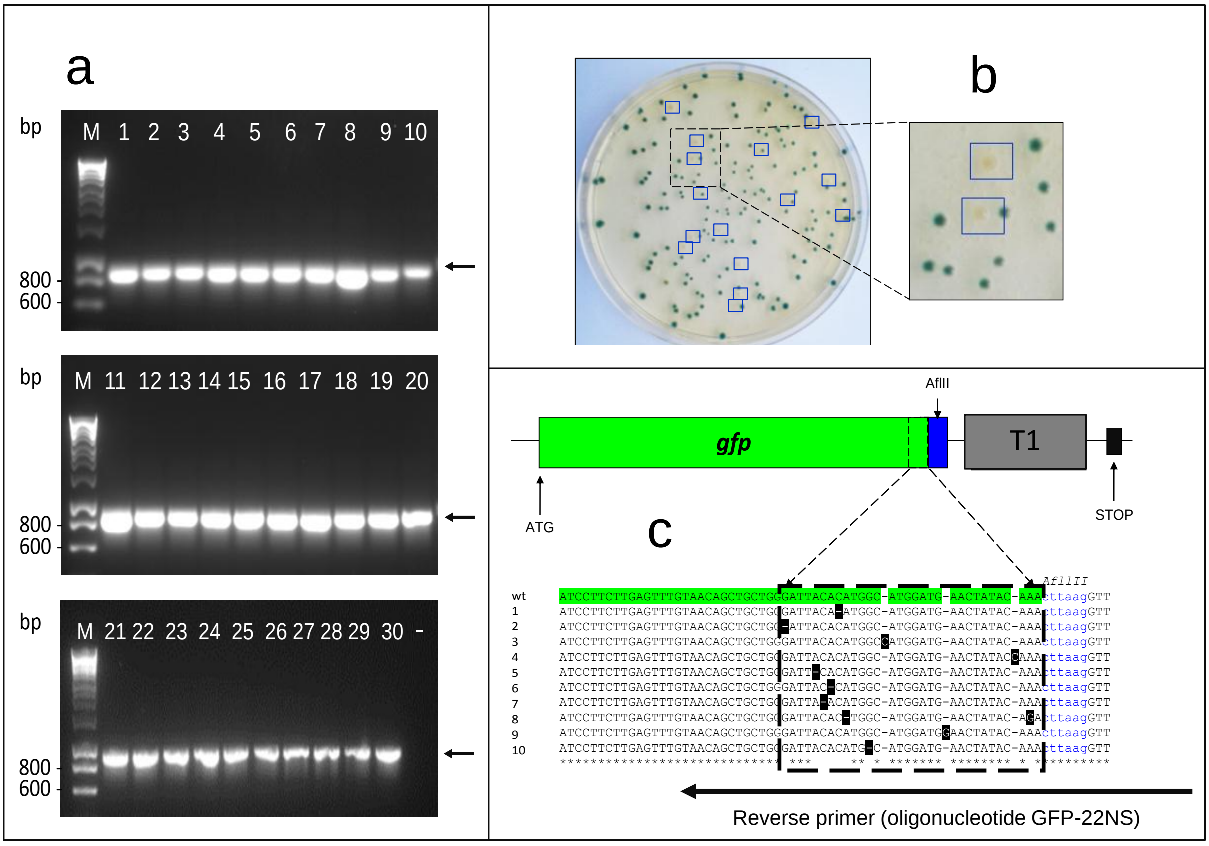

Supplementary Figure S6. Sequence accuracy of the oligonucleotides used in this study. (a) A PCR amplicon obtained using *gfp-wt* (under P_{recA*}) as template and primers *precA**_top and GFP-22NS (Supplementary Table S1), was cloned into pRG2 by Gibson assembly. After transformation the overgrown culture was plated both on LB plus Carbenicillin and on CIX plates. Thirty samples isolated randomly from the LB plate (no colour development as X-Gal was omitted) were subject to colony PCR. (b) Fifteen out of the 141 colonies isolated on CIX showed a clear phenotype. (c) Ten among the negative samples shown in (b) were sequenced. All of them carried mutations within the *gfp* CDS, along the sequence of primer GFP-22NS.

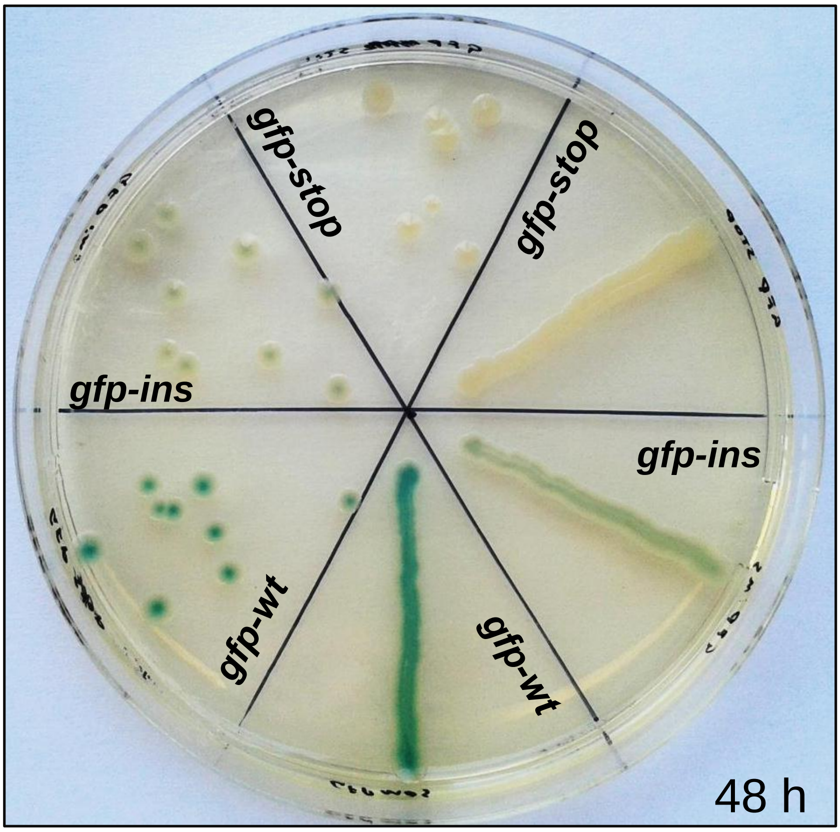

Supplementary Figure S7. Colour development conferred by *gfp-ins* (P_{recA*}). Colour development associated with *gfp-ins* was compared to that of *gfp* wild-type (*gfp-wt*, positive control) and *gfp-stop* (negative control) both as colonies and as streaked samples. While darkening of *gfp-ins* was evident after ~48 hours incubation at 37°C, the difference between this sample and the positive control remained clearly visible. *gfp-ins*, pRG2 harbouring *gfp* with a single insertion after position 183. *gfp-stop*, pRG2 harbouring *gfp* with a stop codon after the last codon. All constructs were expressed under P_{recA*}. Numbering refers to *gfp* coding sequence.

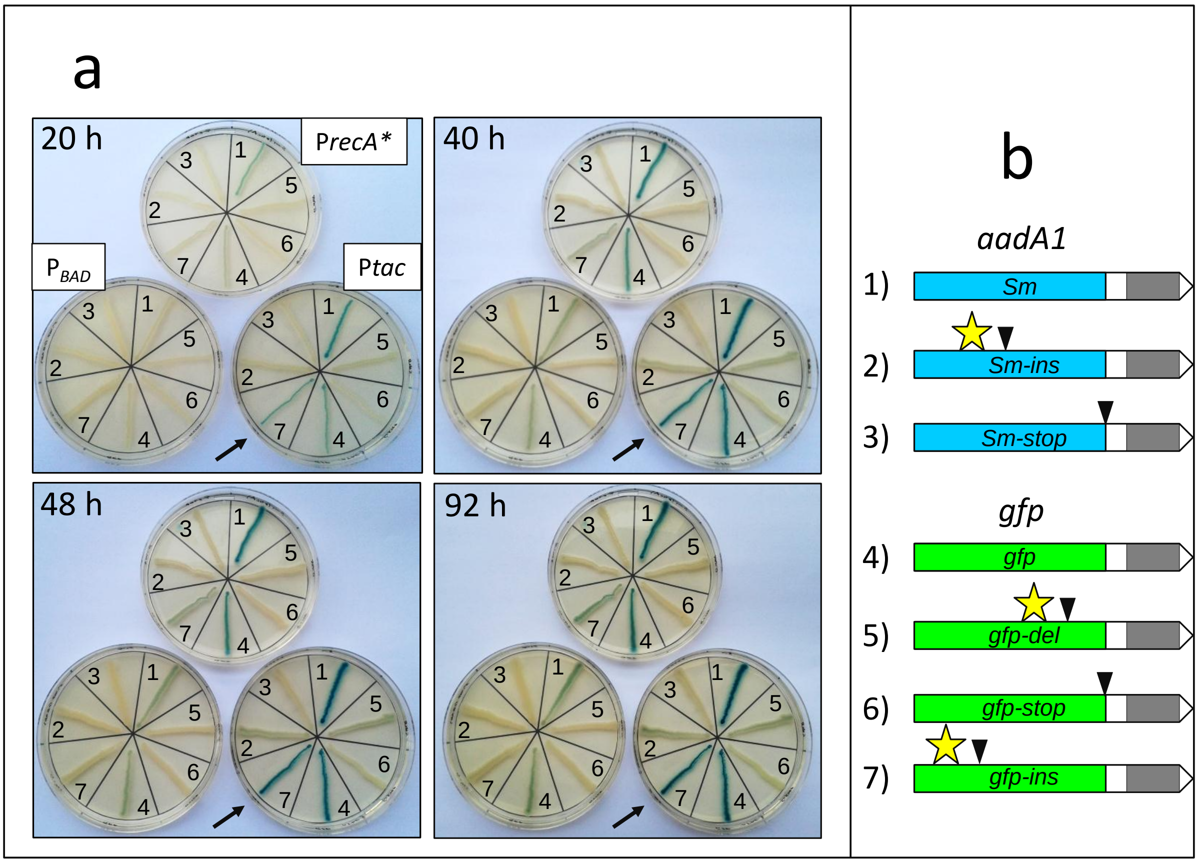

Supplementary Figure S8. (a) *aadA1*, *gfp*, and their nonsense mutants tested on CIX plates under different promoters (P_{recA*}, P_{BAD}, P_{tac}). Arrows indicate *gfp-ins*. (b) legend.

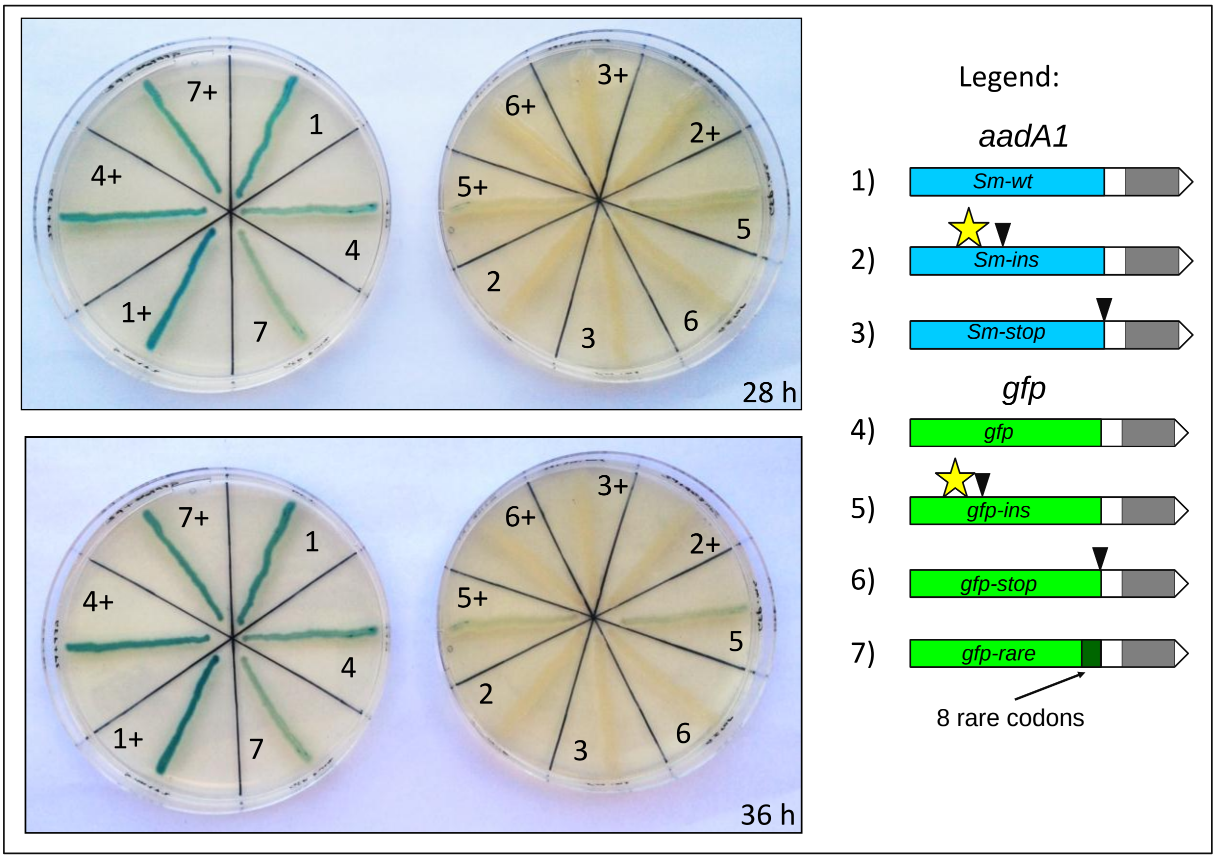

Supplementary Figure S9. Colour development is a function of codon usage. This figure shows the effect of pRARE2 (encoding tRNAs for *E. coli* rare codons) on the colour development associated with *aadA1* and *gfp* wild-type and mutants (expression from P_{recA*}). pRARE2 increases the colour intensity on CIX plates, this explaining the darker colour conferred by *aadA1 as* compared to wild-type *gfp*. The *gfp-rare* mutant was designed so that the last eight codons in *gfp* were replaced by eight consecutive rare codons in *E. coli* (AGG, AGA, CGG, CGA, GGA, ATA, CTA, CCC). This sample and those presented in Figure 3a were streaked on CIX plates together with identical samples co-transformed with pRARE2. Each pRG2 derivative and the same derivative co-transformed with pRARE2 occupy opposite positions on the plates. The latter are indicated by the + symbols. Sample *gfp-rare*, and to a lesser extent also *Sm-wt* and *gfp-wt*, produced a brighter colony colour compared to the equivalent mutants co-transformed with pRARE2. No difference was visible between all the other pairs of mutant each of which carried at least one stop codon upstream of the T1 terminator.

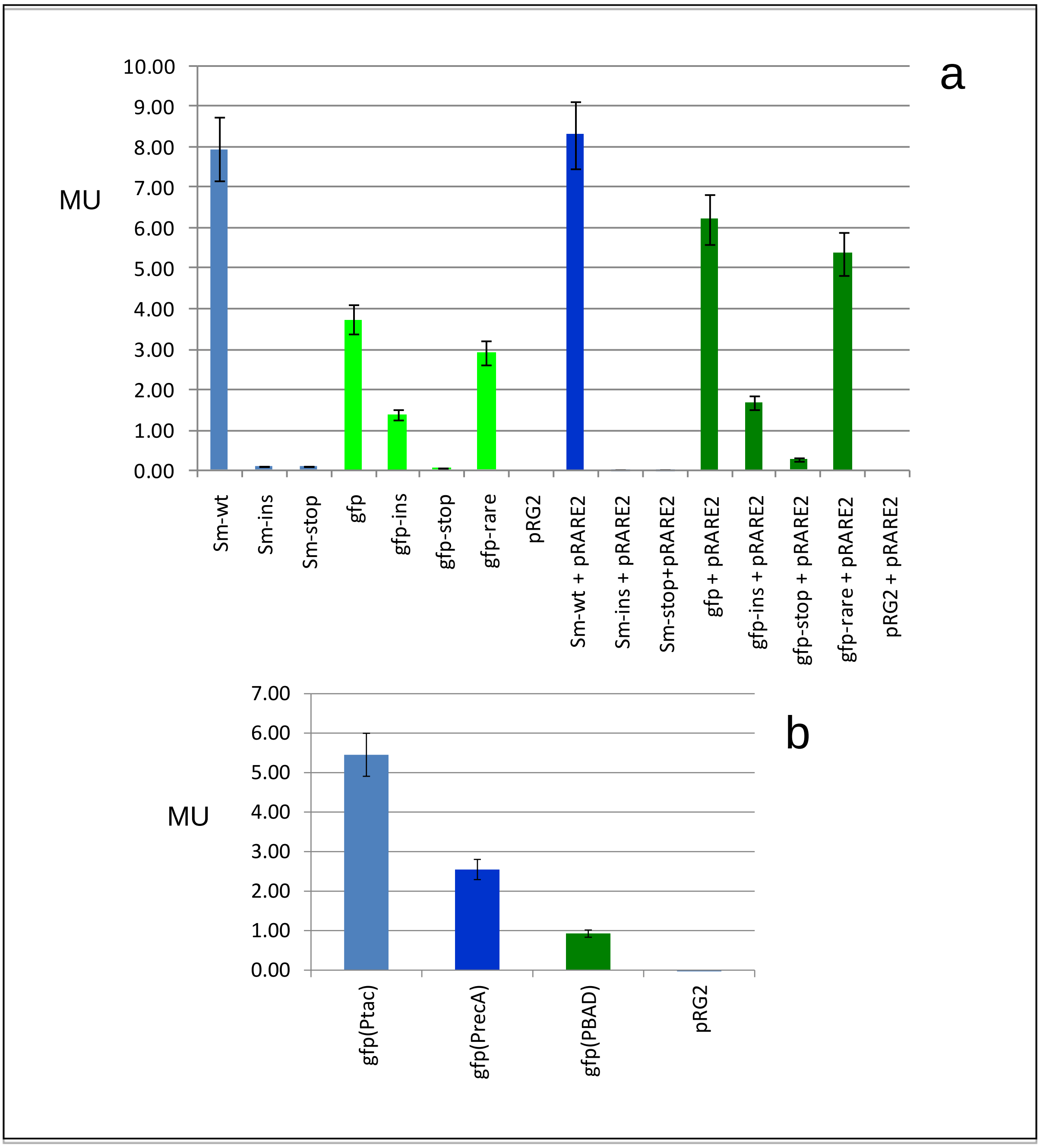

Supplementary Figure S10. ONPG assay. (a) Colour intensity for samples described in Supplementary Figure S9 were determined by the ONPG assay (see Methods). The presence of plasmid pRARE2, carrying tRNAs for codons rare in *E. coli*, visibly increases colour intensity for samples *gfp* and *gfp-rare*. Consistently with the plate assay (Supplementary Fig. 9), pRARE2 did not improved significantly colour development for *Sm-wt*. (b) ONPG assay was also performed of *gfp-wt* samples expressed under either one of the three different promoters P*tac*, P*recA** and P_{BAD}. As from Supplementary Figure S5 the strengths of these three promoters followed the order P*tac*, P*recA** and $P_{BAD.}$

*V*alues represent means ± SD of the results of at least three independent experiments. MU, Miller Units.

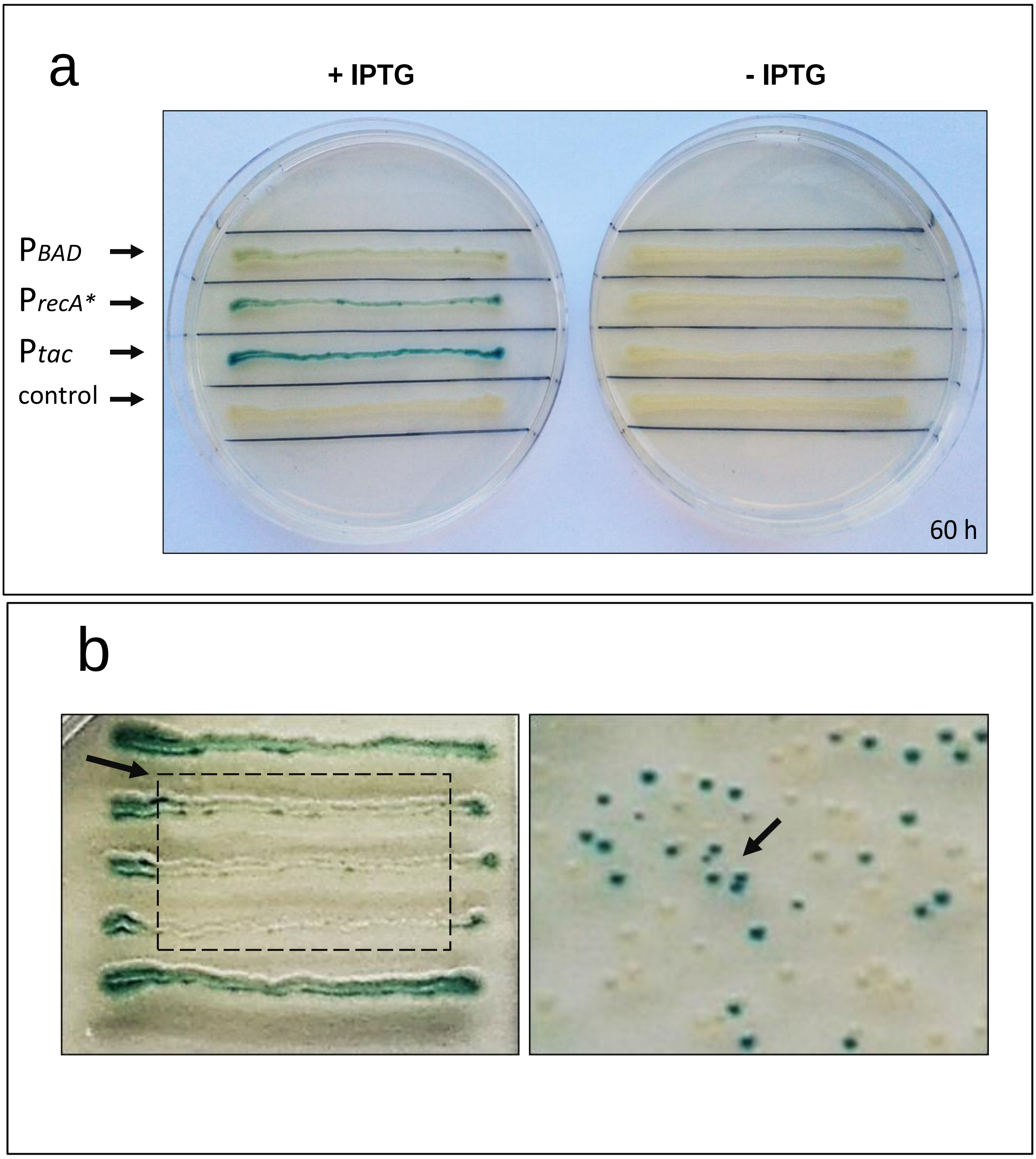

Supplementary Figure S11. (a) Effect of IPTG on colour development for *gfp* constructs expressed from either P_{BAD}, P_{recA*} or P_{tac} promoters. On plates containing X-Gal only (no IPTG, right) bacteria did not develop the typical blue colour. (b) When screening for phenotype by streaking samples on CIX plates, streaks should be spaced at least 1 cm apart to reduce growth and colour inhibition (boxed area, arrow). However colour inhibition is not observed when transformants grow as single colonies on the same medium, even when in close proximity (right panel, arrow).

Name	Sequence (5'-3')	Restriction sites	Signals
**precA*_top**	AAAAAAGAAAAAGGAGAGCTAGCAAACACTTGACACTGTATGAGCATACAGTATAATTGCTTC	NheI	P_{recA*}
**precA*_bottom**	TTTCTCTCCTTTACACCGTCCTACGGAAGCAATTATACTGTATGCTCATACAGTGTCAAGTGTTT		P_{recA*}
**GFP-1_RBS**	CGTAGGACGGTGTAAAGGAGAGAAAGCATGAGTAAAGGAGAAGAACTTTTCACTGGAGTTGTCCCAATTC		RBS
**ptac_top**	AAAAAAGAAAAAGGAGAGCTAGCAAAGCTGTTGACAATTAATCATCGGCTCGTATAATGTGTGGTC		P_{tac}
**ptac_bottom**	TTTCTCTCCTTTACACCGTCCTACGGACCACACATTATACGAGCCGATGATTAATTGTCAACAGCTTT		P_{tac}
**pBAD_top**	AAAAAAGAAAAAGGAGAGCTAGCAAACTACCTGACGCTTTTTATCGCAACTCTCTACTGTTTCTGGTC		P_{BAD}
**pBAD_bottom**	TTTCTCTCCTTTACACCGTCCTACGGACCAGAAACAGTAGAGAGTTGCGATAAAAAGCGTCAGGTAGTTT		P_{BAD}
**GFP-22NS**	GCACCCACCAACAACCTTAAGTTTGTATAGTTCATCCATGCCATGTGTAATCCCAGCAGCTGTTA	AflII	
**GFP-1 to GFP-21**	Derived from a previous study[a]		

Supplementary Table S1. Oligonucleotides used to assemble the synthetic *gfp*.
[a] Zampini, M. *et al.* RapGene: a fast and accurate strategy for synthetic gene assembly in *Escherichia coli*. *Sci Rep* **5**, 11302 (2015).
