# Supplementary material for: Terminator Operon Reporter: combining a transcription termination switch with reporter technology for improved gene synthesis and synthetic biology applications
Source: Sci Rep. 2016 May 25;6:26572. doi: 10.1038/srep26572 (PMC4879669; doi:10.1038/srep26572)
Supplement: Supplementary Data 1 [file srep26572-s2.pdf]

Supplementary Data 1

(Sequence alignment of the samples described in Table 1. The alignment spans the region between NheI and AflII and was performed with the online multiple sequence alignment software TCOFFE (http://tcoffee.crg.cat/apps/tcoffee/do:regular))

Terminator Operon Reporter: combining a transcription termination switch with reporter technology for improved gene synthesis and synthetic biology applications

Massimiliano Zampini[1*], Luis A J Mur[1], Pauline Rees Stevens[1], Justin A Pachebat[1], C James Newbold[1], Finbarr Hayes[2*] & Alison Kingston-Smith[1*]
[1] Institute of Biological, Environmental and Rural Sciences, Edward Llwyd Building, Aberystwyth University, Aberystwyth SY23 3FG, UK, [2] Faculty of Life Sciences, University of Manchester, Manchester M13 9PL, UK
*Correspondence should be addressed to M.Z. (M.Zampini@outlook.com) or F.H. (Finbarr.Hayes@manchester.ac.uk) or A. K-S. (ahk@aber.ac.uk)

1st PCA-*gfp* assembly (P_{recA*}) (Note: sample 15 is not reported as not readable)

```
                               NheI               PrecA*
1   AACCTATAATAAAAAAGAAAAAGGAGAGCTAGCAAACACTTGACACTGTATGAGCATACAGTATAATTGCTTCCGTAGGACG-GTGTAAAGGAGAGAAAGCATGAGTAAAGGAGAAGAACTTTTCACTGGAGTTGTCCCAATTCTTGTTGAATTAGATGGCGATGTTAATGGGCAAAAATTCTCTGTCAGTGGAGAGGGT 199
2   AACCTATAATAAAAAAGAAAAAGGAGAGCTAGCAAACACTTGACACTGTATGAGCATACAGTATAATTGCTTCCGTAGGACG-GTGTAAAGGAGAGAAAGCATGAGTAAAGGAGAAGAACTTTTCACTGGAGTTGTCCCAATTCTTGTTGAATTAGATGGCGATGTTAATGGGCAAAAATTCTCTGTCAGTGGAGAGGGT 199
3   AACCTATAATAAAAAAGAAAAAGGAGAGCTAGCAAACACTTGACACTGTATGAGCATACAGTATAATTGCTTCCGTAGGACG-GTGTAAAGGAGAGAAAGCATGAGTAAAGGAGAAGAACTTTTCACTGGAGTTGTCCCAATTCTTGTTGAATTAGATGGCGATGTTAATGGGCAAAAATTCTCTGTCAGTGGAGAGGGT 199
4   AACCTATAATAAAAAAGAAAAAGGAGAGCTAGCAAACACTTGACACTGTATGAGCATACAGTATAATTGCTTCCGTAGGACG-GTGTAAAGGAGAGAAAGCATGAGTAAAGGAGAAGAACTTTTCACTGGAGTTGTCCCAATTCTTGTTGAATTAGATGGCGATGTTAATGGGCAAAAATTCTCTGTCAGTGGAGAGGGT 199
5   AACCTATAATAAAAAAGAAAAAGGAGAGCTAGCAAACACTTGACACTGTATGAGCATACAGTATAATTGCTTCCGTAGGACG-GTGTAAAGGAGAGAAAGCATGAGTAAAGGAGAAGAACTTTTCACTGGAGTTGTCCCAATTCTTGTTGAATTAGATGGCGATGTTAATGGGCAAAAATTCTCTGTCAGTGGAGAGGGT 199
6   AACCTATAATAAAAAAGAAAAAGGAGAGCTAGCAAACACTTGACACTGTATGAGCATACAGTATAATTGCTTCCGTAGGACG-GTGTAAAGGAGAGAAAGCATGAGTAAAGGAGAAGAACTTTTCACTGGAGTTGTCCCAATTCTTGTTGAATTAGATGGCGATGTTAATGGGCAAAAATTCTCTGTCAGTGGAGAGGGT 199
7   AACCTATAATAAAAAAGAAAAAGGAGAGCTAGCAAACACTTGACACTGTATGAGCATACAGTATAATTGCTTCCGTAGGACG-GTGTAAAGGAGAGAAAGCATGAGTAAAGGAGAAGAACTTTTCACTGGAGTTGTCCCAATTCTTGTTGAATTAGATGGCGATGTTAATGGGCAAAAATTCTCTGTCAGTGGAGAGGGT 199
8   AACCTATAATAAAAAAGAAAAAGGAGAGCTAGCAAACACTTGACACTGTATGAGCATACAGTATAATTGCTTCCGTAGGACG-GTGTAAAGGAGAGAAAGCATGAGTAAAGGAGAAGAACTTTTCACTGGAGTTGTCCCAATTCTTGTTGAATTAGATGGCGATGTTAATGGGCAAAAATTCTCTGTCAGTGGAGAGGGT 199
9   AACCTATAATAAAAAAGAAAAAGGAGAGCTAGCAAACACTTGACACTGTATGAGCATACAGTATAATTGCTTCCGTAGGACG-GTGTAAAGGAGAGAAAGCATGAGTAAAGGAGAAGAACTTTTCACTGGAGTTGTCCCAATTCTTGTTGAATTAGATGGCGATGTTAATGGGCAAAAATTCTCTGTCAGTGGAGAGGGT 199
10  AACCTATAATAAAAAAGAAAAAGGAGAGCTAGCAAACACTTGACACTGTATGAGCATACAGTATAATTGCTTCCGTAGGACGGGTGTAAAGGAGAGAAAGCATGAGTAAAGGAGAAGAACTTTTCACTGGAGTTGTCCCAATTCTTGTTGAATTAGATGGCGATGTTAATGGGCAAAAATTCTCTGTCAGTGGAGAGGGT 200
11  AACCTATAATAAAAAAGAAAAAGGAGAGCTAGCAAACACTTGACACTGTATGAGCATACAGTATAATTGCTTCCGTAGGACG-GTGTAAAGGAGAGAAAGCATGAGTAAAGGAGAAGAACTTTTCACTGGAGTTGTCCCAATTCTTGTTGAATTAGATGGCGATGTTAATGGGCAAAAATACTCTGTCAGTGGAGAGGGT 199
12  AACCTATAATAAAAAAGAAAAAGGAGAGCTAGCAAACACTTGACACTGTATGAGCATACAGTATAATTGCTTCCGTAGGACG-GTGTAAAGGAGAGAAAGCATGAGTAAAGGAGAAGAACTTTTCACTGGAGTTGTCCCAATTCTTGTTGAATTAGATGGCGATGTTAATGGGCAAAAATTCTCTGTCAGTGGAGAGGGT 199
13  AACCTATAATAAAAAAGAAAAAGGAGAGCTAGCAAACACTTGACACTGTATGAGCATACAGTATAATTGCTTCCGTAGGACG-GTGTAAAGGAGAGAAAGCATGAGTAAAGGAGAAGAACTTTTCACTGGAGTTGTCCCAATTCTTGTTGAATTAGATGGCGATGTTAATGGGCAAAAATTCTCTGTCAGTGGAGAGGGT 199
14  AACCTATAATAAAAAAGAAAAAGGAGAGCTAGCAAACACTTGACACTGTATGAGCATACAGTATAATTGCTTCCGTAGGACG-GTGTAAAGGAGAGAAAGCATGAGTAAAGGAGAAGAACTTTTCACTGGAGTTGTCCCAATTCTTGTTGAATTAGATGGCGATGTTAATGGGCAAAAATTCTCTGTCAGTGGAGAGGGT 199
    ********************************************************************************** ************************************************************************************************* *******************

1   GAAGGTGATGCAACATACGGAAAACTTACCCTTAAATTTATTTGCACTACTGGGAAGCTACCTGTTCCATGGCCAACACTTGTCACTACTTTCTCTTATGGTGTTCAATGCTTTTCAAGATACCCAGATCATATGAAACAGCATGACTTTTTCAAGAGTGCCATGCCCGAAGGTTATGTACAGGAAAGAACTATATTTTA 399
2   GAAGGTGATGCAACATACGGAAAACTTACCCTTAAATTTATTTGCACTACTGGGAAGCTACCTGTTCCATGGCCAACACTTGTCACTACTTTCTCTTATGGTGTTCAATGCTTTTCAAGATACCCAGATCATATGAAACAGCATGACTTTTTCAAGAGTGCCATGCCCGAAGGTTATGTACAGGAAAGAACTATATTTTA 399
3   GAAGGTGATGCAACATACGGAAAACTTACCCTTAAATTTATTTGCACTACTGGGAAGCTACCTGTTCCATGGCCAACACTTGTCACTACTTTCTCTTATGGTGTTCAATGCTTTTCAAGATACCCAGATCATATGAAACAGCATGACTTTTTCAAGAGTGCCATGCCCGAAGGTTATGTACAGGAAAGAACTATATTTTA 399
4   GAAGGTGATGCAACATACGGAAAACTTACCCTTAAATTTATTTGCACTACTGGGAAGCTACCTGTTCCATGGCCAACACTTGTCACTACTTTCTCTTATGGTGTTCAATGCTTTTCAAGATACCCAGATCATATGAAACAGCATGACTTTTTCAAGAGTGCCATGCCCGAAGGTTATGTACAGGAAAGAACTATATTTTA 399
5   GAAGGTGATGCAACATACGGAAAACTTACCCTTAAATTTATTTGCACTACTGGGAAGCTACCTGTTCCATGGCCAACACTTGTCACTACTTTCTCTTATGGTGTTCAATGCTTTTCAAGATACCCAGATCATATGAAACAGCATGACTTTTTCAAGAGTGCCATGCCCGAAGGTTATGTACAGGAAAGAACTATATTTTA 399
6   GAAGGTGATGCAACATACGGAAAACTTACCCTTAAATTTATTTGCACTACTGGGAAGCTACCTGTTCCATGGCCAACACTTGTCACTACTTTCTCTTATGGTGTTCAATGCTTTTCAAGATACCCAGATCATATGAAACAGCATGACTTTTTCAAGAGTGCCATGCCCGAAGGTTATGTACAGGAAAGAACTATATTTTA 399
7   GAAGGTGATGCAACATACGGAAAACTTACCCTTAAATTTATTTGCACTACTGGGAAGCTACCTGTTCCATGGCCAACACTTGTCACTACTTTCTCTTATGGTGTTCAATGCTTTTCAAGATACCCAGATCATATGAAACAGCATGACTTTTTCAAGAGTGCCATGCCCGAAGGTTATGTACAGGAAAGAACTATATTTTA 399
8   GAAGGTGATGCAACATACGGAAAACTTACCCTTAAATTTATTTGCACTACTGGGAAGCTACCTGTTCCATGGCCAACACTTGTCACTACTTTCTCTTATGGTGTTCAATGCTTTTCAAGATACCCAGATCATATGAAACAGCATGACTTTTTCAAGAGTGCCATGCCCGAAGGTTATGTACAGGAAAGAACTATATTTTA 399
9   GAAGGTGATGCAACATACGGAAAACTTACCCTTAAATTTATTTGCACTACTGGGAAGCTACCTGTTCCATGGCCAACACTTGTCACTACTTTCTCTTATGGTGTTCAATGCTTTTCAAGATACCCAGATCATATGAAACAGCATGACTTTTTCAAGAGTGCCATGCCCGAAGGTTATGTACAGGAAAGAACTATATTTTA 399
10  GAAGGTGATGCAACATACGGAAAACTTACCCTTAAATTTATTTGCACTACTGGGAAGCTACCTGTTCCATGGCCAACACTTGTCACTACTTTCTCTTATGGTGTTCAATGCTTTTCAAGATACCCAGATCATATGAAACAGCATGACTTTTTCAAGAGTGCCATGCCCGAAGGTTATGTACAGGAAAGAACTATATTTTA 400
11  GAAGGTGATGCAACATACGGAAAACTTACCCTTAAATTTATTTGCACTACTGGGAAGCTACCTGTTCCATGGCCAACACTTGTCACTACTTTCTCTTATGGTGTTCAATGCTTTTCAAGATACCCAGATCATATGAAACAGCATGACTTTTTCAAGAGTGCCATGCCCGAAGGTTATGTACAGGAAAGAACTATATTTTA 399
12  GAAGGTGATGCAACATACGGAAAACTTACCCTTAAATTTATTTGCACTACTGGGAAGCTACCTGTTCCATGGCCAACACTTGTCACTACTTTCTCTTATGGTGTTCAATGCTTTTCAAGATACCCAGATCATATGAAACAGCATGACTTTTTCAAGAGTGCCATGCCCGAAGGTTATGTACAGGAAAGAACTATATTTTA 399
13  GAAGGTGATGCAACATACGGAAAACTTACCCTTAAATTTATTTGCACTACTGGGAAGCTACCTGTTCCATGGCCAACACTTGTCACTACTTTCTCTTATGGTGTTCAATGCTTTTCAAGATACCCAGATCATATGAAACAGCATGACT---------------TGCCCGAAGGTTATGTACAGGAAAGAACTATATTTTA 384
14  GAA----------------------------------------------------------------------------------------------------------------------------------------------------------------------------------------------------- 203
    ***

1   CAAAGATGACGGGAACTACAAGACACGTGCTGAAGTCAAGTTTGAAGGTGATACCCTTGTTAATAGAATCGAGTTAAAAGGTATTGATTTTAAAGAAGATGGAAACATTCTTGGACACAAAATGGAATACAACTATAACTCACATAATGTATACATCATGGCAGACAAACCAAAGAATGGAATCAAAGTTAACTTCAAAA 599
2   CAAAGATGACGGGAACTACAAGACACGTGCTGAAGTCAAGTTTGAAGGTGATACCCTTGTTAATAGAATCGAGTTAAAAGGTATTGATTTTAAAGAAGATGGAAACATTCTTGGACACAAAATGGAATACAACTATAACTCACATAATGTATACATCATGGCAGACAAACCAAAGAATGGAATCAAAGTTAACTTCAAAA 599
3   CAAAGATGACGGGAACTACAAGACACGTGCTGAAGTCAAGTTTGAAGGTGATACCCTTGTTAATAGAATCGAGTTAAAAGGTATTGATTTTAAAGAAGATGGAAACATTCTTGGACACAAAATGGAATACAACTATAACTCACATAATGTATACATCATGGCAGACAAACCAAAGAATGGAATCAAAGTTAACTTCAAAA 599
4   CAAAGATGACGGGAACTACAAGACACGTGCTGAAGTCAAGTTTGAAGGTGATACCCTTGTTAATAGAATCGAGTTAAAAGGTATTGATTTTAAAGAAGATGGAAACATTCTTGGACACAAAATGGAATACAACTATAACTCACATAATGTATACATCATGGCAGACAAACCAAAGAATGGAATCAAAGTTAACTTCAAAA 599
5   CAAAGATGACGGGAACTACAAGACACGTGCTGAAGTCAAGTTTGAAGGTGATACCCTTGTTAATAGAATCGAGTTAAAAGGTATTGATTTTAAAGAAGATGGAAACATTCTTGGACACAAAATGGAATACAACTATAACTCACATAATGTATACATCATGGCAGACAAACCAAAGAATGGAATCAAAGTTAACTTCAAAA 599
6   CAAAGATGACGGGAACTACAAGACACGTGCTGAAGTCAAGTTTGAAGGTGATACCCTTGTTAATAGAATCGAGTTAAAAGGTATTGATTTTAAAGAAGATGGAAACATTCTTGGACACAAAATGGAATACAACTATAACTCACATAATGTATACATCATGGCAGACAAACCAAAGAATGGAATCAAAGTTAACTTCAAAA 599
7   CAAAGATGACGGGAACTACAAGACACGTGCTGAAGTCAAGTTTGAAGGTGATACCCTTGTTAATAGAATCGAGTTAAAAGGTATTGATTTTAAAGAAGATGGAAACATTCTTGGACACAAAATGGAATACAACTATAACTCACATAATGTATACATCATGGCAGACAAACCAAAGAATGGAATCAAAGTTAACTTCAAAA 599
8   CAAAGATGACGGGAACTACAAGACACGTGCTGAAGTCAAGTTTGAAGGTGATACCCTTGTTAATAGAATCGAGTTAAAAGGTATTGATTTTAAAGAAGATGGAAACATTCTTGGACACAAAATGGAATACAACTATAACTCACATAATGTATACATCATGGCAGACAAACCAAAGAATGGAATCAAAGTTAACTTCAAAA 599
9   CAAAGATGACGGGAACTACAAGACACGTGCTGAAGTCAAGTTTGAAGGTGATACCCTTGTTAATAGAATCGAGTTAAAAGGTATTGATTTTAAAGAAGATGGAAACATTCTTGGACACAAAATGGAATACAACTATAACTCACATAATGTATACATCATGGCAGACAAACCAAAGAATGGAATCAAAGTTAACTTCAAAA 599
10  CAAAGATGACGGGAACTACAAGACACGTGCTGAAGTCAAGTTTGAAGGTGATACCCTTGTTAATAGAATCGAGTTAAAAGGTATTGATTTTAAAGAAGATGGAAACATTCTTGGACACAAAATGGAATACAACTATAACTCACATAATGTATACATCATGGCAGACAAACCAAAGAATGGAATCAAAGTTAACTTCAAAA 600
11  CAAAGATGACGGGAACTACAAGACACGTGCTGAAGTCAAGTTTGAAGGTGATACCCTTGTTAATAGAATCGAGTTAAAAGGTATTGATTTTAAAGAAGATGGAAACATTCTTGGACACAAAATGGAATACAACTATAACTCACATAATGTATACATCATGGCAGACAAACCAAAGAATGGAATCAAAGTTAACTTCAAAA 599
12  CAAAGATGACGGGAACTACAAGACACGTGCTGAAGTCAAGTTTGAAGGTGATACCCTTGTTAATAGAATCGAGTTAAAAGGTATTGATTTTAAAGAAGATGGAAACATTCTTGGACACAAAATGGAATACAACTATAACTCACATAATGTATACATCATGGCAGACAAACCAAAGAATGGAATCAAAGTTAACTTCAAAA 599
13  CAAAGATGACGGGAACTACAAGACACGTGCTGAAGTCAAGTTTGAAGGTGATACCCTTGTTAATAGAATCGAGTTAAAAGGTATTGATTTTAAAGAAGATGGAAACATTCTTGGACACAAAATGGAATACAACTATAACTCACATAATGTATACATCATGGCAGACAAACCAAAGAATGGAATCAAAGTTAACTTCAAAA 584
14  -------------------------------------------------------------------------------------------------------------------------------------------------------------------------------------------------------- 203

1   TTAGACACAACATTAAAGATGGAAGCGTTCAATTAGCAGACCATTATCAACAAAATACTCCAATTGGCGATGGCCCTGTCCTTTTACCAGACAACCATTACCTGTCCACACAATCTGCCCTTTCCAAAGATCCCAACGAAAAGAGAGATCACATGATCCTTCTTGAGTTTGTAACAGCTGCTGGGATTACACATGGCATG 799
2   TTAGACACAACATTAAAGATGGAAGCGTTCAATTAGCAGACCATTATCAACAAAATACTCCAATTGGCGATGGCCCTGTCCTTTTACCAGACAACCATTACCTGTCCACACAATCTGCCCTTTCCAAAGATCCCAACGAAAAGAGAGATCACATGATCCTTCTTGAGTTTGTAACAGCTGCTGGGATTACACATGGCATG 799
3   TTAGACACAACATTAAAGATGGAAGCGTTCAATTAGCAGACCATTATCAACAAAATACTCCAATTGGCGATGGCCCTGTCCTTTTACCAGACAACCATTACCTGTCCACACAATCTGCCCTTTCCAAAGATCCCAACGAAAAGAGAGATCACATGATCCTTCTTGAGTTTGTAACAGCTGCTGGGATTACACATGGCATG 799
4   TTAGACACAACATTAAAGATGGAAGCGTTCAATTAGCAGACCATTATCAACAAAATACTCCAATTGGCGATGGCCCTGTCCTTTTACCAGACAACCATTACCTGTCCACACAATCTGCCCTTTCCAAAGATCCCAACGAAAAGAGAGATCACATGATCCTTCTTGAGTTTGTAACAGCTGCTGGGATTACACATGGCATG 799
5   TTAGACACAACATTAAAGATGGAAGCGTTCAATTAGCAGACCATTATCAACAAAATACTCCAATTGGCGATGGCCCTGTCCTTTTACCAGACAACCATTACCTGTCCACACAATCTGCCCTTTCCAAAGATCCCAACGAAAAGAGAGATCACATGATCCTTCTTGAGTTTGTAACAGCTGCTGGGATTACACATGGCATG 799
6   TTAGACACAACATTAAAGATGGAAGCGTTCAATTAGCAGACCATTATCAACAAAATACTCCAATTGGCGATGGCCCTGTCCTTTTACCAGACAACCATTACCTGTCCACACAATCTGCCCTTTCCAAAGATCCCAACGAAAAGAGAGATCACATGATCCTTCTTGAGTTTGTAACAGCTGCTGGGATTACACATGGCATG 799
7   TTAGACACAACATTAAAGATGGAAGCGTTCAATTAGCAGACCATTATCAACAAAATACTCCAATTGGCGATGGCCCTGTCCTTTTACCAGACAACCATTACCTGTCCACACAATCTGCCCTTTCCAAAGATCCCAACGAAAAGAGAGATCACATGATCCTTCTTGAGTTTGTAACAGCTGCTGGGATTACACATGGCATG 799
8   TTAGACACAACATTAAAGATGGAAGCGTTCAATTAGCAGACCATTATCAACAAAATACTCCAATTGGCGATGGCCCTGTCCTTTTACCAGACAACCATTACCTGTCCACACAATCTGCCCTTTCCAAAGATCCCAACGAAAAGAGAGATCACATGATCCTTCTTGAGTTTGTAACAGCTGCTGGGATTACACATGGCATG 799
9   TTAGACACAACATTAAAGATGGAAGCGTTCAATTAGCAGACCATTATCAACAAAATACTCCAATTGGCGATGGCCCTGTCCTTTTACCAGACAACCATTACCTGTCCACACAATCTGCCCTTTCCAAAGATCCCAACGAAAAGAGAGATCACATGATCCTTCTTGAGTTTGTAACAGCTGCTGGGATTACACATGGCATG 799
10  TTAGACACAACATTAAAGATGGAAGCGTTCAATTAGCAGACCATTATCAACAAAATACTCCAATTGGCGATGGCCCTGTCCTTTTACCAGACAACCATTACCTGTCCACACAATCTGCCCTTTCCAAAGATCCCAACGAAAAGAGAGATCACATGATCCTTCTTGAGTTTGTAACAGCTGCTGGGATTACACATGGCATG 800
11  TTAGACACAACATTAAAGATGGAAGCGTTCAATTAGCAGACCATTATCAACAAAATACTCCAATTGGCGATGGCCCTGTCCTTTTACCAGACAACCATTACCTGTCCACACAATCTGCCCTTTCCAAAGATCCCAACGAAAAGAGAGATCACATGATCCTTCTTGAGTTTGTAACAGCTGCTGGGATTACACATGGCATG 799
12  TTAGACACAACATTAAAGATGGAAGCGTTCAATTAGCAGACCATTATCAACAAAATACTCCAATTGGCGATGGCCCTGTCCTTTTACCAGACAACCATTACCTGTCCACACAATCTGCCCTTTCCAAAGATCCCAACGAAAAGAGAGATCACATAATCCTTCTTGAGTTTGTAACAGCTGCTGGGATTACACATGGCATG 799
13  TTAGACACAACATTAAAGATGGAAGCGTTCAATTAGCAGACCATTATCAACAAAATACTCCAATTGGCGATGGCCCTGTCCTTTTACCAGACAACCATTACCTGTCCACACAATCTGCCCTTTCCAAAGATCCCAACGAAAAGAGAGATCACATGATCCTTCTTGAGTTTGTAACAGCTGCTGGGATTACACATGGCATG 784
14  --------------------------------------------------------------------------------------------------------------------------------------------------------------------GAGTTTGTAACAGCTGCTGGGATTACACATGGCATG 238
                                                                                                                                                                        ************************************

                   AflII
1   GATGAACTATACAAACTTAAGGTTGTTGGTGGGTGCACTAACCTAA 845
2   GATGAACTATACAAACTTAAGGTTGTTGGTGGGTGCACTAACCTAA 845
3   GATGAACTATACAAACTTAAGGTTGTTGGTGGGTGCACTAACCTAA 845
4   GATGAACTATACAAACTTAAGGTTGTTGGTGGGTGCACTAACCTAA 845
5   GATGAACTATACAAACTTAAGGTTGTTGGTGGGTGCACTAACCTAA 845
6   GATGAACTATACAAACTTAAGGTTGTTGGTGGGTGCACTAACCTAA 845
7   GATGAACTATACAAACTTAAGGTTGTTGGTGGGTGCACTAACCTAA 845
8   GATGAACTATACAAACTTAAGGTTGTTGGTGGGTGCACTAACCTAA 845
9   GATGAACTATACAAACTTAAGGTTGTTGGTGGGTGCACTAACCTAA 845
10  GATGAACTATACAAACTTAAGGTTGTTGGTGGGTGCACTAACCTAA 846
11  GATGAACTATACAAACTTAAGGTTGTTGGTGGGTGCACTAACCTAA 845
12  GATGAACTATACAAACTTAAGGTTGTTGGTGGGTGCACTAACCTAA 845
13  GATGAACTATACAAACTTAAGGTTGTTGGTGGGTGCACTAACCTAA 830
14  GATGAACTATACAAACTTAAGGTTGTTGGTGGGTGCACTAACCTAA 284
    **********************************************
```

1st RapGene-*gfp* assembly (P_{recA*})

```
                                      NheI                   PrecA*
16   AACCTATAATAAAAAAGAAAAAGGAGAGCTAGCAAACACTTGACACTGTATGAGCATACAGTATAATTGCTTCCGTAGGACGGTGTAAAG-GAGAGAAAGCATGAGTAAAGGAGAAGAACTTTTCACTGGAGTTGTCCCAATTCTTGTTGAATTAGATGGCGATGTTAATGGGCAAAAATTCTCTGTCAGTGGAGAGGGT 199
17   AACCTATAATAAAAAAGAAAAAGGAGAGCTAGCAAACACTTGACACTGTATGAGCATACAGTATAATTGCTTCCGTAGGACGGTGTAAAG-GAGAGAAAGCATGAGTAAAGGAGAAGAACTTTTCACTGGAGTTGTCCCAATTCTTGTTGAATTAGATGGCGATGTTAATGGGCAAAAATTCTCTGTCAGTGGAGAGGGT 199
18   AACCTATAATAAAAAAGAAAAAGGAGAGCTAGCAAACACTTGACACTGTATGAGCATACAGTATAATTGCTTCCGTAGGACGGTGTAAAG-GAGAGAAAGCATGAGTAAAGGAGAAGAACTTTTCACTGGAGTTGTCCCAATTCTTGTTGAATTAGATGGCGATGTTAATGGGCAAAAATTCTCTGTCAGTGGAGAGGGT 199
19   AACCTATAATAAAAAAGAAAAAGGAGAGCTAGCAAACACTTGACACTGTATGAGCATACAGTATAATTGCTTCCGTAGGACGGTGTAAAG-GAGAGAAAGCATGAGTAAAGGAGAAGAACTTTTCACTGGAGTTGTCCCAATTCTTGTTGAATTAGATGGCGATGTTAATGGGCAAAAATTCTCTGTCAGTGGAGAGGGT 199
20   AACCTATAATAAAAAAGAAAAAGGAGAGCTAGCAAACACTTGACACTGTATGAGCATACAGTATAATTGCTTCCGTAGGACGGTGTAAAG-GAGAGAAAGCATGAGTAAAGGAGAAGAACTTTTCACTGGAGTTGTCCCAATTCTTGTTGAATTAGATGGCGATGTTAATGGGCAAAAATTCTCTGTCAGTGGAGAGGGT 199
21   AACCTATAATAAAAAAGAAAAAGGAGAGCTAGCAAACACTTGACACTGTATGAGCATACAGTATAATTGCTTCCGTAGGACGGTGTAAAG-GAGAGAAAGCATGAGTAAAGGAGAAGAACTTTTCACTGGAGTTGTCCCAATTCTTGTTGAATTAGATGGCGATGTTAATGGGCAAAAATTCTCTGTCAGTGGAGAGGGT 199
22   AACCTATAATAAAAAAGAAAAAGGAGAGCTAGCAAACACTTGACACTGTATGAGCATACAGTATAATTGCTTCCGTAGGACGGTGTAAAG-GAGAGAAAGCATGAGTAAAGGAGAAGAACTTTTCACTGGAGTTGTCCCAATTCTTGTTGAATTAGATGGCGATGTTAATGGGCAAAAATTCTCTGTCAGTGGAGAGGGT 199
23   AACCTATAATAAAAAAGAAAAAGGAGAGCTAGCAAACACTTGACACTGTATGAGCATACAGTATAATTGCTTCCGTAGGACGGTGTAAAGGGAGAGAAAGCATGAGTAAAGGAGAAGAACTTTTCACTGGAGTTGTCCCAATTCTTGTTGAATTAGATGGCGATGTTAATGGGCAAAAATTCTCTGTCAGTGGAGAGGGT 200
24   AACCTATAATAAAAAAGAAAAAGGAGAGCTAGCAAACACTTGACACTGTATGAGCATACAGTATAATTGCTTCCGTAGGACGGTGTAAAG-GAGAGAAAGCATGAGTAAAGGAGAAGAACTTTTCACTGGAGTTGTCCCAATTCT------ATTAGATGGCGATGTTAATGGGCAAAAATTCTCTGTCAGTGGAGAGGGT 193
25   AACCTATAATAAAAAAGAAAAAGGAGAGCTAGCAAACACTTGACACTGTATGAGCATACAGTATAATTGCTTCCGTAGGACGGTGTAAAG-GAGAGAAAGCATGAGTAAAGGAGAAGAACTTTTCACTGGAGTTGTCCCAATTCTTGTTGAATTAGATGGCGATGTTAATGGGCAAAAATTCTCTGTCAGTGGAGAGGGT 199
26   AACCTATAATAAAAAAGAAAAAGGAGAGCTAGCAAACACTTGACACTGTATGAGCATACAGTATAATTGCTTCCGTAGGACGGTGTAAAG-GAGAGAAAGCATGAGTAAAGGAGAAGAACTTTTCACTGGAGTTGTCCCAATTCTTGTTGAATTAGATGGCGATGTTAATGGGCAAAAATTCTCTGTCAGTGGAGAGGGT 199
27   AACCTATAATAAAAAAGAAAAAGGAGAGCTAGCAAACACTTGACCCTGTATGAGCATACAGTATAATTGCTTCCGTAGGACGGTGTAAAG-GAGAGAAAGCATGAGTAAAGGAGAAGAACTTTT---------------------------------------------------------------------------- 123
28   AACCTATAATAAAAAAGAAAAAGGAGAGCTAGCAAACACTTGACACTGTATGAGCATACAGTATAATTGCTTCCGTAGGACGGTGTAAAG-GAGAGAAAGCATGAGTAAAGGAGAAGAACTTTTCACTGGAGTTGTCCCAATTCTTGTTGAATTAGATGGCGATGTTAATGGGCAAAAATTCTCTGTCAGTGGAGAGTG- 198
     ******************************************** ********************************************* *********************************

16   GAAGGTGATGCAACATACGGAAAACTTACCCTTAAATTTATTTGCACTACTGGGAAGCTACCTGTTCCATGGCCAACACTTGTCACTACTTTCTCTTATGGTGTTCAATGCTTTTCAAGATACCCAGATCATATGAAACAGCATGACTTTTTCAAGAGTGCCATGCCCGAAGGTTATGTACAGGAAAGAACTATATTTTA 399
17   GAAGGTGATGCAACATACGGAAAACTTACCCTTAAATTTATTTGCACTACTGGGAAGCTACCTGTTCCATGGCCAACACTTGTCACTACTTTCTCTTATGGTGTTCAATGCTTTTCAAGATACCCAGATCATATGAAACAGCATGACTTTTTCAAGAGTGCCATGCCCGAAGGTTATGTACAGGAAAGAACTATATTTTA 399
18   GAAGGTGATGCAACATACGGAAAACTTACCCTTAAATTTATTTGCACTACTGGGAAGCTACCTGTTCCATGGCCAACACTTGTCACTACTTTCTCTTATGGTGTTCAATGCTTTTCAAGATACCCAGATCATATGAAACAGCATGACTTTTTCAAGAGTGCCATGCCCGAAGGTTATGTACAGGAAAGAACTATATTTTA 399
19   GAAGGTGATGCAACATACGGAAAACTTACCCTTAAATTTATTTGCACTACTGGGAAGCTACCTGTTCCATGGCCAACACTTGTCACTACTTTCTCTTATGGTGTTCAATGCTTTTCAAGATACCCAGATCATATGAAACAGCATGACTTTTTCAAGAGTGCCATGCCCGAAGGTTATGTACAGGAAAGAACTATATTTTA 399
20   GAAGGTGATGCAACATACGGAAAACTTACCCTTAAATTTATTTGCACTACTGGGAAGCTACCTGTTCCATGGCCAACACTTGTCACTACTTTCTCTTATGGTGTTCAATGCTTTTCAAGATACCCAGATCATATGAAACAGCATGACTTTTTCAAGAGTGCCATGCCCGAAGGTTATGTACAGGAAAGAACTATATTTTA 399
21   GAAGGTGATGCAACATACGGAAAACTTACCCTTAAATTTATTTGCACTACTGGGAAGCTACCTGTTCCATGGCCAACACTTGTCACTACTTTCTCTTATGGTGTTCAATGCTTTTCAAGATACCCAGATCATATGAAACAGCATGACTTTTTCAAGAGTGCCATGCCCGAAGGTTATGTACAGGAAAGAACTATATTTTA 399
22   GAAGGTGATGCAACATACGGAAAACTTACCCTTAAATTTATTTGCACTACTGGGAAGCTACCTGTTCCATGGCCAACACTTGTCACTACTTTCTCTTATGGTGTTCAATGCTTTTCAAGATACCCAGATCATATGAAACAGCATGACTTTTTCAAGAGTGCCATGCCCGAAGGTTATGTACAGGAAAGAACTATATTTTA 399
23   GAAGGTGATGCAACATACGGAAAACTTACCCTTAAATTTATTTGCACTACTGGGAAGCTACCTGTTCCATGGCCAACACTTGTCACTACTTTCTCTTATGGTGTTCAATGCTTTTCAAGATACCCAGATCATATGAAACAGCATGACTTTTTCAAGAGTGCCATGCCCGAAGGTTATGTACAGGAAAGAACTATATTTTA 400
24   GAAGGTGATGCAACATACGGAAAACTTACCCTTAAATTTATTTGCACTACTGGGAAGCTACCTGTTCCATGGCCAACACTTGTCACTACTTTCTCTTATGGTGTTCAATGCTTTTCAAGATACCCAGATCATATGAAACAGCATGACTTTTTCAAGAGTGCCATG---GAAGGTTATGTACAGGAAAGAACTATATTTTA 390
25   GAAGGTGATGCAACATACGGAAAACTTACCCTTAAATTTATTTGCACTACTGGGAAGCTACCTGTTCCATGGCCAACACTTGTCACTACTTTCTCTTATGGTGTTCAATGCTTTTCAAGATACCCAGATCATATGAAACAGCATGACTTTTTCAAGAGTGCCATGCCCGAAGGTTATGTACAGGAAAGAACTATATTTTA 399
26   GAAGGTGATGCAACATACGGAAAACTTACCCTTAAATTTATTTGCACTACTGGGAAGCTACCTGTTCCATGGCCAACACTTGTCACTACTTTCTCTTATGGTGTTCAATGCTTTTCAAGATACCCAGATCATATGAAACAGCATGACTTTTTCAAGAGTGCCATGCCCGAAGGTTATGTACAGGAAAGAACTATATTTTA 399
27   -------------------------------------------------------------------------------------------------------------------------------------------------------------------------------------------------------- 123
28   -------------------------------------------------------------------------------------------------------------------------------------------------------------------------------------------------------- 198

16   CAAAGATGACGGGAACTACAAGACACGTGCTGAAGTCAAGTTTGAAGGTGATACCCTTGTTAATAGAATCGAGTTAAAAGGTATTGATTTTAAAGAAGATGGAAACATTCTTGGACACAAAATGGAATACAACTATAACTCACATAATGTATACATCATGGCAGACAAACCAAAGAATGGAATCAAAGTTAACTTCAAAA 599
17   CAAAGATGACGGGAACTACAAGACACGTGCTGAAGTCAAGTTTGAAGGTGATACCCTTGTTAATAGAATCGAGTTAAAAGGTATTGATTTTAAAGAAGATGGAAACATTCTTGGACACAAAATGGAATACAACTATAACTCACATAATGTATACATCATGGCAGACAAACCAAAGAATGGAATCAAAGTTAACTTCAAAA 599
18   CAAAGATGACGGGAACTACAAGACACGTGCTGAAGTCAAGTTTGAAGGTGATACCCTTGTTAATAGAATCGAGTTAAAAGGTATTGATTTTAAAGAAGATGGAAACATTCTTGGACACAAAATGGAATACAACTATAACTCACATAATGTATACATCATGGCAGACAAACCAAAGAATGGAATCAAAGTTAACTTCAAAA 599
19   CAAAGATGACGGGAACTACAAGACACGTGCTGAAGTCAAGTTTGAAGGTGATACCCTTGTTAATAGAATCGAGTTAAAAGGTATTGATTTTAAAGAAGATGGAAACATTCTTGGACACAAAATGGAATACAACTATAACTCACATAATGTATACATCATGGCAGACAAACCAAAGAATGGAATCAAAGTTAACTTCAAAA 599
20   CAAAGATGACGGGAACTACAAGACACGTGCTGAAGTCAAGTTTGAAGGTGATACCCTTGTTAATAGAATCGAGTTAAAAGGTATTGATTTTAAAGAAGATGGAAACATTCTTGGACACAAAATGGAATACAACTATAACTCACATAATGTATACATCATGGCAGACAAACCAAAGAATGGAATCAAAGTTAACTTCAAAA 599
21   CAAAGATGACGGGAACTACAAGACACGTGCTGAAGTCAAGTTTGAAGGTGATACCCTTGTTAATAGAATCGAGTTAAAAGGTATTGATTTTAAAGAAGATGGAAACATTCTTGGACACAAAATGGAATACAACTATAACTCACATAATGTATACATCATGGCAGACAAACCAAAGAATGGAATCAAAGTTAACTTCAAAA 599
22   CAAAGATGACGGGAACTACAAGACACGTGCTGAAGTCAAGTTTGAAGGTGATACCCTTGTTAATAGAATCGAGTTAAAAGGTATTGATTTTAAAGAAGATGGAAACATTCTTGGACACAAAATGGAATACAACTATAACTCACATAATGTATACATCATGGCAGACAAACCAAAGAATGGAATCAAAGTTAACTTCAAAA 599
23   CAAAGATGACGGGAACTACAAGACACGTGCTGAAGTCAAGTTTGAAGGTGATACCCTTGTTAATAGAATCGAGTTAAAAGGTATTGATTTTAAAGAAGATGGAAACATTCTTGGACACAAAATGGAATACAACTATAACTCACATAATGTATACATCATGGCAGACAAACCAAAGAATGGAATCAAAGTTAACTTCAAAA 600
24   CAAAGATGACGGGAACTACAAGACACGTGCTGAAGTCAAGTTTGAAGGTGATACCCTTGTTAATAGAATCGAGTTAAAAGGTATTGATTTTAAAGAAGATGGAAACATTCTTGGACACAAAATGGAATACAACTATAACTCACATAATGTATACATCATGGCAGACAAACCAAAGAATGGAATCAAAGTTAACTTCAAAA 590
25   CAAAGATGACGGGAACTACAAGACACGTGCTGAAGTCAAGTTTGAAGGTGATACCCTTGTTAATAGAATCGAGTTAAAAGGTATTGATTTTAAAGAAGATGGAAACATTCTTGGACACAAAATGGAATACAACTATAACTCACATAATGTATACATCATGGCAGACAAACCAAAGAATGGAATCAAAGTTAACTTCAAAA 599
26   CAAAGATGACGGGAACTACAAGACACGTGCTGAAGTCAAGTTTGAAGGTGATACCCTTGTTAATAGAATCGAGTTAAAAGGTATTGATTTTAAAGAAGATGGAAACATTCTTGGACACAAAATGGAATACAACTATAACTCACATAATGTATACATCATGGCAGACAAACCAAAGAATGGAATCAAAGTTAACTTCAAAA 599
27   -------------------------------------------------------------------------------------------------------------------------------------------------------------------------------------------------------- 123
28   ------------------------------------------TGAAGGTGATACCCTTGTTAATAGAATCGAGTTAAAAGGTATTGATTTTAAAGAAGATGGAAACATTCTTGGACACAAAATGGAATACAACTATAACTCACATAATGTATACATCATGGCAGACAAACCAAAGAATGGAATCAAAGTTAACTTCAAAA 356

16   TTAGACACAACATTAAAGATGGAAGCGTTCAATTAGCAGACCATTATCAACAAAATACTCCAATTGGCGATGGCCCTGTCCTTTTACCAGACAACCATTACCTGTCCACACAATCTGCCCTTTCCAAAGATCCCAACGAAAAGAGAGATCACATGATCCTTCTTGAGTTTGTAACAGCTGCTGGGATTACACATGGCATG 799
17   TTAGACACAACATTAAAGATGGAAGCGTTCAATTAGCAGACCATTATCAACAAAATACTCCAATTGGCGATGGCCCTGTCCTTTTACCAGACAACCATTACCTGTCCACACAATCTGCCCTTTCCAAAGATCCCAACGAAAAGAGAGATCACATGATCCTTCTTGAGTTTGTAACAGCTGCTGGGATTACACATGGCATG 799
18   TTAGACACAACATTAAAGATGGAAGCGTTCAATTAGCAGACCATTATCAACAAAATACTCCAATTGGCGATGGCCCTGTCCTTTTACCAGACAACCATTACCTGTCCACACAATCTGCCCTTTCCAAAGATCCCAACGAAAAGAGAGATCACATGATCCTTCTTGAGTTTGTAACAGCTGCTGGGATTACACATGGCATG 799
19   TTAGACACAACATTAAAGATGGAAGCGTTCAATTAGCAGACCATTATCAACAAAATACTCCAATTGGCGATGGCCCTGTCCTTTTACCAGACAACCATTACCTGTCCACACAATCTGCCCTTTCCAAAGATCCCAACGAAAAGAGAGATCACATGATCCTTCTTGAGTTTGTAACAGCTGCTGGGATTACACATGGCATG 799
20   TTAGACACAACATTAAAGATGGAAGCGTTCAATTAGCAGACCATTATCAACAAAATACTCCAATTGGCGATGGCCCTGTCCTTTTACCAGACAACCATTACCTGTCCACACAATCTGCCCTTTCCAAAGATCCCAACGAAAAGAGAGATCACATGATCCTTCTTGAGTTTGTAACAGCTGCTGGGATTACACATGGCATG 799
21   TTAGACACAACATTAAAGATGGAAGCGTTCAATTAGCAGACCATTATCAACAAAATACTCCAATTGGCGATGGCCCTGTCCTTTTACCAGACAACCATTACCTGTCCACACAATCTGCCCTTTCCAAAGATCCCAACGAAAAGAGAGATCACATGATCCTTCTTGAGTTTGTAACAGCTGCTGGGATTACACATGGCATG 799
22   TTAGACACAACATTAAAGATGGAAGCGTTCAATTAGCAGACCATTATCAACAAAATACTCCAATTGGCGATGGCCCTGTCCTTTTACCAGACAACCATTACCTGTCCACACAATCTGCCCTTTCCAAAGATCCCAACGAAAAGAGAGATCACATGATCCTTCTTGAGTTTGTAACAGCTGCTGGGATTACACATGGCATG 799
23   TTAGACACAACATTAAAGATGGAAGCGTTCAATTAGCAGACCATTATCAACAAAATACTCCAATTGGCGATGGCCCTGTCCTTTTACCAGACAACCATTACCTGTCCACACAATCTGCCCTTTCCAAAGATCCCAACGAAAAGAGAGATCACATGATCCTTCTTGAGTTTGTAACAGCTGCTGGGATTACACATGGCATG 800
24   TTAGACACAACATTAAAGATGGAAGCGTTCAATTAGCAGACCATTATCAACAAAATACTCCAATTGGCGATGGCCCTGTCCTTTTACCAGACAACCATTACCTGTCCACACAATCTGCCCTTTCCAAAGATCCCAACGAAAAGAGAGATCACATGATCCTTCTTGAGTTTGTAACAGCTGCTGGGATTACACATGGCATG 790
25   TTAGACACAACATTAAAGATGGAATCGTTCAATTAGCAGACCATTATCAACAAAATACTCCAATTGGCGATGGCCCTGTCCTTTTACCAGACAACCATTACCTGTCCACACAATCTGCCCTTTCCAAAGATCCCAACGAAAAGAGAGATCACATGATCCTTCTTGAGTTTGTAACAGCTGCTGGGATTACACATGGCATG 799
26   TTAGACACAACATTAAAGATGGAAGCGTTCAATTAGCAGACCATTATCAACAAAATACTCCAATTGGCGATGGCCCTGTCCTTTTACCAGACAACCATTACCTGCCCACACAATCTGCCCTTTCCAAAGATCCCAACGAAAAGAGAGATCACATGATCCTTCTTGAGTTTGTAACAGCTGCTGGGATTACACATGGCATG 799
27   ----------------------------------------------------------------------------------------------------------------------------CAAAGATCCCAACGAAAAGAGAGATCACATGATCCTTCTTGAGTTTGTAACAGCTGCTGGGATTACACATGGCATG 199
28   TTAGACACAACATTAAAGATGGAAGCGTTCAATTAGCAGACCATTATCAACAAAATACTCCAATTGGCGATGGCCCTGTCCTTTTACCAGACAACCATTACCTGTCCACACAATCTGCCCTTTCCAAAGATCCCAACGAAAAGAGAGATCACATGATCCTTCTTGAGTTTGTAACAGCTGCTGGGATTACACATGGCATG 556
                                                                                                                                 ****************************************************************************

                    AflII
16   GATGAACTATACAAACTTAAGGTTGTTGGTGGGTGCACTAACCTAA 845
17   GATGAACTATACAAACTTAAGGTTGTTGGTGGGTGCACTAACCTAA 845
18   GATGAACTATACAAACTTAAGGTTGTTGGTGGGTGCACTAACCTAA 845
19   GATGAACTATACAAACTTAAGGTTGTTGGTGGGTGCACTAACCTAA 845
20   GATGAACTATACAAACTTAAGGTTGTTGGTGGGTGCACTAACCTAA 845
21   GATGAACTATACAAACTTAAGGTTGTTGGTGGGTGCACTAACCTAA 845
22   GATGAACTATACAAACTTAAGGTTGTTGGTGGGTGCACTAACCTAA 845
23   GATGAACTATACAAACTTAAGGTTGTTGGTGGGTGCACTAACCTAA 846
24   GATGAACTATACAAACTTAAGGTTGTTGGTGGGTGCACTAACCTAA 836
25   GATGAACTATACAAACTTAAGGTTGTTGGTGGGTGCACTAACCTAA 845
26   GATGAACTATACAAACTTAAGGTTGTTGGTGGGTGCACTAACCTAA 845
27   GATGAACTATACAAACTTAAGGTTGTTGGTGGGTGCACTAACCTAA 245
28   GATGAACTATACAAACTTAAGGTTGTTGGTGGGTGCACTAACCTAA 602
     **********************************************
```

2nd PCA-*gfp* assembly (P_{recA*})

```
                                NheI                PrecA*
29   AACCTATAATAAAAAAGAAAAAGGAGAGCTAGCAAACACTTGACACTGTATGAGCATACAGTATAATTGCTTCCGTAGGACGGTG-TAAAGGAGAGAAAGCATGAGTAAAGGAGAAGAACTTTTCACTGGAGTTGTCCCAATTCTTGTTGAATTAGATGGCGATGTTAATGGGCAAAAATTCTCTGTCAGTGGAGAGGGT 199
30   AACCTATAATAAAAAAGAAAAAGGAGAGCTAGCAAACACTTGACACTGTATGAGCATACAGTATAATTGCTTCCGTAGGACGGTG-TAAAGGAGAGAAAGCATGAGTAAAGGAGAAGAACTTTTCACTGGAGTTGTCCCAATTCTTGTTGAATTAGATGGCGATGTTAATGGGCAAAAATTCTCTGTCAGTGGAGAGGGT 199
31   AACCTATAATAAAAAAGAAAAAGGAGAGCTAGCAAACACTTGACACTGTATGAGCATACAGTATAATTGCTTCCGTAGGACGGTG-TAAAGGAGAGAAAGCATGAGTAAAGGAGAAGAACTTTTCACTGGAGTTGTCCCAATTCTTGTTGAATTAGATGGCGATGTTAATGGGCAAAAATTCTCTGTCAGTGGAGAGGGT 199
32   AACCTATAATAAAAAAGAAAAAGGAGAGCTAGCAAACACTTGACACTGTATGAGCATACAGTATAATTGCTTCCGTAGGACGGTG-TAAAGGAGAGAAAGCATGAGTAAAGGAGAAGAACTTTTCACTGGAGTTGTCCCAATTCTTGTTGAATTAGATGGCGATGTTAATGGGCAAAAATTCTCTGTCAGTGGAGAGGGT 199
33   AACCTATAATAAAAAAGAAAAAGGAGAGCTAGCAAACACTTGACACTGTATGAGCATACAGTATAATTGCTTCCGTAGGACGGTG-TAAAGGAGAGAAAGCATGAGTAAAGGAGAAGAACTTTTCACTGGAGTTGTCCCAATTCTTGTTGAATTAGATGGCGATGTTAATGGGCAAAAATTCTCTGTCAGTGGAGAGGGT 199
34   AACCTATAATAAAAAAGAAAAAGGAGAGCTAGCAAACACTTGACACTGTATGAGCATACAGTATAATTGCTTCCGTAGGACGGTG-TAAAGGAGAGAAAGCATGAGTAAAGGAGAAGAACTTTTCACTGGAGTTGTCCCAATTCTTGTTGAATTAGATGGCGATGTTAATGGGCAAAAATTCTCTGTCAGTGGAGAGGGT 199
35   AACCTATAATAAAAAAGAAAAAGGAGAGCTAGCAAACACTTGACACTGTATGAGCATACAGTATAATTGCTTCCGTAGGACGGTG-TAAAGGAGAGAAAGCATGAGTAAAGGAGAAGAACTTTTCACTGGAGTTGTCCCAATTCTTGTTGAATTAGATGGCGATGTTAATGGGCAAAAATTCTCTGTCAGTGGAGAGGGT 199
36   AACCTATAATAAAAAAGAAAAAGGAGAGCTAGCAAACACTTGACACTGTATGAGCATACAGTATAATTGCTTCCGTAGGACGGTG-TAAAGGAGAGAAAGCATGAGTAAAGGAGAAGAACTTTTCACTGGAGTTGTCCCAATTCTTGTTGAATTAGATGGCGATGTTAATGGGCAAAAATTCTCTGTCAGTGGAGAGGGT 199
37   AACCTATAATAAAAAAGAAAAAGGAGAGCTAGCAAACACTTGACACTGTATGAGCATACAGTATAATTGCTTCCGTAGGACGGTG-TAAAGGAGAGAAAGCATGAGTAAAGGAGAAGAACTTTTCACTGGAGTTGTCCCAATTCTTGTTGAATTAGATGGCGATGTTAATGGGCAAAAATTCTCTGTCAGTGGAGAGGGT 199
38   AACCTATAATAAAAAAGAAAAAGGAGAGCTAGGAAACACTTGACACTGTATGAGCATACAGTATAATTGCTTCCGTAGGACGGTG-TAAAGGAGAGAAAG-ATGAGTAAAGGAGAAGAACTTTTCACTGGAGTTGTCCCAATTCTTGTTGAATTAGATGGCGATGTTAATGGGCAAAAATTCTCTGTCAGTGGAGAGGGT 198
39   AACCTATAATAAAAAAGAAAAAGGAGAGCTAGCAAACACTTGACACTGTATGAGCATACAGTATAATTGCTTCCGTAGGACGGTGGTAAAGGAGAGAAAGCATGAGTAAAGGAGAAGAACTTTTCACTGGAGTTGTCCCAATTCTTGTTGAATTAGATGGCGATGTTAATGGGCAAAAATTCTCTGTCAGTGGAGAGGGT 200
40   AACCTATAATAAAAAAGAAAAAGGAGAGCTAGCAAACACTTGACACTGTATGAGCATACAGTATAATTGCTTCCGTAGGACGGTG-TAAAGGAGAGAAAGCATGAGTAAAGGAGAAGAACTTTTCACTGGAGTTGTCCCAATTCTTGTTGAATTAGATGGCGATGTTAATGGGCAAAAATTCTCTGTCAGTGGAGAGGGT 199
41   AACCTATAATAAAAAAGAAAAAGGAGAGCT-GCAAACACTTGACACTGTATGAGCATACAGTATAATTGCTTCCGTAGGACGGTG-TAAAGGAGAGAAAGCATGAGTAAAGGAGAAGAACTTTTCACTGGAGTTGTCCCAATTCTTGTTGAATTAGATGGCGATGTTAATGGGCAAAAATTCTCTGTCAGTGGAGAGGGT 198
42   AACCTATAATAAAAAAGAAAAAGGAGAGCTAGCAAACACTTGACACTGTATGAGCATACAGTATAATTGCTT-CGTAGGACGGTG-TAAAGGAGAGAAAGCATGAGTAAAGGAGAAGAACTTTTCACTGGAGTTGTCCCAATTCTTGTTGAATTAGATGGCGATGTTAATGGGCAAAAATTCTCTGTCAGTGGAGAGGGT 198
43   AACCTATAATAAAAAAGAAAAAGGAGAGCTAGCAAACACTTGACACTGTATGAGCATACAGTATAATTGCTTCCGTAGGACGGTG-TAAAGGAGAGAAAGCATGAGTAAAGGAGAAGAACTTTTCACTGGAGTTGTCCCAATTCTTGTTGAATTAGATGGCGATGTTAATGGGCAAAAATTCTCTGTCAGTGGAGAGGGT 199
     ****************************** * *************************************** *********** ************** ****************************************************************************************************

29   GAAGGTGATGCAACATACGGAAAACTTACCCTTAAATTTATTTGCACTACTGGGAAGCTACCTGTTCCATGGCCAACACTTGTCACTACTTTCTCTTATGGTGTTCAATGCTTTTCAAGATACCCAGATCATATGAAACAGCATGACTTTTTCAAGAGTGCCATGCCCGAAGGTTATGTACAGGAAAGAACTATATTTTTA 399
30   GAAGGTGATGCAACATACGGAAAACTTACCCTTAAATTTATTTGCACTACTGGGAAGCTACCTGTTCCATGGCCAACACTTGTCACTACTTTCTCTTATGGTGTTCAATGCTTTTCAAGATACCCAGATCATATGAAACAGCATGACTTTTTCAAGAGTGCCATGCCCGAAGGTTATGTACAGGAAAGAACTATATTTTTA 399
31   GAAGGTGATGCAACATACGGAAAACTTACCCTTAAATTTATTTGCACTACTGGGAAGCTACCTGTTCCATGGCCAACACTTGTCACTACTTTCTCTTATGGTGTTCAATGCTTTTCAAGATACCCAGATCATATGAAACAGCATGACTTTTTCAAGAGTGCCATGCCCGAAGGTTATGTACAGGAAAGAACTATATTTTTA 399
32   GAAGGTGATGCAACATACGGAAAACTTACCCTTAAATTTATTTGCACTACTGGGAAGCTACCTGTTCCATGGCCAACACTTGTCACTACTTTCTCTTATGGTGTTCAATGCTTTTCAAGATACCCAGATCATATGAAACAGCATGACTTTTTCAAGAGTGCCATGCCCGAAGGTTATGTACAGGAAAGAACTATATTTTTA 399
33   GAAGGTGATGCAACATACGGAAAACTTACCCTTAAATTTATTTGCACTACTGGGAAGCTACCTGTTCCATGGCCAACACTTGTCACTACTTTCTCTTATGGTGTTCAATGCTTTTCAAGATACCCAGATCATATGAAACAGCATGACTTTTTCAAGAGTGCCATGCCCGAAGGTTATGTACAGGAAAGAACTATATTTTTA 399
34   GAAGGTGATGCAACATACGGAAAACTTACCCTTAAATTTATTTGCACTACTGGGAAGCTACCTGTTCCATGGCCAACACTTGTCACTACTTTCTCTTATGGTGTTCAATGCTTTTCAAGATACCCAGATCATATGAAACAGCATGACTTTTTCAAGAGTGCCATGCCCGAAGGTTATGTACAGGAAAGAACTATATTTTTA 399
35   GAAGGTGATGCAACATACGGAAAACTTACCCTTAAATTTATTTGCACTACTGGGAAGCTACCTGTTCCATGGCCAACACTTGTCACTACTTTCTCTTATGGTGTTCAATGCTTTTCAAGATACCCAGATCATATGAAACAGCATGACTTTTTCAAGAGTGCCATGCCCGAAGGTTATGTACAGGAAAGAACTATATTTTTA 399
36   GAAGGTGATGCAACATACGGAAAACTTACCCTTAAATTTATTTGCACTACTGGGAAGCTACCTGTTCCATGGCCAACACTTGTCACTACTTTCTCTTATGGTGTTCAATGCTTTTCAAGATACCCAGATCATATGAAACAGCATGACTTTTTCAAGAGTGCCATGCCCGAAGGTTATGTACAGGAAAGAACTATATTTTTA 399
37   GAAGGTGATGCAACATACGGAAAACTTACCCTTAAATTTATTTGCACTACTGGGAAGCTACCTGTTCCATGGCCAACACTTGTCACTACTTTCTCTTATGGTGTTCAATGCTTTTCAAGATACCCAGATCATATGAAACAGCATGACTTTTTCAAGAGTGCCATGCCCGAAGGTTATGTACAGGAAAGAACTATATTTTTA 399
38   GAAGGTGATGCAACATACGGAAAACTTACCCTTAAATTTATTTGCACTACTGGGAAGCTACCTGTTCCATGGCCAACACTTGTCACTACTTTCTCTTATGGTGTTCAATGCTTTTCAAGATACCCAGATCATATGAAACAGCATGACTTTTTCAAGAGTGCCATGCCCGAAGGTTATGTACAGGAAAGAACTATATTTTTA 398
39   GAAGGTGATGCAACATACGGAAAACTTACCCTTAAATTTATTTGCACTACTGGGAAGCTACCTGTTCCATGGCCAACACTTGTCACTACTTTCTCTTATGGTGTTCAATGCTTTTCAAGATACCCAGATCATATGAAACAGCATGACTTTTTCAAGAGTGCCATGCCCGAAGGTTATGTACAGGAAAGAACTATATTTTTA 400
40   GAAGGTGATGCAACATACGGAAAACTTACCCTTAAATTTATTTGCACTACTGGGAAGCTACCTGTTCCATGGCCAACACTTGTCACTACTTTCTCTTATGGTGTTCAATGCTTTTCAAGATACCCAGATCATATGAAACAGCATGACTTTTTCAAGAGTGCCATGCCCGAAGGTTATGTACAGGAAAGAACTATATTTTTA 399
41   GAAGGTGATGCAACATACGGAAAACTTACCCTTAAATTTATTTGCACTACTGGGAAGCTACCTGTTCCATGGCCAACACTTGTCACTACTTTCTCTTATGGTGTTCAATGCTTTTCAAGATACCCAGATCATATGAAACAGCATGACTTTTTCAAGAGTGCCATGCCCGAAGGTTATGTACAGGAAAGAACTATATTTTTA 398
42   GAAGGTGATGCAACATACGGAAAACTTACCCTTAAATTTATTTGCACTACTGGGAAGCTACCTGTTCCATGGCCAACACTTGTCACTACTTTCTCTTATGGTGTTCAATGCTTTTCAAGATACCCAGATCATATGAAACAGCATGACTTTTTCAAGAGTGCCATGCCCGAAGGTTATGTACAGGAAAGAACTATATTTTTA 398
43   -------------------------------------------------------------------------------------------------------------------------------------------------------------------------------------------------------- 205

29   CAAAGATGACGGGAACTACAAGACACGTGCTGAAGTCAAGTTTGAAGGTGATACCCTTGTTAATAGAATCGAGTTAAAAGGTATTGATTTTAAAGAAGATGGAAACATTCTTGGACACAAAATGGAATACAACTATAACTCACATAATGTATACATCATGGCAGACAAACCAAAGAATGGAATCAAAGTTAACTTCAAAA 599
30   CAAAGATGACGGGAACTACAAGACACGTGCTGAAGTCAAGTTTGAAGGTGATACCCTTGTTAATAGAATCGAGTTAAAAGGTATTGATTTTAAAGAAGATGGAAACATTCTTGGACACAAAATGGAATACAACTATAACTCACATAATGTATACATCATGGCAGACAAACCAAAGAATGGAATCAAAGTTAACTTCAAAA 599
31   CAAAGATGACGGGAACTACAAGACACGTGCTGAAGTCAAGTTTGAAGGTGATACCCTTGTTAATAGAATCGAGTTAAAAGGTATTGATTTTAAAGAAGATGGAAACATTCTTGGACACAAAATGGAATACAACTATAACTCACATAATGTATACATCATGGCAGACAAACCAAAGAATGGAATCAAAGTTAACTTCAAAA 599
32   CAAAGATGACGGGAACTACAAGACACGTGCTGAAGTCAAGTTTGAAGGTGATACCCTTGTTAATAGAATCGAGTTAAAAGGTATTGATTTTAAAGAAGATGGAAACATTCTTGGACACAAAATGGAATACAACTATAACTCACATAATGTATACATCATGGCAGACAAACCAAAGAATGGAATCAAAGTTAACTTCAAAA 599
33   CAAAGATGACGGGAACTACAAGACACGTGCTGAAGTCAAGTTTGAAGGTGATACCCTTGTTAATAGAATCGAGTTAAAAGGTATTGATTTTAAAGAAGATGGAAACATTCTTGGACACAAAATGGAATACAACTATAACTCACATAATGTATACATCATGGCAGACAAACCAAAGAATGGAATCAAAGTTAACTTCAAAA 599
34   CAAAGATGACGGGAACTACAAGACACGTGCTGAAGTCAAGTTTGAAGGTGATACCCTTGTTAATAGAATCGAGTTAAAAGGTATTGATTTTAAAGAAGATGGAAACATTCTTGGACACAAAATGGAATACAACTATAACTCACATAATGTATACATCATGGCAGACAAACCAAAGAATGGAATCAAAGTTAACTTCAAAA 599
35   CAAAGATGACGGGAACTACAAGACACGTGCTGAAGTCAAGTTTGAAGGTGATACCCTTGTTAATAGAATCGAGTTAAAAGGTATTGATTTTAAAGAAGATGGAAACATTCTTGGACACAAAATGGAATACAACTATAACTCACATAATGTATACATCATGGCAGACAAACCAAAGAATGGAATCAAAGTTAACTTCAAAA 599
36   CAAAGATGACGGGAACTACAAGACACGTGCTGAAGTCAAGTTTGAAGGTGATACCCTTGTTAATAGAATCGAGTTAAAAGGTATTGATTTTAAAGAAGATGGAAACATTCTTGGACACAAAATGGAATACAACTATAACTCACATAATGTATACATCATGGCAGACAAACCAAAGAATGGAATCAAAGTTAACTTCAAAA 599
37   CAAAGATGACGGGAACTACAAGACACGTGCTGAAGTCAAGTTTGAAGGTGATACCCTTGTTAATAGAATCGAGTTAAAAGGTATTGATTTTAAAGAAGATGGAAACATTCTTGGACACAAAATGGAATACAACTATAACTCACATAATGTATACATCATGGCAGACAAACCAAAGAATGGAATCAAAGTTAACTTCAAAA 599
38   CAAAGATGACGGGAACTACAAGACACGTGCTGAAGTCAAGTTTGAAGGTGATACCCTTGTTAATAGAATCGAGTTAAAAGGTATTGATTTTAAAGAAGATGGAAACATTCTTGGACACAAAATGGAATACAACTATAACTCACATAATGTATACATCATGGCAGACAAACCAAAGAATGGAATCAAAGTTAACTTCAAAA 598
39   CAAAGATGACGGGAACTACAAGACACGTGCTGAAGTCAAGTTTGAAGGTGATACCCTTGTTAATAGAATCGAGTTAAAAGGTATTGATTTTAAAGAAGATGGAAACATTCTTGGACACAAAATGGAATACAACTATAACTCACATAATGTATACATCATGGCAGACAAACCAAAGAATGGAATCAAAGTTAACTTCAAAA 600
40   CAAAGATGACGGGAACTACAAGACACGTGCTGAAGTCAAGTTTGAAGGTGATACCCTTGTTAATAGAATCGAGTTAAAAGGTATTGATTTTAAAGAAGATGGAAACATTCTTGGACACAAAATGGAATACAACTATAACTCACATAATGTATACATCATGGCAGACAAACCAAAGAATGGAATCAAAGTTAACTTCAAAA 599
41   CAAAGATGACGGGAACTACAAGACACGTGCTGAAGTCAAGTTTGAAGGTGATACCCTTGTTAATAGAATCGAGTTAAAAGGTATTGATTTTAAAGAAGATGGAAACATTCTTGGACACAAAATGGAATACAACTATAACTCACATAATGTATACATCATGGCAGACAAACCAAAGAATGGAATCAAAGTTAACTTCAAAA 598
42   CAAAGATGACGGGAACTACAAGACACGTGCTGAAGTCAAGTTTGAAGGTGATACCCTTGTTAATAGAATCGAGTTAAAAGGTATTGATTTTAAAGAAGATGGAAACATTCTTGGACACAAAATGGAATACAACTATAACTCACATAATGTATACATCATGGCAGACAAACCAAAGAATGGAATCAAAGTTAACTTCAAAA 598
43   -------------------------------------------GAAGGTGATACCCTTGTTAATAGAATCGAGTTAAAAGGTATTGATTTTAAAGAAGATGGAAACATTCTTGGACACAAAATGGAATACAACTATAACTCACATAATGTATACATCATGGCAGACAAACCAAAGAATGGAATCAAAGTTAACTTCAAAA 356
                                                *************************************************************************************************************************************************************

29   TTAGACACAACATTAAAGATGGAAGCGTTCAATTAGCAGACCATTATCAACAAAATACTCCAATTGGCGATGGCCCTGTCCTTTTACCAGACAACCATTACCTGTCCACACAATCTGCCCTTTCCAAAGATCCCAACGAAAAGAGAGATCACATGATCCTTCTTGAGTTTGTAACAGCTGCTGGGATTACACATGGCATG 799
30   TTAGACACAACATTAAAGATGGAAGCGTTCAATTAGCAGACCATTATCAACAAAATACTCCAATTGGCGATGGCCCTGTCCTTTTACCAGACAACCATTACCTGTCCACACAATCTGCCCTTTCCAAAGATCCCAACGAAAAGAGAGATCACATGATCCTTCTTGAGTTTGTAACAGCTGCTGGGATTACACATGGCATG 799
31   TTAGACACAACATTAAAGATGGAAGCGTTCAATTAGCAGACCATTATCAACAAAATACTCCAATTGGCGATGGCCCTGTCCTTTTACCAGACAACCATTACCTGTCCACACAATCTGCCCTTTCCAAAGATCCCAACGAAAAGAGAGATCACATGATCCTTCTTGAGTTTGTAACAGCTGCTGGGATTACACATGGCATG 799
32   TTAGACACAACATTAAAGATGGAAGCGTTCAATTAGCAGACCATTATCAACAAAATACTCCAATTGGCGATGGCCCTGTCCTTTTACCAGACAACCATTACCTGTCCACACAATCTGCCCTTTCCAAAGATCCCAACGAAAAGAGAGATCACATGATCCTTCTTGAGTTTGTAACAGCTGCTGGGATTACACATGGCATG 799
33   TTAGACACAACATTAAAGATGGAAGCGTTCAATTAGCAGACCATTATCAACAAAATACTCCAATTGGCGATGGCCCTGTCCTTTTACCAGACAACCATTACCTGTCCACACAATCTGCCCTTTCCAAAGATCCCAACGAAAAGAGAGATCACATGATCCTTCTTGAGTTTGTAACAGCTGCTGGGATTACACATGGCATG 799
34   TTAGACACAACATTAAAGATGGAAGCGTTCAATTAGCAGACCATTATCAACAAAATACTCCAATTGGCGATGGCCCTGTCCTTTTACCAGACAACCATTACCTGTCCACACAATCTGCCCTTTCCAAAGATCCCAACGAAAAGAGAGATCACATGATCCTTCTTGAGTTTGTAACAGCTGCTGGGATTACACATGGCATG 799
35   TTAGACACAACATTAAAGATGGAAGCGTTCAATTAGCAGACCATTATCAACAAAATACTCCAATTGGCGATGGCCCTGTCCTTTTACCAGACAACCATTACCTGTCCACACAATCTGCCCTTTCCAAAGATCCCAACGAAAAGAGAGATCACATGATCCTTCTTGAGTTTGTAACAGCTGCTGGGATTACACATGGCATG 799
36   TTAGACACAACATTAAAGATGGAAGCGTTCAATTAGCAGACCATTATCAACAAAATACTCCAATTGGCGATGGCCCTGTCCTTTTACCAGACAACCATTACCTGTCCACACAATCTGCCCTTTCCAAAGATCCCAACGAAAAGAGAGATCACATGATCCTTCTTGAGTTTGTAACAGCTGCTGGGATTACACATGGCATG 799
37   TTAGACACAACATTAAAGATGGAAGCGTTCAATTAGCAGACCATTATCAACAAAATACTCCAATTGGCGATGGCCCTGTCCTTTTACCAGACAACCATTACCTGTCCACACAATCTGCCCTTTCCAAAGATCCCAACGAAAAGAGAGATCACATGATCCTTCTTGAGTTTGTAACAGCTGCTGGGATTACACATGGCATG 799
38   TTAGACACAACATTAAAGATGGAAGCGTTCAATTAGCAGACCATTATCAACAAAATACTCCAATTGGCGATGGCCCTGTCCTTTTACCAGACAACCATTACCTGTCCACACAATCTGCCCTTTCCAAAGATCCCAACGAAAAGAGAGATCACATGATCCTTCTTGAGTTTGTAACAGCTGCTGGGATTACACATGGCATG 798
39   TTAGACACAACATTAAAGATGGAAGCGTTCAATTAGCAGACCATTATCAACAAAATACTCCAATTGGCGATGGCCCTGTCCTTTTACCAGACAACCATTACCTGTCCACACAATCTGCCCTTTCCAAAGATCCCAACGAAAAGAGAGATCACATGATCCTTCTTGAGTTTGTAACAGCTGCTGGGATTACACATGGCATG 800
40   TTAGACACAACATTAAAGATGGAAGCGTTCAATTAGCAGACCATTATCAACAAAATACTCCAATTGGCGATGGCCCTGTCCTTTTACCAGACAACCATTACCTGTCCACACAATCTGCCCTTTCCAAAGATCCCAACGAAAAGAGAGATCACATGATCCTTCTTGAGTTTGTAACAGCTGCTGGGATTACACATGGCATG 799
41   TTAGACACAACATTAAAGATGGAAGCGTTCAATTAGCAGACCATTATCAACAAAATACTCCAATTGGCGATGGCCCTGTCCTTTTACCAGACAACCATTACCTGTCCACACAATCTGCCCTTTCCAAAGATCCCAACGAAAAGAGAGATCACATGATCCTTCTTGAGTTTGTAACAGCTGCTGGGATTACACATGGCATG 798
42   TTAGACACAACATTAAAGATGGAAGCGTTCAATTAGCAGACCATTATCAACAAAATACTCCAATTGGCGATGGCCCTGTCCTTTTACCAGACAACCATTACCTGTCCACACAATCTGCCCTTTCCAAAGATCCCAACGAAAAGAGAGATCACATGATCCTTCTTGAGTTTGTAACAGCTGCTGGGATTACACATGGCATG 798
43   TTAGACACAACATTAAAGATGGAAGCGTTCAATTAGCAGACCATTATCAACAAAATACTCCAATTGGCGATGGCCCTGTCCTTTTACCAGACAACCATTACCTGTCCACACAATCTGCCCTTTCCAAAGATCCCAACGAAAAGAGAGATCACATGATCCTTCTTGAGTTTGTAACAGCTGCTGGGATTACACATGGCATG 556
     ********************************************************************************************************************************************************************************************************

                    AflII
29   GATGAACTATACAAACTTAAGGTTGTTGGTGGGTGCACTAACCTAA 845
30   GATGAACTATACAAACTTAAGGTTGTTGGTGGGTGCACTAACCTAA 845
31   GATGAACTATACAAACTTAAGGTTGTTGGTGGGTGCACTAACCTAA 845
32   GATGAACTATACAAACTTAAGGTTGTTGGTGGGTGCACTAACCTAA 845
33   GATGAACTATACAAACTTAAGGTTGTTGGTGGGTGCACTAACCTAA 845
34   GATGAACTATACAAACTTAAGGTTGTTGGTGGGTGCACTAACCTAA 845
35   GATGAACTATACAAACTTAAGGTTGTTGGTGGGTGCACTAACCTAA 845
36   GATGAACTATACAAACTTAAGGTTGTTGGTGGGTGCACTAACCTAA 845
37   GATGAACTATACAAACTTAAGGTTGTTGGTGGGTGCACTAACCTAA 845
38   GATGAACTATACAAACTTAAGGTTGTTGGTGGGTGCACTAACCTAA 844
39   GATGAACTATACAAACTTAAGGTTGTTGGTGGGTGCACTAACCTAA 846
40   GATGAACTATACAAATTTAAGGTTGTTGGTGGGTGCACTAACCTAA 845
41   GATGAACTATACAAACTTAAGGTTGTTGGTGGGTGCACTAACCTAA 844
42   GATGAACTATACAAACTTAAGGTTGTTGGTGGGTGCACTAACCTAA 844
43   GATGAACTATACAAACTTAAGGTTGTTGGTGGGTGCACTAACCTAA 602
     *************** ******************************
```

2nd PCA-*gfp* assembly (P_{BAD})

```
                                                   NheI                      PBAD
44    AACCTATAATAAAAAAGAAAAAGGAGAGCTAGCAAACTACCTGACGCTTTTTATCGCAACTCTCTACTGTTTCTGGTC-CGTAGGACGGTGTAAAGGAGAG-AAAGCATGAGTAAAGGAGAAGAACTTTTCACTGGAGTTGTCCCAATTCTTGTTGAATTAGATGGCGATGTTAATGGGCAAAAATTCTCTGTCAGTGGA 198
45    AACCTATAATAAAAAAGAAAAAGGAGAGCTAGCAAACTACCTGACGCTTTTTATCGCAACTCTCTACTGTTTCTGGTC-CGTAGGACGGTGTAAAGGAGAGGAAAGCATGAGTAAAGGAGAAGAACTTTTCACTGGAGTTGTCCCAATTCTTGTTGAATTAGATGGCGATGTTAATGGGCAAAAATTCTCTGTCAGTGGA 199
46    AACCTATAATAAAAAAGAAAAAGGAGAGCTAGCAAACTACCTGACGCTTTTTATCGCAACTCTCTACTGTTTCTGGTC-CGTAGGACGGTGTAAAGGAGAG-AAAGCATGAGTAAAGGAGAAGAACTTTTCACTGGAGTTGTCCCAATTCTTGTTGAATTAGATGGCGATGTTAATGGGCAAAAATTCTCTGTCAGTGGA 198
47    AACCTATAATAAAAAAGAAAAAGGAGAGCTAGCAAACTACCTGACGCTTTTTATCGCAACTCTCTACTGTTTCTGGTC-CGTAGGACGGTGTAAAGGAGAG-AAAGCATGAGTAAAGGAGAAGAACTTTTCACTGGAGTTGTCCCAATTCTTGTTGAATTAGATGGCGATGTTAATGGGCAAAAATTCTCTGTCAGTGGA 198
48    AACCTATAATAAAAAGGAAAAAGGAGAGCTAGCAAACTACCTGACGCTTTTTATCGCAACTCTCTACTGTTTCTGGTC-CGTAGGACGGTGTAAAGGAGAG-AAAGCATGAGTAAAGGAGAAGAACTTTTCACTGGAGTTGTCCCAATTCTTGTTGAATTAGATGGCGATGTTAATGGGCAAAAATTCTCTGTCAGTGGA 198
49    AACCTATAATAAAAAAGAAAAAGGAGAGCTAGCAAACTACCTGACGCTTTTTATCGCAACTCTCTACTGTTTCTGGTC-CGTAGGACGGTGTAAAGGAGAG-AAAGCATGAGTAAAGGAGAAGAACTTTTCACTGGAGTTGTCCCAATTCTTGTTGAATTAGATGGCGATGTTAATGGGCAAAAATTCTCTGTCAGTGGA 198
50    AACCTATAATAAAAAAGAAAAAGGAGAGCTAGCAAACTACCTGACGCTTTTTATCGCAACTCTCTACTGTTTCTGGTC-CGTAGGACGGTGTAAAGGAGAG-AAAGCATGAGTAAAGGAGAAGAACTTTTCACTGGAGTTGTCCCAATTCTTGTTGAATTAGATGGCGATGTTAATGGGCAAAAATTCTCTGTCAGTGGA 198
51    AACCTATAATAAAAAAGAAAAAGGAGAGCTAGCAAACTACCTGACGCTTTTTATCGCAACTCTCTACTGTTTCTGGTC-CGTAGGACGGTGTAAAGGAGAG-AAAGCATGAGTAAAGGAGAAGAACTTTTCACTGGAGTTGTCCCAATTCTTGTTGAATTAGATGGCGATGTTAATGGGCAAAAATTCTCTGTCAGTGGA 198
52    AACCTATAATAAAAAAGAAAAAGGAGAGCTAGCAAACTACCTGACGCTTTTTATCGCAACTCTCTACTGTTTCTGGTC-CGTAGGACGGTGTAAAGGAGAG-AAAGCATGAGTAAAGGAGAAGAACTTTTCACTGGAGTTGTCCCAATTCTTGTTGAATTAGATGGCGATGTTAATGGGCAAAAATTCTCTGTCAGTGGA 198
53    AACCTATAATAAAAAAGAAAAAGGAGAGCTAGCAAACTACCTGACGCTTTTTATCGCAACTCTCTACTGTTTCTGGTC-CGTAGGACGGTGTAAAGGAGAG-AAAGCATGAGTAAAGGAGAAGAACTTTTCACTGGAGTTGTCCCAATTCTTGTTGAATTAGATGGCGATGTTAATGGGCAAAAATTCTCTGTCAGTGGA 198
54    AACCTATAATAAAAAAGAAAAAGGAGAGCTAGCAAACTACCTGACGCTTTTTATCGCAACTCTCTACTGTTTCTGGTC-CGTAGGACGGTGTAAAGGAGAG-AAAGCATGAGTAAAGGAGAAGAACTTTTCACTGGAGTTGTCCCAATTCTTGTTGAATTAGATGGCGATGTTAATGGGCAAAAATTCTCTGTCAGTGGA 198
55    AACCTATAATAAAAAAGAAAAAGGAGAGCTAGCAAACTACCTGACGCTTTTTATCGCAACTCTCTACTGTTTCTGGTC-CGTAGGACGGTGTAAAGGAGAG-AAAGCATGAGTAAAGGAGAAGAACTTTTCACTGGAGTTGTCCCAATTCTTGTTGAATTAGATGGCGATGTTAATGGGCAAAAATTCTCTGTCAGTGGA 198
56    AACCTATAATAAAAAAGAAAAAGGAGAGCTAGCAAACTACCTGACGCTTTTTATCGCAACTCTCTACTGTTTCTGGTCCCGTAGGACGGTGTAAAGGAGAG-AAAGCATGAGTAAAGGAGAAGAACTTTTCACTGGAGTTGTCCCAATTCTTGTTGAATTAGATGGCGATGTTAATGGGCAAAAATTCTCTGTCAGTGGA 199
57    AACCTATAATAAAAAAGAAAAAGGAGAGCTAGCAAACTACCTGACGCTTTTTATCGCAACTCTCTACTGTTTCTGGTC-CGTAGGACGGTGTAAAGGAGAG-AAAGCATGAGTAAAGGAGAAGAACTTTTCACTGGAGTTGTCCCAATTCTTGTTGAATTAGATGGCGATGTTAATGGGCAAAAATTCTCTGTCAGTGGA 198
58    AACCTATAATAAAAAAGAAAAAGGAGAGCTAGCAAACTACCTGACGCTTTTTATCGCAACTCTCTACTGTTTCTGGTC-CGTAGGACGGTGTAAAGGAGAG-AAAGCATGAGTAAAGGAGAAGAACTTTTCACTGGAGTTGTCCCAATTCTTGTTGAATTAGATGGCGATGTTAATGGGCAAAAATTCTCTGTCAGTGGA 198
      *************** ************************************************************** ********************** **************************************************************************************************

44    GAGGGTGAAGGTGATGCAACATACGGAAAACTTACCCTTAAATTTATTTGCACTACTGGGAAGCTACCTGTTCCATGGCCAACACTTGTCACTACTTTCTCTTATGGTGTTCAATGCTTTTCAAGATACCCAGATCATATGAAACAGCATGACTTTTTCAAGAGTGCCATGCCCGAAGGTTATGTACAGGAAAGAACTAT 398
45    GAGGGTGAAGGTGATGCAACATACGGAAAACTTACCCTTAAATTTATTTGCACTACTGGGAAGCTACCTGTTCCATGGCCAACACTTGTCACTACTTTCTCTTATGGTGTTCAATGCTTTTCAAGATACCCAGATCATATGAAACAGCATGACTTTTTCAAGAGTGCCATGCCCGAAGGTTATGTACAGGAAAGAACTAT 399
46    GAGGGTGAAGGTGATGCAACATACGGAAAACTTACCCTTAAATTTATTTGCACTACTGGGAAGCTACCTGTTCCATGGCCAACACTTGTCACTACTTTCTCTTATGGTGTTCAATGCTTTTCAAGATACCCAGATCATATGAAACAGCATGACTTTTTCAAGAGTGCCATGCCCGAAGGTTATGTACAGGAAAGAACTAT 398
47    GAGGGTGAAGGTGATGCAACATACGGAAAACTTACCCTTAAATTTATTTGCACTACTGGGAAGCTACCTGTTCCATGGCCAACACTTGTCACTACTTTCTCTTATGGTGTTCAATGCTTTTCAAGATACCCAGATCATATGAAACAGCATGACTTTTTCAAGAGTGCCATGCCCGAAGGTTATGTACAGGAAAGAACTAT 398
48    GAGGGTGAAGGTGATGCAACATACGGAAAACTTACCCTTAAATTTATTTGCACTACTGGGAAGCTACCTGTTCCATGGCCAACACTTGTCACTACTTTCTCTTATGGTGTTCAATGCTTTTCAAGATACCCAGATCATATGAAACAGCATGACTTTTTCAAGAGTGCCATGCCCGAAGGTTATGTACAGGAAAGAACTAT 398
49    GAGGGTGAAGGTGATGCAACATACGGAAAACTTACCCTTAAATTTATTTGCACTACTGGGAAGCTACCTGTTCCATGGCCAACACTTGTCACTACTTTCTCTTATGGTGTTCAATGCTTTTCAAGATACCCAGATCATATGAAACAGCATGACTTTTTCAAGAGTGCCATGCCCGAAGGTTATGTACAGGAAAGAACTAT 398
50    GAGGGTGAAGGTGATGCAACATACGGAAAACTTACCCTTAAATTTATTTGCACTACTGGGAAGCTACCTGTTCCATGGCCAACACTTGTCACTACTTTCTCTTATGGTGTTCAATGCTTTTCAAGATACCCAGATCATATGAAACAGCATGACTTTTTCAAGAGTGCCATGCCCGAAGGTTATGTACAGGAAAGAACTAT 398
51    GAGGGTGAAGGTGATGCAACATACGGAAAACTTACCCTTAAATTTATTTGCACTACTGGGAAGCTACCTGTTCCATGGCCAACACTTGTCACTACTTTCTCTTATGGTGTTCAATGCTTTTCAAGATACCCAGATCATATGAAACAGCATGACTTTTTCAAGAGTGCCATGCCCGAAGGTTATGTACAGGAAAGAACTAT 398
52    GAGGGTGAAGGTGATGCAACATACGGAAAACTTACCCTTAAATTTATTTGCACTACTGGGAAGCTACCTGTTCCATGGCCAACACTTGTCACTACTTTCTCTTATGGTGTTCAATGCTTTTCAAGATACCCAGATCATATGAAACAGCATGACTTTTTCAAGAGTGCCATGCCCGAAGGTTATGTACAGGAAAGAACTAT 398
53    GAGGGTGAAGGTGATGCAACATACGGAAAACTTACCCTTAAATTTATTTGCACTACTGGGAAGCTACCTGTTCCATGGCCAACACTTGTCACTACTTTCTCTTATGGTGTTCAATGCTTTTCAAGATACCCAGATCATATGAAACAGCATGACTTTTTCAAGAGTGCCATGCCCGAAGGTTATGTACAGGAAAGAACTAT 398
54    GAGGGTGAAGGTGATGCAACATACGGAAAACTTACCCTTAAATTTATTTGCACTACTGGGAAGCTACCTGTTCCATGGCCAACACTTGTCACTACTTTCTCTTATGGTGTTCAATGCTTTTCAAGATACCCAGATCATATGAAACAGCATGACTTTTTCAAGAGTGCCATGCCCGAAGGTTATGTACAGGAAAGAACTAT 398
55    GAGGGTGAAGGTGATGCAACATACGGAAAACTTACCCTTAAATTTATTTGCACTACTGGGAAGCTACCTGTTCCATGGCCAACACTTGTCACTACTTTCTCTTATGGTGTTCAATGCTTTTCAAGATACCCAGATCATATGAAACAGCATGACTTTTTCAAGAGTGCCATGCCCGAAGGTTATGTACAGGAAAGAACTAT 398
56    GAGGGTGAAGGTGATGCAACATACGGAAAACTTACCCTTAAATTTATTTGCACTACTGGGAAGCTACCTGTTCCATGGCCAACACTTGTCACTACTTTCTCTTATGGTGTTCAATGCTTTTCAAGATACCCAGATCATATGAAACAGCATGACTTTTTCAAGAGTGCCATGCCCGAAGGTTATGTACAGGAAAGAACTAT 399
57    GAGGGTGAAG---ATGCAACATACGGAAAACTTACCCTTAAATTTATTTGCACTACTGGGAAGCTACCTGTTCCATGGCCAACACTTGTCACTACTTTCTCTTATGGTGTTCAATGCTTTTCAAGATACCCAGATCATATGAAACAGCATGACTTTTTCAAGAGTGCCATGCCCGAAGGTTATGTACAGGAAAGAACTAT 395
58    GAGGGTGAAGGTGATGCAACATACGGAAAACTTACCCTTAAATTTATTTGCACTACTGGGAAGCTACCTGTTCCATGGCCAACACTTGTCACTACTTTCTCTTATGGTGTTCAATGCTTTTCAAGATACCCAGATCATATGAAACAGCATGACTTTTTCAAGAGTGCCATGCCCGAAGGTTATGTACAGGAAAGAACTA- 397
      **********   ******************************************************************************************************************************************************************************************

44    ATTTTACAAAGATGACGGGAACTACAAGACACGTGCTGAAGTCAAGTT-TGAAGGTGATACCCTTGTTAATAGAATCGAGTTAAAAGGTATTGATTTTAAAGAAGATGGAAACATTCTTGGACACAAAATGGAATACAACTATAACTCACATAATGTATACATCATGGCAGACAAACCAAAGAATGGAATCAAAGTTAAC 597
45    ATTTTACAAAGATGACGGGAACTACAAGACACGTGCTGAAGTCAAGTT-TGAAGGTGATACCCTTGTTAATAGAATCGAGTTAAAAGGTATTGATTTTAAAGAAGATGGAAACATTCTTGGACACAAAATGGAATACAACTATAACTCACATAATGTATACATCATGGCAGACAAACCAAAGAATGGAATCAAAGTTAAC 598
46    ATTTTACAAAGATGACGGGAACTACAAGACACGTGCTGAAGTCAAGTT-TGAAGGTGATACCCTTGTTAATAGAATCGAGTTAAAAGGTATTGATTTTAAAGAAGATGGAAACATTCTTGGACACAAAATGGAATACAACTATAACTCACATAATGTATACATCATGGCAGACAAACCAAAGAATGGAATCAAAGTTAAC 597
47    ATTTTACAAAGATGACGGGAACTACAAGACACGTGCTGAAGTCAAGTT-TGAAGGTGATACCCTTGTTAATAGAATCGAGTTAAAAGGTATTGATTTTAAAGAAGATGGAAACATTCTTGGACACAAAATGGAATACAACTATAACTCACATAATGTATACATCATGGCAGACAAACCAAAGAATGGAATCAAAGTTAAC 597
48    ATTTTACAAAGATGACGGGAACTACAAGACACGTGCTGAAGTCAAGTT-TGAAGGTGATACCCTTGTTAATAGAATCGAGTTAAAAGGTATTGATTTTAAAGAAGATGGAAACATTCTTGGACACAAAATGGAATACAACTATAACTCACATAATGTATACATCATGGCAGACAAACCAAAGAATGGAATCAAAGTTAAC 597
49    ATTTTACAAAGATGACGGGAACTACAAGACACGTGCTGAAGTCAAGTT-TGAAGGTGATACCCTTGTTAATAGAATCGAGTTAAAAGGTATTGATTTTAAAGAAGATGGAAACATTCTTGGACACAAAATGGAATACAACTATAACTCACATAATGTATACATCATGGCAGACAAACCAAAGAATGGAATCAAAGTTAAC 597
50    ATTTTACAAAGATGACGGGAACTACAAGACACGTGCTGAAGTCAAGTT-TGAAGGTGATACCCTTGTTAATAGAATCGAGTTAAAAGGTATTGATTTTAAAGAAGATGGAAACATTCTTGGACACAAAATGGAATACAACTATAACTCACATAATGTATACATCATGGCAGACAAACCAAAGAATGGAATCAAAGTTAAC 597
51    ATTTTACAAAGATGACGGGAACTACAAGACACGTGCTGAAGTCAAGTT-TGAAGGTGATACCCTTGTTAATAGAATCGAGTTAAAAGGTATTGATTTTAAAGAAGATGGAAACATTCTTGGACACAAAATGGAATACAACTATAACTCACATAATGTATACATCATGGCAGACAAACCAAAGAATGGAATCAAAGTTAAC 597
52    ATTTTACAAAGATGACGGGAACTACAAGACACGTGCTGAAGTCAAGTT-TGAAGGTGATACCCTTGTTAATAGAATCGAGTTAAAAGGTATTGATTTTAAAGAAGATGGAAACATTCTTGGACACAAAATGGAATACAACTATAACTCACATAATGTATACATCATGGCAGACAAACCAAAGAATGGAATCAAAGTTAAC 597
53    ATTTTACAAAGATGACGGGAACTACAAGACACGTGCTGAAGTCAAGTT-TGAAGGTGATACCCTTGTTAATAGAATCGAGTTAAAAGGTATTGATTTTAAAGAAGATGGAAACATTCTTGGACACAAAATGGAATACAACTATAACTCACATAATGTATACATCATGGCAGACAAACCAAAGAATGGAATCAAAGTTAAC 597
54    ATTTTACAAAGATGACGGGAACTACAAGACACGTGCTGAAGTCAAGTT-TGAAGGTGATACCCTTGTTAATAGAATCGAGTTAAAAGGTATTGATTTTAAAGAAGATGGAAACATTCTTGGACACAAAATGGAATACAACTATAACTCACATAATGTATACATCATGGCAGACAAACCAAAGAATGGAATCAAAGTTAAC 597
55    ATTTTACAAAGATGACGGGAACTACAAGACACGTGCTGAAGTCAAGTT-TGAAGGTGATACCCTTGTTAATAGAATCGAGTTAAAAGGTATTGATTTTAAAGAAGATGGAAACATTCTTGGACACAAAATGGAATACAACTATAACTCACATAATGTATACATCATGGCAGACAAACCAAAGAATGGAATCAAAGTTAAC 597
56    ATTTTACAAAGATGACGGGAACTACAAGACACGTGCTGAAGTCAAGTT-TGAAGGTGATACCCTTGTTAATAGAATCGAGTTAAAAGGTATTGATTTTAAAGAAGATGGAAACATTCTTGGACACAAAATGGAATACAACTATAACTCACATAATGTATACATCATGGCAGACAAACCAAAGAATGGAATCAAAGTTAAC 598
57    ATTTTACAAAGATGACGGGAACTACAAGACACGTGCTGAAGTCAAGTT-TGAAGGTGATACCCTTGTTAATAGAATCGAGTTAAAAGGTATTGATTTTAAAGAAGATGGAAACATTCTTGGACACAAAATGGAATACAACTATAACTCACATAATGTATACATCATGGCAGACAAACCAAAGAATGGAATCAAAGTTAAC 594
58    ATTTTACAAAGATGACGGGAACTACAAGACACGTGCTGAAGTCAAGTTTTGAAGGTGATACCCTTGTTAATAGAATCGAGTTAAAAGGTATTGATTTTAAAGAAGATGGAAACATTCTTGGACACAAAATGGAATACAACTATAACTCACATAATGTATACATCATGGCAGACAAACCAAAGAATGGAATCAAAGTTAAC 597
      ************************************************ *******************************************************************************************************************************************************

44    TTCAAAATTAGACACAACATTAAAGATGGAAGCGTTCAATTAGCAGACCATTATCAACAAAATACTCCAATTGGCGATGGCCCTGTCCTTTTACCAGACAACCATTACCTGTCCACACAATCTGCCCTTTCCAAAGATCCCAACGAAAAGAGAGATCACATGATCCTTCTTGAGTTTGTAACAGCTGCTGGGATTACACA 797
45    TTCAAAATTAGACACAACATTAAAGATGGAAGCGTTCAATTAGCAGACCATTATCAACAAAATACTCCAATTGGCGATGGCCCTGTCCTTTTACCAGACAACCATTACCTGTCCACACAATCTGCCCTTTCCAAAGATCCCAACGAAAAGAGAGATCACATGATCCTTCTTGAGTTTGTAACAGCTGCTGGGATTACACA 798
46    TTCAAAATTAGACACAACATTAAAGATGGAAGCGTTCAATTAGCAGACCATTATCAACAAAATACTCCAATTGGCGATGGCCCTGTCCTTTTACCAGACAACCATTACCTGTCCACACAATCTGCCCTTTCCAAAGATCCCAACGAAAAGAGAGATCACATGATCCTTCTTGAGTTTGTAACAGCTGCTGGGATTACACA 797
47    TTCAAAATTAGACACAACATTAAAGATGGAAGCGTTCAATTAGCAGACCATTATCAACAAAATACTCCAATTGGCGATGGCCCTGTCCTTTTACCAGACAACCATTACCTGTCCACACAATCTGCCCTTTCCAAAGATCCCAACGAAAAGAGAGATCACATGATCCTTCTTGAGTTTGTAACAGCTGCTGGGATTACACA 797
48    TTCAAAATTAGACACAACATTAAAGATGGAAGCGTTCAATTAGCAGACCATTATCAACAAAATACTCCAATTGGCGATGGCCCTGTCCTTTTACCAGACAACCATTACCTGTCCACACAATCTGCCCTTTCCAAAGATCCCAACGAAAAGAGAGATCACATGATCCTTCTTGAGTTTGTAACAGCTGCTGGGATTACACA 797
49    TTCAAAATTAGACACAACATTAAAGATGGAAGCGTTCAATTAGCAGACCATTATCAACAAAATACTCCAATTGGCGATGGCCCTGTCCTTTTACCAGACAACCATTACCTGTCCACACAATCTGCCCTTTCCAAAGATCCCAACGAAAAGAGAGATCACATGATCCTTCTTGAGTTTGTAACAGCTGCTGGGATTACACA 797
50    TTCAAAATTAGACACAACATTAAAGATGGAAGCGTTCAATTAGCAGACCATTATCAACAAAATACTCCAATTGGCGATGGCCCTGTCCTTTTACCAGACAACCATTACCTGTCCACACAATCTGCCCTTTCCAAAGATCCCAACGAAAAGAGAGATCACATGATCCTTCTTGAGTTTGTAACAGCTGCTGGGATTACACA 797
51    TTCAAAATTAGACACAACATTAAAGATGGAAGCGTTCAATTAGCAGACCATTATCAACAAAATACTCCAATTGGCGATGGCCCTGTCCTTTTACCAGACAACCATTACCTGTCCACACAATCTGCCCTTTCCAAAGATCCCAACGAAAAGAGAGATCACATGATCCTTCTTGAGTTTGTAACAGCTGCTGGGATTACACA 797
52    TTCAAAATTAGACACAACATTAAAGATGGAAGCGTTCAATTAGCAGACCATTATCAACAAAATACTCCAATTGGCGATGGCCCTGTCCTTTTACCAGACAACCATTACCTGTCCACACAATCTGCCCTTTCCAAAGATCCCAACGAAAAGAGAGATCACATGATCCTTCTTGAGTTTGTAACAGCTGCTGGGATTACACA 797
53    TTCAAAATTAGACACAACATTAAAGATGGAAGCGTTCAATTAGCAGACCATTATCAACAAAATACTCCAATTGGCGATGGCCCTGTCCTTTTACCAGACAACCATTACCTGTCCACACAATCTGCCCTTTCCAAAGATCCCAACGAAAAGAGAGATCACATGATCCTTCTTGAGTTTGTAACAGCTGCTGGGATTACACA 797
54    TTCAAAATTAGACACAACATTAAAGATGGAAGCGTTCAATTAGCAGACCATTATCAACAAAATACTCCAATTGGCGATGGCCCTGTCCTTTTACCAGACAACCATTACCTGTCCACACAATCTGCCCTTTCCAAAGATCCCAACGAAAAGAGAGATCACATGATCCTTCTTGAGTTTGTAACAGCTGCTGGGATTACACA 797
55    TTCAAAATTAGACACAACATTAAAGATGGAAGCGTTCAATTAGCAGACCATTATCAACAAAATACTCCAATTGGCGATGGCCCTGTCCTTTTACCAGACAACCATTACCTGTCCACACAATCTGCCCTTTCCAAAGATCCCAACGAAAAGAGAGATCACATGATCCTTCTTGAGTTTGTAACAGCTGCTGGGATTACACA 797
56    TTCAAAATTAGACACAACATTAAAGATGGAAGCGTTCAATTAGCAGACCATTATCAACAAAATACTCCAATTGGCGATGGCCCTGTCCTTTTACCAGACAACCATTACCTGTCCACACAATCTGCCCTTTCCAAAGATCCCAACGAAAAGAGAGATCACATGATCCTTCTTGAGTTTGTAACAGCTGCTGGGATTACACA 798
57    TTCAAAATTAGACACAACATTAAAGATGGAAGCGTTCAATTAGCAGACCATTATCAACAAAATACTCCAATTGGCGATGGCCCTGTCCTTTTACCAGACAACCATTACCTGTCCACACAATCTGCCCTTTCCAAAGATCCCAACGAAAAGAGAGATCACATGATCCTTCTTGAGTTTGTAACAGCTGCTGGGATTACACA 794
58    TTCAAAATTAGACACAACATTAAAGATGGAAGCGTTCAATTAGCAGACCATTATCAACAAAATACTCCAATTGGCGATGGCCCTGTCCTTTTACCAGACAACCATTACCTGTCCACACAATCTGCCCTTTCCAAAGATCCCAACGAAAAGAGAGATCACATGATCCTTCTTGAGTTTGTAACAGCTGCTGGGATTACACA 797
      ********************************************************************************************************************************************************************************************************

                            AflII
44    TGGCATGGATGAACTATACAAACTTAAGGTTGTTGGTGGGTGCACTAACCTAA 850
45    TGGCATGGATGAACTATACAAACTTAAGGTTGTTGGTGGGTGCACTAACCTAA 851
46    TGGCATGGATGAACTATACAAACTTAAGGTTGTTGGTGGGTGCACTAACCTAA 850
47    TGGCATGGATGAACTATACAAACTTAAGGTTGTTGGTGGGTGCACTAACCTAA 850
48    TGGCATGGATGAACTATACAAACTTAAGGTTGTTGGTGGGTGCACTAACCTAA 850
49    TGGCATGGATGAACTATACAAACTTAAGGTTGTTGGTGGGTGCACTAACCTAA 850
50    TGGCATGGATGAACTATACAAACTTAAGGTTGTTGGTGGGTGCACTAACCTAA 850
51    TGGCATGGATGAACTATACAAACTTAAGGTTGTTGGTGGGTGCACTAACCTAA 850
52    TGGCATGGATGAACTATACAAACTTAAGGTTGTTGGTGGGTGCACTAACCTAA 850
53    TGGCATGGATGAACTATACAAACTTAAGGTTGTTGGTGGGTGCACTAACCTAA 850
54    TGGCATGGATGAACTATACAAACTTAAGGTTGTTGGTGGGTGCACTAACCTAA 850
55    TGGCATGGATGAACTATACAAACTTAAGGTTGTTGGTGGGTGCACTAACCTAA 850
56    TGGCATGGATGAACTATACAAACTTAAGGTTGTTGGTGGGTGCACTAACCTAA 851
57    TGGCATGGATGAACTATACAAACTTAAGGTTGTTGGTGGGTGCACTAACCTAA 847
58    TGGCATGGATGAACTATACAAACTTAAGGTTGTTGGTGGGTGCGCTAACCTAA 850
      ******************************************* *********
```

2nd PCA-*gfp* assembly (P$_{tac}$)

```
                                   NheI               Ptac
59  AACCTATAATAAA-AAAGAAAAAGGAGAGCTAGCAAAGCTGTTGACAATTAATCATCGGCTCGTATAATGTGTGGTCCGTAGGACGGTGTAAAGGAGAGAAAGCATGAGTAAAGGAGAAGAACTTTTCACTGGAGTTGTCCCAATTCTTGTTGAATTAGATGGCGATGTTAATGGGCAAAAATTCTCTGTCAGTGGAGAG 199
60  AACCTATAATAAA-AAAGAAAAAGGAGAGCTAGCAAAGCTGTTGACAATTAATCATCGGCTCGTATAATGTGTGGTCCGTAGGACGGTGTAAAGGAGAGAAAGCATGAGTAAAGGAGAAGAACTTTTCACTGGAGTTGTCCCAATTCTTGTTGAATTAGATGGCGATGTTAATGGGCAAAAATTCTCTGTCAGTGGAGAG 199
61  AACCTATAATAAA-AAAGAAAAAGGAGAGCTAGCAAAGCTGTTGACAATTAATCATCGGCTCGTATAATGTGTGGTCCGTAGGACGGTGTAAAGGAGAGAAAGCATGAGTAAAGGAGAAGAACTTTTCACTGGAGTTGTCCCAATTCTTGTTGAATTAGATGGCGATGTTAATGGGCAAAAATTCTCTGTCAGTGGAGAG 199
62  AACCTATAATAAA-AAAGAAAAAGGAGAGCTAGCAAAGCTGTTGACAATTAATCATCGGCTCGTATAATGTGTGGTCCGTAGGACGGTGTAAAGGAGAGAAAGCATGAGTAAAGGAGAAGAACTTTTCACTGGAGTTGTCCCAATTCTTGTTGAATTAGATGGCGATGTTAATGGGCAAAAATTCTCTGTCAGTGGAGAG 199
63  AACCTATAATAAA-AAAGAAAAAGGAGAGCTAGCAAAGCTGTTGACAATTAATCATCGGCTCGTATAATGTGTGGTCCGTAGGACGGTGTAAAGGAGAGAAAGCATGAGTAAAGGAGAAGAACTTTTCACTGGAGTTGTCCCAATTCTTGTTGAATTAGATGGCGATGTTAATGGGCAAAAATTCTCTGTCAGTGGAGAG 199
64  AACCTATAATAAA-AAAGAAAAAGGAGAGCTAGCAAAGCTGTTGACAATTAATCATCGGCTCGTATAATGTGTGGTCCGTAGGACGGTGTAAAGGAGAGAAAGCATGAGTAAAGGAGAAGAACTTTTCACTGGAGTTGTCCCAATTCTTGTTGAATTAGATGGCGATGTTAATGGGCAAAAATTCTCTGTCAGTGGAGAG 199
65  AACCTATAATAAA-AAAGAAAAAGGAGAGCTAGCAAAGCTGTTGACAATTAATCATCGGCTCGTATAATGTGTGGTCCGTAGGACGGTGTAAAGGAGAGAAAGCATGAGTAAAGGAGAAGAACTTTTCACTGGAGTTGTCCCAATTCTTGTTGAATTAGATGGCGATGTTAATGGGCAAAAATTCTCTGTCAGTGGAGAG 199
66  AACCTATAATAAA-AAAGAAAAAGGAGAGCTAGCAAAGCTGTTGACAATTAATCATCGGCTCGTATAATGTGTGGTCCGTAGGACGGTGTAAAGGAGAGAAAGCATGAGTAAAGGAGAAGAACTTTTCACTGGAGTTGTCCCAATTCTTGTTGAATTAGATGGCGATGTTAATGGGCAAAAATTCTCTGTCAGTGGAGAG 199
67  AACCTATAATAAA-AAAGAAAAAGGAGAGCTAGCAAAGCTGTTGACAATTAATCATCGGCTCGTATAATGTGTGGTCCGTAGGACGGTGTAAAGGAGAGAAAGCATGAGTAAAGGAGAAGAACTTTTCACTGGAGTTGTCCCAATTCTTGTTGAATTAGATGGCGATGTTAATGGGCAAAAATTCTCTGTCAGTGGAGAG 199
68  AACCTATAATAAAAAAAGAAAAAGGAGAGCTAGCAAAGCTGTTGACAATTAATCATCGGCTCGTATAATGTGTGGTCCGTAGGACGGTGTAAAGGAGAGAAAGCATGAGTAAAGGAGAAGAACTTTTCACTGGAGTTGTCCCAATTCTTGTTGAATTAGATGGCGATGTTAATGGGCAAAAATTCTCTGTCAGTGGAGAG 200
69  AACCTATAATAAA-AAAGAAAAAAGAGAGCTAGCAAAGCTGTTGACAATTAATCATCGGCTCGTATAATGTGTGGTCCGTAGGACGGTGTAAAGGAGAGAAAGCATGAGTAAAGGAGAAGAACTTTTCACTGGAGTTGTCCCAATTCTTGTTGAATTAGATGGCGATGTTAATGGGCAAAAATTCTCTGTCAGTGGAGAG 199
70  AACCTATAATAAA-AAAGAAAAAGGAGAGCTAGCAAAGCTGTTGACAATTAATCATCGGCTCGTATAATGTGTGGTCCGTAGGACGGTGTAAAGGAGAGAAAGCATGAGTAAAGGAGAAGAACTTTTCACTGGAGTTGTCCCAATTCTTGTTGAATTAGATGGCGATGTTAATGGGCAAAAATTCTCTGTCAGTGGAGAG 199
71  AACCTATAATAAA-AAAGAAAAAGGAGAGCTAGCAAAGCTGTTGACAATTAATCATCGGCTCGTATAATGTGTGGTCCGTAGGACGGTGTAAAGGAGAGAAAGCATGAGTAAAGGAGAAGAACTTTTCACTGGAGTTGTCC----------------------------------------------------------- 140
72  AACCTATAATAAA-AAAGAAAAAGGAGAGCTAGCAAAGCTGTTGACAATTAATCATCGGCTCGTATAATGTGTGGTCCGTAGGACGGTGTAAAGGAGAGAAAGCATGAGCAAAGGAGAAGAAC----------------------------------------------------------------------------- 122
73  AACCTATAATAAA-AAAGAAAAAGGAGAGCTAGCAAAGCTGTTGACAATTAATCATCGGCTCGTATAATGTGTGGTCCGTAGGACGGTGTAAAGGAGAGAAAGCATGAGTAAAGGAGAAGAACTTTTCACTGGAGTTGTCCCAATTCTTGTTGAATTAGATGGCGATGTTAATGGGCAAAAATTCTCTGTCAGTGGAGAG 199
    ************* ********* ************************************************************************************* *************

59  GGTGAAGGTGATGCAACATACGGAAAACTTACCCTTAAATTTATTTGCACTACTGGGAAGCTACCTGTTCCATGGCCAACACTTGTCACTACTTTCTCTTATGGTGTTCAATGCTTTTCAAGATACCCAGATCATATGAAACAGCATGACTTTTTCAAGAGTGCCATGCCCGAAGGTTATGTACAGGAAAGAACTATATT 399
60  GGTGAAGGTGATGCAACATACGGAAAACTTACCCTTAAATTTATTTGCACTACTGGGAAGCTACCTGTTCCATGGCCAACACTTGTCACTACTTTCTCTTATGGTGTTCAATGCTTTTCAAGATACCCAGATCATATGAAACAGCATGACTTTTTCAAGAGTGCCATGCCCGAAGGTTATGTACAGGAAAGAACTATATT 399
61  GGTGAAGGTGATGCAACATACGGAAAACTTACCCTTAAATTTATTTGCACTACTGGGAAGCTACCTGTTCCATGGCCAACACTTGTCACTACTTTCTCTTATGGTGTTCAATGCTTTTCAAGATACCCAGATCATATGAAACAGCATGACTTTTTCAAGAGTGCCATGCCCGAAGGTTATGTACAGGAAAGAACTATATT 399
62  GGTGAAGGTGATGCAACATACGGAAAACTTACCCTTAAATTTATTTGCACTACTGGGAAGCTACCTGTTCCATGGCCAACACTTGTCACTACTTTCTCTTATGGTGTTCAATGCTTTTCAAGATACCCAGATCATATGAAACAGCATGACTTTTTCAAGAGTGCCATGCCCGAAGGTTATGTACAGGAAAGAACTATATT 399
63  GGTGAAGGTGATGCAACATACGGAAAACTTACCCTTAAATTTATTTGCACTACTGGGAAGCTACCTGTTCCATGGCCAACACTTGTCACTACTTTCTCTTATGGTGTTCAATGCTTTTCAAGATACCCAGATCATATGAAACAGCATGACTTTTTCAAGAGTGCCATGCCCGAAGGTTATGTACAGGAAAGAACTATATT 399
64  GGTGAAGGTGATGCAACATACGGAAAACTTACCCTTAAATTTATTTGCACTACTGGGAAGCTACCTGTTCCATGGCCAACACTTGTCACTACTTTCTCTTATGGTGTTCAATGCTTTTCAAGATACCCAGATCATATGAAACAGCATGACTTTTTCAAGAGTGCCATGCCCGAAGGTTATGTACAGGAAAGAACTATATT 399
65  GGTGAAGGTGATGCAACATACGGAAAACTTACCCTTAAATTTATTTGCACTACTGGGAAGCTACCTGTTCCATGGCCAACACTTGTCACTACTTTCTCTTATGGTGTTCAATGCTTTTCAAGATACCCAGATCATATGAAACAGCATGACTTTTTCAAGAGTGCCATGCCCGAAGGTTATGTACAGGAAAGAACTATATT 399
66  GGTGAAGGTGATGCAACATACGGAAAACTTACCCTTAAATTTATTTGCACTACTGGGAAGCTACCTGTTCCATGGCCAACACTTGTCACTACTTTCTCTTATGGTGTTCAATGCTTTTCAAGATACCCAGATCATATGAAACAGCATGACTTTTTCAAGAGTGCCATGCCCGAAGGTTATGTACAGGAAAGAACTATATT 399
67  GGTGAAGGTGATGCAACATACGGAAAACTTACCCTTAAATTTATTTGCACTACTGGGAAGCTACCTGTTCCATGGCCAACACTTGTCACTACTTTCTCTTATGGTGTTCAATGCTTTTCAAGATACCCAGATCATATGAAACAGCATGACTTTTTCAAGAGTGCCATGCCCGAAGGTTATGTACAGGAAAGAACTATATT 399
68  GGTGAAGGTGATGCAACATACGGAAAACTTACCCTTAAATTTATTTGCACTACTGGGAAGCTACCTGTTCCATGGCCAACACTTGTCACTACTTTCTCTTATGGTGTTCAATGCTTTTCAAGATACCCAGATCATATGAAACAGCATGACTTTTTCAAGAGTGCCATGCCCGAAGGTTATGTACAGGAAAGAACTATATT 400
69  GGTGAAGGTGATGCAACATACGGAAAACTTACCCTTAAATTTATTTGCACTACTGGGAAGCTACCTGTTCCATGGCCAACACTTGTCACTACTTTCTCTTATGGTGTTCAATGCTTTTCAAGATACCCAGATCATATGAAACAGCATGACTTTTTCAAGAGTGCCATGCCCGAAGGTTATGTACAGGAAAGAACTATATT 399
70  GGTGAAGGTGATGCAACATACGGAAAACTTACCCTTAAATTTATTTGCACTACTGGGAAGCTACCTGTTCCATGGCCAACACTTGTCACTACTTTCTCTTATGGTGTTCAATGCTTTTCAAGATACCCAGATCATATGAAACAGCATGACTTTTTCAAGAGTGCCATGCCCGAAGGTTATGTACAGGAAAGAACTATATT 399
71  -------------------------------------------------------------------------------------------------------------------------------------------------------------------------------------------------------- 140
72  -------------------------------------------------------------------------------------------------------------------------------------------------------------------------------------------------------- 122
73  GGTGAAGGTGATGCAACATACGGAAAACTTACCCTTAAATTTATTTGCACTACTGGGAAGCTACCTGTTCCATGGCCAACACTTGTCACTACTTTCTCTTATGGTGTTCAATGCTTTTCAAGATACCCAGATCATATGAAACAGCATGACTTTTTCAAGAGTGCCATGCCCGAAGGTTATGTACAGGAAAGAACTATATT 399

59  TTACAAAGATGACGGGAACTACAAGACACGTGCTGAAGTCAAGTTTGAAGGTGATACCCTTGTTAATAGAATCGAGTTAAAAGGTATTGATTTTAAAGAAGATGGAAACATTCTTGGACACAAAATGGAATACAACTATAACTCACATAATGTATACATCATGGCAGACAAACCAAAGAATGGAATCAAAGTTAACTTCA 599
60  TTACAAAGATGACGGGAACTACAAGACACGTGCTGAAGTCAAGTTTGAAGGTGATACCCTTGTTAATAGAATCGAGTTAAAAGGTATTGATTTTAAAGAAGATGGAAACATTCTTGGACACAAAATGGAATACAACTATAACTCACATAATGTATACATCATGGCAGACAAACCAAAGAATGGAATCAAAGTTAACTTCA 599
61  TTACAAAGATGACGGGAACTACAAGACACGTGCTGAAGTCAAGTTTGAAGGTGATACCCTTGTTAATAGAATCGAGTTAAAAGGTATTGATTTTAAAGAAGATGGAAACATTCTTGGACACAAAATGGAATACAACTATAACTCACATAATGTATACATCATGGCAGACAAACCAAAGAATGGAATCAAAGTTAACTTCA 599
62  TTACAAAGATGACGGGAACTACAAGACACGTGCTGAAGTCAAGTTTGAAGGTGATACCCTTGTTAATAGAATCGAGTTAAAAGGTATTGATTTTAAAGAAGATGGAAACATTCTTGGACACAAAATGGAATACAACTATAACTCACATAATGTATACATCATGGCAGACAAACCAAAGAATGGAATCAAAGTTAACTTCA 599
63  TTACAAAGATGACGGGAACTACAAGACACGTGCTGAAGTCAAGTTTGAAGGTGATACCCTTGTTAATAGAATCGAGTTAAAAGGTATTGATTTTAAAGAAGATGGAAACATTCTTGGACACAAAATGGAATACAACTATAACTCACATAATGTATACATCATGGCAGACAAACCAAAGAATGGAATCAAAGTTAACTTCA 599
64  TTACAAAGATGACGGGAACTACAAGACACGTGCTGAAGTCAAGTTTGAAGGTGATACCCTTGTTAATAGAATCGAGTTAAAAGGTATTGATTTTAAAGAAGATGGAAACATTCTTGGACACAAAATGGAATACAACTATAACTCACATAATGTATACATCATGGCAGACAAACCAAAGAATGGAATCAAAGTTAACTTCA 599
65  TTACAAAGATGACGGGAACTACAAGACACGTGCTGAAGTCAAGTTTGAAGGTGATACCCTTGTTAATAGAATCGAGTTAAAAGGTATTGATTTTAAAGAAGATGGAAACATTCTTGGACACAAAATGGAATACAACTATAACTCACATAATGTATACATCATGGCAGACAAACCAAAGAATGGAATCAAAGTTAACTTCA 599
66  TTACAAAGATGACGGGAACTACAAGACACGTGCTGAAGTCAAGTTTGAAGGTGATACCCTTGTTAATAGAATCGAGTTAAAAGGTATTGATTTTAAAGAAGATGGAAACATTCTTGGACACAAAATGGAATACAACTATAACTCACATAATGTATACATCATGGCAGACAAACCAAAGAATGGAATCAAAGTTAACTTCA 599
67  TTACAAAGATGACGGGAACTACAAGACACGTGCTGAAGTCAAGTTTGAAGGTGATACCCTTGTTAATAGAATCGAGTTAAAAGGTATTGATTTTAAAGAAGATGGAAACATTCTTGGACACAAAATGGAATACAACTATAACTCACATAATGTATACATCATGGCAGACAAACCAAAGAATGGAATCAAAGTTAACTTCA 599
68  TTACAAAGATGACGGGAACTACAAGACACGTGCTGAAGTCAAGTTTGAAGGTGATACCCTTGTTAATAGAATCGAGTTAAAAGGTATTGATTTTAAAGAAGATGGAAACATTCTTGGACACAAAATGGAATACAACTATAACTCACATAATGTATACATCATGGCAGACAAACCAAAGAATGGAATCAAAGTTAACTTCA 600
69  TTACAAAGATGACGGGAACTACAAGACACGTGCTGAAGTCAAGTTTGAAGGTGATACCCTTGTTAATAGAATCGAGTTAAAAGGTATTGATTTTAAAGAAGATGGAAACATTCTTGGACACAAAATGGAATACAACTATAACTCACATAATGTATACATCATGGCAGACAAACCAAAGAATGGAATCAAAGTTAACTTCA 599
70  TTACAAAGATGACGGGAACTACAAGACACGTGCTGAAGTCAAGTTTGAAGGTGATACCCTTGTTAATAGAATCGAGTTAAAAGGTATTGATTTTAAAGAAGATGGAAACATTCTTGGACACAAAATGGAATACAACTATAACTCACATAATGTATACATCATGGCAGACAAACCAAAGAATGGAATCAAAGTTAACTTCA 599
71  -------------------------------------------------------------------------------------------------------------------------------------------------------------------------------------------------------- 140
72  -------------------------------------------------------------------------------------------------------------------------------------------------------------------------------------------------------- 122
73  TTACAAAGATGACGGGAACTACAAGACACGTGCTGAAGTCAAGTTTGAAGGTGATACCCTTGTTAATAGAATCGAGTTAAAAGGTATTGATTTTAAAGAAGATGGAAACATTCTTGGACACAAAATGGAATACAACTATAACTCACATAATGTATACATCATGGCAGACAAACCAAAGAATGGAATCAAAGTTAACTTCA 599

59  AAATTAGACACAACATTAAAGATGGAAGCGTTCAATTAGCAGACCATTATCAACAAAATACTCCAATTGGCGATGGCCCTGTCCTTTTACCAGACAACCATTACCTGTCCACACAATCTGCCCTTTCCAAAGATCCCAACGAAAAGAGAGATCACATGATCCTTCTTGAGTTTGTAACAGCTGCTGGGATTACACATGGC 799
60  AAATTAGACACAACATTAAAGATGGAAGCGTTCAATTAGCAGACCATTATCAACAAAATACTCCAATTGGCGATGGCCCTGTCCTTTTACCAGACAACCATTACCTGTCCACACAATCTGCCCTTTCCAAAGATCCCAACGAAAAGAGAGATCACATGATCCTTCTTGAGTTTGTAACAGCTGCTGGGATTACACATGGC 799
61  AAATTAGACACAACATTAAAGATGGAAGCGTTCAATTAGCAGACCATTATCAACAAAATACTCCAATTGGCGATGGCCCTGTCCTTTTACCAGACAACCATTACCTGTCCACACAATCTGCCCTTTCCAAAGATCCCAACGAAAAGAGAGATCACATGATCCTTCTTGAGTTTGTAACAGCTGCTGGGATTACACATGGC 799
62  AAATTAGACACAACATTAAAGATGGAAGCGTTCAATTAGCAGACCATTATCAACAAAATACTCCAATTGGCGATGGCCCTGTCCTTTTACCAGACAACCATTACCTGTCCACACAATCTGCCCTTTCCAAAGATCCCAACGAAAAGAGAGATCACATGATCCTTCTTGAGTTTGTAACAGCTGCTGGGATTACACATGGC 799
63  AAATTAGACACAACATTAAAGATGGAAGCGTTCAATTAGCAGACCATTATCAACAAAATACTCCAATTGGCGATGGCCCTGTCCTTTTACCAGACAACCATTACCTGTCCACACAATCTGCCCTTTCCAAAGATCCCAACGAAAAGAGAGATCACATGATCCTTCTTGAGTTTGTAACAGCTGCTGGGATTACACATGGC 799
64  AAATTAGACACAACATTAAAGATGGAAGCGTTCAATTAGCAGACCATTATCAACAAAATACTCCAATTGGCGATGGCCCTGTCCTTTTACCAGACAACCATTACCTGTCCACACAATCTGCCCTTTCCAAAGATCCCAACGAAAAGAGAGATCACATGATCCTTCTTGAGTTTGTAACAGCTGCTGGGATTACACATGGC 799
65  AAATTAGACACAACATTAAAGATGGAAGCGTTCAATTAGCAGACCATTATCAACAAAATACTCCAATTGGCGATGGCCCTGTCCTTTTACCAGACAACCATTACCTGTCCACACAATCTGCCCTTTCCAAAGATCCCAACGAAAAGAGAGATCACATGATCCTTCTTGAGTTTGTAACAGCTGCTGGGATTACACATGGC 799
66  AAATTAGACACAACATTAAAGATGGAAGCGTTCAATTAGCAGACCATTATCAACAAAATACTCCAATTGGCGATGGCCCTGTCCTTTTACCAGACAACCATTACCTGTCCACACAATCTGCCCTTTCCAAAGATCCCAACGAAAAGAGAGATCACATGATCCTTCTTGAGTTTGTAACAGCTGCTGGGATTACACATGGC 799
67  AAATTAGACACAACATTAAAGATGGAAGCGTTCAATTAGCAGACCATTATCAACAAAATACTCCAATTGGCGATGGCCCTGTCCTTTTACCAGACAACCATTACCTGTCCACACAATCTGCCCTTTCCAAAGATCCCAACGAAAAGAGAGATCACATGATCCTTCTTGAGTTTGTAACAGCTGCTGGGATTACACATGGC 799
68  AAATTAGACACAACATTAAAGATGGAAGCGTTCAATTAGCAGACCATTATCAACAAAATACTCCAATTGGCGATGGCCCTGTCCTTTTACCAGACAACCATTACCTGTCCACACAATCTGCCCTTTCCAAAGATCCCAACGAAAAGAGAGATCACATGATCCTTCTTGAGTTTGTAACAGCTGCTGGGATTACACATGGC 800
69  AAATTAGACACAACATTAAAGATGGAAGCGTTCAATTAGCAGACCATTATCAACAAAATACTCCAATTGGCGATGGCCCTGTCCTTTTACCAGACAACCATTACCTGTCCACACAATCTGCCCTTTCCAAAGATCCCAACGAAAAGAGAGATCACATGATCCTTCTTGAGTTTGTAACAGCTGCTGGGATTACACATGGC 799
70  AAATTAGACACAACATTAAAGATGGAAGCGTTCAATTAGCAGACCATTATCAACAAAATACTCCAATTGGCGATGGCCCTGTCCTTTTACCAGACAACCAGTACCTGTCCACACAATCTGCCCTTTCCAAAGATCCCAACGAAAAGAGAGATCACATGATCCTTCTTGAGTTTGTAACAGCTGCTGGGATTACACATGGC 799
71  ------------------------------------------------------------------------------------------------------------------------------------------ACGAAAAGAGAGATCACATGATCCTTCTTGAGTTTGTAACAGCTGCTGGGATTACACATGGC 202
72  ---------------------------------------------------------------------------------------------------------------------------TTTCCAAAGATCCCAACGAAAAGAGAGATCACATGATCCTTCTTGAGTTTGTAACAGCTGCTGGGATTACACATGGC 199
73  AAATTAGACACAACATTAAAGATGGAAGCGTTCAATTAGCAGACCATTATCAACAAAATACTCCAATTGGCGATGGCCCTGTCCTTTTACCAGACAACCATTACCTGTCCACACAATCTGTCCTTTCCAAAGATCCCAACGAAAAGAGAGATCACATGATCCTTCTTGAGTTTGTAACAGCTGCTGGGATTACACATGGC 799
                                                                                                                                              **************************************************************

59  ATGGATGAACTATACAAACTTAAGGTTGTTGGTGGGTGCACTAACCTAA 848
60  ATGGATGAACTATACAAACTTAAGGTTGTTGGTGGGTGCACTAACCTAA 848
61  ATGGATGAACTATACAAACTTAAGGTTGTTGGTGGGTGCACTAACCTAA 848
62  ATGGATGAACTATACAAACTTAAGGTTGTTGGTGGGTGCACTAACCTAA 848
63  ATGGATGAACTATACAAACTTAAGGTTGTTGGTGGGTGCACTAACCTAA 848
64  ATGGATGAACTATACAAACTTAAGGTTGTTGGTGGGTGCACTAACCTAA 848
65  ATGGATGAACTATACAAACTTAAGGTTGTTGGTGGGTGCACTAACCTAA 848
66  ATGGATGAACTATACAAACTTAAGGTTGTTGGTGGGTGCACTAACCTAA 848
67  ATGGATGAACTATACAAACTTAAGGTTGTTGGTGGGTGCACTAACCTAA 848
68  ATGGATGAACTATACAAACTTAAGGTTGTTGGTGGGTGCACTAACCTAA 849
69  ATGGATGAACTATACAAACTTAAGGTTGTTGGTGGGTGCACTAACCTAA 848
70  ATGGATGAACTATACAAACTTAAGGTTGTTGGTGGGTGCACTAACCTAA 848
71  ATGGATGAACTATACAAACTTAAGGTTGTTGGTGGGTGCACTAACCTAA 251
72  ATGGATGAACTATACAAACTTAAGGTTGTTGGTGGGTGCACTAACCTAA 248
73  ATGGATGAACTATACAAACTTAAGGTTGTTGGTGGGTGCACTAACCTAA 848
    *************************************************
```

2nd PCA-*gfp* assembly (P_{recA*})(negative samples)

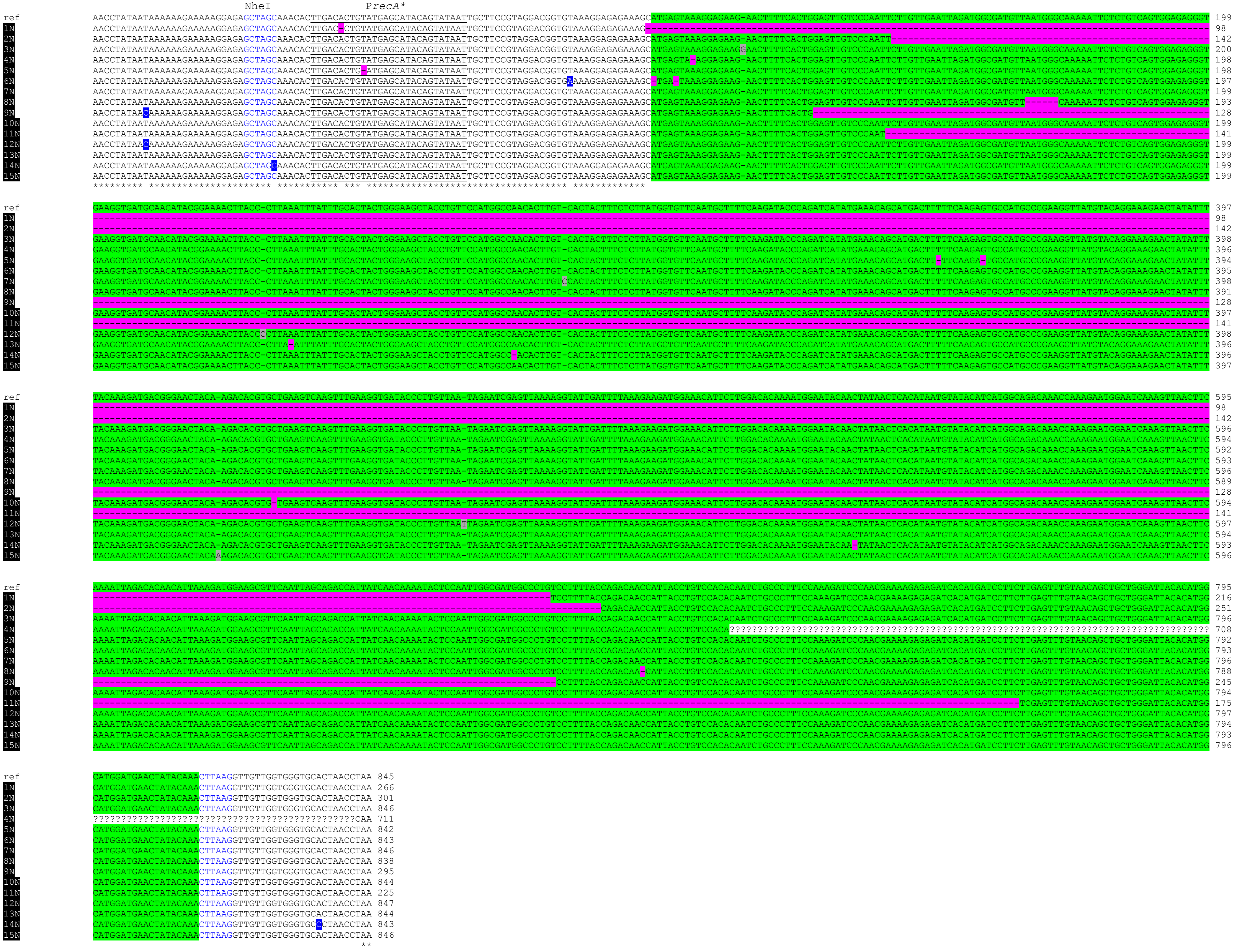

Legend:

X= base(s) inserted
X= base(s) substituted
--= base(s) deleted
?= sequence not readable
X= base(s) within the *gfp* coding sequence
X= non-correct sequence(s)
