# Supplementary material for: Terminator Operon Reporter: combining a transcription termination switch with reporter technology for improved gene synthesis and synthetic biology applications
Source: Sci Rep. 2016 May 25;6:26572. doi: 10.1038/srep26572 (PMC4879669; doi:10.1038/srep26572)
Supplement: Supplementary Data 2 [file srep26572-s3.pdf]

Supplementary Data 2

(Frame-shift mutations and emerging stop codons in the first 447 protein coding genes of *Escherichia coli* K 12 MG1655 (NCBI:NC_000913.3))

Terminator Operon Reporter: combining a transcription termination switch with reporter technology for improved gene synthesis and synthetic biology applications

Massimiliano Zampini[1*], Luis A J Mur[1], Pauline Rees Stevens[1], Justin A Pachebat[1], C James Newbold[1], Finbarr Hayes[2*] & Alison Kingston-Smith[1*]

[1] Institute of Biological, Environmental and Rural Sciences, Edward Llwyd Building, Aberystwyth University, Aberystwyth SY23 3FG, UK, [2] Faculty of Life Sciences, University of Manchester, Manchester M13 9PL, UK

*Correspondence should be addressed to M.Z. (M.Zampini@outlook.com) or F.H. (Finbarr.Hayes@manchester.ac.uk) or A. K-S. (ahk@aber.ac.uk)

In the first 447 protein coding genes derived from *E.coli* K-12 MG1655 (NCBI:NC_000913.3) a single insertion (adenine) was introduced at position 100 of the coding sequence. The amino acids translated in the new reading frame are highlighted in green (below). As two genes were shorter than 99 bp, a total of 445 genes were considered for statistical analysis as reported in Supplementary Figure S2 (and in the following figure).

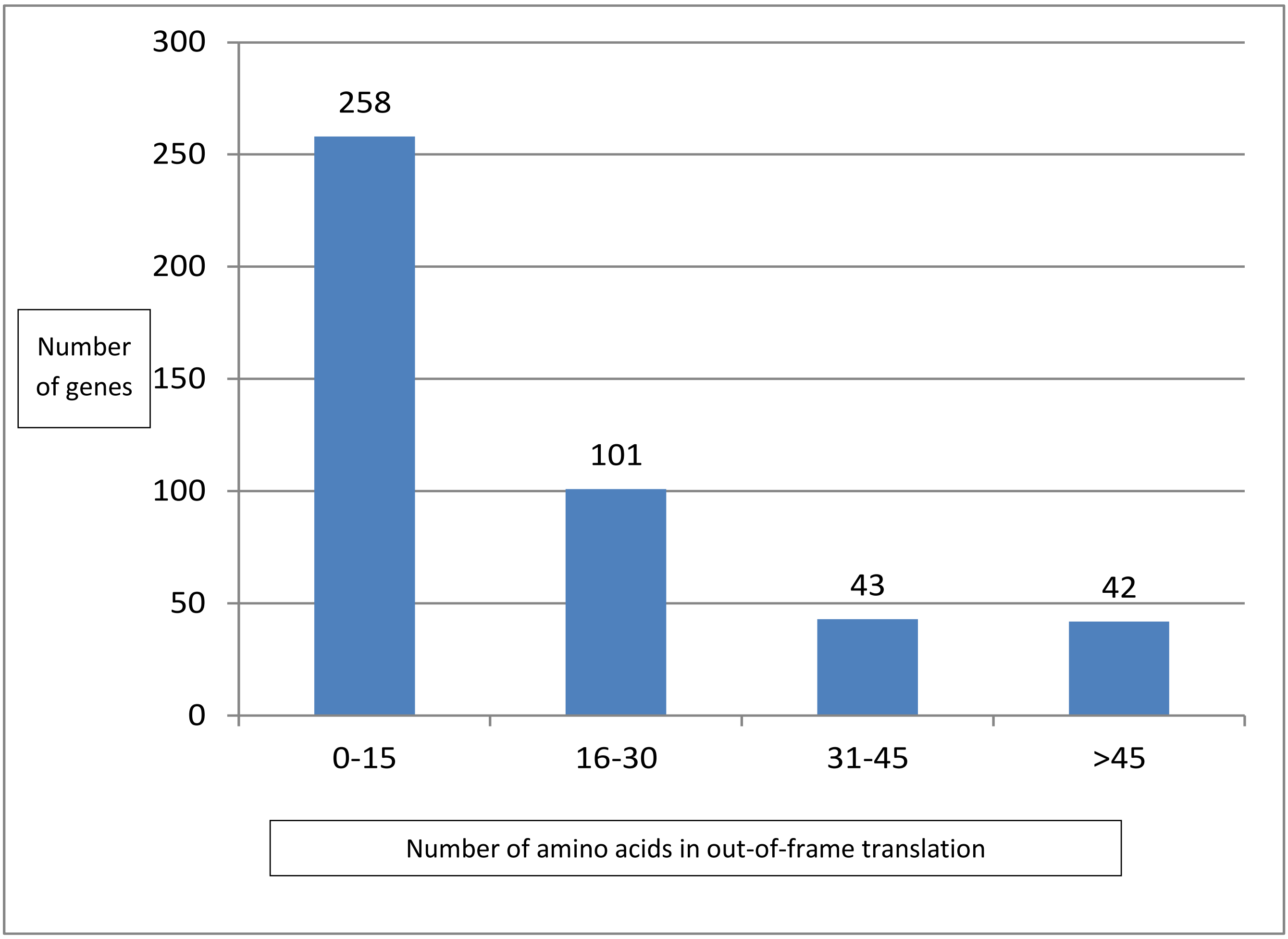

- In 258 genes the frame-shift mutation introduced from 0 to 15 new amino acids before the stop codon
- In 101 genes the frame-shift mutation introduced from 16 to 30 new amino acids before the stop codon
- In 43 genes the frame-shift mutation introduced from 31 to 45 new amino acids before the stop codon
- In 42 genes the frame-shift mutation introduced > 45 new amino acids before the stop codon

Legend:
X= amino acids between an adenine inserted in the coding region at position 100 and the first stop codon emerging downstream this mutation.
***= stop codon**

```
>NC_000913.3_cds_NP_414542.1_1_1 [gene=thrL] [protein=thr operon leader peptide]
[protein_id=NP_414542.1] [location=190..255]
MKRISTTITTTITITTGNGAG*
>NC_000913.3_cds_NP_414543.1_2_1 [gene=thrA] [protein=Bifunctional aspartokinase/homoserine
dehydrogenase 1] [protein_id=NP_414543.1] [location=337..2799]
MRVLKFGGTSVANAERFLRVADILESNARQGQV-SHRPLCPRQNHQPPGGDD*KNH*RPGCFTQYQRCRTYFCRTFDGTRRRPAGVPAGAIEN
FRRSGICPNKTCPAWH*FVGAVPG*HQRCADLPWRENVDRHYGRRIRSARSQRYCYRSGRKTAGSGALPRIYRRYC*VHPPYCGKPHSG*SHG
ADGRFHRR**KRRTGGAWTQRFRLLCCGAGCLFTRRLLRDLDGR*RGLYLRPASGARCEVVEVDVLPGSDGAFLLRR*SSSPPHHYPHRPVPD
PLPD*KYRKSSSTRYAHWCQP**RRITGQGHFQSE*HGNVQRFWSGDERDGRHGGARLCSDVTRPYFRGADYAIIFRIQHQFLRSTKRLCAS*
TGNAGRVLPGTERRLTGAAGSDGTAGHYLGGR*WYAHLAWDLGEILCRTGPRQYQHCRHCSGIF*TLNLCRGK*R*CDHWRARYSSDAVQYRS
GYRSVCDWRRWRWRCAAGATEASAKLAEE*TYRLTCLRCCQLEGSAHQCTWP*SGKLAGRTGASQRAV*SRALNSPRERISSAEPGHC*LHFQ
PGSGGSICRLPARRFPRCHAEQKGQHLVDGLLPSVALCGGKIAA*IPL*HQRWGWITGY*EPAKSAQCR**IDEVLRHSFWFAFLYLRQVRRR
HEFLRGDHAGAGNGLYRTGPAR*SFWYGCGA*TIDSRS*NGT*TGAGGY*N*TCAARRV*RRG*CCRFYGESVTTRRSLCRARGEGP**RKSF
ALCWQY**RWRLPREDCRSGW**SAVQSEKWRKRPGLL*PLLSAAAVGTARIWCGQ*RYSCRCLC*SATYPLMEVRSL
>NC_000913.3_cds_NP_414544.1_3_1 [gene=thrB] [protein=homoserine kinase]
[protein_id=NP_414544.1] [location=2801..3733]
MVKVYAPASSANMSVGFDVLGAAVTPVDGALLG-RCSHG*GGRDIQSQQPRTLCR*AAVRTTGKYRLSVLGAFLPGTG*ANSSGDDPGKEYAD
RFGLRLQCLFGGRGADGDE*TLRQAA**HSFAGFDGRAGRPYLRQHSLRQRGTVFSRWYAVDDRRKRHHQPASARV**VAVGAGVSGD*SLDG
RSQGYFTGAVSPPGLHCARATSGRLHSRLLFPSA*ACREADERCYR*TLP*TVTARLPAGAAGGRGNRRGSERYLRLRPDLVRSV*QAGNRPA
RCRLVG*ELPAKSGRFCSYLPAGYGGRTSTGKL
>NC_000913.3_cds_NP_414545.1_4_1 [gene=thrC] [protein=L-threonine synthase]
[protein_id=NP_414545.1] [location=3734..5020]
MKLYNLKDHNEQVSFAQAVTQGLGKNQGLFFPH-RPAGIQPD*N**DAEAGFCHPQCEDPLGVYW**NPTGNPGRARARGVCLPGSGRQC*KR
CRLSGIVPRANAGI*RFRRSLYGTNADPYCG**ASDHSDRDLR*YRSGSGSCFLRFTECESGYPLSTRQNQSTARKTVLYIGRQYRNCCHRRR
FRCLSGAGEAGV***RTESGARVKLG*LD*HQPFAGADLLLL*SCCAAAAGDAQPAGCLGAKRKLRRFDGGSAGEVTRSAGETFYCCDQRERY
RATFPARRSVVTQSDSGDVIQRDGRESAEQLAACGRVVPPQNLATERAGLCSRG**NHATDNA*VKRTGLHFGAARCRSLSCAA*SVESRRIW
LVPRHRASGEI*RERGSDSR*NVGSAKRAGRTC*FTLAFT*SARRFCCVA*IDDESSV
>NC_000913.3_cds_NP_414546.1_5_1 [gene=yaaX] [protein=DUF2502 family putative periplasmic
protein] [protein_id=NP_414546.1] [location=5234..5530]
VKKMQSIVLALSLVLVAPMAAQAAEITLVPSVK-ITDRRS**SWLLLGWRSLARPRLVETTL*MARQSLAPTRTAATAAPP*ESSS*SSRRSW
SRQTSPL
>NC_000913.3_cds_NP_414547.1_6_1 [gene=yaaA] [protein=peroxide resistance protein, lowers
intracellular iron] [protein_id=NP_414547.1] [location=complement(5683..6459)]
MLILISPAKTLDYQSPLTTTRYTLPELLDNSQQ-IDP*GAETDASAD*HADAHQRQTGGYQRRSLS*LAARFHAGECPPGDSGV*R*CLHRLA
GRNLQRRRFRFCPTAFANAFRLVWRTPPARFNAALSSGNGDPS*ECPRERSVSILGRYHHQQAERGARSTRR*CGD*SGVR*VF*IGEAEEIE
CRDYQAGVPR*EERQI*DHQLLR*ESTRSDESFHY*KSADQTRATDWF**RRLLL**RFLQQWRTGV*TLRAAL
>NC_000913.3_cds_NP_414548.1_7_1 [gene=yaaJ] [protein=putative transporter]
[protein_id=NP_414548.1] [location=complement(6529..7959)]
MPDFFSFINSVLWGSVMIYLLFGAGCWFTFRTG-ICAVSLHPPVWQKS*K*HSSTARRFNLISVIVYQSCGARG*RQPGRRCAGYYRRWTWSR
LLDVGCRVYRHGDLVCRMFSGTTL*RT*RQWAVSWRTGMVYGARAGDALDGRSVRRLFAHRLWHNFQRSSSERRCPRPEFFF*FSPAGDRHYS
RCLYSAGNHSRSSWRRPAHAGLCPVDGDNLGTDQPGNLRNEYRATSPRHLVYF*ECFWLAGSGRRRGGIYLKPGDY*RFSAQYVFQ*GGNGFD
AKRGSGSGVLASASGSARDCPDDWHFYRHPGHLYGKRHADITGG*RHNLHAAGRYSAYPEGDAGANGFLGC*VCYPCGYSVCLQLHRCQLHLC
RKQSLLFTPEQP*SDLVFADLHLRNGHRRHLAKPSADVATGRYHNGLHGYYQFDRHFTALACGSYHCQ*LSTPA*TRRAPGV*SIALSGYRSP
AFSGRVG*CFAGV
>NC_000913.3_cds_NP_414549.1_8_1 [gene=talB] [protein=transaldolase B]
[protein_id=NP_414549.1] [location=8238..9191]
MTDKLTSLRQYTTVVADTGDIAAMKLYQPQDAT-NQPFSHS*RSADSGIP*VD**CCRLGETAEQRSRAADRGRDRQTGSKYWSGNPETGSGP
YLN*S*CASFL*HRSVNCESKTPDQTLQRCWY*QRSYSDQTGFYLAGYPCCRTAGKRRHQL*PDPAVLLRSGSCLCGSGRVPDLAVCWPYS*L
VQSEYR*ERVRSGRRSGRGFCI*NLPVLQRARL*NRGYGRKLP*HRRNSGTGRLRPSDHRTGTAERAGGERRGYRT*TVLHRRSESASGAYH*
VRVPVAAQPGSNGSR*TGGRYP*VCY*PGKTGKNDRRSAV
>NC_000913.3_cds_NP_414550.1_9_1 [gene=mog] [protein=molybdochelatase incorporating
molybdenum into molybdopterin] [protein_id=NP_414550.1] [location=9306..9893]
MNTLRIGLVSISDRASSGVYQDKGIPALEEWLT-IGANHAV*TGNPLNPR*AGDHRANVV*AGG*NELPSGAHHGRNWPGAS*RNARCDAGSS
GPRDAWLW*TDAPDQPAFCTNCDPFASGGRDSQTGADP*LTRSAEVY*RDAGRCEGR*G*RCGTRYFCQRTVLHSVAGRAIR*NGTGSGCSIQ
TEECKTRR*RI
>NC_000913.3_cds_NP_414551.1_10_1 [gene=satP] [protein=succinate-acetate transporter]
[protein_id=NP_414551.1] [location=complement(9928..10494)]
```

MGNTKLANPAPLGLMGFGMTTILLNLHNVGYFA-TGRYYSCHGHFLRRHRANFCWSAGVQKRQHFRFNRIHLLRFFLADAGCDSADAETGSDR
CAKCTVPWCLPGSVGRIYAVYVLRHAERRTRSAIRFL*PDRAVCPAGDR*HCR*RRNHPLCRLDWADLRCQRNLSGDG*STERAVWSHRSADW
*IPL
>NC_000913.3_cds_NP_414552.1_11_1 [gene=yaaW] [protein=UPF0174 family protein]
[protein_id=NP_414552.1] [location=complement(10643..11356)]
MNVNYLNDSDLDFLQHCSEEQLANFARLLTHNE-KRQNSPLQRTDAQRTV*IDGRASRATSPQLAADCRRITAFWWR*YRQQTARTR*IVSGH
FARCFKAIEAESRQRDVYV*N*ATVTGTISA*YLEENGRGT*AGVSARGRCQGE*AGRAAAAADERQIIGKRCVAFAFQPTDPHFTHPRSNER
TWAWFAARRGAGRPCRCGTKWG*SGQRQRLSRDDSSRTANRLPAPDG*RHSGL
>NC_000913.3_cds_NP_414554.1_12_1 [gene=yaaI] [protein=UPF0412 family protein]
[protein_id=NP_414554.1] [location=complement(11382..11786)]
MKSVFTISASLAISLMLCCTAQANDHKLLGAIA-NAA*RNQRSGAETSCLSHCETHTTLCRPWRFTVKRCIGLFQSRP*RQSEPEYSFRNKRR
ANH*LDQH*QR*RQ*TLRLKNHLFGSYGELIGYGHAENYRRRL
>NC_000913.3_cds_NP_414555.1_13_1 [gene=dnaK] [protein=chaperone Hsp70, with co-chaperone
DnaJ] [protein_id=NP_414555.1] [location=12163..14079]
MGKIIGIDLGTTNSCVAIMDGTTPRVLENAEGD-THHAFYHCLYPGW*NSSWSAG*TSGSDEPAKHSVCD*TPDWSPLPGRRSTA*CFHHAVQ
NYCC**RRRMGRS*RPENGTAADFC*SAEKNEENR*RLPG*TGN*SCYHRTGIL*RCSASGNQRRRPYRWSGSKTYHQRTDRSCAGLRSGQRH
WQPYYRGL*PGWWYFRYFYYRNRRS*RRKNLRSSGNQR*YPPGG*RLRQPSDQLSG*RIQERSGH*PAQRSAGNAAPERSGRKSENRTVFRSA
DRR*PAIHHCRRDRSETHEHQSDSCETGKPG*RSGKPFH*AAESCTAGRWPVRI*YRRRYPRWWSDSYANGSEESC*VLW*RAA*RR*PGRSC
SNRCCCSGWCSDW*RKRRTAAGRYPAVSGYRNHGRCDDDADREKHHYPDQAQPGVLYR*RQPVCGNHPCAAG*T*TCG**QISGSVQPRWYQP
GTARHAADRSYLRYRC*RYPARFRER*KQR*RAEDHHQGFFWSERR*NPENGTRRRS*RRS*P*V*RAGTDSQPGRPSAAQHP*AG*RSRRQT
AG*RQNCYRVCADCTGNCSER*RQSRYRSENAGTGTGFPETDGNRPAATCPAADCRC*CFCKQRER*RCCRR*I*RSQRQKI
>NC_000913.3_cds_NP_414556.1_14_1 [gene=dnaJ] [protein=chaperone Hsp40, DnaK co-chaperone]
[protein_id=NP_414556.1] [location=14168..15298]
MAKQDYYEILGVSKTAEEREIRKAYKRLAMKYH-TGP*PG*QRGRGEI*RDQGSL*SSDRLAKTCGIRSVWSCCV*ARWHGRRRFWRRRRLQR
YFW*RFRRYFWRRTWSSTCGARC*FTL*HGAHPRRSCTWRDQRDPHSDSGRV*RLPR*RCKTRYTAADLSDLSWFWSGADAPGILRCTADLST
LSGPRYADQRSVQQMSWSWSC*AQQNAVR*NPGRGGHWRPHPSCGRR*SGRAWRTGRRSVRSGSG*TAPDFRA*RQQPVLRSPDQLRYGGAGW
RNRSTDP*WSRQTESAWRNPDR*AIPYAR*RRQVCPRWRTG*FAVPRCRRNTGRPERKAETAAARAARKLRWPNRRAQQPALKELL*WCEEVF
*RPDPL
>NC_000913.3_cds_NP_414557.1_15_1 [gene=insL1] [protein=IS186 transposase]
[protein_id=NP_414557.1] [location=15445..16557]
MNYSHDNWSAILAHIGKPEELDTSARNAGALTR-TPRNS*CCNSATSGAGLRPRGDVIT*SHCMGSAP*RCNII*RGSPEAAAECRRLVWHTC
RTNTCCTRRSYGLYKRKEIASCRWNSNQCARGRQR*MATTYGI*SSYLSVH*F*ANRQQRR*TAGPICANGRRDTHC*PGIRFASRMYPLTCF
WRS*LYRPGSLARIALVNCRRNAL*HDGFSARAGLR*ER*NHCNDRQFR**KSRSSLSGTSHCRITSSRKSINQ*NPTAQRESSKRTSSSGGN
AGSSGPCAIANIITGR*IFSRASG*LLPSAMAN*TGF*AAQKFAAPGCFACKGT*TRESVDIC*STRRIFN*RHNPAIAGFPPQKCRIRKEEL
>NC_000913.3_cds_NP_414559.1_16_1 [gene=mokC] [protein=regulatory protein for HokC, overlaps
CDS of hokC] [protein_id=NP_414559.1] [location=complement(16751..16960)]
MLNTCRVPLTDRKVKEKRAMKQHKAMIVALIVI-MYHRRSGGAGNEKRPL*GSHPNWPDGGCCFHGLRIRV
>NC_000913.3_cds_YP_025292.1_17_1 [gene=hokC] [protein=toxic membrane protein, small]
[protein_id=YP_025292.1] [location=complement(16751..16903)]
MKQHKAMIVALIVICITAVVAALVTRKDLCEVH-NPNWPDGGCCFHGLRIRV
>NC_000913.3_cds_NP_414560.1_18_1 [gene=nhaA] [protein=sodium-proton antiporter]
[protein_id=NP_414560.1] [location=17489..18655]
VKHLHRFFSSDASGGIILIIAAILAMIMANSGA-NQWMVSRLSGDAGSAPGWFTRNQQKHAVMDK*RADGGIFPVSRSGS*T*TDARIASQLT
PGRISSYRRYWWDDCAGITLSGF*LCRSDYPRRVGDPGGY*HCFCTWCTGAVGKSCSVSAEDLFDGSGYYRRSWGHHYHRIVLH**LIDGLSW
RRGCSNCGTRGIESVWCTPHGRLYSCWRGVVDCGVEIGGSRNSGGGNCRLLYSFEREAWAFSSEATGACVAPVGGVSDFAAVCIC*CWRFTAR
RHAGWLDLHSAIGDHRWLADWQTAGD*SVLLVGAAFETGASA*GNDLSANYGGGDPVRYRFYYVYLYCQPGLW*RRSRTD*LGETRYPGRFYL
FGGNWIQLVTRSFASISL
>NC_000913.3_cds_NP_414561.1_19_1 [gene=nhaR] [protein=transcriptional activator of nhaA]
[protein_id=NP_414561.1] [location=18715..19620]
MSMSHINYNHLYYFWHVYKEGSVVGAAEALYLT-TTNHYRTDSSAGRAPARQII*TQGTWSRTQRAGRTGLSLCR*NVHLKPGNAGYCELSQR
IQFIV*RWRG*CTFQTPGQ*RT*RRSGRRRAHSSSLLRIHPRNAAGAIKSA*TGYDHF*LSDRLYAAGRPVLRENWRMWREFLVYKSTTRKTV
PGLSGRTATFDSWATFNVRAQIA*LV*LPGIKRRNPRRV**CRFDESFWCDAQCNLRCPNALCI*LLCR*NCRRNWSRRECDGRVPCYFC*AD
DSAPGGTANLQYGLFCAF*SSGAL
>NC_000913.3_cds_NP_414562.1_20_1 [gene=insB1] [protein=IS1 transposase B]
[protein_id=NP_414562.1] [location=complement(19811..20314)]
MPGNSPHYGRWPQHDFTSLKKLRPQSVTSRIQP-RQ*RHRLRGNGRTVGLCRG*IAPALAVLRV*QSPEDGCCARIR*THYGDAGASYEPAVT
L*RGDMDDGWLAAV*IPPEGKAARNQQAIYAAN*AA*PESEAAPGTAGTEVAVVLKIGGAA*QSHRALSEHKTLSI
>NC_000913.3_cds_NP_414563.1_21_1 [gene=insA] [protein=IS1 repressor TnpA]
[protein_id=NP_414563.1] [location=complement(20233..20508)]
VASVSISCPSCSATDGVVRNGKSTAGHQRYLCS-TLP*NMATAVHLHRFSTRYAPENH*YGHEWRWMPGNSPHYGRWPQHDFTSLKKLRPQSV
>NC_000913.3_cds_NP_414564.1_22_1 [gene=rpsT] [protein=30S ribosomal subunit protein S20]
[protein_id=NP_414564.1] [location=complement(20815..21078)]
LANIKSAKKRAIQSEKARKHNASRRSMMRTFIK-KSIRSYRSWRQSCCTESI*RNATDRGPSGC*RSDPQKQSCTS*G*PDCTDQQTGL
>NC_000913.3_cds_NP_414565.1_23_1 [gene=yaaY] [protein=uncharacterized protein]
[protein_id=NP_414565.1] [location=21181..21399]
MCRHSLRSDGAGFYQLAGCEYSFSAIKIAAGGQ-ISSRDLRHGNEKPLLSDFGTQSPVNLDRCTRYTRTIFTVL
>NC_000913.3_cds_NP_414566.1_24_1 [gene=ribF] [protein=bifunctional riboflavin kinase/FAD
synthetase] [protein_id=NP_414566.1] [location=21407..22348]

```
MKLIRGIHNLSQAPQEGCVLTIGNFDGVHRGHR-SAVTGLAGRRAQAQLTGDGDAF*TSTTGTVCYR*SPGKTDPAAGKTALPCRVWR*LRAV
RAFRQAFRGVNRAKFHQRSSGEAFARKISCRR**FPLWRWS*RRFLVITESWHGIRLRYHQYANFLRRWRAHQQHRRASGPCG*QSGSGREFT
GAPVCHLRACSPR**IRAHYRFPDGECTAAPSGFPGERGLCGRSAGPR*KAVTRRGKHRNTPNGCRYSPAAGSAFVRCCNGPLRSPYTSSAA*
KNTQ*AAICVAGRTESADCA**INRPRIFWANKTGL
>NC_000913.3_cds_NP_414567.1_25_1 [gene=ileS] [protein=isoleucyl-tRNA synthetase]
[protein_id=NP_414567.1] [location=22391..25207]
MSDYKSTLNLPETGFPMRGDLAKREPGMLARWT-R**SVRHHPCG*KRQKNLHSA*WPSLCEWQHSYWSLG*QDSERHYREVQRAFRL*LAVC
AWLGLPRSADRAESRARIR*AG*EIHRRRVPRQVPRIRGDPG*RSTQRLYPSGRAGRLVAPVPDHGLQN*SQHHPRAGQNHRQRSPAQRREAS
SLVR*LPFCAGGSGS*VLRQNFSVHRRCFPGSRSGCTESKICRKQR*RPNLAGNLDHHAVDSACQPRNLYCTRFRLCAGADRRSGRDSGERSG
*KRNAAYRRDRLHHSRHGKRCGA*AAALYPSVYGLRRSGNPRRSRYPGCRYRCRSHRAWPRPGRLCDRSEIRPGNR*PGWPGRHLSAGHLSDA
GWRERLQSERHRRCAAAGKRRAAAR*ENAAQLSVLLASQNADHLPRDAAVVRQHGSERSACAVTERDQRRAVDPGLGPGAYRVDGC*PS*LVY
LPSAHLGCTDVTVRAQRHGRAASAYP*TDGRSGKTR*SRWHPGVVGSRCERDPRRRS*SVRESAGHIGCMV*LRIYPLFCC*RASGICRSRSG
HVSGRF*PTPRLVHVFPNDLHRDEG*SAVSSGTDPRLYRGWSGPQDV*IHRQYRFAAGCDEQTGRGYSASVGGINRLHR*NGRF*RDPETCCR
*LSSYP*HRALPAGKPERF*SSKRYGETGRDGGTGSLGRRLCESGTGRHPQGVRSIRFPRSGTASDALLLR*DGFLLPRHHQRPSVHRQSGQC
GAS*LPDCAISHRRSAGALDGTNPLLHR**SVGLPAGRT*KIRLHR*VVRRPVWPGRQ*SDERCVLGRAVESAWRSEQSH*ASACRQESGWLA
GSGSNLVCRTGTVGETDRAGR*ITICPVDLRRYRCRL*RRTC*CSAERSTQRAESRVE*SRR*EVPTLLALHPGCRQGGGTRRNLRPLCQQRR
R*R*KT*VCL
>NC_000913.3_cds_NP_414568.1_26_1 [gene=lspA] [protein=prolipoprotein signal peptidase
(signal peptidase II)] [protein_id=NP_414568.1] [location=25207..25701]
MSQSICSTGLRWLWLVVVVLIIDLGSKYLILQN-ICSGGYGPAVPVA*SALCA*LWRGV*FPCR*RRLAALVLCRYCDWY*RDPGSDDVSLEG
HAEAKQYRLRADYWRRAGQPVRPPVARLRCRYDRLLRRRLALRHLQPCRYCHLCRCGTDCAGRFFAF*SEKTI
>NC_000913.3_cds_NP_414569.1_27_1 [gene=fkpB] [protein=FKBP-type peptidyl-prolyl cis-trans
isomerase (rotamase)] [protein_id=NP_414569.1] [location=25826..26275]
MSESVQSNSAVLVHFTLKLDDGTTAESTRNNGK-TGAVPPG*CFSF*RAGATPVGAESGR*NHLLVGARCGVWRAVTGPDSVLLPP*IYGCRR
ARNWRNHAFYRNGWQ*DAWRDPRN*RRLHYR*FQPSAGRADRSF*Y*SAGNRSGTGGV
>NC_000913.3_cds_NP_414570.1_28_1 [gene=ispH] [protein=4-hydroxy-3-methylbut-2-enyl
diphosphate reductase, 4Fe-4S protein] [protein_id=NP_414570.1] [location=26277..27227]
MQILLANPRGFCAGVDRAISIVENALAIYGAPI-ICPSRSGT*PLCGR*LA*AWGYLY*AD*RSTGRRDPDFLRTRCFSGGT*RSKKSRFDGV
*CHLSAGDQSAYGSRPRQSPWRRIYSHRSRRAPGSGRDNGPVQ*PGRGNVSGRIAGRCVETDGQKRREALLYDPDHAVGG*HV*CDRRAA*TL
PENCRSAQR*HLLRHD*PSGSGTRPGRTGGSCVGGRFEKLLQLQPSGGAGPAYGQTRVFD*RCERHPGRVGERG*MRRRDCGRIGSGYSGAEC
GGTFAAAGRW*SHSAGRP*RKHCFRSAERAACRYS*SRL
>NC_000913.3_cds_NP_414571.1_29_1 [gene=rihC] [protein=ribonucleoside hydrolase 3]
[protein_id=NP_414571.1] [location=27293..28207]
MRLPIFLDTDPGIDDAVAIAAAIFAPELDLQLM-NHRRG*CLG*ENYPQCPATAAFLECGDSARPRGRCATGTRTA*CGICARRIGNGWLRLC
*AQPKAARDTGVSGDSGCPDACTRACYPGGHRPVNQYCAVTFTMPGMQAVYSPSGDHGWFCRTRQLYAKRRV*YCCRSRSCCLCLPQWY*NRH
VRFGCHQSGNINS*LSLYTAAVKPYRENASRPV*PLP*RQYAKRLANARSLRHRLAGAPGPVHSQTLFCGSGNSGRIYLRHDGG*YRRLPGQA
SQCTGGIGSGCERLPAVGG*GAGSGVV
>NC_000913.3_cds_NP_414572.1_30_1 [gene=dapB] [protein=dihydrodipicolinate reductase]
[protein_id=NP_414572.1] [location=28374..29195]
MHDANIRVAIAGAGGRMGRQLIQAALALEGVQL-RRCAGA*RIFFTGQRRR*AGRSRENRRYRAKQPRCGKR*F*CVYRFYPSGRYAEPSRFL
SPAWQRDGDRHYGV*RSR*TSNS*RRCRYCDCLCCQF*RWR*RHA*AAGESSQSDG*LHRYRNY*STS*T*S*CAVRHRTGNGRGDRPRP**R
SERLRGLQS*RPHR*TCAWHHWFCHRACR*HRW*TYRDVCRYWRASGDHP*GVQPYDIC*RRGKIGFVVEW*GKRSF*YARCT*SQ*FV
>NC_000913.3_cds_NP_414573.1_31_1 [gene=carA] [protein=carbamoyl phosphate synthetase small
subunit, glutamine amidotransferase] [protein_id=NP_414573.1] [location=29651..30799]
LIKSALLVLEDGTQFHGRAIGATGSAVGEVVFN-NFNDRLSRNPH*SFLFSSNRYSYLSPYWQCRHQ*RR*RIFSGTCTRSGDSRPAADCQQL
P*YRRPLFLPETP*HRGDCRYRYP*ADAFTARERRTEWLHYRGR*PGCGAGVRKSPRVPRSEWHGSGKRSDHRRSL*LDTRELDVDRWPARSE
KRRRAAVPRRGL*FWCQAQHPADAGG*RLSPDHRSGANFCGRCAENESRRHLPLQRSWRPGPVRLRHYRHPEIPRNRYSGIRHLSRSSAAGAG
ERCEDCQNEIWSPRRQPSG*RCGEKRGNDHRPEPRFCGGRSNITCKPACHA*IPVRRYVTGHSSHR*TGIQLPGAP*SQPWSTRRRAVVRPLY
RVN*AVP*NR*V
>NC_000913.3_cds_NP_414574.1_32_1 [gene=carB] [protein=carbamoyl-phosphate synthase large
subunit] [protein_id=NP_414574.1] [location=30817..34038]
MPKRTDIKSILILGAGPIVIGQACEFDYSGAQA-M*SPA*RGLPRHSGELQPGDHHDRPGNG*CNLHRADSLGSCTQDY*KRAPGRGAANDGR
SDGAELRAGAGTSGRVGRVRCHHDWCHCRCD**SRRPPSFRRSDEENWSGNRAFRYRTHDGRSAGGCR*RGLPVHYSPILYHGR*RRRYRL*P
*RV*RNLRPRSGSLSDQRVAD**VADRLERVRDGSGA**KRQLHHRLLYRKLRCDGHPHR*LHHCRASPNADRQRISNHA*RLDGGAA*NRR*
NRWFQRSVCGEPEKRSSDCYRNEPTRVPFFGAGVESDRFPDC*SGGETGGGLHPRRTDERHHWRTYSGLLRAVHRLCGY*NSSLQLRKIRRC*
RPSDHSDEIGWRSDGDWSHAAGIPAKSAARPGSRCDWIRPESEPG*PGSVNQNPSRTERRRRRSYLVHRRCVPCGPVCGRRLQPDQH*PLVPG
TD*RAGASGRESGGSGHHWPER*LPAPAETQRLCRCALGKTGGRTRSGNP*AA*PV*PAPGL*ARGYLCGRVRHRHRLHVLHL*RRVRSESVY
RP*KNHGAWRRPEPYRSGYRIRLLLRTRLAGAARRRLRNHYG*L*PGNRLHRLRHFRPPLLRAGNSGRCAGNRAYREAERRYRPVRRSDPAET
GARAGSCWRTGYRHQPGCYRPCRRP*TLPACG*ASETETTGERHRYRY*NGGREGERDWLPAGGTSVLRSRRSGDGNRL*RS*PASLLPDGGQ
RV*RCASVAGPLPR*RGRS*RGCHLRRRNGADWRHHGAY*AGGRALR*LRMFSASLHLKSGNSGCDAPAGAETGLRIAGARPDERAVCGEKQR
SLPD*S*PACGAYRSVRLQSHRRTAGKSGGARDGWQIAG*AGRNQRSYPAVLLGERSGAAVQ*IPGR*PAVRARNALYRGSHGRGPHLR*SVC
QSAAGQQLHHEETRSCAAFRARRR*RTRGGPGGKTAETGLRAGCDPRHGDCAGRSRYQPASGKQGA*RPSAHSGPYQEWRIYLHHQHHLRPSC
D*RLPRDSSQCAAI*SALRHHPERRLCHRDGAECRCD*KSNFGAGNARTDQI
>NC_000913.3_cds_NP_414576.4_33_1 [gene=caiF] [protein=cai operon transcriptional activator]
[protein_id=NP_414576.4] [location=34300..34695]
MCEGYVEKPLYLLIAEWMMAENRWVIAREISIH-IRY*TQQGG*YPDLYSVGSHRNKLRS*DDP**AGRAGMPVSATG*SGRYR*ANLRAPAQ
*QSGKISRCKKDAAYSCRSAHGT*PRAEVADDVVKEYASL
```

>NC_000913.3_cds_NP_414577.2_34_1 [gene=caiE] [protein=stimulator of CaiD and CaiB enzyme activities] [protein_id=NP_414577.2] [location=complement(34781..35371)]
VSYYAFEGLIPVVHPTAFVHPSAVLIGDVIVGA-RCLHRPTRLTAW*LRAVDRASGSQYSGWLHYAWLLRH*HYRWGKRPYRARSDPAWLFDW
SRCIGRDEQRDYGWRGHWRREHCCRHELCQSGLSRRETPVVDGYASPRCTQC**RRVTLETVEYQRVSGSCRALSCIVT*NAATEANGGKSPP
FAGDDGCDA*TV
>NC_000913.3_cds_NP_414578.2_35_1 [gene=caiD] [protein=carnitinyl-CoA dehydratase] [protein_id=NP_414578.2] [location=complement(35377..36162)]
MSESLHLTRNGSILEITLDRPKANAIDAKTSFE-NGRSISKFP*RSAITCRHYYRCRREVLFRGLGFKSGSRRRSTGC*LWSGWFCGINRNFQ
SRQTGYRSCERLCLWRRL*TGAGGRFYCLCR*RQLRPAGSQTGHRS*QRRCAASAEDPAACHRQ*NGDDRQTNGRRRGAALGDSQPRG*PGGT
DG*RPRTGSAAG*QRPAGDCGAERDLPHHQRNAGRRSVSLYSQRRVETLSIGSAFGRCH*RAAGVCREARSGVERTL
>NC_000913.3_cds_NP_414579.4_36_1 [gene=caiC] [protein=putative crotonobetaine/carnitine-CoA ligase] [protein_id=NP_414579.4] [location=complement(36271..37824)]
MDIIGGQHLRQMWDDLADVYGHKTALICESSGG-SR*PV*LS*VKSGD*PHGKPVLYAGDSQRRQGCTTSRQLPGIYLLLVRAGKNWRDYGAD
*RPPVVRGKRVDPAK*PGVPAGDQCAILSYVSTDSAGRCHSIAAHLPDRCGTSR**WREFVYSTEKSTTCHLVLCTAAID*RYGGNSLHLRHH
LPTERCGDYPLQPALRWILLRLAVCTA*R*RLPDGNACVSYRLPVYCGDGGVFCRGHLCAGREIQRPRLLGTGTEVPRHRYRMYSDDDPYVDG
TAAFSERSATPPAGSDVLSQLVGAGKRCVL*TLRRSLADVLWDDGNHCGHYRRSSWR*TTLAVDWSGGVLLRSGDPRRSQSPAPGW*DR*NLH
*RHTWENHLQRVLSQPTSHCESAGSRWLAAYRRYRIPRRRGLFLFRRSPLQYD*TWRRECLLRGAGKYYRRAPENSGHRGCGY*RFDSR*SHQ
SICGAE*R*NIERRGIFPLLRTKYGEI*SALLSGDQKRSAT*LLGENN*KESEI
>NC_000913.3_cds_NP_414580.1_37_1 [gene=caiB] [protein=crotonobetainyl CoA:carnitine CoA transferase] [protein_id=NP_414580.1] [location=complement(37898..39115)]
MDHLPMPKFGPLAGLRVVFSGIEIAGPFAGQMF-SRMGRGSYLDRERRLGRHHSRSTELPATLPPQFARAVVKYFQR*RPRSVSEINGNHRYL
HRSQ*RSGLCPSWHYR*STVAAQPETGYRSPVRFWSVRHRGVHQSSGL*HYRPGL*WLPDSER*C*PANACLPVYRRLLFWPDRHHGGAGSTA
*SA*NR*RRKYRHRHV*SDAAYGPVLHDGLLQRRRNVPAHEQR*RSLLRRLRSV*MCRRLHRDGTGGHYPN*RVL*RYWPRTSAWHARNPGRH
SAYPPYRMPLRPTG*RETRCLAGDTYHRGSKRTLC*TEYRLRQSADRTGTGKQSTVCGSRINHSVANDGWSHLQRAEHHAEIQK*PRTNLARN
ALTWHGHGCHFEKYRLQRKRHSGVGQQRSGQS*GL
>NC_000913.3_cds_NP_414581.1_38_1 [gene=caiA] [protein=crotonobetaine reductase subunit II, FAD-binding] [protein_id=NP_414581.1] [location=complement(39244..40386)]
MDFNLNDEQELFVAGIRELMASENWEAYFAECD-T*QRLPGTFCQSTGGYGYRQSADP*RARWSGRGVCYSRRRVDGAGTSGGTNLCAVPVAG
RVQHLPARRHTRADRQNYGFPRHR*ADVELSDYRTGRGLRRG*PENDLYP*KW*DLS*W**VFYYQQRLHPVHRGDGARRGFSGQTCLHRMVC
*YEQTGHQSDQT*KARSAYG*LL*NHL*RRGTGRERHVRSGR*RL*PRQRRVRP*TFPGSPHQLRYGDVRL*RCGALRQSARAVWRGYWSFPV
DSGKIRPHGDQIKLHEKHAV*SSVESRQRHHHLWRCSDVQILLRQCGI*SCG*RNAGAGRCRDCGQPPHQPLLA*SACRPRLRGI*RNADPDA
GSCGAEAIPL
>NC_000913.3_cds_NP_414582.1_39_1 [gene=caiT] [protein=putative transporter] [protein_id=NP_414582.1] [location=complement(40417..41931)]
MKNEKRKTGIEPKVFFPPLIIVGILCWLTVRDL-RCSECRY*CCIQLRHQCMGMGI*MVYGGDAFRLVLAGVWPVCQKAFR*RTARI*HRQLD
LYDVRLLYVCCRTVLGID*DLLLHLHPAVWLRTELDRGERVGAGLQLVPLGTSAVGHLQLPFSRLRLLLLCPQNGSDSPQLDTGAAGR*KTRQ
RVVRHYRRQLLSRRLDLRDGYQSGPCHAAGDRVYAMVVWHSAYPATGRYHHYLLDYPQRHLRRLRSAKRGTYRQ*RA*LPELPDAGLGVHCQR
CQLHHELLHRFGGDVADVSAAHVVLYRSHR*RRLPAGLDRVLLGMVGDLCYPDEYLPRPHLPWSYCA*TVLRHGAGADSVNLDPVDCTR**HS
AVDR*KHHQHSKSDRTVRCGARHH*NLGRSATQHRHHVGLLHPLLYCHRYAG*RLLLYPGDVHLPRSTRW*RTTSAGAYRLVNSGWHYRYCSA
GARRPETDSNRHYRRRMPAVLRQHYGDALLY*RRETELERL
>NC_000913.3_cds_NP_414583.2_40_1 [gene=fixA] [protein=anaerobic carnitine reduction putative electron transfer flavoprotein subunit] [protein_id=NP_414583.2] [location=42403..43173]
MKIITCYKCVPDEQDIAVNNADGSLDFSKADAK-NKPIRSQRY*SGLPAKATGSRGAGDSLKCGR*SPDQRQRA*RCAIARPG*TDCGD**PV
RAGTAATNGERTGCSRPESRL*SDPLWRWFFRPLCPAGWSAGGRNPQYSGS*RRQQNYLPDGRYPHR*ARTGR*NRNLKHSAACGCCCFH*YQ
LPTNSFDESHSRRGEKARPGMVGGGYWF*RRGSLVRTTGCRAETARTSAHRD*RRRRRTDRRIC*KSSQSHL
>NC_000913.3_cds_NP_414584.1_41_1 [gene=fixB] [protein=putative electron transfer flavoprotein, NAD/FAD-binding domain and ETFP adenine nucleotide-binding domain-like protein] [protein_id=NP_414584.1] [location=43188..44129]
MNTFSQVWVFSDTPSRLPELMNGAQALANQINT-ICPQ*CRRRTGNPARR*SCLEIKRQTGRSDDRRLRRCHG*HYSPARRRRPGAAAKHPSR
QITGGKTGLSP*SGGV*RCQHRQRTGR*SDSETHGLRWSGDWRRTHCHAVCGTDHQQRHVRCGSARRVTHWRNAHRGVAGSGCGDYPHGNPGA
PEQQRRSRQSPSGGQRRSRYWQQREHCAGRTALQGDRCGVGLFSSGGGKRKMDGARTLCRYLQPDAET*TVPGGGDLRADPAHGWR*RVANHF
RHQ*R*KCADLPVRGLRHCWRRREDPSGADRSFSAL
>NC_000913.3_cds_NP_414585.1_42_1 [gene=fixC] [protein=putative oxidoreductase] [protein_id=NP_414585.1] [location=44180..45466]
MSEDIFDAIIVGAGLAGSVAALVLAREGAQVLV-NRAWQFRRCQERHRRASLCPQSGTHYSWFRRLRPRRTPDHP*KTRVYDGKVSDDYGLLQ
W*RNLAIPAFLLRFAQ*I*CLADGAGRRSGRAVNYRDPRR*PRTARWQSRRCRSRWRCD*SENGDPC*WGELHPCRKIGDGKTRQTDGCGGWR
EGTDRVTEVGY*RPFSVAG*SGGGLPVCGITHRWPDGRRLPLYQ*KHPVAGAGLWFASSA*REKIGAANAGRFQTASGRCTADRGRQAGGIFR
SRSAGSRHQHAAGVGW*RRIDCR*CRRNVYEPRFYHSRYGSGDCRRGSRSKNRAFSDEKRRFQ*AKTGGISSAS*EWSAARYAYVPETTGVP*
*PTHV*RLPGAGGGCGA*PVHH*WQRAGTDAQENPPPRQESGLHQSNQGWHERSDRFM
>NC_000913.3_cds_NP_414586.1_43_1 [gene=fixX] [protein=putative 4Fe-4S ferredoxin-type protein] [protein_id=NP_414586.1] [location=45463..45750]
MTSPVNVDVKLGVNKFNVDEEHPHIVVKADADK-TGAGAAGESVPRRSVQEAG*RQCALRLRRMSGVRHLSHSGAGVGAGTVGIPARHLWCGV
PLRL
>NC_000913.3_cds_NP_414587.1_44_1 [gene=yaaU] [protein=putative MFS sugar transporter; membrane protein] [protein_id=NP_414587.1] [location=45807..47138]
MQPSRNFDDLKFSSIHRRILLWGSGGPFLDGYV-TGNDWRGAGATDAGAETGR*LDWLAGRGNARRAVRWHIAVWLYFR*SRTAQNVPH*YHR
HRRDIGGDDVCFIPRRTVGDAGTYRHCHRCRLSHRHLNDHRVLQYPSAGVFHQLYCRDVVCRRDLCRSGRLLAL*CGRRLALDAG*RGDPLFV
DFDWSIRTA*ISPLVITQRASKRVRRDDDQTVWRTGGFR*RAAAANPFSRSV*SPPFSFCSVCCRHLDLPGDPNVRHLHLWPANRWFVGIGGW

QKRGTRECGD*PVLYARLYSADAVVKHCRTASIVDWQLCHDDAGAGGFGANPGYGDLAGSDGLCGVCLFLWRAG*FAVALS**TLPDRYPRLC
RGRDYVLKSYWHHCFDLGTTDLYQ*LRYQ*HDANGGGYLAVWLVDFRSVCPGDSRDVTGADQQYDDPRAENGV
>NC_000913.3_cds_NP_414588.1_45_1 [gene=kefF] [protein=potassium-efflux system ancillary protein for KefC, glutathione-regulated; quinone oxidoreductase, FMN-dependent] [protein_id=NP_414588.1] [location=47246..47776]
MILIIYAHPYPHHSHANKRMLEQARTLEGVEIR-ISLSTLS*LQYRYCRRAGGAVSRRSDRLAASDAVVQHSSAPQTLDR*SFLPRLGLRSWR
HGAAWQTFAVGGDDRRRGKPF*NWCASGL*CAVAAATGDGNLLRAELAATVCHALHLYL*RRNPRRAGASL*ATSAGMAGGPSWI
>NC_000913.3_cds_NP_414589.1_46_1 [gene=kefC] [protein=potassium:proton antiporter] [protein_id=NP_414589.1] [location=47769..49631]
MDSHTLIQALIYLGSAALIVPIAVRLGLGSVLG-IPDRRLHYWPVGAATGDRCRIYSALCRDWGGADAVYYRPRTRSTKAVEAACGSVRLWRI
ADGDLRRPAGAVLHVTWAALAGRGIDRHDAGALLYGDCHAGDE*TQSDGDANGSQCLCGAAVPGYRGDPAGGDDSATGNEQCLDDDGRICSLG
VKSGGCAGAGGIAGALCHASGAAFCSPLWLAGSV*CRGVIPRVWLWFAAGRGRLVDGDGRVSGGRTAGKLGIPSCAGERYRTI*RFAVGAVFH
RCWHVDRLWHAA*KPIAHCHFAARFPHHQNRHAVADCPTVASAK*TASLVCGVVRAGQ*VCLCGIWRGADGECAGAGVGEIADPGGGAVDGSN
ADSAGDPQSP*AIFY*GSA*SR*DRRRTAARDYRRIRSFWADYRTFTALQRGENGGTRSRSGPYRNLA*IWYESVLWRCHADGFTGICRSGES
GSAD*RHRRSANQPATDRDGERTFPAFADYCPRPRCRPLHSFASGRR*KAGA*NLRRCAENRASGTGKFRSGAV*SARTCRCVPPL*YSDGGR
DGNG*ERHQSPRGGL*THQRDVK*DHYRGPRTSVINSTTWLAGNRRR*TYRQHGG*TGNETLIL
>NC_000913.3_cds_NP_414590.1_47_1 [gene=folA] [protein=dihydrofolate reductase] [protein_id=NP_414590.1] [location=49823..50302]
MISLIAALAVDRVIGMENAMPWNLPADLAWFKR-KHLK*TRDYGPPYLGINRSSVARTQKYYPQQSTGYGRSRNVGEVGG*SHRGVW*RTRNH
GDWRRSRL*TVLAKSAKTVSDAYRRRSGRRHPFPGLRAG*LGIGIQRIPRC*CAELSQLLL*DSGAAV
>NC_000913.3_cds_NP_414591.1_48_1 [gene=apaH] [protein=diadenosine tetraphosphatase] [protein_id=NP_414591.1] [location=complement(50380..51222)]
MATYLIGDVHGCYDELIALLHKVEFTPGKDTLW-TDGRSGRARAGFAGCSALCEILRRQRTSGAGQSRSASAGGICRDQPQ*TERSPDTAAGS
AGCRRAA*LAAAPASAANRRREKAGDGPRRDHAAVGSADRQRVRTRCRSGAIE*LLSLLS*CHVRRYAK*LVTGIAGAGKTAFYHQRFYPYAF
LLPERSTGYVQQRIAGRGPCPTETVVCDSWPCR*RIQHRLWSLGIAGGQRYAGRYIRAGYRLLLGWYINLPALGR*TVFCPAVEPA*GFGRSG
GVL
>NC_000913.3_cds_NP_414592.1_49_1 [gene=apaG] [protein=protein associated with Co2+ and Mg2+ efflux] [protein_id=NP_414592.1] [location=complement(51229..51606)]
MINSPRVCIQVQSVYIEAQSSPDNERYVFAYTV-NHTQSGASASAVVGALLADHQWQWP*NRSPGRRSGWRPATYRAWRRVPVHQRCNH*NPA
GHHAGSLRNDR*KWRPFQHRHSRIPTRRSHTHSL
>NC_000913.3_cds_NP_414593.1_50_1 [gene=rsmA] [protein=16S rRNA m(6)A1518, m(6)A1519 dimethyltransferase, SAM-dependent] [protein_id=NP_414593.1] [location=complement(51609..52430)]
MNNRVHQGHLARKRFGQNFLNDQFVIDSIVSAI-KPAKGPGDGRNRPRSGGIDRTGRRTSGPADGHRT*PRSGGTSANASILRPETDDLSAGC
DDL*LW*TGRENGSAAACFRQPAL*HLHAVDVPSV*LY*CHCRHALYVAKRGGESSGCRTEQQSVWSIKRHGAILLQCDPGTGSTAVSLYTTT
QSGFRRRAPGSSCNDASPG*RCSCVEPHHHRSL*PAS*NHS*QPRQPV*RRGVNGNGDRPGDASGKYLCRAILPDGELSGGERAFAGEL
>NC_000913.3_cds_NP_414594.1_51_1 [gene=pdxA] [protein=4-hydroxy-L-threonine phosphate dehydrogenase, NAD-dependent] [protein_id=NP_414594.1] [location=complement(52427..53416)]
MVKTQRVVITPGEPAGIGPDLVVQLAQREWPVE-TGCLCRCHSPYQPGSDARFAAHPPPLFPQLPCTTANCGHINATSCRATCTCHCGAVSG*
KWALCGGNAGASVRWLSERRICRADHRSGA*RRY*RRWHSFYRSYRVFRRAFAGEKGGDDAGDRRTSRGAGNDAFTAARYRRRYHPCTFARSD
CYFASRFADQIWYCRTAHSGLRAESARGRRRSYGYGRDRHHYSGAQ*AAGAGDETQRAAACRYPVSAEIS**RRRRAGDVPRSGSSRAKIPGL
RARCEHYAGPALYSHISGPRHRA*TGGTWQSRCRQFYYGA*SRHQNDC*HPM
>NC_000913.3_cds_NP_414595.1_52_1 [gene=surA] [protein=peptidyl-prolyl cis-trans isomerase (PPIase)] [protein_id=NP_414595.1] [location=complement(53416..54702)]
MKNWKTLLLGIAMIANTSFAAPQVVDKVAAVVN-KRRRAGKRR*WINAVGKTERCSGKAATS**RDAAPPNHGTFDHGSNHPADGAENGSENL
R*AAGSGDC*HRETEQHDAGSDAQPSGLRWTELQHLS*PDPQRDDYL*SA*QRGASSHHHPAAGSRIPGAAGG*PKRRQH*AEPEPHPDPAAG
KPDL*SGERSGKPGARHCRSGA*RR*FR*AGDCSFCRPAGAERRPDGLGPYSGVARDLRPGIKHREERRHCWPDSFRRWLPYSES*RPARRKQ
KYLGDRSSCSPYSAETVADHD*RTGPCETGTDCC*YQEW*NDFCCRSERVLSGSRLC*PGRRSRLGYTRYFRSGLP*RPDSPEQRSNECTGSL
FIRLAFNRTAGYP*CR*NRRCAERSCIPHADEP*VLGRSSKLDAGTTCQRLR*NPEQL
>NC_000913.3_cds_NP_414596.1_53_1 [gene=lptD] [protein=LPS assembly OM complex LptDE, beta-barrel component] [protein_id=NP_414596.1] [location=complement(54755..57109)]
MKKRIPTLLATMIATALYSQQGLAADLASQCML-RRAKL*PSSGTGRYQ*LTRDYQC*PRERGLPG*RRVYWQRGYHAG*QPSAGRRSAAPSK
RGTRTTGAGTYR*CAR*CPLRR*PGDPQRAERLGESEHQRYQRLGR*LPDGGSPGSR*SGPDETTWRKPLYHSG*R*LYLLSAGF*HLERGR*
RNYS*PRRTSCGDLERPL*GGSGTDLL*PLFAVAGG*QTSLWFLDPERQVHHHQLL*VLPAILLEHRAKYGCHHHAALYASSWQHHVGERIPL
PLPGGRWLDGTGLSAFR*SL*R*TPER*QFTSLVILLEPLRGHGSGVAFQRRLHQGQRS*LLQ*FR*QVRFQY*RLRNAKIQRWLCGAKLQCH
RFNQAVPGFQRTEHQ*LLGRAAVRR*LLPE*CWSV*YAYLRPGSALC*HQRRHA*SNPCSPGTDHQFAAL**LGQHQYRSEVAGNPLSANQS*
LV*LQKHDQAGRIR*PRNAAIQS*RQNGL*TRYGNAGSGLYPNAGTARAVFVRAVSRSERHL*LRLVSAAI*LLWPVPGPDLRRS*PYCLR*P
GDDRCHISHI**CCR*TF*YFRWSNLLFHGVSHWR*QHNMGE*RQNGFTGVGRRYLLAYLRALGIAWRDSVRYTSG*RSDQ*LQH*IPSG*RP
SGTAELPLRQPGIYSGYAA*VLFHC*AI*EWYFAGRCCRQLANCRSLVHCWGLLLRHQC*QASRLYVRCAIQLLLLCNSRRLRAEAERLG*R*
TTCGI*QRNRL*HRTSRPELQLRSGYARDAAFEHSAVSKHFV
>NC_000913.3_cds_NP_414597.1_54_1 [gene=djlA] [protein=membrane-anchored DnaK co-chaperone, DNA-binding protein] [protein_id=NP_414597.1] [location=57364..58179]
MQYWGKIIGVAVALLMGGGFWGVVLGLLIGHMF-R*SP*P*NGVVRQPA*ASGAVFCHHF*SDGAFNQIQRSRHGG*YSYRQPVDGPNESSWR
FPYCGAKCVPGGKIRQLPAARKDAPVSQCLLWSF*LNSYVSGDPDSGGVC*WFTAPE*TGGAVCHCRRIRDLPRSV*PVFAHDAGRCTVWRRL
SAANWRW*LAASAAWPNAGRCL*CAGREADG*CDHHQTCLP*ADE*TPSR*AGGERFAA*DDGDGEAESAGNSAGI*ADKAAERV*M
>NC_000913.3_cds_NP_414600.1_56_1 [gene=rluA] [protein=dual specificity 23S rRNA pseudouridine(746), tRNA pseudouridine(32) synthase, SAM-dependent] [protein_id=NP_414600.1] [location=complement(59687..60346)]

MGMENYNPPQEPWLVILYQDDHIMVVNKPSGLL-ISAGSSGRAQRQRDDAHSA*LSAGRIGASSGYGYQRRDCSGADQSRGAGVKTPVPRARA
EKAVCGPRLGASIPRRRSGGSAADLRLAKPPETESLLRNG*TCADGI*SGGVCGG*HGKSGVKTDYRAFASIACAYAGAGSSDSRRSFLCITR
SESDGTTFVATCRDVDDYPSGVWQ*YDV*SASGFL
>NC_000913.3_cds_NP_414601.1_57_1 [gene=rapA] [protein=RNA polymerase remodeling/recycling
factor ATPase; RNA polymerase-associated, ATP-dependent RNA translocase]
[protein_id=NP_414601.1] [location=complement(60358..63264)]
MPFTLGQRWISDTESELGLGTVVAVDARTVTLL-IPIYW*KPSVRTQ*FPRDPRDVQPW*YHYQP*RLADASRRSKRRKWLADLYRYSPGY*R
VRRSPA*SFP**QTGVQQTAGPSVCRAD*PYGPLCAALSRA*IFQRTVPYAVQRPARSAYQPDPASAQHRS*CWSPPRAARPAG*RSGFRENH
*SRDDPASATALWRC*TCANYRPGNLTASVAGRNAAPFQPALCAI***ALCRSSARCLQPV*HRTAGDLLAGFCPS*QTAPGTSL*SRMGPAG
GR*SASPGVERRCAKP*ISGH*TTGRARAGRSAADRDPGTAGDGKPLRPSASAGPEPFPRFCAVR*RAEKLSSGCGRRCHAAGR*QTEQ*RTE
HARRDDRRAGYRAAVAGSKQRQRRCPERPSGAGFDADGSPRHQPRAVP*HA*RCERIPETRAAHH*AAATDAVSDGY*SLRHYGRT*KCGRSC
SRYALPGAYLSGI*R**RHLVELRSAR*VADGLPDQPSLSESAGDLRQSCHCAATGAGTART*RYSRCGVPRRYVDYRT*PRCRLVCRRRHRR
TGTAVLRNRF*RT*LPVRQPHGDV*PAIQPGSTGAAYWSSGSYRPGARYSDPCALSGENRSVGAGALVSRRSGCI*AHLPDRTHYLR*RIQRS
D*LSGFTGSNRRL*RSDQKLPRAT*SAESTAGTGS*PPAGNPLQRWRKSPGTGRKH*RAG*RYQPDRLRHEPVRYYRYQSGRSRRQHDRADAV
RSYAGAGLPWPVGRWHHHHL*S*SGAGA*RCTVYYLGASADPQRSGSDPFWRYR*QHDFTVKKQSVAGRYAVGGTDLCG*SPGSEAVAAQPLP
ATDAGTYAAG*KRQQPGGAGRV*NL*PPA*RG*PSHRQQTG*RRAAGCSRYPSTG*SADREICPCID*CSA*RSRRKTVCRAVSSGSSACSEP
EHS*RRTDRH*EQPSAGNGKPGSGRLASGCPAFDRCNASV
>NC_000913.3_cds_NP_414602.1_58_1 [gene=polB] [protein=DNA polymerase II]
[protein_id=NP_414602.1] [location=complement(63429..65780)]
VAQAGFILTRHWRDTPQGTEVSFWLATDNGPLQ-SYACTARVRGVYSRRSGSPRSAYFAG*TRLSPDTAGVKGFSPPAGVWPLLSRPSPIDEL
RKAPA*RWRYRLRGRCASARTLSDGAVYHLTGVGRG*YAQWHYR*CPSETASRLSSAAQVGFYRY*NHPPR*AVLHRPGRLRAAHRLYAGAGE
WRRLLA*FRTGIRRQPPAVAGKTQRLVCQLRS*CDHRLERGAVRSANAAKTCRALPSSAASWAR**RAGVARARL*KRRLFCPG*RSANYRRY
RGAEIRVLEFLFILAGNCRSGAIRRRKIYR*PVGSNGRN*PPFRRR*TCAGNL*PERLRAGDADLPQN*NHAIFTRTGNGERPAGGPTRRFGG
GIWSSLFSANASRWLCRA*SRRSAAARQPWRLRDGFTARAL*FSAGAGL*KPVPVDHPHLSD*SRRAGGRHGAA*SRAQYRRFSRCLVLARKT
LPAGDCD*HLARAR*SQTPG*QTAVAGAENHHECLLWRARHHRLPLLRSAAGIVDHHAWSSDHAANQSVD*STGLRRYLRRYRLNVCLAERRT
FGRRSGENRSCTGAAR*RLVGGNAAKTTADQRIRTGV*NPFLPFSDANHSRSRYRQ*KALCRTDSGGRQAADGV*RAGNRAHRLDAAGPAVSA
GAIPAHLPQRAISGICTRNHRQTDGG*TGCATGLP*TPSPSAERVSA*CAASCTRRSPCR*RKPKAWSPLAISESRHH*VRMDHQRPGAAGLP
TFTTGLRTLSDPPATTRGGGNTPFY*G*FCYTYDRATWAIL
>NC_000913.3_cds_NP_414603.1_59_1 [gene=araD] [protein=L-ribulose-5-phosphate 4-epimerase]
[protein_id=NP_414603.1] [location=complement(65855..66550)]
MLEDLKRQVLEANLALPKHNLVTLTWGNVSAVD-TRARRLCDQTFRRRLQRHDR*RYGRG*HRNR*SG*RYEKALLRHANSPAALSGIPLHWR
HCAYALAPRHHLGAGGSVDSSNRHHPRRLFLRHHSLHPQNDRRRNQRRI*VGNR*RHRRNL*KTGYRCSANARRSGPFPRPVCMGQKCRRCGA
*RHRAGRGRLYGDILPSVSAAVTGYAANAAG*TLSA*AWREGILRAV
>NC_000913.3_cds_NP_414604.1_60_1 [gene=araA] [protein=L-arabinose isomerase]
[protein_id=NP_414604.1] [location=complement(66835..68337)]
MTIFDNYEVWFVIGSQHLYGPETLRQVTQHAEH-SR*CAEYGSETALQTGVETAGHHAG*NHRYLPRRELRRSLRWSGGVAAHLLPGQNVDQR
PDHAQQTVAAIPHPVQRGAAVGQYRYGLYEPEPDCTWRSRVRLHWRAYASATCRGYRSLAG*TSP*AYRLLDASGGL*TGYPSSESLPIWR*H
A*SGGHRWR*SCRTDQVRFLRQYLGGWRSGAGGELHQRRRC*RAGR*VRKLLHHDACHTNPRQKTTERAGSGAY*AGDEAFPGTRWLPRVHHH
L*RFARSETASWSGRTASDAAGLRLCGRRRLENCRPASHHEGDVNRSAGRHLLYGGLHLSLRER**PGARLPYAGSLPVDRRRRETDPRRSAS
RYWW*GRSCPPDLQYPNRPSDCRQLD*SRRSLPSTG*LHRHGENTALPAETAGGECAVESATGSANCFRSVDPRWWRAPYRLQPCTEPQRYAP
IRRDARH*NHGD**RHTPASV*RRAALERSVLRVSSL
>NC_000913.3_cds_NP_414605.1_61_1 [gene=araB] [protein=L-ribulokinase]
[protein_id=NP_414605.1] [location=complement(68348..70048)]
MAIAIGLDFGSDSVRALAVDCATGEEIATSVEW-ISPLAERAIL*CPE*PVPSSSA*LH*VNGSGTENRACRA*RRTARSCGRDWR*QYRLDA
RTD*CRRKRAGAAPGVCRKPERDVRIVERPHCG*RSGRDYPFVPRAGQR*LLPLHWWYLFQRMVLGKNPACDSPGQRRGAICRIVD*AVRLGA
SSAFRYHPPAGYSSRTLQRRA*ISVARKLGRPAASQFL**AGPDPQSPFAFPAVH*HLDCRYSGGHLMPGMGAASRPA*KRGDFRRRV*LPYG
RSWRRRTA*RTGKSYRYFHLRHSDCRQTERWRAGS*RYLRSG*WQRGAWIYRSGSRPIGVW*YLRLVWSRTRLAAGTACRPASGTENANQRQP
ETTASGADRSMGQKSVSGSPAGGARLV*RPPHTER*PTPERGDYRS*PRYRRSAAVRRFDCCHRLWRTRNHGVLYRSGDRR**RDGTGRHRAE
KPGHYAGLLRRAESPAANCCL*PVLCARCGDFCCRRRESARRHPISSAKNGQCGRENPATVQRAGTTL*TALSPLSAMGDERRTTLSSNFRPG
TGCPGRCDSI
>NC_000913.3_cds_NP_414606.1_62_1 [gene=araC] [protein=ara regulon transcriptional activator;
autorepressor] [protein_id=NP_414606.1] [location=70387..71265]
MAEAQNDPLLPGYSFNAHLVAGLTPIEANGYLD-IFYRPTAGNERLYSQSHHSRSGGGEKSGTRICLPTG*YFAVPARRDSSLRSSSGGSRMV
SPVGLLSSARLLA*MA*LAVNICQYGFLSPG*SAPAAFQRPVWANH*RRARGRALFGAAGDKSA*AIVTAAHGSD*RVAPSTDG*SGTRGLSV
HQRSPGRQQF*YRQRRTACLLVAVASVTSFPPAVRD*RLKLARGPTH*SGEAAFEHYPDAYRHRRSQCWF*RSTLFLASI*KMHRGQPERVSC
RL*RKSE*CSRQVVI
>NC_000913.3_cds_NP_414607.1_63_1 [gene=yabI] [protein=DedA family inner membrane protein]
[protein_id=NP_414607.1] [location=71351..72115]
MQALLEHFITQSTVYSLMAVVLVAFLESLALVG-IDSTRYGADGGAGSADWQRRVKFLARLAGRDYWLLDGRLDFFLAGLAF*KAVASLVISE
EKQSTT**N*TCVASTQHVHHSGRSFCWPDASAGANGGGNAGSAGG*IYYAEYYRLPAVAAVLLPARDSGGRGDRYSCRNAER*V*MVAAGNS
GVFVGWWLAVLAVMAQR*SD*PFESLFVPRSFVVADAVDFCHRRGGAGGVNSPPVDAGVYRYFA*SGWGL
>NC_000913.3_cds_NP_414608.1_64_1 [gene=thiQ] [protein=thiamine/thiamine pyrophosphate ABC
transporter ATPase] [protein_id=NP_414608.1] [location=complement(72229..72927)]
MLKLTDITWLYHHLPMRFSLTVERGEQVAILGP-KRRG*KYPAEFDRRFSDASQRFADYRWRRSHNYAAVTPSGVDAVSGEQPVQPPDGRTEH
RAGAKSGIETERGTAGENARYRPPDGD**FNGAVTGRAFRRSATASGVSALSGTRTADFIAR*TVLCARSGVTSGDVDAGEHELPAAKNDAID
GVAQRGRCGADRHALGSSRRRAHRLAGYDQ*VVER*GECFGTIGDYGL
>NC_000913.3_cds_NP_414609.1_65_1 [gene=thiP] [protein=thiamine/thiamine pyrophosphate ABC
transporter permease] [protein_id=NP_414609.1] [location=complement(72911..74521)]

MATRRQPLIPGWLIPGVSATTLVVAVALAAFLA-TVVERAAG*LGGSLAGQLSVACGALLLLAGVSLGTALCHTRDIPRPRALSQALSGSAGA
VASVCNDLDPPGVGRCFRHS*RLWSPGLAGNTLPIARSGVDLFALRPARYFAGPCVF*SADGEPLITPGTGKHPRRTASTCRPAWDA*LAFFP
LRRMAVVTATNPAGCCAYLYALFRQLRHRAIAGGRSAGDHYRAGNLSGAELRLRSCPRGNAGAAPDGVLPRAGAVESAIE*GHCARHHAAARL
ARPGRSSA*PHLRHGVNCAGAAAVAATVTGGDRRWGKSPVAGSAGTTGAVAGAVDLVAYCAGGRCIVRSADHDAAMEQSRTAGAAENAGGSGA
GDERHVDPRHAGDCAGYRLLFTAQQHYRPATIC*RHCDFHQCVNGDPLCAESAGKPDARYHRPLQHVMSVAGD*RLVTLKSGGAARPETSTGA
GAGLCMRAVDW*FWRGGVVR*R*FPHPAVLSLPANWLLSQPGRCGHRVNSAAALFSAVYRD*KTTGAKC*N*L
>NC_000913.3_cds_NP_414610.1_66_1 [gene=thiB] [protein=thiamine/thiamine
pyrophosphate/thiamine monophosphate ABC transporter periplasmic binding protein]
[protein_id=NP_414610.1] [location=complement(74497..75480)]
VLKKCLPLLLLCTAPVFAKPVLTVYTYDSFAAD-MGAWSGG*KSL*SRL*LRTETGGAGRWRFASQPSTDGRQKQ*SRCGAGAG*QPVRRRQ*
NRTVCQKRCGSGCR*RSRRLE**HFRTV*LWLLRLRL*QEQTEKPATKPERTG*ERSKLAGDLSGSAHQYTGAGSVAMDAKSLWR*RPTSLAE
TGEENGHGHQRLERSLRPVFKR*KRSGTELHHLSGLSHSRREER*LRRRELQRRSLSASGSRRPHRCQQAAGAGAKIPPVYGFSGFPECDPNR
QLDVSGGKRHAACRF*KIDQTRNHVGVHASRSGGTTSGMD*RMATRRQPL
>NC_000913.3_cds_NP_414611.1_67_1 [gene=sgrR] [protein=transcriptional DNA-binding
transcriptional activator of sgrS sRNA] [protein_id=NP_414611.1]
[location=complement(75644..77299)]
MPSARLQQQFIRLWQCCEGKSQDTTLNELAALL-KLLASSYAHPAQHHAGSRLADVGSGSRAR*TLASDIPLYRAGASATAGGRPAGAGSYRS
TGAVGWRQSDCAANAGFSSGPQLPPGAAHPARALLSSVA*SATWQRIAPFRNPYRPANLQFANAHK*GKWGTGSRHRPPLAANFTASLAFLFA
SRSPFSPWS*TGNGRCDRLFKTNQYAAALFAYC*HCVADALDAGYPSHATGPLVTVTAGASSGDDPAARMGNPQ*LCQPSHRHRSVCGDSQQH
QSTENSGIR*LLRLPGINRRS*RLGSAGNCRRASRRADAKRSTGRGKRD*KPPGGRLLLFTVRQPHPSRGESASQGLGKLCAFSN*SGLFR*G
TVPATVVPGLWTAPPLAPCPHHKERKTGWPGKPHPNLLSGSQ*ASGDCRDHAADSGKSPGHAENQRDRLRSVAYRRDRK*YLAKQRQLYPAAG
LLCFRTFMRSATATTLHSH*LASRRCSLAQWRDESGELVPATGRQQSDGAIIAPLADHSGATQYARPAHEYPRLVRF*ISVVCATGSM
>NC_000913.3_cds_YP_002791237.1_68_1 [gene=sgrT] [protein=inhibitor of glucose uptake]
[protein_id=YP_002791237.1] [location=77388..77519]
MRQFYQHYFTATAKLCWLRWLSVPQRLTMLEGL-NAVG*PQF*KL
>NC_000913.3_cds_YP_025293.1_69_1 [gene=setA] [protein=broad specificity sugar efflux system]
[protein_id=YP_025293.1] [location=77621..78799]
MIWIMTMARRMNGVYAAFMLVAFMMGVAGALQA-TYIELISES*GWRATFLDRPLLYGECYCWDRRKPLVGKTF*QSGRSAKTDYILLFDGYR
QCAIVCI*SSLSDAYHLWCASGISGQYGNATVICSGAGICG*LGARSGDV*LGDACAAFSGMGYRSTVGLYAGVELRLYGDVFDCRRDIHTQS
GIDCIYASVCGAGRTAVGKCFINARWLAG**RTDVICRLDVNVDLQHHVHY*YAVVDQ*RVRIARQTGGFPDGDGSWTGNTSNDSGWLLCQTL
W*AANDGHSSGGRSTVLHRIDFL**PYGVDDAATF*RCIYRHCCGYWDAMVSGFNAWKSGGSYHLIY*QYFYRGNSGWRYSGSNCTKLGALCC
LLGNCGYFCCRIIFNRKG*RRL
>NC_000913.3_cds_NP_414613.1_70_1 [gene=leuD] [protein=3-isopropylmalate dehydratase small
subunit] [protein_id=NP_414613.1] [location=complement(78848..79453)]
MAEKFIKHTGLVVPLDAANVDTDAIIPKQFLQK-SDPYGFWRASV*RLAFSG*KRPTAKPGLRAELPAVSGRFHFAGTRKLRLWLFA*ARALG
IDRLRF*SGDCAEFC*HLLRQ*L*QPAAAGEIKRCRSGRTVCAGES*SGDPFRRGSGSARGESGRENLSLYHRCLPPPLHDERSGQYWAYLAA
RRRHCRL*SKTTCVYEL
>NC_000913.3_cds_NP_414614.1_71_1 [gene=leuC] [protein=3-isopropylmalate dehydratase large
subunit] [protein_id=NP_414614.1] [location=complement(79464..80864)]
MAKTLYEKLFDAHVVYEAENETPLLYIDRHLVH-RSDLTAGVRWSARPRSPGTSAGQNLRYHGSQRLYPDQRH*CLR*NGAYPDAGTDQKLQR
IWRRTV*PESPVSGDRPRNGAGTGRHLAGDDHCLRRLAYRHPRRVWRTGLWYRHFRS*TRTGNANPETGPRKNHEN*SPGQSRAGHYRKRYRA
GNYR*NR*RRRHRACGGVLRRSNP*FKHGRSYDPVQYGNRNGRKSRSGCTGRNHL*LCQRPSACAERQRFRRRRCLLENPANRRRRNFRYRCH
SASRRNFTAGHLGHQSRPGDFRERQYSRSGFVCRSG*TRVGRKSAGLYGAETGYSADRSGYRQSVYRFLYQLAH*RFTRGSGDRQRAKSRARR
AGTGGSRLWPGKSPGGSGRSG*NLY*SRF*MALAWLLNVSGDEQRPSESGRTLCLHQQP*L*RPPGARRAHASGQPGNGCRCCCDRTFRRHSQ
H*I
>NC_000913.3_cds_NP_414615.4_72_1 [gene=leuB] [protein=3-isopropylmalate dehydrogenase,
NAD(+)-dependent] [protein_id=NP_414615.4] [location=complement(80867..81958)]
MSKNYHIAVLPGDGIGPEVMTQALKVLDAVRNR-ICDAHHHQPLRCRRRSH**PRATTAACDG*RL*ASRCRAVWLGRRPEVGTFTTRPATRT
RRAAASA*ALQIIQQPAPGKTVSGAGSILSAACRHCRKRLRHPVCARTDRRHLFRSAKRPRR*RTI*KSL*YRGVSPF*DRTYRPHRV*ICSQ
ASPQSDVDR*SQRAAILYFMAGDR*RDRHGIPGCRTGAYVHRQRHHAAD*RSITV*RSAVLQPVWRHSV*RVRNDHWLDGDVAFRQPERARFW
TV*TGGRLGTRYRRQKHRQPDCTNPFAGTAAALQPGCR*CGLRH*TRH*PRIRRRHSHRGFSPWRCRR*YR*NGRYHCPLCSRRGV
>NC_000913.3_cds_NP_414616.1_73_1 [gene=leuA] [protein=2-isopropylmalate synthase]
[protein_id=NP_414616.1] [location=complement(81958..83529)]
MSQQVIIFDTTLRDGEQALQASLSVKEKLQIAL-SP*AYGC*RDGSRFPRLFAGRF*IGANHRPPG*KQPRMCVSSLRGKRYRRGGRIPESRR
SLPYSYLYCHFANAHRHQAAQHAGRGDRTRYLYGETRP*LHR*C*IFLRRCRAYTHCRSGASGRSGD*CRCHHHQHSGHRGLHHAV*VRRNHQ
RPV*TRA*HRQSHYLRTYPRRFGPGGRKLTGGGTCRCTPGGRRNERDRRACRKLFPGRSHHGDQSS*GYSQRPHRH*SPGDMAHQPVS*PDL*
YADPGKQSHCWQRRIRTLLRYTPGWRAEKPRKLRNHDTRIYWSEPNPAESDLSFGACGGETSHG*DGV*RK*I*FRQFVRCFPEAGGQKRSGV
*LRSGGAGLHR*AARRAGAFPSGLLQRAVWL*RYRHRRRQTGLWRRSQSRSRQR*RSGRCRLSGN*PHH*I*RRTGEIQPDRQRPR*RCAGSG
GYRR*LQRSPLPRRRPGYRYCRVICQSHGARSEQYLACRRSRKRVATQSSTQRKQQGNRV
>NC_000913.3_cds_NP_414617.1_74_1 [gene=leuL] [protein=leu operon leader peptide]
[protein_id=NP_414617.1] [location=complement(83622..83708)]
MTHIVRFIGLLLLNASSLRGRRVSGIQH*
>NC_000913.3_cds_NP_414618.4_75_1 [gene=leuO] [protein=global transcription factor]
[protein_id=NP_414618.4] [location=84368..85312]
MPEVQTDHPETAELSKPQLRMVDLNLLTVFDAV-NAGAKHYSCRSCSGNVATCGQ*RCCTPEGDV**RAFCSLWPWYSTDCSRISTFWFSSSG
IATSTK*IAWFRF*TREQ*TCISSLCLQPVRQHSDLADL*SH*ADCAKYTCYVQVFIKSEH*TSAALSGNGVCD*L*RLPSS*IYQRTII*R*
NGAGSQQKSSNN*GPVTET*CL*RTTCGGFARSFRVI*STLV*HGR*ASQYRVSGHGNDERT*RGVANAFGRYCAALAG*RVR*ILRITGITA
AVKTKQQNLLSLLA*SCRAR*RPSVDGRAISLNLQTL

>NC_000913.3_cds_YP_025294.2_76_1 [gene=ilvI] [protein=acetolactate synthase 3 large subunit] [protein_id=YP_025294.2] [location=85630..87354]
MEMLSGAEMVVRSLIDQGVKQVFGYPGGAVLDI-I*CIAYRGWY*SCISSS*AGGGAYGRWPGARDRGSRRRAGNVGSRGDQCDYWHRHRLYG
FHSISCPFRAGSDLVDRLRCLSGVRHGGDFATGG*TQFSG*ANGRHSAGAEKGFLAGGKWSPRTSSR*FTERYS*SGEQITLCLAGVGQYAFL
QSHYYRT*RAN*ACSANAGSGKKTGCLRRRWGNHGGLPSAVERNGGGVESARCLLIDGAGGVSGNASSGTGHAGNARYLRSQYDDA*RGCDFR
RRGTI**PNDEQSGKVLPKCHCSAYRY*SYFHF*NRDCGYPDCGGCSPGPRTNA*TLVARIRPSTTG*DPRLVAAN*TVARSSVPEI*HSQ*K
D*TAGGDRDSLAVDEGRRLRDVRCRAAPDVCCTLLSIRQTASLDQFRWPRHDGFWFTCGTGRQNGVARRNRGLRHWRRQYSDEHPGTVYRVAI
RVARTGGESQ*PLSGDGEAVAGHDLFRPSFTILYAIATRFRPSGGSLWACRDPDFSSA*AGKQT*RGAGTGAQ*SPGVC*CYRRWQRARLPDA
DSRGRNG*NVVKQNGENL
>NC_000913.3_cds_NP_414620.1_77_1 [gene=ilvH] [protein=acetolactate synthase 3, small subunit, valine-sensitive] [protein_id=NP_414620.1] [location=87357..87848]
MRRILSVLLENESGALSRVIGLFSQRGYNIESL-NRCANRRSDIIAYDHPDRGR*KST*ADRKAITQTGRCLARE*VGAGRAC*AGNHAGENS
GQRLRA*RSET*YGNIPWANYRCHTLALYRSISRHQR*A*CIFSIDSRCGENCGGCSLWCGRTFARR*NNAL
>NC_000913.3_cds_NP_414622.1_78_1 [gene=cra] [protein=transcriptional repressor-activator for carbon metabolism] [protein_id=NP_414622.1] [location=88028..89032]
VKLDEIARLAGVSRTTASYVINGKAKQYRVSDK-NR*KSHGCGA*AQLPPERRGSWASCWTHTFYWSCDPRSGEHQLYPHR*LS*TPGAATGL
STADCLLRRSARQRNAVH*APFTASG*CHYCFDVVAS*ASFLSTLG*RPVPDCRAGPRPRS*TLHQRGWCRSG*CRNAGGRVT*VSRRDGALS
WCATGAFCQLPA*TRFPYCLER*SARSAFPVCQQL*AGGGCPVIRKMAGNASDAAGAVHNVVCVVARSDGCHAASRRQTAF*PGNCHLWR*RT
ARLLTVSGAGSGSTSPRCRRACAGDCPGKPGRTA*AKTWFNAH*T*SLSPRRAQP*L
>NC_000913.3_cds_NP_414623.1_79_1 [gene=mraZ] [protein=RsmH methytransferase inhibitor] [protein_id=NP_414623.1] [location=89634..90092]
MFRGATLVNLDSKGRLSVPTRYREQLLENAAGQ-NGLHH*HLSPVPAALPPA*MGNYRAKIIASVEHEPG*APCAAPTVRSCQRMSDGWRRSI
VNRASTAATCRADKRSDAGWTVQQV*AVG*NNLASTGQGRYRRRAVGYRRLIGATAGLVSI
>NC_000913.3_cds_NP_414624.1_80_1 [gene=rsmH] [protein=16S rRNA m(4)C1402 methyltransferase, SAM-dependent] [protein_id=NP_414624.1] [location=90094..91035]
MMENYKHTTVLLDEAVNGLNIRPDGIYIDGTFG-TRWSLTSDPLAAWRRGAFAGDRSRPAGYRRCEDY**SALLHHPRTFLRAGRIRCRARSY
RQDRRHSPRSWRLFTAT**C*TWLFLYARWSAGHAYGPNPWAVSR*MATNRRRSRYRLGIENLW*RAFCQTHCPRHCRA*PRTADDPHQRTGG
SRGCCNAGER*V*TSRDPYLPGGAHLGKQ*TGGDRAGAKKLAQRAGPGWAAFDHQLPLAGRPYCETFYA*KQPRSASSGRVTDD*RAAQKTGW
PSAASTRQVNAGRRRGG*EPSCP*FSSAYCREDECM
>NC_000913.3_cds_NP_414625.1_81_1 [gene=ftsL] [protein=membrane bound cell division leucine zipper septum protein] [protein_id=NP_414625.1] [location=91032..91397]
MISRVTEALSKVKGSMGSHERHALPGVIGDDLL-TIWEAATLPVHLHYFDGGDCGNHGAPYPFTDRSARTTGAGARCFRH*MAQPDP*RECAR
RP*PGGKDRHGKAANAAC*SVTRKYRSAKI
>NC_000913.3_cds_NP_414626.1_82_1 [gene=ftsI] [protein=transpeptidase involved in septal peptidoglycan synthesis; penicillin-binding protein 3] [protein_id=NP_414626.1] [location=91413..93179]
MKAAAKTQKPKRQEEHANFISWRFALLCGCILL-SAGFSARTRSVVTSYLPGYAGERGRHAFSSRSASFHLPRHDY*PFWSPVSGERAGKSDL
G*PERSA*RWRYQRR*PLEGAG*RAQYSAGSAFSPH*RQPERALYLSGASGEP*HGGLHQKTETAGDSSA*RVSPLLSVRRSDCSPHRLY*RR
*SRD*GR*EEFR*MAYRAAG*AHCA*RPLWSRN*RYFFY*QPGSAQPGAEY**TPAGAGLSRTEQRGGL*QG*IW*RRAGGCQHR*SAGDG*Q
PVIQP*QSERHAERGDA*PYHHRRV*TGLNG*TDGGNDRVATWRGAGKLGTQYHSLSN*RPRNQRRGTLQRINPDRGITEVE*RRCFQAGVSD
AVLSVSRYLLTFWTGKSDQFGVGRRTQWLISSKTTVV*HREGHLLFRLRANGNTITVSASLRNYRQLRHLSPTVDYQS*PPGSR*TCLPGIHC
PHCGAYDGKRGATRRRRREGGD*RLSYRH*NRYREKGRAGRSLHQ*IYCLYRRRCACESAALRAGCCYQRSAGG*ILRRRRFRAGLWCHHGRR
IAYHEHRAGCADNGR*K*ICD*SRRGDRWQIV
>NC_000913.3_cds_NP_414627.1_83_1 [gene=murE] [protein=UDP-N-acetylmuramoyl-L-alanyl-D-glutamate:meso-diaminopimelate ligase] [protein_id=NP_414627.1] [location=93166..94653]
VADRNLRDLLAPWVPDAPSRALREMTLDSRVAA-SGRSLCSCSRSSGGRASIYPAGDSARCGCHYCRGER*GDRW*NP*NARRTGHLSQPAQR
AFICTGGPLLP*TL*QFTSRGRNGHQRQNHDYPAVGAVEPTAWRNQRGNGHRW*RPAGESDPDRKYNRFGSRCSA*AGGAGGSGRDVLRNGSF
LPRAGTAPCGGIEICGVGLYQLKPRSP*LSW*YGTLRSREMAALF*ASLRSGDY*RRR*SGPPLAGKTAGRGCGINGRSY*SELSRTLVESDR
SELSRQRCDDSL*LKLGRWRN*KPSDGRF*RQQPAARAGDTVGTRLSTG*SAENRRASATGLRTYGSVHCARQTDGGGGLRAYAGCTGKSLTG
GASALCGQAVVCLWLWWRSR*R*ASTDGRNCRRVC*RGGGDGR*PAYRRTACHHQRYSGGNVRCRTCQSDGRPC*SGDLRRYAG*RE*CGTGR
GQRP*RLPDCWQSASGLLRSRHGGASAGGDCM
>NC_000913.3_cds_NP_414628.1_84_1 [gene=murF] [protein=UDP-N-acetylmuramoyl-tripeptide:D-alanyl-D-alanine ligase] [protein_id=NP_414628.1] [location=94650..96008]
MISVTLSQLTDILNGELQGADITLDAVTTDTRK-TDAGLPVCCPERRTF*CPRFCRPGESWRRRRTTG*PSAGHRPAAVNRQGYASGVW*TGC
MGSPASSGARGCSDGVLRQNLR*RDDGGDFKPVRQHALYGRQSQQRHRCTDDAVALNAGIRLRSY*TWREPSGRNSLDCESDSPGSCAGQQPG
SGASGRFWLACGCRESER*NL*RPAGKRYRHYERRQQRLAELAERNWLTQSVAFLTQCRQQRFHRHQYPCDLARYGIYPTNPNR*RRCSAAVA
GASQYCECAGSRCALHVRGRNA*CYQSGAGKSESCSRPSVPHPTGRKPVAARRLLQRQCRFNDCSSPGTG*NAGLPRAGGGRYGGTGR*KRSL
PCTGGRGGKSCWY*PRVKRG*TKPCYQHRQRRWRTFC**NCVNYAS*ITDC*ATGNYDFS*GFT*CRHGRGSTRFTGEWDML
>NC_000913.3_cds_NP_414629.1_85_1 [gene=mraY] [protein=phospho-N-acetylmuramoyl-pentapeptide transferase] [protein_id=NP_414629.1] [location=96002..97084]
MLVWLAEHLVKYYSGFNVFSYLTFRAIVSLLTA-TVHLIVDGPAYDCSFAKTFLWSGGA*RRS*ITLQQARYADHGRDYDPDGDCDLRTAVGL
PVQSVRLVRVGGAGRLRCYWLC**LSQSGA*RHQRVDRSLEVFLDVGHCAGCRLRPVPCRQRHARNAAGGPIL*RCDAAAGAVLHSAGLLRHC
GYWQRGKPDRWSRRPGNYADRICRRWFCAGGVGDRQYELCQLLAYTVSATRRGTGYCLYRDSRGRTGLPVV*HLSGAGLYGRCRFAGVRWCVR
HYRRTATSGIPAGDYGGRVRGRNAFCHPAGRLL*TARTTYFPHGTDSSPL*TERLAGTARHCAFLDYFADAGSDWSGNAEGTL
>NC_000913.3_cds_NP_414630.1_86_1 [gene=murD] [protein=UDP-N-acetylmuramoyl-L-alanine:D-glutamate ligase] [protein_id=NP_414630.1] [location=97087..98403]
MADYQGKNVVIIGLGLTGLSCVDFFLARGVTPR-SYGYAYDTAWPG*ITRSRRTPHGQSE**MADGGRSDCRQSRYCTGASILKRCR*CRNRN
RWRYRAVLSRSTSTDCGDYRF*RQKHGHHASG*NGESGGG*RWCGWQYWPACVDATG**V*TVRAGTVELPAGNHLQLTGGSSDHSERD*RSY

GSLSVWFTTVSCSKTAHLRKRESLRG*C**CLNNADSRCG*TLRQLWRQHG*LSPESSAGRNLAAG*RRESAECERDETFRAA*LHQCAGGAG
AGRCCRVTACQQPESVNHIHWSAASL*SCAGA*RRTLD*RFESDQRRQYGSGAEWPARRRHTAFVAGWRW*IGGL*PTGALPEWR*RTSVLFR
S*RRAAGGATPGSGRTNRNYGTGDALAGSACSAGRYGSALPSLCQP*SVQEL*TTRQ*VCPSGEGVRL
>NC_000913.3_cds_NP_414631.1_87_1 [gene=ftsW] [protein=putative lipid II flippase; integral membrane protein; FtsZ ring stabilizer] [protein_id=NP_414631.1] [location=98403..99647]
MRLSLPRLKMPRLPGFSILVWISTALKGWVMGS-TGKRYRQPDHVRSHLTVADLRPRGDWLYHGDLGVNAHRATLNQRSVLLREA*WCLSDFG
VYSGDHYAASADGVLATLQCHDAARIYHPADDRPGSG*LG*RGIALDRSRFAAYPACGADKTVAVLLYRQLSGA*RRRST**PARLPETDGRD
SGVGSVTAGTARPWYGGGVVCDYAGDVVPGGSEIVAVHCHYRYGHFSGCVADTRRTVPYPPCYRILEPVGRSLWQRLSVNAIADGVWSRRTLG
ARFR*LGTKTGVSAGSAH*LYFRHYRRRTGVCRCGAGTFNGILRRFSRDVDWP*SIRN*PPFFRFSRLFYWHLV*LPGAG*RRRGGGDVTDQR
SDIAADQLRWFELTDYVDSHHDAVAY*L*NASGESAGVCTRFTM
>NC_000913.3_cds_NP_414632.1_88_1 [gene=murG] [protein=N-acetylglucosaminyl transferase] [protein_id=NP_414632.1] [location=99644..100711]
MSGQGKRLMVMAGGTGGHVFPGLAVAHHLMAQG-MASSLAGDCRPYGSGLSAKTWHRN*FHSYLWSAWKRYKSTDSCPAAYLQRLASGAGDYE
SVQT*RGARYGRLRVRSRWSGRVVVRHSGCTS*TKRYCGLNQ*MAGEDCHQSDAGVSRCFP*CGSSG*PGAYRCVGAAVAAATFGWT*RSGSC
AGSGWFSGRTHS*PDNAAGCCETG*FSHYLASERQRFATIR*TGVCRSGATAA*SDGIY**YGGGVCVGGCRRLPLRCVNGE*NRRGRTTGVV
CAVST*RPPAILECATAGKSGRSQNYRAATA*RGCCRQHPGRVVARNLINHGRTRPRCIHSGCHRASGK*SEPGCPGV
>NC_000913.3_cds_NP_414633.1_89_1 [gene=murC] [protein=UDP-N-acetylmuramate:L-alanine ligase] [protein_id=NP_414633.1] [location=100765..102240]
MNTQQLAKLRSIVPEMRRVRHIHFVGIGGAGMG-RYCRSSGQ*RLSDQWFRFSAKSGHAAVNESGCDDLFQPSPGKRT*CQRGRCFQRDFCR*
PGNCRRS*SAYSGDPSCRNAG*VNAFSSWHRHCRNARQNDNHRDGFQHLRRSGARPNLR*RRAGKSGGGSCAFGAWSVPDCRSR*E*CIVPAS
ATDGGDCHQYRSRPHGYLPGRL*EFKTDFY*FSAQPAVLRSCGDVC**SGDPRIVTASGASDHDLRLQRRCRRACRRLSADWPAGALYAAAPG
QRADARHPECARSS*RAERRSCGCGCYGRGH*RRGYFAGA*KLPGDWSPF*FPR*IPAGASEW*KRYGNAGR*LRPPPDGSGRHH*SGARRLA
G*KPGNAVSAAPFYPYARPV**FRQCADAG*YPVDAGSVSGWRSANSGSGQPFAVSHNSWTWEN*SHSGAGSGAGSRDAGTGINR*RPDSRSG
GW*YWKNCPFFS*NQTEAANSGGRTT*L
>NC_000913.3_cds_NP_414634.1_90_1 [gene=ddlB] [protein=D-alanine:D-alanine ligase] [protein_id=NP_414634.1] [location=102233..103153]
MTDKIAVLLGGTSAEREVSLNSGAAVLAGLREG-RY*RVSCRPERSRRDATEVDGLSESVYRATRSRR*RWYAAGDARADGLALYRKRSDGIC
AFNG*TTQQTSMARCRFTGRAVGSVNPRRV*KRPER*AVSRNFCSGFAGYR*AEPRRFQCGNVKSSSRKCSTRCIKIGISAR*RSID*KMAKW
AGVHGCDTR*RNFTVNTYSTVRNLL*L*GEVSL**DTVFLPRRSGSVTRGQFAGISAESMDDVRLQRMGTY*RYAGQRWTVLSAGSQYLTGYD
QPQPGADGGTSGRYELLAVGSTNSGTGGL
>NC_000913.3_cds_NP_414635.1_91_1 [gene=ftsQ] [protein=divisome assembly protein, membrane anchored protein involved in growth of wall at septum] [protein_id=NP_414635.1] [location=103155..103985]
MSQAALNTRNSEEEVSSRRNNGTRLAGILFLLT-SFNDSVGERLGRVGLDGRCATPAALKAGVDR*TPLHT**RYPAVDPGIG*AGYLYDPGC
QHHPDANRTTPAVD*AGERQKAVA**IEDSSG*ICADCAVE*STYGRRGRKYLQRAARTHQQAGASNAVWPGRQRQ*SVAGLSRNGADAGKGQ
IYSEGSGDDRAAFLAVDAE*RY*AQSWPGRYDETFGSLCRTLSGFTAAGANRWQTD*LR*FAL*LWSGSRLGALAARGIYSATKSGTGRTTM
>NC_000913.3_cds_NP_414636.1_92_1 [gene=ftsA] [protein=ATP-binding cell division FtsK recruitment protein] [protein_id=NP_414636.1] [location=103982..105244]
MIKATDRKLVVGLEIGTAKVAALVGEVLPDGMV-KYHWRGQLPVAWYG*RRGERPRIRGQVRTTRH*PGRIDGRLSDLFGISGAFW*AHQLPE
*NWYGAYF*RRSDARRCGKRRPYREIGACAR*ASCAACDPARVCD*LSGRDQESGRTFGRADAGKSAPDHMSQRYGEKHRQSG*TLWAES*PT
DICRTGIKLFGIDGR*T*TGCLRRRYRWWYNGYRRLYRWGIAPH*GNSLCWQCRDQ*YRLRLWHAAKRRRSD*SSPRLCAGFHRWKR*ERGSA
ERRWSSATESATSDTGRGDRAALYRAAQPGQRRDIAVAGKASPTRG*TSPGGRHCINRWRSADRRSCSLCSARVSYASAYRRAAEHYRFNGLC
SGAVLFDGGGIASLWERVTS*R*S*SRKTCYSISWLVDQATQ*LAAKRVL
>NC_000913.3_cds_NP_414637.1_93_1 [gene=ftsZ] [protein=GTP-binding tubulin-like cell division protein] [protein_id=NP_414637.1] [location=105305..106456]
MFEPMELTNDAVIKVIGVGGGGGNAVEHMVRER-N*RC*ILRGKYRCTSAA*NSGWTDDSNR*RYHQRTGRWR*SRSWPQCG**GSRCIACGA
GRCRHGLYCCGYGWWYRYRCSTSRR*SGKRFGYPDRCCRH*AFQL*RQEAYGIRGAGDH*TVQACGLSDHYPERQTAESSGPRYLPAGCVWRS
ERCTERRCARYR*TDYSSGFDERGLCRRTHRNV*DGLRNDGFWRGER*RPCGRSC*NGYLFSAAGRYRPVWRARRAG*HHGGLRPASG*VRNG
R*HHPCICFRQRDCGYRYFS*PGYE*RAARNRCCDRYRHGQTS*NHSGDQ*AGSAASDGSLPAAWDGSADPGAEAGC*SRE*QCAANCERAGL
SGYPSIPA*AS*L
>NC_000913.3_cds_NP_414638.1_94_1 [gene=lpxC] [protein=UDP-3-O-acyl N-acetylglucosamine deacetylase] [protein_id=NP_414638.1] [location=106557..107474]
MIKQRTLKRIVQATGVGLHTGKKVTLTLRPAPA-KHRGHLSSHRLESTGRFPGRCQICA*YHALYVSGQRA*CTDFNRRAPQCCSRGLGHR*H
CYRS*RAGNPDHGRQRRSVCIPAA*RRYRRVELRQKICSHQRDCSCRRWR*VG*I*AVQWFFAGFHHRF*PSGY*FQQPALCDELLR*CVYAP
DQPCAYVRFHA*YRISAVPWFVPGRQLRLCHRC*RLSRTERRRPAF*RRICASQNARCDR*LVHVWSQYYWCIYRL*IRSCTE*QTAAGCPGE
TGSLGICDLPGRRRTAVGLQSAFSCTGI
>NC_000913.3_cds_NP_414639.2_95_1 [gene=secM] [protein=regulator of secA translation] [protein_id=NP_414639.2] [location=107705..108217]
VSGILTRWRQFGKRYFWPHLLLGMVAASLGLPA-TQQRRRTKRARKSDNPQPRAFSQS*LWSIGLAGSEHTPPEFELFR*LLASTCHSHGNPS
SFFRNGTANTARC*RIFASSGATSCITGYAQRAADPGRHAV*KGLSH*LCAFYPTSKIQHARLDKPGARHPCWPSTPHL
>NC_000913.3_cds_NP_414640.1_96_1 [gene=secA] [protein=preprotein translocase subunit, ATPase] [protein_id=NP_414640.1] [location=108279..110984]
MLIKLLTKVFGSRNDRTLRRMRKVVNIINAMEP-RDGKTLRRRTERENRRVSCTSGKRRSAGKSDPGSFRRGT*GK*ARLWYASLRRSVTRRY
GS*RTLHRRNAYR*RKNPDRNAACLPERTNR*RRARSYRQRLPGAT*RRKQPSAV*IPWPDCRYQPAGHASTGKARSLRS*HHLRYEQRIRL*
LPARQHGVQP*RTCTA*TALCAGGRSGLHPDR*SAYTADHFRPGRRQLGNV*TRE*NYSAPDPSGKRRLRNLPGRRPLLGGRKISPGEPDRTW
SGAD*RTAGERGHHG*RGVSVLSGQHHADAPRNGGAARSCAVYP*RRLHR*RW*SYHR*RTHRSYHAGPSLVRWSAPGCGSERRCADPERKPN
AGFDHLPELLPSV*KTGGDDRYC*YRSFRI*LNLQAGYRRCSDQPSNDS*RSAGPGLHD*SGKNSGDH*RYQRTYCERPAGAGGYYLHRKIGA
GVKRTDQSRY*AQRPERQIPRQRSGDCCSGRLSGCGDYRDQYGGSWYRYCARW*LAGRSCRAGKSDRRAN*KN*SRLAGTSRCGTGSRWPAYH

```
RYRASRIPSYR*PVARSFWSSGGCWFFPFLPVDGRCADAYFCFRPSIRHDA*TGYEARRSH*TPVGD*SDCQRPA*S*KP*LRHS*ATAGI**
RG*RSASRHLLPA*RTVGCQRCERNH*QHS*RCVQSDH*CLHSTTVAGRNVGYSGAAGTSEERFRPRFANCRVAG*RTRTA*RDAA*AHSGAV
HRSVSA*RRSGWC*DDASLRERRHAANA*LPVERAPGSDGLSASGYPPAWLRTERSEAGIQT*IVLHVCSDAGVVEI*SYQYAEQSSGTYA*R
G*GAGTTASYGSRAFSANAAA*PSG*RLCSRSCTGGANRRAQSRT*RSLPVRFW*KIQAVPWPPAI
>NC_000913.3_cds_NP_414641.1_97_1 [gene=mutT] [protein=dGTP-preferring nucleoside
triphosphate pyrophosphohydrolase] [protein_id=NP_414641.1] [location=111044..111433]
MKKLQIAVGIIRNENNEIFITRRAADAHMANKL-RVSRR*N*NG*NAGTGGGA*TSGRSRDYPPTFFAI*KTGI*IPGQAYNTVVLAGRTLGR
GAVG*RRATR*VDVAGRS*CR*FSASQ*TGNCEA*TSV
>NC_000913.3_cds_NP_414643.1_98_1 [gene=yacG] [protein=DNA gyrase inhibitor]
[protein_id=NP_414643.1] [location=complement(111649..111846)]
MSETITVNCPTCGKTVVWGEISPFRPFCSKRCQ-TDRPRRMGC*RKTNPQQWRSFRKR*LERRTKAV
>NC_000913.3_cds_NP_414644.1_99_1 [gene=zapD] [protein=FtsZ stabilizer]
[protein_id=NP_414644.1] [location=complement(111856..112599)]
MQTQVLFEHPLNEKMRTWLRIEFLIQQLTVNLP-NR*PRWRAAFLP*CQ*ITGCFRARRSPH*AVERT*PAAT*TPDLDWRAWRGPEPY*SIN
SAVKSGGERINFRAAYRAISA*RSFDCSGASATEHPRRLLQL*FTYIAHLAASTPGAARQPGRNLDCQPEPAHPGTYHGAGFNSPVGPLP*TN
QPEWFLSG*RWRCRLAAPESVARFTALSANFRT*EPFCHSFYAAGH*KRTGTGTSGFRTGLLL
>NC_000913.3_cds_NP_414645.1_100_1 [gene=coaE] [protein=dephospho-CoA kinase]
[protein_id=NP_414645.1] [location=complement(112599..113219)]
MRYIVALTGGIGSGKSTVANAFADLGINVIDAD-NYCASGG*TRCTCATCHC*SLWR*HDCC*WNIAAPGLARADLRQPGREKLA*RPAASAD
SARDATPDPASYFPLCTVGCAIAGRKLTV*KSESSACGGCQPRNAT*AHHAAR*CNSRACRTNPCCSGNARSPPCRGR*RH***RRTGCYRIG
CCPPARTLFAACVAVCLTGKTV
>NC_000913.3_cds_NP_414646.1_101_1 [gene=guaC] [protein=GMP reductase]
[protein_id=NP_414646.1] [location=113444..114487]
MRIEEDLKLGFKDVLIRPKRSTLKSRSDVELER-TIHLQTFRSELVRRADYRRKYGHRRHIFYGLCAGFF*YFDCCA*TLFCRRVASVYQQFF
R*CAETCDGFYRYV*CGFRKN*TDSRPEPGIKLRLY*RGEWLFRTLRAVRCESA*SVADQNHLCW*RSDW*NV*GAYPLRCRYR*SWHWPRFC
LYNSRQNRRRLSATFCGNRMCRCCARSGRNDRQRWWLHHAGRCGESLWRRCRFRHAWRHAGGPRRERRSHR*GERREIYAVLRHELRVCDETS
RWRRCGISRSRR*NR*AAAARPG*KYRARYFGRPAFSLYIRWGFTPERADQAHHVYSCAGTRKPHLQQPV
>NC_000913.3_cds_NP_414648.1_102_1 [gene=hofC] [protein=assembly protein in type IV pilin
biogenesis, transmembrane protein] [protein_id=NP_414648.1]
[location=complement(114522..115724)]
MASKQLWRWHGITGDGNAQDGMLWAESRTLLLM-STTATDGYPTKPEANRHQFCAVARR*KRGSHSSTGDATQSRVNAF*RAGSAGGTASQ*A
MASVAAIAGARSRTGHCFFQCLITLVRGISAALSGDDPHG*TDR*AG*MLL*TGASAKSPASVDRQSEISVTLSHHHFSDGNHGGCGNAAFCS
AGVCRYL*DLQHPTTGTNAGDHDAGRL*WRMELAAGVVRLSAGDSQ*VADAPTDLAYSAAEIAVTHPDYGFTDAGTKTHADLYDSGADTKCRH
YFFTGRRERQRNNALPVLGATSDTNPARYQ*RSTHLAGAKKYR*V*PALFAISENRRGIRLSGSHVRQPRPSSSGKHNGAGG*PRSLTGTGVA
DHNGRNYWYAGGGNVSANFPFRRCDEWDGI
>NC_000913.3_cds_NP_414649.1_103_1 [gene=hofB] [protein=T2SE secretion family protein; P-loop
ATPase superfamily protein] [protein_id=NP_414649.1] [location=complement(115714..117099)]
MNIPQLTALCLRYHGVLLDASEEVVHVAVVDAP-IA*ATGRIAFRYHQTY*DHLLDAPTNGRSRQSHTTDIARSCSGEASAQSRVANSNVTIC
AGTTRV*YSYRTSGQCLPHPLAYRRRIASFTGCFTGCRSRINRQIKSAGKPGYCGTSPAAGRAIHCRTGRKRRLISYCDLTMSGW*KGGIKVV
TAGGSGTGCQHAWNAAVTTGGLCSCLATTTGTGAGNWPYRQRQNGHAL*CPAKAEYR*H*YL*RRRSS*DPHSRTKPDANPSACRTHLSGRFA
CVIAPGS*RHHDRRDPRWRNSRDRY*SGANWSPGVVYPTH*FHLRNAGTFTANGGRPLDAIIGAYAGNSPASGTQTLPTLSPAARGAHPHSRQ
CMAIAAAPLAGTRLCTLLPRFLWSYGLI*SSAHNAGHSSAYFR*YRR*IAGNARTTGGYAYAF*KRLPGRRARLNHL*RVNPRTGDAAWRV
>NC_000913.3_cds_NP_414650.1_104_1 [gene=ppdD] [protein=putative prepilin peptidase-dependent
pilin] [protein_id=NP_414650.1] [location=complement(117109..117549)]
MDKQRGFTLIELMVVIGIIAILSAIGIPAYQNY-TAQSRTHRHATNLCALPYRRRVVRAGTWWIRYLRRWQQWHSLAYHHPLCFSHECGKGRG
VADRARKSQWAKRRHDTGLG*RKRRHRLDAQLQYSK*QRIAASLRRCLPL**RQL
>NC_000913.3_cds_NP_414651.1_105_1 [gene=nadC] [protein=quinolinate
phosphoribosyltransferase] [protein_id=NP_414651.1] [location=complement(117752..118645)]
MPPRRYNPDTRRDELLERINLDIPGAVAQALRE-RFRRNSRCQQ*YYGKTFTGKFSLSCHGDHPREWRLLRQTLG*RGVYSTGRRRCHHNLAC
G*RRCHQCQSILVRT*RPIPRAVNGRTHCA*FCANPFRSCQ*GTPLCRIAGRHQHAVVGYAQNLTRPAFSSEIRGTLRRRSESPSGAF*CLPD
QRKPYYCLRLSAPGGRKSVLAAPGCASRSRSRESGRT**SPESRSRYHHAG*LRNRTDARSRQTHQRQGATGSVWQRH*QNTA*ICRNGRGLY
LRRCAN*TRTSTRPFNAFSL
>NC_000913.3_cds_NP_414652.1_106_1 [gene=ampD] [protein=1,6-anhydro-N-acetylmuramyl-L-alanine
amidase, Zn-dependent; murein amidase] [protein_id=NP_414652.1] [location=118733..119284]
MLLEQGWLVGARRVPSPHYDCRPDDETPTLLVV-TQY*PAARRVWRSVDRRIIHWNY*SAGTSFLC*DRPFARLRSLFDSP*W*NSPVCSFR*
TCMACGSLSVSGARTLQ*FFYWD*A*RHRYAGVYRCAVSTACGGYAGTD*LLSGYR*KHDGPL*YCAGSENRSRSCI*LGTVSCAGQQGDNM
>NC_000913.3_cds_NP_414653.1_107_1 [gene=ampE] [protein=ampicillin resistance inner membrane
protein; putative signaling protein in beta-lactamase regulation] [protein_id=NP_414653.1]
[location=119281..120135]
MTLFTTLLVLIFERLFKLGEHWQLDHRLEAFFR-TGETFFSRAHVRHDHYCDGRDFFTVTRIAGSIVQRSHATGVAADWFAVYWRR*SSSSLS
CLSDSCFT***PCPCHDGWRTHHDSRRPGRLRRT*VFA*AAKCIAVD*LSFLSCTAVLADCGGNLGTRYADGVCVFACMAILAGAISDAASSF
TVRH*CRASCTGLGAGSSCGCGICLDRSW*ESVTGLVCFAG*FPYFAVSGVNASGAVLSGA*TACR*GGDAEGSGFNGEENLVRGRGGDCTTD
DLRGVGV
>NC_000913.3_cds_NP_414654.1_108_1 [gene=aroP] [protein=aromatic amino acid transporter]
[protein_id=NP_414654.1] [location=complement(120178..121551)]
MMEGQQHGEQLKRGLKNRHIQLIALGGAIGTGL-IPG*RLRNTVRRARDYPGLRHCWFYRLSDHASAG*NGGRRTCRRLL*PLCL*ILGQFCR
FRLWLELLGTVRFSCHG*ADCRG*IHSVLVSGNPHLGFCRRILCGD*RHQPDQR*SVWRDGVLVCHYQSYRGGSDDHLRRLAAIQWQRRPAGD
R*QPVGSGWFPAARLHRAGDDDGDYHVLVRWSGTGGDHRSRS**PGAKYTESN*PGYLPHPDFLYWFVSRSALTDAVDPRYRRYQSVCADLPR
```

VRRYLCGECAEHRGTDCGALRVQQLRILQQPYAVWSGTTG*CAKSAGVCR*TWCTSKYHSGVCTGNGVVRTD*LPCPRVRFRTVNGAGGICTG
NQLGDD*PGAYEIPSRQAGTRRGNSLPCSALSAG*LDLPAVYGGGTGDYADDPRNGDFGIPDPGMADRVRYRLSV*RENRQSRKSAL
>NC_000913.3_cds_NP_414655.1_109_1 [gene=pdhR] [protein=pyruvate dehydrogenase complex repressor; autorepressor] [protein_id=NP_414655.1] [location=122092..122856]
MAYSKIRQPKLSDVIEQQLEFLILEGTLRPGEK-TPTGTRTGKTV*RLPSLLA*GDSTSRSEGLVASSPGWRHFCPEQPMAKLQRSAGGAALR
PS*VTV*LARNTTRPGRYRRLLRRAA*YR*RQGTHP*TPPRHRAGAAVWRSGRGIKRRTPVSDCRHRSGPQCGSASSAKVYGADVGPECPPEL
RIALFASRDAAAGE*SPHPHI*SDYGR*AGRSARSIASPSGLYRRNFARQKS*REPP*AFSASSGATKEL
>NC_000913.3_cds_NP_414656.1_110_1 [gene=aceE] [protein=pyruvate dehydrogenase, decarboxylase component E1, thiamine triphosphate-binding] [protein_id=NP_414656.1] [location=123017..125680]
MSERFPNDVDPIETRDWLQAIESVIREEGVERA-TVSDRPTAC*SPQRRCKRSRRHRYQQLHQHHPR*RTTGVSG*SGTGTPYSFSYPLERHH
DGAACVEKRPRTGRPYGVLPVFRNHL*CVL*PLLPCTQRAGWRRPGLLPGPHLPGRVRSCFPGRSSDSGAAG*LPSGSSRQWPLFLSAPETDA
GILAVPDRIYGSGSDWCYLPG*IPEISGTPWPERYL*TNRLRVPR*R*NGRTGIQRCDHHRYP*KTG*PGLRYQL*PAAS*RPGHR*RQDHQR
TGRHLRRCWLERDQSDVG*PLG*TAA*GYQR*TDPADERNR*RRLPDLQIERWCVRS*TLLR*IS*NRSTGCRLD*RADLGTEPWWSRSEENL
RCIQESAGNQRQSDSNPCSYH*RLRHGRRG*R*KHRAPG*ENEHGRCASYPRPFQCAGV*CRYRKTAVHHLPGRF*RAYLSARSASETARLSA
KPSAELHREA*AAEPARLRRAVGRAEQRDLYHYRFRSCSERDAEEQVDQRSSGTDHRRRSAYFRYGRSVPSDWYLQPERSAVHPAGPRAGCLL
*RRRERSDSAGRDQRAGRRLFLAGSGDLLQHQQSADDPVLHLLLDVRLPAYWRSVLGGWRPASAWLPDRRYFRSYHPERRRSAARRWSQPHSV
ADYPELYLLRPGLRLRSCCHHA*RSGAYVR*KTRERLLLHHYAERKLPHAGNAGRC*GRYP*RYLQTRNY*R*QR*SSAARLRFYPASRP*SS
*DPGERLRRRF*RL*RDLLHRAGA*WSGL*TLEHAAPAGNSARSVYRSGDERRSGSGIYRLYETVR*AGPYLRTG*RLPRTGY*WLRSFRQP*
EPASPLRS*CFLCRGCGAGRTG*TWRNR*ESGC*RNRQIQHRCR*S*PASGV
>NC_000913.3_cds_NP_414657.1_111_1 [gene=aceF] [protein=pyruvate dehydrogenase, dihydrolipoyltransacetylase component E2] [protein_id=NP_414657.1] [location=125695..127587]
MAIEIKVPDIGADEVEITEILVKVGDKVEAEQS-TDHRRRRQSLYGSSVSAGGYR*RDQSLCWR*NPDRRTDYDFRFRRRCSRRCTCSGRREE
RSSSGSSTSGCGGKRR*RSGYRQRRS*SDRNPGESWR*S*S*TVADHRRRRQGFYGSSGSVCWHRERDQSERG*QSVYRLADYGLRSRG*SRR
GSSGR*TGSSSGSGPCTSGWRERS*RSGYRR*RS*SD*SDGESGRQSCR*TVTDHRRRRQSFYGSSGAVCRRREGTESQRWR*SENWLADYDL
RS*RRSACGSSCETGSGSAGTGSKS*SPGSSTSCESGRQI*IC*KRRLCSRDSADPPSGTRVWC*PCESEGHWP*RSYPARRRSGLRERSYQT
CRSSSGSDWRWYPWHAAVAEGGLQQVW*NRRSGTGPHPENLWCEPEP*LGNDPACYSLRQNRYHRVGSVP*TAERRSGET*AGCEDHPGCLHH
ESRCCSS*ADASLQ*FAVGRRSASDPEEIHQHRCGGGYPERSGCSGIQRRQQERHHRAVSRADDYF*ESA*R*ADCGRNAGRLLHHLQHRRPG
YYPLRADCERAGSGYPRRFQVRDGAGVEW*RVRAASDAADFSLLRPPRDRRC*WCPFHYHH*QHAV*HSPSGDV
>NC_000913.3_cds_NP_414658.1_112_1 [gene=lpd] [protein=dihydrolipoyl dehydrogenase; E3 component of pyruvate and 2-oxoglutarate dehydrogenases complexes; glycine cleavage system L p] [protein_id=NP_414658.1] [location=127912..129336]
MSTEIKTQVVVLGAGPAGYSAAFRCADLGLETV-NRRTLQHPWRCLPERRLYPF*STAARSKSYRRSQSAG*TRYRLRRTENRYRQDSYLERE
SDQSADRWSGWYGERPQSQSGQRSG*IHRG*HPGS*R*ERQNRDQLRQRDHCSGFSPDPTAVYSA*RSAYLGLH*RAGTERSTRTPAGNGWRY
HRSGNGHRLPRAGFTD*RG*NVRPGYPGS*QRHR*SLHQAYQQEIQPDAGNQSYRR*SERRRHLCDDGRQKSTR*TAALRRRAGSDWSCAER*
KPRRRQSRRGS*RPWFHPR*QTAAYQRTAHLCYRRYRRSTDAGTQRCSRRSRCR*SYRR*ETLLRSESYPVHRLYRTRSCMGGSD*ERSERER
HQL*NRHLPVGCFWSCYRFRLRRRYDQADFRQRISPCDRWCDCRY*RRRAAG*NRPGNRNGL*C*RHRTDHPRAPDSARVCGPGGRSVRR*HY
RPAEPESEEEV
>NC_000913.3_cds_NP_414659.1_113_1 [gene=yacH] [protein=DUF3300 family protein] [protein_id=NP_414659.1] [location=complement(129407..131260)]
MKMTLPFKPHVLALICSAGLCAASTGLYIKSRT-SGSACGTAIDTTGCV*RCRGYVSCNGFRTSRNTRRRQIRIQHCTNRSMGRARRAVSRRP
TFAGADGINLSDKRCSSSAMVAR*ST*TRRCCYSGGI*PAVGRQR*ITGGLSTIDGIDGRKPAMGAKPGRCFSGPAAGRDGLGTAIAATGATN
RLAEVINRTESYYHNEESCTGKTDSHGTRHTIQYSFNCQPRHYRAGNNRHFH*ARQS*CGLYSQLQPNRGLRELGQYCVSAGLSATTSRRTVC
*QLCTRIRL*HGRCYHVRTIQQHRLGRRRS*PSSS*Q**LSSPRWRSS*R*WLATQRRQHQYRRQQFQPYHR*ASY**EYGMAAQSKLP*WCA
LS*SGYGKAVSSNRCQRRNERHAITCPNARQPASGGSKSVSATNTRRTSHYTRYPTSGSGTAV**S*TLWEL*RLPRLQPSPTTDPATKGRRS
SALSVRLA*AAPGSSRENAD*PEDPAAKRGSA*AHSVRLA*AAPGGSRENAD*PAEPAAKRRSA*AYSVRITRAAPGV*GKSTAAPTESTAT*
*RPPACSIGIT*TTSGFSGESSGEPPTTSKRQ*PYCQAE*RATVSSMRTSL*TWSKKTGKV
>NC_000913.3_cds_NP_414660.1_114_1 [gene=acnB] [protein=aconitate hydratase 2; aconitase B; 2-methyl-cis-aconitate hydratase] [protein_id=NP_414660.1] [location=131615..134212]
VLEEYRKHVAERAAEGIAPKPLDANQMAALVEL-TEKPARGRRRIPVRSVNQPCSPRRR*SRLCQSRLPGCYRERRSQIPSADSGKSHRTAGH
HAGWLQHSSADRRAG*CQTGTYCCQSTFSHAADVR*LL*RRRESESRQRICEAGYAVLGGCRMVPESPGAG*KTDRYCLQSHWRN*HR*PFSG
TGCVVTPGYPTARAGDAEKRP*RY*ARPAWCCWSDQANRSSATERFPAGVRR*RCGYGFFA*IRH*LRSVVYGR*YSTCAEQTRRWFVPRR*N
CTHLL*HDGRRGCTANRSRRL*PEHGRRD*RLPVQR*SA*PRNRRTAGDLRTENRRAD**SACWWPYSADYRAWPDHQSA*STWSAAQ*CVPS
GERCR*ERSRLLAGAKNGRPCLWRERHSSGRVL*TENDFCRFPGHHRPDDP**TERPGVPGLLG*PGDAVFLPHRGVSEAS*REHAPHAAGLH
YEPWRCVAASG*RRHSLLAEPYAAAGYRRYRW*LPYPFPDRYLFPGGFWSGGVCCRNWRNAA*YAGIRSGALQRQNAAGHHPARSGTRYSAVC
DQTRSADR*EERQEKHLLWPHPGN*RSAGSES*AGL*ANRCVRRAFCRWLYHQAEQRTDHRIPEL*HRPAEVDDRGRLRRSSYPGTSYSGHGK
MAGES*AAGSRCRCGIRGSDRHRSGGY*RANPVCSERPG*RASAVCGTG*EDRRSVYRFLHDQHRSLPCCG*TAGCA*RSVADPPVGGTANPY
GRRTVDRRRLLQRLR*EWCAYRDPWLFPVYG*PGACGGRCNGGFHLYP*LPEPSGYWRECLPGFCGTGGCCGADWQTADAGRVPDLRGAGR*N
SR*YLPLSELQPAFSVHRESRWGDFPDCGL
>NC_000913.3_cds_NP_414661.2_115_1 [gene=yacL] [protein=UPF0231 family protein] [protein_id=NP_414661.2] [location=134388..134750]
MDYEFLRDITGVVKVRMSMGHEVVGHWFNEEVK-RKPGLA**SGTSSARTER*RTVLATGRA*IHPVDGR*RGDGSRQSTGIRWR*NGRGDEL
LRRRKPVAMRR*GFSAGRGGLPQFRAAEV
>NC_000913.3_cds_NP_414662.1_116_1 [gene=speD] [protein=S-adenosylmethionine decarboxylase] [protein_id=NP_414662.1] [location=complement(134788..135582)]
LKKLKLHGFNNLTKSLSFCIYDICYAKTAEERD-RLYCLYR*TL*CQPSDRNPVRNLFHYRG*YS*HRPPGLRTTGCQRHYSGE*RTG*PETH
RQNRTPRPTARNGRCPS**KSYLRTYLPGKSS*RRFMYLPRRY*SLYLRRDFSAEGAELPDPPA*VRYRNH*LSRARFYPRH*RYEALYRP*D
*FDSELYV*RYEGAV*HGGCERLSGKYLPYQDVA*RVRP*ALHVPHQTGRLNRQRAPGNYRCAVERNARDLLRAQYASCL

>NC_000913.3_cds_NP_414663.1_117_1 [gene=speE] [protein=spermidine synthase (putrescine aminopropyltransferase)] [protein_id=NP_414663.1] [location=complement(135598..136464)]
MAEKKQWHETLHDQFGQYFAVDNVLYHEKTDHQ-RSDHF*ERCIWSRNGAGWRSTNHRARRVYLS*DDDPCSATGPWSRETCADYRRRRRCHA
A*SNPT*KR*VNHDGGNRCGCRIVLPSVSTQP*RR*LRRSAL*AGDRRWRQFR*SNQPDL*CHYLRLHRSYRSRRKPFHFGIL*RLQTLPESW
RYLRRTKRRLLFTAGRSHRQPSQTQPLLQRRWLLSGGDPDLLRRYHDFCMGDR*RRLTPSLNRNYSGAFSRLWPEMPLLQSGNPYGSFCLTSV
SARRTGFTAVL
>NC_000913.3_cds_NP_414664.4_118_1 [gene=yacC] [protein=PulS_OutS family protein] [protein_id=NP_414664.4] [location=complement(136570..136917)]
MKTFFRTVLFGSLMAVCANSYALSESEAEDMAD-INGSFCLSEERLWLPELT*RANSSRTGLFRSAKPVGPQ*LRHLRHESPR*RQLPRSQRH
WHSRR*KMQSPGPRFLKPACLRQI
>NC_000913.3_cds_NP_414665.1_119_1 [gene=cueO] [protein=multicopper oxidase (laccase)] [protein_id=NP_414665.1] [location=137083..138633]
MQRRDFLKYSVALGVASALPLWSRAVFAAERPT-ITDP*FAHDRCP*SHSVNYWRRPVHLWRENCNYLGL*RQSAGAGGEITARQSGNG*YLQ
PTDGRDNVALARAGSTG*SRRRPAGNYSARWQALGDVER*STCRYLLVPSASARQNRATGGDGAGWAGGD*R*RDPEINAAKTVGYR*CSGDR
SG*EI*RRRAD*LSTGCDDRRRGLVWRYVADQRCNLPATRCPAWLAAPAFAQWL*CPFAQFRHQRQSPAVCDCQRRWSAT*TSEGERTAGADG
RAF*SAGGG*R*QTL*PGDAAGQPDGDGDCAV**ASSGNADSADCY*CLRCFARHIK*PACVTFAGRADGTQAATLYGPDARYDGDADANGEI
WRSGDGRDGSQPDDGPYGARQYESYEPRREVRFPPCQQNQRSGV*YEQADVCGGERAIRTLGYLWRGRHDAASVPYPRHAVPYLVRKWQTASG
SSRGLERYR*GRR*CQRSAGEV*SRCTERTCLYGALPSAGA*RYGDDVRVYGI
>NC_000913.3_cds_NP_414666.1_120_1 [gene=gcd] [protein=glucose dehydrogenase] [protein_id=NP_414666.1] [location=complement(138835..141225)]
MAINNTGSRRLLVTLTALFAALCGLYLLIGGGW-TGRDWRLLVLPYRWPCDARRRLDAVAQ*TRRALAIRSPAARHHDLGRLGSWFRLLGADS
AQRHSGLLRHLADPAVCLASPGHSCQRRSCRTGGRTAD*RWYPDLGRI*RSAGDQRHLKRRCHTC*SYLPRSRSGLACLWS*SGRSTLFAAET
N*RR*RP*SERSLGVPYWRCEAAERSG*NHQ*SDAD*SGRHPLPVYRSPAPVCA*CRQRQREMALRS*AENQRVFPARNLPWCLLS*SQSRNR
FAGSDGGLPASYHSSGQ*WSTDCD*R*KRQTVRNLRQ*RRAQSAKQYARHQTGSV*TDFATDYHR*NHRDGRFSYR*LLNPRNVWRDPWF*CQ
HRGAAVGF*SRRERSERNPV*RTHLYL*LAKLLGTSGL*REAGSGLSADGRDHAGYLGR*PHTGTGTLCQLDSGAECHYRETGVELPDRSPRP
VGHGSSGTADAGGHHR*WSESASYLRSGENRQHFCARSS*WRTGGSGTGKTGSPRCSERRLRNPNSTVF*TELPSDERFERCGYVGSHHV*PT
GVPRDVPPDAL*RHFHPAI*TGYAGLPG*PGDVRMGRDFR*SKS*SGDCQPNGTAVCFETDPAWSWQPDGAAERCQRHGYGIRHSATVRCTVW
CHAQPVPLTIWSAM*TASMGLYLGAGSEN**SGVEETYWYAAGQYAVPDAGSGAVQYGYADAGRANLHGG*RAVYRRYGR*LPARLQHEQR*K
TVAGSFTSGWSGYANDL*SEW*AVCGDLRRRSRFIWYEDGRLYCGLCAAG*CEV
>NC_000913.3_cds_NP_414667.4_121_1 [gene=hpt] [protein=hypoxanthine phosphoribosyltransferase] [protein_id=NP_414667.4] [location=141431..141967]
MKHTVEVMIPEAEIKARIAELGRQITERYKDSG-KRYGAGGSAAWLIYVYGGPVP*SSGIS*SRLYDRLQLR*RHVHHP*CENPQRSG*RYPW
QGRADC*RYHRLGEYTVESA*DLKPARTEVAGDLYAAG*TVPS*SERPGRIYRFLDPG*VCGGLRH*LRTALPSSAVYRQSDSAGRV
>NC_000913.3_cds_NP_414668.1_122_1 [gene=can] [protein=carbonic anhydrase] [protein_id=NP_414668.1] [location=complement(142008..142670)]
MKDIDTLISNNALWSKMLVEEDPGFFEKLAQAQ-KTALSMDWMFRQSRSCRTFNRS*AGRTLCSP*CC*PGHSH*PELPFRGSVCSGCTRS*T
HYYLWPLRLRRRTSRS*KPGTGAYQQLAAAYPRYLVQT*LIARRNAARAPSGYLV*TERHGTGV*PGPLHHYAISVETRAESYHSRLGLRHSR
RLAA*SGCYRHQPRNP*ATLPSRDFQPQAETRQPQI
>NC_000913.3_cds_NP_414669.1_123_1 [gene=yadG] [protein=putative ABC transporter ATPase] [protein_id=NP_414669.1] [location=142779..143705]
MTIALELQQLKKTYPGGVQALRGIDLQVEAGDF-ICASRAERGREIDHYRYYQLSGK*NLRAGQRIWLRSREGCRER*TSVGTGAAGI*LQPV
*NRAANCGESGRVLRRGAQRSVHPQRKVS*TTRSMGKTQRTCAYVIWRDEAPFNDCPCVNA*T*TTDSRRTDRRRGY*TSPLNVGLFEGFKRQ
RHHHHSHHTLPGRSRNAVPQYRHYSTR*AGGKYLDEGAAGEAEIGNLYSRSRTEKPVTEARWLSVSTGRYRDAGS*SAA*AGDQQRIYAVK*A
GHSGIKYA*QS*PSGRAVCFTG**KTRRSRM
>NC_000913.3_cds_NP_414670.1_124_1 [gene=yadH] [protein=putative ABC transporter permease] [protein_id=NP_414670.1] [location=143702..144472]
MMHLYWVALKSIWAKEIHRFMRIWVQTLVPPVI-NHDPLLYYLR*PDWFAYWRYAWLQLYAVHRTGADHDVGDHQCLRQRCVIIFWCQVPA*Y
*RAAGSAGSDSRHYCRICRRWRGAWSVCWHSGDGNFTVFCAISGAFVGIRCLNAGAHGGVVLPCGFAERCVCQNVR*HQPGANLCVNATHVFG
RGLLLTDFVAAVLARAVAPEPNRLYDQWFPLRLPRYQ*CSAGHYLWRTGGLYCGVLFDLLVADPTWTWFA*L
>NC_000913.3_cds_NP_414671.1_125_1 [gene=yadI] [protein=putative PTS Enzyme IIA] [protein_id=NP_414671.1] [location=144577..145017]
MLGWVITCHDDRAQEILDALEKKHGALLQCRAV-KFLARIKL*YAQPHDVRCSA*SGLW*GCHLLNRYSRSATVSRGFIIKPQTLPLRSDFWC
HVTVN*TDDGLP*NHDQFRVSRAYCRTGCAGGE*SLAPTTKKSAFRPQT*FV*VL
>NC_000913.3_cds_NP_414672.1_126_1 [gene=yadE] [protein=putative polysaccharide deacetylase lipoprotein] [protein_id=NP_414672.1] [location=145081..146310]
MYKQAVILLLMLFTASVSAALPARYMQTIENAA-SLGANW*QDGDRGEYSGRTNHCRGAHCRKLLRI*FWLWQRFYR*RSSRAGSGATKS*RR
FGRPQQAAE*SELSYLERYAGL*RAECGKCAIWGTGGQFALPDFA*TERQVKSNLVSDPYWRSTGLYQRTGCPTR*WPVGANLSPYSARRRKH
PFSPYFDDHIGTRFQ*PDGLAA*QGIRDTEHGAAGRLREE*DQSPCASGGDYL**WPQVGEPLCVSCVETIWHEGDGVYCYLTHQTSPAEVEP
KIAAIYERF*A*RNSRCI*FPVTYPFFASGRWLSPTHITEP**AQYSV*FCTFTPRSGAI*SACLVSFVSVWRI**QRREGSKRCRISPGGDN
HERQSKTGG*SVVTKTTLYLKNGFAGDDVAAGE*PAAGI
>NC_000913.3_cds_NP_414673.1_127_1 [gene=panD] [protein=aspartate 1-decarboxylase] [protein_id=NP_414673.1] [location=complement(146314..146694)]
MIRTMLQGKLHRVKVTHADLHYEGSCAIDQDFL-RRSRYSRKRSH*YLECHQRQAFLHLCHRGRTRFENYFC*RCGGPLRQCRRYCHHRQLRY
HAR*RSSHLATQRRLF*RRQ*NETYRESDSGTGCL
>NC_000913.3_cds_NP_414674.1_128_1 [gene=yadD] [protein=transposase_31 family protein] [protein_id=NP_414674.1] [location=146968..147870]
MDAPSTTPHDAVFKQFLMHAETARDFLEIHLPV-RITRTL*PQHASFRVGEFH*REPERTQHGRALFRANAGQSRLSACCD*TPKQAG*ENGL
SHDALFYSRHAPASGG*PR*AAAGGADTVLSGRGHTLSAINVLV*YVLLAGAGATRL*QSFPAGGYHHHTG*RNHATSADCDSRTTAKTYSPA

RLNVIA*ATGHADRRRVH*RKSVSCHAKLYAATRSY*TSGFVLRCVERQGNGRGVYDDAGAVV*RERD*EGDSAGKTGSKSGIRPASSE*RNV
SGRRCRDGKFTSC*D**GN*PYL
>NC_000913.3_cds_NP_414675.1_129_1 [gene=panC] [protein=pantothenate synthetase]
[protein_id=NP_414675.1] [location=complement(147944..148795)]
VLIIETLPLLRQQIRRLRMEGKRVALVPTMGNL-TRWPYEAGRRSQSPRRCGRRQYFR*PDAVRPPGRSGSLSTDLAGGLREAKQT*SGFSFR
PFGKRDLPERY*NPHLR*RSWPFDHAGRCQPSGTFSRRFDYCQQAVQPGPAGHRLLR*KRFSATGADPQNGCRYGLRY*DCRCANYARQRRSG
AKFP*RLSDGGTTQNCAWSVQSFKFDC*QIAGWGTGSR*NYYHCGARTE*KRLPRR*YSDSRCRHIAGSF*NQQTGSNSGSRLAWRCSPDRQQ
NGRAGV
>NC_000913.3_cds_NP_414676.1_130_1 [gene=panB] [protein=3-methyl-2-oxobutanoate
hydroxymethyltransferase] [protein_id=NP_414676.1] [location=complement(148807..149601)]
MKPTTISLLQKYKQEKKRFATITAYDYSFAKLF-S**RA*RHAGGRFAGHDGSGARLHPASYRCRYRLPHCRRTSRRTKLPAAG*PAVYGVCH
AGTSLRKRRNGYACRC*HGQN*RR*VAGRNRTNADRTCRSCMWSLRFNTTVSEYFRWLQSSGARR*SGRSTAQRCISLRSCWGTAAGAGMRAG
*TGKTYYRSTGDPGYWHWRRQRH*RADPRDARRLWYYRRSHS*IR*KFPRRNGRHPRGCAAVYG*SGVRRLSGRRTQFPL
>NC_000913.3_cds_NP_414677.1_131_1 [gene=yadC] [protein=putative fimbrial-like adhesin
protein] [protein_id=NP_414677.1] [location=complement(149715..150953)]
MKTIFRYILFLALYSCCNTVSAYTSFIVGNNAG-S**LSRPLHCRTDDL*LHINSKQLGFL*THAARPDWGKNVLVIPGYRYRWWYSLLQYIW
QSESWSNNY*KCHGLFR*RLWRT*TI*YICSWSVLHHVNIKGLVCIRYNN*HSIARNLYRRSFQPRIFLFRHRQRSTN*GLQQSRRLR*VLGY
WWYSTQHNC*ILYRY*FRSYT*SASPVIQFIKLPLFV*GLQSWYKSCRSQQPHLCQFHTE*CQINLTNMFYLHTYRAISQWFNG*NGRI*LWD
N*KWRFTCSL*YLASELYSCA*Y*NKTRHWKSRYSKHTTAW*YAYWKHCRQRRWRTH*RFSNQ*KSANDIKT**YEFCLYRL*NRR*HLRWGL
P*SR*RHITASSFPGNIKARREYCYRTRRI*SHQYFPGNLSL
>NC_000913.3_cds_NP_414678.1_132_1 [gene=yadK] [protein=putative fimbrial-like adhesin
protein] [protein_id=NP_414678.1] [location=complement(151003..151599)]
MHPTQRKLMKRIILFLSLLFCIACPAIAGQDID-TCCQCKKQHLQKRNQ*PG*Y*SWRRWGGIFFR*CYS*KLSTRWKRVHYHCIRLCITGNW
RCAKSVTY*F*SP*RCHGCWL*ANIC**NFVRSK*CRSSYIFYSGFGEYIQCS*CFRRISFRLSSNVG*HEWFILEI*HPNAKNRSCIECYIW
STYEPCVSGYLLRI
>NC_000913.3_cds_NP_414679.1_133_1 [gene=yadL] [protein=putative fimbrial-like adhesin
protein] [protein_id=NP_414679.1] [location=complement(151626..152231)]
MMTFKNLRYGLSSSVVLAASLFSVLSYAATDSI-RTDRYYYCRNGYLYRYISK*L*SGHFCC*FW*CIYF*NQCQDQSKNIQTQIQRLCGYPQ
*KSANKINQASHMRGNC**RCGVCKWFHSRR*SKCCRR*SLEHCNSGNRECNTI*LCNTSITRGNNLHCSQCGRLLSDECTPGRGKK*NRKQC
HCG*VFCTSHIYSNL*L
>NC_000913.3_cds_NP_414680.4_134_1 [gene=yadM] [protein=putative fimbrial-like adhesin
protein] [protein_id=NP_414680.4] [location=complement(152243..152812)]
MIKTTPHKIVILMGILLSPSVFATDINVEFTAT-SQSDNL*HHTYW**RHE*WQ**LHIENP*DGSG*DRE*NDRISG*F*TGCQWVQQWHQL
D*YHSDRKCIIKLT*AYYTAVW*FIFDDK*YRYGFQKTDY**CHFP*T*QCGKDTLEHRRDAAR*GS*NDRCAT*NRCRARRTGEFSCTGDV*
FHLSI
>NC_000913.3_cds_NP_414681.1_135_1 [gene=htrE] [protein=putative outer membrane usher
protein] [protein_id=NP_414681.1] [location=complement(152829..155426)]
VTIEYTKNYHHLTRIATFCALLYCNTAFSAELV-RI*PYLPDGAECI*Y*SQPVQ*R*PRYTGCLRRQCLCKRPTNH*PKYYICRN*RKKERP
GLYHIKEFIAVSY*FSRYK*RKSRSACQG*NARQLPQFDGNYPSGFCSL*R*RSTSGYRRSSSLGNEKLPKLC*SIVMGKRH*CGHVVIQPQR
IS**NPWSKK*KHLCCI*RWDEFRCMATACLGQLQLDDRFWQ*L*F*ESVCSA*YRLAAFSTHSW*VLYDGRNL*FRQYPRHSFIQ*QPHVAS
DFSQLCAYHSWRCQYQRQSHYYARWL*DL*NDGAARRFRH**SESVRVRQRSYCYHRRIRWLKADILATFLIRCSNVTPWRWTLGY*RRSGLK
R*YSG*A*FISSKLLLRPE*LSDGLYRYSDNR**LYRWFVRSWSEYFSWCIFFRCDSFQCSYPG**NIPGAKLSCFLEQVIRRNKYFTEYRGL
SLFDTELPWS**CTNSN**SETSRTRS*TEIHA*LLTHEKSGYGQY*PTVEI*EKRLRFILSFRKLVRLLGFRTKS*QLLYWLQ*QYILGQLQ
CQCPAFME*RRRH*R*RLS*FHHSN*KITWH*TTYFRFPEY*YSNKQ*L*G**PTQR*QQWL*R*RSRQL*REYWLYDE*SQQRFELCWGLCQ
L*VTMGNAGRFNFCK*R*QPSSFSQHRRWFCIA*RWTDFQ***F*RLRYTGGSSGSRCSRSANKLWQQYYRSMGLWCHQRSFSLS*KPYRAGY
QRS*ERC*IKKYQCSSCTASGFSRLC*F*NRARAISHYEHHTK*W*KYSICCRYL**ARQCHW*CWTGWTSICSWY*AAGKYQH*MARTK*TR
KLSCALSTKPRSRKNSTIYYSEWNSVSDSV
>NC_000913.3_cds_NP_414682.1_136_1 [gene=yadV] [protein=putative periplasmic pilin chaperone]
[protein_id=NP_414682.1] [location=complement(155461..156201)]
MFFNTKHTTALCFVTCMAFSSSSIADIVISGTR-SNI*KRSKKCQRTSGK*RE*PVACPELVRYWR*QR*AWQYYSPFYCYAASIAY*CQTWA
NNQINVHSQHLTA*RQRERVLV*RTGSSTKTRCRKGRESKPAATGISHTYKTFLSPGWIEGKSL*SPVSPEVVLVRFRR*GVITSDQSNPLLR
LF*QW*FRS*R*TLSD*CENDCTI***GHESQWP*WQSEFCKSAFLRH**LWWRN*R*CQAV
>NC_000913.3_cds_NP_414683.1_137_1 [gene=yadN] [protein=putative fimbrial-like adhesin
protein] [protein_id=NP_414683.1] [location=complement(156299..156883)]
MSKKLGFALSGLMLAMVAGTASADMDGGQLNIS-RSGC**HL*NPR*RR*QGWSDPAANRNRW*NRCWRTE*HRWR*S*TFQHHR*LQQSKSE
SR*HG*NDLRFCFLW*QQRDFEQRHVY*QPI*RR*YRSTQY*WFHYQTGSN*QPWRCVY*SPGCNDKICCL*F*SVLRSCRRRPNSNCWLCKN
*HCIHHYLSV
>NC_000913.3_cds_NP_414684.1_138_1 [gene=folK] [protein=2-amino-4-hydroxy-6-
hydroxymethyldihyropteridine pyrophosphokinase] [protein_id=NP_414684.1]
[location=complement(157253..157732)]
MTVAYIAIGSNLASPLEQVNAALKALGDIPESH-NSYRFFVLPHPTAGAARSTRLLKRSRGAGNLSCT*RATQSHTAY*IAARSRPQS*TLGT
THAGSRHHAVW**SDKY*TPDRSALRYEESWIYAVAAV*NRAGVGVS*WGDVASNLTYKSI*QIKQMV
>NC_000913.3_cds_NP_414685.4_139_1 [gene=pcnB] [protein=poly(A) polymerase]
[protein_id=NP_414685.4] [location=complement(157729..159126)]
IFTRVANFCRKVLSREESEAEQAVARPQVTVIP-T*AACYFPQRYQ*KCPEGNVQAQ*SGIRSLAGWRRRARPVTWQKAERF*RNH*RHA*AG
AQTVP*LPPGGSPFPSGSCNVWPGDYRSCDLPWTPRR*RQRPHDLPTRAKRHVAARQHFRLHRRRPAPRFHYQQPVLQRSGFYRP*LRWRHE
GSEGRRYPSDW*PGNALP*RSGTYAARGTFCRQIGYAHQPGNRRTDPSPRYPAERYPTGTPV*RIA*TATSGLRLRNL*AVV*ISSVPAAVPD
HYPLLHGKWRQPDGADH*TGAEEYRYAYP*RYAREPGVPVCRHVLVPTAGDGTEDRPGKRPDLSRRFRAGDERRAGRSLPFTGNPETSDDINP

RYLAVAVAYVPSSG*TRMETAGAS*VPCGL*PVGLAS*S*A*R*TAASGEMVG*VPGFRATRPKRDAQRAG*RTVTASSYSSSTQTRTTS*GY
RM
>NC_000913.3_cds_NP_414686.3_140_1 [gene=gluQ] [protein=glutamyl-Q tRNA(Asp) synthetase]
[protein_id=NP_414686.3] [location=complement(159186..160112)]
MLPPYFLFKEMTDTQYIGRFAPSPSGELHFGSL-NRRARQLFAGSRPARSLAGTHRRYRPAS*SSRCRRNYPAPAGTLRSALGRRCSLAIATS
RRLS*STRLVT*TRTKLLLHLYACAYSKHWRYLRRSLPGVASWTRQRRSAYPPAASGHAIY*PAARHYSRRRKTGTGRFYHSSP*WVVRLQPG
CCG**SFPGRYRNSAWG*SD*TNSKANLAVPAFWLESARLHSSAAGA*STRR*TFQAESCACVAERRSTPGTNRGTSISGAAGRSTLAGFQRR
ANPSVSRQKLAAYRRARVGNCKFNILKCVML
>NC_000913.3_cds_NP_414687.1_141_1 [gene=dksA] [protein=transcriptional regulator of rRNA
transcription; DnaK suppressor protein] [protein_id=NP_414687.1]
[location=complement(160149..160604)]
MQEGQNRKTSSLSILAIAGVEPYQEKPGEEYMN-RSPAGALPSYSGSMA*STQG*SRSHRYTYAG*SSQLPGPGRPCSPGRRVQPRTA*PRSR
A*ADQKDREDAEKSGRRRFRLLRILRC*NWYSPSGSAPDSRSVHRLQNAG*NSRKTDGWL
>NC_000913.3_cds_NP_414688.1_142_1 [gene=sfsA] [protein=sugar fermentation stimulation
protein A] [protein_id=NP_414688.1] [location=complement(160782..161486)]
MEFSPPLQRATLIQRYKRFLADVITPDGRELTL-TLPEYGCDDRLCNAWRYRLVFDFRQHQTEIPTHLGINSKPERRIYLRQHALG*QVDERG
YP**INFRTVRL*LAEKRSKIRRRTQPY*LYVAGGFASRLLY*SEIGYVSGERTGIFSRCGH*TRSETPSGVDERSG*RPACGYLFRRAAFSH
YTVFTRAPHR*EIRATIVRSSTEGGRNSGLQSGNFC*RHGS*KITAGYIV
>NC_000913.3_cds_NP_414689.4_143_1 [gene=ligT] [protein=2*-5* RNA ligase]
[protein_id=NP_414689.4] [location=complement(161501..162031)]
MSEPQRLFFAIDLPAEIREQIIHWRATHFPPEA-RTSGRRR*FASDSGIFRRSERREREGAFSFSRTDSSTWFHTHA**RRTMAAFACGVVRD
ASAATRLNPAGEYAPFTGCPQRLFSKQSSVSSTYYLIARRQRGGDNPAARF*LVVCGDGVHPLRLLVCPWTHTLHAAKTLGANAI
>NC_000913.3_cds_NP_414690.4_144_1 [gene=hrpB] [protein=putative ATP-dependent helicase]
[protein_id=NP_414690.4] [location=162105..164534]
VSSLPVAAVLPELLTALDCAPQVLLSAPTGAGK-INLAAAATAGASRH*RENYPAGAASSGGA*RRATAGGAA*RKARRYRWLPDACAKLRRA
EYPPGSGYRRRADAHDPA*PGTERCWTGDP**IS*AQLAGGFGVGAVTRCATRSA**P*TADYVGYPGQRPLAANAARSACRHLRRALVSG*T
PLFTAARASAF*RCRCGSHR*NAASGKRIITVIFTWRRRNSACAGTTGFAHRQ*CIALPAVWRVVAERSAKSDPPGTARDAQSGAGDQYC*NQ
FNH*RYSSGGGLCPGACGAF*SAHGAYATDYSTR*PGIHDAACRARRASGAGYQPAFNRQRTSRTRRGAK*TGDLTKRSFRFADGITAMGMQR
SGADELAGSTASSESTGRETSVTNAGGTGG*TA*CARAKNGSAG*RSAFSGNAG*REERRRSCYRGKNCRHSRRAATDGQ**PGRGVFAQSTS
LAAT*SATVKTLKRTWR*GRQFAYRAATCRGVCRSHCSSPWARWTLSTGKRHGSDARCQRRAKPPRMVDRTVIIAGQRLAGCADFTGAAGRY*
*VSTTLPAAGTAV*HCGVG*RARYAESLASATNRSVDGESAAAGETVRRRVASGDA*WHP**RFKRAQLDGGSGTATLAFVMRRKVVAGI*LA
SG***KFIGSAGNVAAATYDWRTFTTRPEITRHLSGTTRIT*LGNAATSG**IACALHCADGKPDRHSLS*R*PARAGGENARDVWRGHQSDD
RPGARAAGAGVAFTCPKAITNHTRFERLLERSVP*GAKRDERALSQTCLAGRPGKYCTDATDEKVFV
>NC_000913.3_cds_NP_414691.1_145_1 [gene=mrcB] [protein=fused glycosyl transferase and
transpeptidase] [protein_id=NP_414691.1] [location=164730..167264]
MAGNDREPIGRKGKPTRPVKQKVSRRRYEDDDD-IRRL**L*G*RTDAAQR*GQRQRA*ASWQTRLAMATAKTGYRFCRADRHLRRLSRSKNS
*PY*WQGLATACGSLWPNGQS*ARHDHQQERDGEAAGGDPVSSGVENDPSWRIYRAGQQH*DDSPSV*FPGQ*RRTGARASDL*WRSSGDDRQ
YGEQPSVRFLPS*SASDHHDLFAKR*AASVCAAQWFPGFAGGYFAGDRRPSFLRA*WNQSLLNRTCGAGKPDRRTHGTGCEYADATAGEKPVP
LQRAFLLA*SERSLHGADHGRALQQRPYS*AVYERGVSRSERRQRNPRLPAGKLVLLWSPGRRAKPRPAGAVSRYGERGVHLQPVA*PKTGAG
AT*SGAASAATATDY*SRTL*HVECPSAGGSAARWGDLSSASLYATGASGAAGKTGR*GKRSLRREDLHYL*LGGPGRGRKSRRGRHSGTEET
A*VERS*NCDCGRRPL*W*SSCDGRRF*AAVCGLQPCDAGASFDWFPCKTSDLSDGLKPAENLSSEYVDCGCANCAASAEWPGLVTAE**PSL
*RKRQSDAGGCVDPFDERADGKSGDGAGAACGYGDLD*TGRTERSVASGSGNAAGGVELNANRSGAGIPDHRQRW*PCTAFCAAFGNRGRWQS
AVSELPAGGTRCSGAGGVSDTMDHAAGGTTRYGSSAWGEIPEPASGRENRDYQQ*RRYLVCGH*RQHGDHHLGRP**QPADQTVWCQRGNVDL
SALSG*PDANAAESCSARRYCRYGRGLRRQLCLQRWHAYLAGLDQRSAIAVPAERDAAAAVRQSV*SVFSAAATAATATCSARAERQRRCSRL
DQGYVW**L
>NC_000913.3_cds_NP_414692.1_146_1 [gene=fhuA] [protein=ferrichrome outer membrane
transporter] [protein_id=NP_414692.1] [location=167484..169727]
MARSKTAQPKHSLRKIAVVVATAVSGMSVYAQA-SG*TERRHYHRYRCTCAARKRMGACCNYCGATVCYRH*NRYADSKSATVYFCCDRRRDG
AASAEVGKRSA*LHAGCLCWYAWRIQHL*PPDHSRLCGRRPKPE*LSEWPEVAGQLL*RCGH*PVYAGTR*NYAWPGFRALR*KQSWRPVEYG
QQASDHRTAERSSV*SRY*QPVPDWF*L*RFVG**RCLLLSPDRSCAFCQCPAERVRRAALCYCTGVHLASG**NQFYLPFLLPERAGNRLLR
LVAERGNR*AAAER*ASADRL**RGEEQHLFS**EDGRLQLRSRI*RHLYCASEPALC*KQNLAKQRLWLRRLLRSGECLQQTVCGISASG*R
PLSGT*IRR***EAAKLLR*YPVAEQVCHWRYRPHPADRCRLYAYA**HQRLVWLRRLCATAQSVQSGEYRFRLQCQRSGKLRPLPHSE*TET
NGRLCSGSGAVG*SAGHPRRSL*LGRSRIS*PRCRDDR*T**QTVYLAWWC*LPV**WCNTLLQL*RIV*TFFASWEGW*YFRTV*R*AV*SR
REICTGRSSDCSYWCRV*SH*NQQPDGGP*GFLLLG*RWRDPRTWRRNRSESGAVGEC*RSRFLYLHRCGIHHRYYL*RQYACTGAKTHGFVV
G*LHLL*RSAFRSDAGHRWSLYWLQLW*SG*LL*SGKLYGRGCVSTL*SGASRHGWLQRGAAC*QPVRS*IRRQLL*HLWLLLGRRTSGRCNR
NLPFL
>NC_000913.3_cds_NP_414693.1_147_1 [gene=fhuC] [protein=iron(3+)-hydroxamate import ABC
transporter ATPase] [protein_id=NP_414693.1] [location=169778..170575]
MQEYTNHSDTTFALRNISFRVPGRTLLHPLSLT-ISCRESDRSDWSQRFW*IHSAQNAWPSSAAVGRGDSS*CPTAGKLEQQSVCPQSGLFAA
AASSGRRDDRA*TGGDWSLPVAWRAGALWGGRSRKSRGSYLAGWLKTAGASAGR*SLWRRTSAGVDRHAGGAG*PLSVARRTDLGAGYRPPG*
CAVAGAPFKSGAWPDGHCRVARYQYGGTLL*LSGRPARR*NDCSGNACGNYARRNPRNDLWHPDGYFAASGGCCTCEFCLL
>NC_000913.3_cds_NP_414694.1_148_1 [gene=fhuD] [protein=iron(3+)-hydroxamate import ABC
transporter periplasmic binding protein] [protein_id=NP_414694.1] [location=170575..171465]
MSGLPLISRRRLLTAMALSPLLWQMNTAHAAAI-RSQSYCGAGVVAGGITAGARHRALRRGGYHQLSPVGQRTTIAGLSDRRRFAHRT*P*TA
DRNETIVYGLVGRIWPFTRNAGSYCAGSRI*LQ*RQTAVGDGA*IADGNGRFT*PAKRSGNAFSAI*RLYPQHETPLCEAWCASVIADDAYRS
APYAGLRSKQLVPGNS**VRHPKCLARGNQLLGQYRRQYRSSGGV*RR*CALF*SRQQQRHGCANGNAAVAGHAVCPRRTLSARTCSLVLWCD
ALGNALCARSG*RHRR*SV

```
>NC_000913.3_cds_NP_414695.1_149_1 [gene=fhuB] [protein=iron(3+)-hydroxamate import ABC
transporter permease] [protein_id=NP_414695.1] [location=171462..173444]
VSKRIALFPALLLALLVIVATALTWMNFSQALP-T*PVGAGCLVAGY*RHRADDFSLQLVAASGDFAAGGRGSGAGGRAVSASAA*PAGGADD
AWRCYRRATGDYRHYALGDPWCDGEPVCCAGRGLCCWLNCLWRRVGETAVAGNADSRGVGSEPLLRRNQSVTGYLPS*PTAKHVSVEHWNADA
NRLGRR*AFMAAAAGRCDADVAATSSVNPDGA**WRGAQSRAGLVACASGSAVAGDCHQCAAGERCGDYRLYRVVRAAAGKNAGGAASAATTD
AGVVDWCADPLAFRSNHPLADSRVDGSVHRFGHCVDRCAAATVAVAAFTQH*RAGYEGQRSCRG*TPTCAGVCPRGRRAAVDGCGGGAVVWS*
CARLDVGERGVARGFNALALAANYGGAVCGRHAGGGGLYYSATDRKPDGKPGSAGD*LRRGVWRGVDAVSGAG*CLWLAVTCRQSRRGGDAVD
HYDRRRPRWIFPTPYVTGGDGVKHRVHHAFDDVAGKW*PANGASADLDFRFDLQRDRCAGLAHRNCDGDFAGDYPAVPPLADHFTAGW*YRPS
RRNGADADANCAAAVSGLPDGDRDDDYWTVEFCWFNGTAYCADDGLSTDDATHRNFGAGGWFTAGVR*LVWADGAVSIPDPGGAAVNLYRRAI
FYLFVEKAEPL
>NC_000913.3_cds_NP_414696.1_150_1 [gene=hemL] [protein=glutamate-1-semialdehyde
aminotransferase (aminomutase)] [protein_id=NP_414696.1]
[location=complement(173602..174882)]
MSKSENLYSAARELIPGGVNSPVRAFTGVGGTP-TVYRKSGRRLSVRC*WQSLYRLCRFLGADGAGP*PSGNPQCRD*SRRAWFKLWCTNRNG
SENGATGDRTGPDHGYGAHGELRH*SDHERHPPGPWFYRSRQNY*I*RVLPWSR*LPAGESRFWRTHVRPAKLAGRSGRFRQIYLNLYL**SG
FCTRRI*AIPARDCLYYRRAGGRQYELCSAAARVPARSARAVRRIWRVADHR*SDDRFPRSASWRTGLLRRSARFNLPRQNHRRWNAGRRIRW
SS*CNGCAGPDGSGLSGGYAFR*PDCDGSGFRLSE*SRAAGRSRNAG*ADNTSGRRSAGSGRRSRNSAGR*PRWRHVRYFLYRRRVRDVLSGC
DGL*RGTL*AFLPYDAGRRCLPGTVSV*SGLYVRGAQHGRYQ*HHRCCTSGVCEVV
>NC_000913.3_cds_NP_414697.1_151_1 [gene=clcA] [protein=H(+)/Cl(-) exchange transporter]
[protein_id=NP_414697.1] [location=175107..176528]
MKTDTPSLETPQAARLRRRQLIRQLLERDKTPL-SHFVYGGSRRHACWAGSGCF*QRCRLVAEPTYGGAGTYC**LSASVNRRFSLFGGAGDV
WLLFGAQIRAGSRWFGDPGN*RGAGRSTSRSLVACIAGEVLWRAGDTRRRHGVGARRANRADRR*HWPYGA*YFPPER*RSSPYAAGNRCCCG
AGCGL*RAAGGYFVYYRRDASAVSLYVNFD*SGIYWCHYVDHYVPDF*S*SCVD*RR*TF*CAA*YAVALSDPRYYFWHFRPYF**MGAGDAG
FAAPCARRQYYQMGANGRCDWRSVWIAGVCGTSNVGRRF*PDSYRYRGEFQHGNAGVYLRRAGHYHLTLLLFRRAGRYFCPDAGAGYCAGNRF
RNGCR*AVSAISP*GGDVCYCRNGGITGGIYSRAVNGDHSGSGDDR*LPAHFANDYYRSWRNTISAIYRRETAILGDSCAHAGKTGS*ATGAK
QGRISQREYL
>NC_000913.3_cds_NP_414698.1_152_1 [gene=erpA] [protein=iron-sulfur cluster insertion
protein] [protein_id=NP_414698.1] [location=176610..176954]
MSDDVALPLEFTDAAANKVKSLIADEDNPNLKL-TRVYHRWRLQRLPVWFHL**SGERRRYDHRKTGRWPGG*SDEPAISGRRFR*LYRRSGR
FSFHRDQPEREKHLRLRFFL*YL
>NC_000913.3_cds_NP_414699.1_153_1 [gene=yadS] [protein=UPF0126 family inner membrane
protein] [protein_id=NP_414699.1] [location=complement(177001..177624)]
MLVYWLDIVGTAVFAISGVLLAGKLRMDPFGVL-STGRGNRSRRRDNSRHGAGSRPGILGERSHRSGRCNGHQHADHRAGAPAKTLTKMDVAG
AGRRWSGGVCRHWRE*SL*CGSRSVNRGLYGRHYWRWRRDHS*CSGPRNPHDFTYRNLRNCLYYRRYCPRYGLLHIFRTTGNSQYDGHGRDAI
DSAGGYSLAS*ATDVCAG*EWAL
>NC_000913.3_cds_NP_414700.1_154_1 [gene=btuF] [protein=vitamin B12 ABC transporter
periplasmic binding protein] [protein_id=NP_414700.1] [location=complement(177662..178462)]
MAKSLFRALVALSFLAPLWLNAAPRVITLSPAN-N*TCLCRRDHAGWGQQLFRLSSTSAKD*AGFHLAGDESGTHCRAETRSGDCLAWR*CRA
AG*PAGFAGNKSDVGRCDKH*TNCQCVTSTGPLESATRQGRTSRAIPAGSVRAIESAIC**T*KTCFSAIRH*SAIYQWKRVDSEPGTRSLWR
RKHL*RQPGSLAAS*PRTGVSTLATGDCHYRRTGPNS*NQTILG*TAQNSRYSSHE*LV*TCKPTYYPRCTTAL*CAFTGRL
>NC_000913.3_cds_NP_414701.1_155_1 [gene=mtn] [protein=5*-methylthioadenosine/S-
adenosylhomocysteine nucleosidase] [protein_id=NP_414701.1]
[location=complement(178455..179153)]
MKIGIIGAMEEEVTLLRDKIENRQTISLGGCEI-IYRPTEWNRGCASEIGHR*SRCGAGCHFAVGTLQARCDY*HRFCRWPGTNVESGRYRCL
GRSTLSRRGCHGIWL*IRSVTRLSGRL*S*R*TDRCR*GLHCRTES*RCTWPDC*RRRFHQRFCWSGENPPQLPTGHCCRDGSDGNRPCLPQF
QRPVCCRTRHLRRGRSTVSS*LR*VPGCCR*TVQPDG*VTGAETCTWL
>NC_000913.3_cds_NP_414702.1_156_1 [gene=dgt] [protein=deoxyguanosine triphosphate
triphosphohydrolase] [protein_id=NP_414702.1] [location=179237..180754]
MAQIDFRKKINWHRRYRSPQGVKTEHEILRIFE-KRSRAYHQLSGNSSSATKDPGFSTGAQCRRAHASYPLDGSPAGGALHRQRNFKPSERA*
ITGSIRPG*TDRSL*KHC*DVMPDARYRQSAVWSFWRSGDK*LVSPTFAPGRCRKPASD*RSLQRGGTTFTGRGRTA*RAAAQDSSGLMSF*G
ECTRHSPGAYIDADESHLGTGWRYFKIYPSGVVAWRNA*DTSLFNEKAGLLSF*RSLYCPVA*RT*FGALQSFSINVDYGSCRRHLLLCGRP*
RCGREKNIYR*AALSSFARSVGPA*ERFALFAGG*KCLGKITLK*FKPQYGRSVFYVFTGKHPK*TGTLRGTTIY**SACDFRRNV*SCIIGR
CQRMQRSS*AI*KCRCKTCV*PSRCRAA*IAGLSGH*RIIRDLSSFIKPVVIRLY*TGRKRTGETFPY*IALIPQTLDAPSAGLCRGCQ*ITV
RFS*VSAMGILLPLPPAAGLYQRYDRPLCVG*IPTSDGRRTI
>NC_000913.3_cds_NP_414703.1_157_1 [gene=degP] [protein=serine endoprotease (protease Do),
membrane-associated] [protein_id=NP_414703.1] [location=180884..182308]
MKKTTLALSALALSLGLALSPLSATAAETSSAT-NSPADAKPCTDARKGDAFSGQH*RRR*HNR*YAAYAA*FPAVLR**FSVLPGRFSVPEL
SVLPGWPGR*WWRPATEIHGAGFRRHH*CR*RLCRHQQPRC**RDGH*SSTERWP*VRREDGWQRSAL*YRADPNPEPEKPDRN*DGGF*CTA
RG*LHRSDW*PVWSGRDGNFRDCLCAGA*RPECRKLRKLHPDRCSDQPW*LRWCAG*PERRTDRYQHRDPRTGRRQHRYRFCYPE*HGEKPDL
ADGGIRPGETR*AGYYGD*AELRTGESDES*RPARCFRKPGSA*FLRCKSGH*SG*CDHLTER*ADQQLCRTACSGGYYAGRQQTDPGLTARR
*AG*REPGTAAEQPESG*FQLHLQRH*RR*DEQQRQRSGRGSEQRENGHSGCADRPEER*CDYWREPAGSEKHR*TA*SSRQQTVCAGTQHSA
RRQHHLPVNAV
>NC_000913.3_cds_NP_414704.4_158_1 [gene=cdaR] [protein=carbohydrate diacid regulon
transcriptional regulator; autoregulator] [protein_id=NP_414704.4] [location=182463..183620]
MAGWHLDTKMAQDIVARTMRIIDTNINVMDARG-TNYRQRRS*AYW*IARRCIAGTFTGTSRRYR*RGSTSSARCAAGD*STVTAGR*NCRRN
WPDR*TRESA*IWRTGLHDG*NDAGTVAVDALVGAG*PFAGRTGDEPDSGRGEYSRTY*MGATAGDRSQSTASGGYC*GRQRSAWRGQRNGGV
TTTAKRADYARA**SGGDCLANRNGGVETGVELFWALGCRRSS*AS*TTDYPHERVRPAAFSRFTGQLFYRSWQYCPILSYGENDDGGG*TAD
```

ARKSLLFLSGSDVTCVTRQFAWRLAGQRTGATAGAAENDGQ*RLAATNAGGVVSPQCATAGNVKGVVYSS*YPGVSA*SYIGTDRA*FGQF**
QVAAVCGVTTG*RAV
>NC_000913.3_cds_NP_414705.1_159_1 [gene=yaeH] [protein=UPF0325 family protein]
[protein_id=NP_414705.1] [location=complement(183709..184095)]
MYDNLKSLGITNPEEIDRYSLRQEANNDILKIY-IPERQRRVFRQER*V*ISASA*NGRR*WCGSGL*RSPGNQPESTVYH**A*SNLPA*PQ
RS*S*A*DPRRLTSPGVSRNQ*DQRD*SRSGKTNA*I
>NC_000913.3_cds_NP_414706.2_160_1 [gene=yaeI] [protein=phosphodiesterase with model
substrate bis-pNPP] [protein_id=NP_414706.2] [location=complement(184257..185069)]
MISRRRFLQATAATIATSSGFGYMHYCEPGWFE-INPSPPRLF*RQRSTIQNSFSGRSPLLSFCSFKPDF*RDCSWHRTKARFDITGRRLRII
*YVAEFFGV**RTLPPCRMCADVCLLRQPRSPCWYRKKSLNWRDVEISGHHGVV*PGYGDRHAEQAIRTGRHW*LMGRTMQTASRQRSQSAKT
GAGA*SRQQRSHA**TLGSDAVRPYSRRATARTVGW*TFCPCRR*TLRRRIKCLWRKTHLHNPWRGQFVWFASELPPGSNDAGTGV
>NC_000913.3_cds_NP_414708.1_161_1 [gene=dapD] [protein=2,3,4,5-tetrahydropyridine-2-
carboxylate N-succinyltransferase] [protein_id=NP_414708.1]
[location=complement(185123..185947)]
MQQLQNIIETAFERRAEITPANADTVTREAVNQ-SDRPAGFRRTACSGKN*RSVGDASVVEKSGAALFPY***SGDRRGRKPLLRQSADEIRR
LRRSTFPERRLPRCATSGGTSGCVYCP*HRADAVLRQHRRIC**RHHG*YLGDRRFLCADW*KRPPFRWRGHRRRAGTAAG*PNHH*R*LLHR
RAL*SG*RGDCRRRFRHFHGRIHWSEHPYLRP*NRRNPLRSRSGGVCGCFR*SAVKRWQIQPLLCGYR*ES*RENSRQSRH*RTAAYHRL
>NC_000913.3_cds_NP_414709.1_162_1 [gene=glnD] [protein=uridylyltransferase]
[protein_id=NP_414709.1] [location=complement(185978..188650)]
MNTLPEQYANTALPTLPGQPQNPCVWPRDELTV-RWDKSPYRYFPALAG*CL*QWDLCRTVD*GAHRVYRPAPATIMD*SGIQPDCRPGIGRR
RWLRSWRAASTFRRRFTDFKP*KAPGRSGAKSGRAVNAALGCKAGSRS*RAHA*RVHAGRVIGFNRRHQFNRIPLINWRCCAVPRTAKTYFQR
RILAFRQVLRGES*RTEPAPSALPWHQLQP*TRHQKQPWRLARYPHSAMGGPPSFWRNIAG*NGRVWLLNLSGAGGIKRMSAYIVAYSLCPAS
GRQPLR*SPVIRSPA*RRPASELQW*R*RTGRADDEGLLPRYTPRQ*TQPDAAATVR*SHPRPSRRRKTTSNRR*VSATRYANRPA**NTIYA
PAGSHLAYVLHHGAQQCDHRHLLHHAAPVTPCPSPSATTAV*YSGSTKTVFEHSASPRSGAARAIANASP*RARRVYAAMVAYRRADAV*SVP
RLHGG*TYYPRDAETGEFCQ*RNAPAPSVVCGRLAAPAVN*ADFHRRAVSRYRQRTRRRPLHSRCSGCSAFCRTPRAELTRNTAGRLAGSPAP
VDVGDRPTPRYSGPGSHQAVCRRSANGKSSALSGMPDCG*HLRHQRNAVE*LEAKSVA*ALLCHRKAATTRNAKHAGYARTGSPSPTPGTGTT
AHG*HRRRGAAPNLVTLSC*LFCPP*PKSTGLACPPFITA*FKQTAGIA*PAGYAWRHRDFYLEPGPPLSVCRRLCRIRPPQFKCSRRTNFHH
SRRYGDGYLYRAGTRWQPAVRRSS*GYSVWSGASTDAK*LAATAAPSPTRQITPFYC*NRSNVFADPYRPQIVPRTDRPRPTWTAGASRENFC
RSGNFASWCPNYNHWRASRRFIHNCHR*PACA**RVAAGSASAVDRGPQSKR*RV
>NC_000913.3_cds_NP_414710.1_163_1 [gene=map] [protein=methionine aminopeptidase]
[protein_id=NP_414710.1] [location=complement(188712..189506)]
MAISIKTPEDIEKMRVAGRLAAEVLEMIEPYVK-TGRQHRRAGSHL**LHC**TTRGFCLPRLSRLSEIRLHLY**SGVPRYPGRC*AAERWR
YR*H*CHRNQRWFPRRYLENVYRR*ADHHGRTSVPHHARKPVPGATHGKTRH*SARNRCGDSEICRSRRLLRRS*ILRTRYWSRLP*RTAGAA
L*LP*NQRRTETWDDVHHRANGQRG*KRDPHHERWLDGKNQRSQLVCTI*AYYCGD**RLRNSDATQG*HHPGDNLARRI
>NC_000913.3_cds_NP_414711.1_164_1 [gene=rpsB] [protein=30S ribosomal subunit protein S2]
[protein_id=NP_414711.1] [location=189874..190599]
MATVSMRDMLKAGVHFGHQTRYWNPKMKPFIFG-SA*QSSHHQP*ENCTDVQRSSG*TEQDCFSQR*NPFRWY*TRCKRSGERRCSELRPVLR
EPSLAGRYAD*LENRSSVHQTSERPGNSVSGRYFRQADQERSADAHS*AGETGKQPGRYQRHGRSAGRSVCNRC*PRTHCYQRSKQPGYSGIC
YR*YQL*SGRC*LRYPG*RRRNPCCDPVPGRCCCNRT*RPFSGSGFPGGRKLRRS*V
>NC_000913.3_cds_NP_414712.1_165_1 [gene=tsf] [protein=translation elongation factor EF-Ts]
[protein_id=NP_414712.1] [location=190857..191708]
MAEITASLVKELRERTGAGMMDCKKALTEANGD-NRAGNRKHA*VRCY*SSEKSRQRCC*RRDQNQNRRQLRHHSGS*LPD*LRCKRRWFPGV
RRQSSGRSCCWQNH*R*SSESTVRRRTCCAGSENW*KHQHSPRCCAGRRRSGFLSARCAYRRSGCC*RR*RRAG*THRYARCCKQARIHQTGR
RIR*SGRKRIPGTAGYRDAVW*AERNRRENG*RPHEEIHRRSFSDRSAVRYGTKQNCWSAAERA*R*SDWLHPLRSG*RHRES*D*LCSRSCC
DVQAVL
>NC_000913.3_cds_NP_414713.1_166_1 [gene=pyrH] [protein=uridylate kinase]
[protein_id=NP_414713.1] [location=191855..192580]
MATNAKPVYKRILLKLSGEALQGTEGFGIDASI-TGSYGSGNQRTG*TGYSGWCGDWWG*PVPWRWSGESGYEPRCGRPHGDAGDRNERPGNA
*CTAPRLCERSSDVRYSIEWRVRQLQLGRSYQPVAQQPCGDPLRRYR*PVLYHRLSSLPAWYRN*SRCGAESNQS*RRVYR*SGERSNRNHVR
ATDLQRSAGKRAESHGPGGLHAGS*P*ITDSCFQYEQTGCAAPCGNG*KRRDFNHGI
>NC_000913.3_cds_NP_414714.1_167_1 [gene=frr] [protein=ribosome recycling factor]
[protein_id=NP_414714.1] [location=192872..193429]
VISDIRKDAEVRMDKCVEAFKTQISKIRTGRAS-TQPAGWHCRGILRHADAAASAGKRNGRRFPYTENQRV*SFNVSGR*KSDYGVRSWPEPE
LCG*RHPCSAAAADGRTS*RSDQNRSW*SRTSACCST*RAS*RERQSESTVER*RDQRRRRSPFSGRCTETD*CCNQEN*SGAGRQRSRTDAV
L
>NC_000913.3_cds_NP_414715.1_168_1 [gene=dxr] [protein=1-deoxy-D-xylulose 5-phosphate
reductoisomerase] [protein_id=NP_414715.1] [location=193521..194717]
MKQLTILGSTGSIGCSTLDVVRHNPEHFRVVAL-SGRQKCHSHGRTVPGILSPLCRNGR*SECETS*NDATATG*PHRSLKWATSRLRYGSA*
GC*SGDGSHCWRCWAVTYACCDPRG*NHFAGQ*RITGYLRTSVYGRRKAEQSAIVTGR*RT*RHFSEFTATYPA*SGIR*P*AKWRGVHFTYR
VWWPFP*DAIARFGNNDAGSSLPSSELVDGA*NFCRFGYHDEQRSGIH*SALAV*RQRQPDGSADSPAVSDSLNGALSGRQCSGAAGGTGYAY
ANCPHHGMAESRELWREAARFLQTKCVDICRTGL*SLSMPETGDGGVRTRPGSDDSIECRKRNHRCCFSCATNPLYGYRCVEFIRTGKNGYAR
TTMCGRCVIC*CERA*SRQKRGDASRKL
>NC_000913.3_cds_NP_414716.1_169_1 [gene=ispU] [protein=undecaprenyl pyrophosphate synthase]
[protein_id=NP_414716.1] [location=194903..195664]
VMLSATQPLSEKLPAHGCRHVAIIMDGNGRWAK-KAREDSCLWA*SRGKIRPPGCLFCGQQRY*GVNAVCL***KLEPTSAGSQCVNGTVCVG
AR*RSKKSAPT*RASAYYWRYQSL*LAFARTYS*I*SANSREYRSDAEYCGELRWTLGYSPGSQATG*KGAARKPATRSDR*RDAKPACLYA*
TGPCRFSN*DWGGASH**LFALANCLCRTLLYRCSLARFR*TRL*RGVKCLC*SRASFRRHRAR**NSL

>NC_000913.3_cds_NP_414717.2_170_1 [gene=cdsA] [protein=CDP-diglyceride synthase] [protein_id=NP_414717.2] [location=195677..196534]
LLKYRLISAFVLIPVVIAALFLLPPVGFAIVTL-SGLHAGSVGMGTA*RFYHSFAASMVGGVMRVIVGADAFSVAGISPKYSSTAG*NLTLGF
AGLVDCRAIAGAVLPRFRSNLA*L*NIAPYFWRANHCSLLLGHAGVTGLAL*RESLQWRNMAALCHDPGMGR*LRRIYVWQIVW*T*AGTEGF
SG*NLARLYRWTRYCSGNLMGLWHVGESRRRSRHLTHLLYCRSVSLSARRSDREYV*A*SRN*GQRSFNSRTRWYFRSY**PDGCGTGLCLLV
VTGIQDAL
>NC_000913.3_cds_NP_414718.1_171_1 [gene=rseP] [protein=inner membrane zinc RIP metalloprotease; RpoE activator, by degrading RseA; cleaved signal peptide endoprotease] [protein_id=NP_414718.1] [location=196546..197898]
MLSFLWDLASFIVALGVLITVHEFGHFWVARRC-RCSR*AFLNRVW*GALAAN**ARHRICYRPDPVGRLCQNAG*ARRTGRSGTPPPCLQ**
ICRPTSGDYCRRSGCKLHFCYLCLLAGFYYWCAWRTSGGWRNSSQFDSCGSTNCTRYGTKSRRWYRNA*LGCRAFAVGR*NWR*KHHHYSSAI
WQRPTAGCKARFTSLGV*A**RRSGIFAGDSSSWAAN*TCTGKCAAKLGGKQGRFASRRQDR*SRWSALNAVGDLCDACPG*PG*ILSVRNRK
AGESLVFDINPGE*TG*W*SDWFCRY*AESHSFAR*V*SCTPVWAVQRHRRSHGQNVAADEADGQYAGKIDHR*CETEQPQWADLYRQGGWDD
SGTRGCLLPAVSCAY*RELRDN*PVSVART*RGASAVPCDRKDQGRTGIRAGSRLLLSHWLDSAGAVNGACTFQ*FLSVM
>NC_000913.3_cds_NP_414719.1_172_1 [gene=bamA] [protein=BamABCDE complex OM biogenesis outer membrane pore-forming assembly factor] [protein_id=NP_414719.1] [location=197928..200360]
MAMKKLLIASLLFSSATVYGAEGFVVKDIHFEG-TSACRRWCGPPQYAGAHRRHG***RYQ*YHSRSVCYRQL*GCSRPS*W*YPSGSGKRTS
DHCQHYFLR*QIGER*HAEAKPRGFWCACGRIPRSHHHCRYRERSGRLLLQRR*I*RQRKSCRDPAAAQPC*PKTGVPGRCVS*NPAN*HCW*
PCFHHRRTDLSFPTA*RSAVVERGRRS*IPETETGGRP*NPAQLLSGSRLCPFQHRLYPGQSDAR*KRYLRHGEHHRRRSVQAFWR*SERQPC
RALR*N*AAD*DRAG*AV*RHQSDQDGR*HQKASRSLWLCLSARTVDARN*RCRQNR*ITCER*CG*PFLRA*DPF*R*RYLERCRPASRNAS
DGRCMAGERSGRSG*GASESSGLL*NCRYRYPTCSG*PGPG*CRLQGKRAQHR*LQLWYWLRY*KWRELPGWCAAG*LVRYRLCCWYQRDQKR
LPDLC*TVGNQPVLHRRWRKPRWSSLL**LPGR*RRPVRLYQQELWYRRDVGLPD*RI*LAACRSGLCT*LPVQHAASGCDVALSVLYG*TSE
HL*SG*QLQNGRLHVQLWLDL*QA*PWLLPDRWFTCQPDR*SDHSWIG*RILQSDVRHGDLCADR*RSQMGCSGAYPLGLW*WFRRQRDAVLR
ELLCRWFQHRAWLPVQYHWSESSLLPASGQ*L*SGL*LRMCDSGRRERPV*IG*CCRR*RHGGCQPRVHHPDAVY*R*VC*LGSYFLLLGYGY
RLGYKLGFQPIFWISGL**SKQYPYVCGYRITMDVPIGAVGVLLRPAVQKVRWRQGRTVPV*HR*NLV
>NC_000913.3_cds_NP_414720.1_173_1 [gene=skp] [protein=periplasmic chaperone] [protein_id=NP_414720.1] [location=200482..200967]
VKKWLLAAGLGLALATSAQAADKIAIVNMGSLF-TAGSAENRCF*HAGK*VQRPCQRTAAYGNRSAG*NEKAAVHESGQRSH*AGKRRDGSAP
DFCSESAGF*AGSRTSFQRRTRQTGYSYPDCCEIRCQQPGYRSGC*CKRRCLQQQRCKRHHCRRTETG*I
>NC_000913.3_cds_NP_414721.1_174_1 [gene=lpxD] [protein=UDP-3-O-(3-hydroxymyristoyl)-glucosamine N-acyltransferase] [protein_id=NP_414721.1] [location=200971..201996]
MPSIRLADLAQQLDAELHGDGDIVITGVASMQS-STNRSHYVHG*PKIP*AFRLVPGVRGCHDPGRSSFREKCRTGSEESLPDLRAHGANFRY
HAAARAEHCTQCGDRRDGEAG*QRIDWR*RGD*VRR*TGR*RDYRCRLLRR*KQQNRCRFASLGERNHLP*DPDRSELPDPVRNSGRRRRLWL
CQRSW*LGEDPTDWSRNYWRSRGDRCLHNHRSRRAG*HYYWQWRDH**PVPDCT*RRDWRQYGGCRWRHYGGQPENWSLLHDRRSQRNQRAYG
NMRQSDGYGHGYGDASHH*TRRLFLRHSAATQQSLAQNRCTGDEH**HEQASEIA*AQG*STRL
>NC_000913.3_cds_NP_414722.1_175_1 [gene=fabZ] [protein=(3R)-hydroxymyristol acyl carrier protein dehydratase] [protein_id=NP_414722.1] [location=202101..202556]
LTTNTHTLQIEEILELLPHRFPFLLVDRVLDFE-RRSFSARSKKCLCQ*AILPGPFPWKTDFPGCADSGSNGTGNRYSGV*KRRKTGTG*AVL
LRWY*RSALQAPGRAWRSNDHGSHFRKNAPRPDPF*RGCSGRW*SSLRSNDDVCS*PGGL
>NC_000913.3_cds_NP_414723.1_176_1 [gene=lpxA] [protein=UDP-N-acetylglucosamine acetyltransferase] [protein_id=NP_414723.1] [location=202560..203348]
VIDKSAFVHPTAIVEEGASIGANAHIGPFCIVG-TPCRNW*GYRTEISRCREWSY*NWPR**DLSVRLHRRS*PGSEICWRTDPCGNRRS*PH
SRKRHHSSWHSPGRWIDEGGQRQLTDDQRAHCARLYGR*PLYSRQQRNAGGSRIG*RLRDHRRHDRSPSVLHHWCARDGWRLLRCGAGRPSLC
HCAG*PRNAVRCQYRRAEAPRIQP*GDYRYPQCV*ADLS*R*NAR*SETGNC*TGGNISGSESLYRFLCTLNARSDSL
>NC_000913.3_cds_NP_414724.1_177_1 [gene=lpxB] [protein=tetraacyldisaccharide-1-P synthase] [protein_id=NP_414724.1] [location=203348..204496]
MTEQRPLTIALVAGETSGDILGAGLIRALKEHV-TQRPLCWCCRATNAG*RLRSLVRNGRTGGDGHC*SARSSASLTAYSCRSDKAFWRTEAR
CFCWY*CA*LQYYS*R*PQKAGYQNHSLRQSVSLGVATETCFQNRQSHRSGARISAFRKSVL*QIQRTVPLYRSYHG*CHAIRSR*KCRP*CA
GDPSRCPLPGVATGEPWCRS*NA*CRFPENGPAFAPDISGSRNRGATGECQTPRAV*THQS*SRARPFSSFAGWDGP*GDGRQRCGATGVGYG
SPGVYAGEMPDGGGISHEAFYLLVGEAAGEN*LCLAAKSAGGQRVSQRIIAGRV*AAKTGCGAVTAVGERENQPRDARYLP*TASADPLQCR*
AGGTSRSGVSTM
>NC_000913.3_cds_NP_414725.1_178_1 [gene=rnhB] [protein=ribonuclease HII, degrades RNA of DNA-RNA hybrids] [protein_id=NP_414725.1] [location=204493..205089]
MIEFVYPHTQLVAGVDEVGRGPLVGAVVTAAVI-T*PGAPDCRAE*FQKAERKTPSGAL*RDQRESVELESGPRGTPRNRRAEHSSCDHAGDA
ACRRWAAYCAGICVD*W*PLPEITDACDGCGERR*PRTGNQCRVYPGESDA*RRNGGAGYCFPAIWFCPTQRVPNRFSSGKTG*TRRDRTPSA
QLWACQTRTGTCVL
>NC_000913.3_cds_NP_414726.1_179_1 [gene=dnaE] [protein=DNA polymerase III alpha subunit] [protein_id=NP_414726.1] [location=205126..208608]
MSEPRFVHLRVHSDYSMIDGLAKTAPLVKKAAA-IGYASTGDHRFHQPLWSGEVLRSGTWRRD*AYRRGRF*RPVRPAG**VNPPDGTGGEQY
RLSESDVADLKSVSARVRCRRADHRSRLAYRIKRRVDPSFRRTHGRRRTQSFAW*QRAGR*VCRVL*RTLPGSLFSRADPHRQAG*RKLSARG
GGTGGSARFARRGDQRRALYRQQRL*RTRNPRRDPRRLYPRRS*TPA*LFAAAIYA*RRGDV*AVCRHPRSPCQHR*DRQTL*RNRASW*ILP
AAVPDRGHEHRRLSGQACKRGPGRASGLFIP**GRTS*APPGI*RTSGD*TSGYQPDGLPGLLPHRYGIYPVVER*RRTGRARPWLRCGFTGG
LRAENHRPRSAGI*PAVRTFP*PGTCLHA*LRR*LLYGETRSGYRARSGHVRS*CGIADHHLRYNGGESGDPRRRPRAGASVRLCRSYLETDP
ARSGDDAGESV*SRAAAAGNLRSG*RS*GADRHGAQTGRGHP*RR*ARRWGGYRADQNYRFCAALLR*RGQTSGHPV**KRR*IRRTGEVRLP
WFAYAHHHQLGAGDDQQAAGEEWRAAAGYRCDPAG**EKLRHAATLGNHGGIPA*IARHEGPDQASTT*LLRRYDRPSGTVPPRSVAIRDGG*
LYRP*TWS*RDLLSGRTVAA*KPETGTGANLRHYPVSGTGHADCAGAFWLYPRWRGYAASCDG*EKAGRDG*ATFCIC*RCRKERNQR*TGDE
NLRPGGEIRWLRI*QIALCGLCFGVISNVMAESALSCGVYGGGNDRRYGQHREGGGSGG*VLADGAENPATRYKLRSLPFPRQRRRRNRVWYW
RDQRGR*RSD*GHHRSP**RRLLPRTV*SLRPYRHQKVEPSRAGKTDHVRGV*PSWATSRSADELAGRCVKSGRSTRESGSYRSGRYVRRAGR

RAGTN*TILRQLPTVAGAGGIRWGT*NVRPVPDRTPYQPVFKRD*ALCRRRKAERHAPDRTW*SHHGCGARCCRAGYGHQARQSYRYLHAG*P
FRAAGSDVVY*RPG*IPAIAGKRPHTYRQRTGQL**LQRWA*NDRSRSDGY*RSPGKICSRACYLADGQAN**PAFKPTPSVSGTPPLWDNSS
TSLLSEGGCTRAVAFWRDVACLSERSFIKRSPWPHWFGAGGTGV*L
>NC_000913.3_cds_NP_414727.1_180_1 [gene=accA] [protein=acetyl-CoA carboxylase,
carboxytransferase, alpha subunit] [protein_id=NP_414727.1] [location=208621..209580]
MSLNFLDFEQPIAELEAKIDSLTAVSRQDEKLD-N*HR*RSASSA*KKRRTDT*NLRRSRCMADCATGTPSTASLYPGLRSPGI**I*RTGWR
PRVCRR*SYRRWYRPSRWSSGDDHWSSKRS*NQRKNSP*LWYASARRLPQSTASDANG*TL*DAYHHLYRHPGGLSWRGRRRAWSV*SHCTQP
A*NVSPRRTGSLYGYR*RWFWRCAGDWRGR*SEYAAIQHLFRYLAGRLCVHSVEERRQSAAGG*SDGYHCSASERTETDRLHHPGTTGWCSP*
PGSDGGIVESATAGGSGRSRRVKH*RFKKSSLSAPDELRLRV
>NC_000913.3_cds_NP_414728.1_181_1 [gene=ldcC] [protein=lysine decarboxylase 2, constitutive]
[protein_id=NP_414728.1] [location=209679..211820]
MNIIAIMGPHGVFYKDEPIKELESALVAQGFQI-NLATKQR*FAEIYRA*PSNLRRDF*LG*VQSRFM*RYQSA**ISPALCLHQHPLDDGCQ
RAGYADGALVF*ICAGAGGRYRHSYASVHRRIS**HYTAVHESLVYLRQRAEVHLLYAGAYGRHRISKKPGWLSVL*FFRREYS*G*CLYFGH
RAWFVARPHRATPGSGRVHRADFWRGTELYRYQRNIDVEQNCGYVRRAIRQYAVDRPQLS*IAGASVDDERCSASLAETDA*CVGDSWWDPAP
*IYSRQHRRESRCYHASTMAGSCGDHQLHL*WLALQHRLDQTDAGCPVDSLRFCLGAVHPFSSDLPG*KWYERRACCGKSDLRNAIDPQNAGG
VIAGFADPH*RRV*RRGL*RSLYDAYHHLAQLSHCCFG*DGGGDAAW*SGQTAD*PFSRTSSAFSQRGPAAAGRV*RLVFRYLATAAGG*SRM
LARCAWRTVARL*RCGCRSYVSRSG*SHYFDTGDGRAGQYERGGDPGGAGSKIPRRTWDRSRENRPL*PAVSL*YWHR*NQSNGIIAWVDGIQ
TLLRSQPADQKYATRSLCRRSRFLPQYAYSGSGTRDP*ADS*TRSSRFDVAGIRYFAGDDHDATSGMATTN*RRSRNHCAGTTGR*SIGKYDP
ALSTGRTAVDAWRNADQREPHSTRFSTDALFRRATLPRF*NGYSRRETGRRRRLPRTSPKNGGI
>NC_000913.3_cds_NP_414729.4_182_1 [gene=yaeR] [protein=putative lyase]
[protein_id=NP_414729.4] [location=211877..212266]
MLGLKQVHHIAIIATDYAVSKAFYCDILGFTLQ-KRSLSRSARLMERGFGA*WAICD*AFLISVPAGTTQPTGSLRSASSGF*R**HRCGSGA
P*KP*REV*NHPCRSIHAKTLHLL*RSGRAAVGTV*AV
>NC_000913.3_cds_NP_414730.1_183_1 [gene=tilS] [protein=tRNA(Ile)-lysidine synthetase]
[protein_id=NP_414730.1] [location=212331..213629]
MTLTLNRQLLTSRQILVAFSGGLDSTVLLHQLV-TVADGKSGCRSARYSCASRFKCQCRCLGYALRKRLPTVAGAAGGRTRTTCARRTGH*GP
GAAGTLSGICPHLVAR*SAGHRATSRRSM*NLSAGAKTRQWPCRAFGYGGSLGVCRNAAYSPVARPHAGGTGAVGASV*FTLD*RRK*SGRLI
RS*LSAPARSAVIAAALAAFCRSNGPQRRTLC*TREPAG*TAGR*FSTLSIAAGDAADCANAGDE*CPPRGDYPPLAGRAECTDAFPRRVGED
LAGSGAGAGRCLTLFTFGRV*NPTLSVATVVD*IRHRAKRKHCAVADVASTAGITGGAGKCTA*CGRRYSPSACRRSGQRAFQSARIAAYCRA
*RRT*AKENLARAGRAAVAT*HHATAVLWRNADCGGRGICDARRCG*R*EWRKFCLAENA*L
>NC_000913.3_cds_NP_414731.2_184_1 [gene=rof] [protein=modulator of Rho-dependent
transcription termination] [protein_id=NP_414731.2] [location=complement(213678..213932)]
MNDTYQPINCDDYDNLELACQHHLMLTLELKDG-RKIAGKSQ*FSLPQKCGVPGRRGRWRNPRAASG*NYQL*PPGNRYGGGKRIL
>NC_000913.3_cds_YP_026160.1_185_1 [gene=yaeP] [protein=UPF0253 family protein]
[protein_id=YP_026160.1] [location=complement(213925..214125)]
VEKYCELIRKRYAEIASGDLGYVPDALGCVLKV-TE*NGCRRRPF*GCQGKSSIRCGKLTGERLCQ*M
>NC_000913.3_cds_NP_414732.1_186_1 [gene=yaeQ] [protein=PDDEXK superfamily protein]
[protein_id=NP_414732.1] [location=214291..214836]
MALKATIYKATVNVADLDRNQFLDASLTLARHP-IRNPGAYDAALAGVAEIC**TSAIYPWFVCR**AGSVAA*RSPGH*FVD*AGAAG*AAD
*ESLHPGRRSGAVYL**SGGANLVAAKSEQMCAVCQSFRLVSGR*TTGESKRLCRSYHDAAGNDSGWRDLVIG**E*SGSELNRLATTFM
>NC_000913.3_cds_NP_414733.1_187_1 [gene=arfB] [protein=alternative stalled-ribosome rescue
factor B; peptidyl-tRNA hydrolase, ribosome-attached] [protein_id=NP_414733.1]
[location=214833..215255]
MIVISRHVAIPDGELEITAIRAQGAGGQHVNKT-INGYSSAF*HSGVQPARVLQRASARRQPSFDQQ*WRDCH*GTGIPQSGTEPRSSSGPAG
GYD*RINNRKKSPTTHAAHPCIERAQAGIESTKIKREGDARQSAQRSGI
>NC_000913.3_cds_NP_414734.1_188_1 [gene=nlpE] [protein=lipoprotein involved with copper
homeostasis and adhesion] [protein_id=NP_414734.1] [location=215269..215979]
MVKKAIVTAMAVISLFTLMGCNNRAEVDTLSPA-TGCRTETDAAKLARRAAVCRLRRNRNLSVPRKRRNMGDE*ALSRCS*RTFLLRFLRYMG
ANR*QAGINRQQR*KVILSGERRCAGDARS*RQSD*IAVQLYAGSGTIQFTYDADDPAGHVFLYG*CGDLH*LRDRKTFHGSE*RRAGA*LPG
CARSQ*KTGVTVSRRSLYA*G*SGYRCAD*SIGTRYGREILPQPGLQ*FGAV
>NC_000913.3_cds_NP_414735.4_189_1 [gene=yaeF] [protein=putative lipoprotein]
[protein_id=NP_414735.4] [location=complement(216179..217003)]
MDKPKAYCRLFLPSFLLLSACTVDISQPDPSAT-SSRCRGKNMGCQIPASKLFHRTINKRNNGT*SQTRRFAVLLKPWGNLIWNPRLQHFLCE
SRCNLSG***RCRSDRRWRPDRFP*KSDEA***AFRLTSPGSYPATSHRYHRVCQ*NQR*RL*LSRHCRIYSLYGDSPDVLTESVLRGFSPTV
RQRPGESAVKQCGRRRQKVVVLFGICHGCLCQSRASTYTGAIGLD*PCRSDAYAYW*CLCV*ARNAVTVCWAPEAWDLYQGGSLCWFDPV
>NC_000913.3_cds_NP_414736.1_190_1 [gene=proS] [protein=prolyl-tRNA synthetase]
[protein_id=NP_414736.1] [location=complement(217057..218775)]
MRTSQYLLSTLKETPADAEVISHQLMLRAGMIR-KAGLRVIYLAADRRARSEKSRKHRA*RDEQRRCDRGVDAGGSASRFVAREWSLGTVRSG
TAAFC*PWRASVRTRPNS*RSYH*PDS*RA*LLQTAAAELLSDPDQVPRRSASAFRRHAFPRIPDERCLLFPYFSGIPAGNLRCNVCGLQQNL
QPHGAGFPRRTSRHRFYRRQRLSRIPGAGAER*RRCGLLRHL*LCSEH*TGRSYRAERTARCCYPGNDAG*YAERENHRGTG*TVQSAD*ENG
*DSAG*SG*RQQLPAGCAAGAR*SRAERS*SRKTAAGCKPADFRDRRRNSCRG*SRSGFTGSGKHADSGGD*PYRCGDE*FRCWC*HRW*TLL
RHQLGSRCRYPGSCRYP*RGGWRSKPGWPG*AADQTWYRSWSHLPAGYQVLRSTESLRTG*RWP*PNPDDGLLRYRGNACGSCGD*AELRRTR
HRMA*RYRAVPGGDSADEHAQILPRTRAC*ETVQRTACTRYRSAAG*PQRASGRDVC*YGTDRYSAHYCAGRP*PRQRRYRI*ISSQRRETVN
*DW*HRRISGETD*RL
>NC_000913.3_cds_NP_414737.1_191_1 [gene=tsaA] [protein=tRNA-Thr(GGU) m(6)t(6)A37
methyltransferase, SAM-dependent] [protein_id=NP_414737.1]
[location=complement(218887..219594)]

```
MSSFQFEQIGVIRSPYKEKFAVPRQPGLVKSAN-RRTASHCSLQPGRRRSRPGSVQPFMDPFRLSSNDGRRLASDCASTAPRR*RQNGGFRNT
LYFPP*PNWHVAGRAERGCLP*RQRDSEARQSGSGRWYAGSGYQTVSPLCRIASRCQCQLCAKCASCRDGGEFYRRGRKAAFDAGEALSAVNA
VYPRSTGAGPAPGLS*R*GNGQNLCRLAT*F*RSLARHRRRF*SLCAGTAL
>NC_000913.3_cds_NP_414738.1_192_1 [gene=rcsF] [protein=putative outer membrane protein]
[protein_id=NP_414738.1] [location=complement(219591..219995)]
MRALPICLVALMLSGCSMLSRSPVEPVQSTAPQ-TESGACKTESAARHAGPNLYQCRRISRQTVPRSR*SQWRLLPGL*SGLSAEHSNRT*AD
ANQRL*NESQCCITA*LRSHQRYARLLSSGCMYRFCA*HYGEM
>NC_000913.3_cds_NP_414739.1_193_1 [gene=metQ] [protein=DL-methionine transporter subunit]
[protein_id=NP_414739.1] [location=complement(220113..220928)]
MAFKFKTFAAVGALIGSLALVGCGQDEKDPNHI-KSRRDCWCRTAGCRSRAESCERQIWPGR*AGNLQRLCSAKRSIEQRRYRRQRLPA*TVP
*SATERSWLQTGRSRQHFCLSDCWLLQENQITG*TAGWFAGCRAKRPN*PWSFTAAAAKSGLDQTERWRWPAADRS*CC*EPQKSENC*TGST
ATAAFSGRRANRSGSYQYHLCQPDWPDSGERRYLC*R*RVPVRKPDRDA*R*QRRREREEIRPGLSV*RSLRSSKQSV*RRSC*RLV
>NC_000913.3_cds_NP_414740.1_194_1 [gene=metI] [protein=DL-methionine transporter subunit]
[protein_id=NP_414740.1] [location=complement(220968..221621)]
MSEPMMWLLVRGVWETLAMTFVSGFFGFVIGLP-SWRSALCHASGANYC*REAVSYRFCDCEHFPFHPVHYLACMDDSVYPRYCRYIDWFAGS
DCSVNRWCSTVYCPYGRERSAGDPNRVN*SFPRNGCHADADRP*GAVTGSAAGSGECGNYHPDYPGRLFRDGWCSRCRWFRSDWLSVWLHRL*
RDGDEYGTGIAGHSGLFNSVRRRPHRPGCHSQV
>NC_000913.3_cds_NP_414741.1_195_1 [gene=metN] [protein=DL-methionine transporter subunit]
[protein_id=NP_414741.1] [location=complement(221614..222645)]
MIKLSNITKVFHQGTRTIQALNNVSLHVPAGQI-IWRYRCLRRG*EYAYTLCKPAGAPNRG*RAGRWPGTDHAVRIRVDQSSPPDWYDFPAF*
PALFAYCFWQRGSAAGAGQHTERRGQTSRDGIAVIGWSWR*A**LPVESFRWAETTCGNCPCVSQQSQSIAV**SHQRAGPGNDTFYSRTAER
HQPPSGVDDSVDHPRNGRCEAHL*LRGGHQQWRTDRAGHGK*SVLASENAAGAEVYSVDPASGYPGRLPGTSASGAIY*LRADAASGVYRSIG
RCPTAF*NRASFQRQQQHY*RADGLRRWR*VRHHAD*NARHTTRYASRHCLAAGTPCKSRGTGLCL
>NC_000913.3_cds_NP_414742.1_196_1 [gene=gmhB] [protein=D,D-heptose 1,7-bisphosphate
phosphatase] [protein_id=NP_414742.1] [location=222833..223408]
VAKSVPAIFLDRDGTINVDHGYVHEIDNFEFID-RCY*RHARAKKNGLCAGGSNQPVWHCSR*IYRSTV*NADRVDGLVAGGPRCRSGWYLLL
PASSAG*C*RVSPGLRLPQTTSGDAFVSTRLFAY*YGRFLYGGR*IRRYAGSGCGERGNKSAGAYG*TYYA*SRKRGGLGVK*PGRPAASDKK
AAKTGTM
>NC_000913.3_cds_NP_414743.1_197_1 [gene=dkgB] [protein=2,5-diketo-D-gluconate reductase B]
[protein_id=NP_414743.1] [location=229167..229970]
MAIPAFGLGTFRLKDDVVISSVITALELGYRAI-RYRTNL**RSRSRSGDCRKWRATS*TLHHH*NLD*KSQQRQIDPKSEREPAKIAYRLC*
SDANPLAVTKR*SLC*RVYAGAAGSQKTRADA*DRYFQLHDPVDGKSDCCCWC*KHRY*PD*TLSLSAKP*SGCLG*TARHPYYFLYDAGVW*
GPER*GYCSYRS*TQCDSGTSDSGVGYGGRLLSNSFFY*T*KPGK*S*GTKFTA*CRR*KSDRRTGLQRPPG*PGRSGS*MGL
>NC_000913.3_cds_NP_414744.1_198_1 [gene=yafC] [protein=LysR family putative transcriptional
regulator] [protein_id=NP_414744.1] [location=complement(229967..230881)]
MKATSEELAIFVSVVESGSFSRAAEQLGQANSA-SKPGGEKAGDETWR*PA*SDHATT*PDGRRRALFSSRTVNFAGDGSGRIRNYGDA*YTA
WTVTDRCRNSSGAALSDAVN*AFP*TLSGSHFVASLLRNDY*FDRKKSGCRDTRWYVNGFQLTCQAVI*QLSKNYRLPRLYFPLREARNDRRF
KATYLPGIH*TRFPQYLADSP*RWTIT*GEVRFVIQ*WGNTETALPEWQRDCVFVRLHDRQRNRSRRIGGVNGR*SVASGNAIQCRLLQRPCG
KYAHPGFYRFP*RACKNSSRRSCQRGL
>NC_000913.3_cds_NP_414745.1_199_1 [gene=yafD] [protein=endo/exonuclease/phosphatase family
protein] [protein_id=NP_414745.1] [location=231122..231922]
VRKNTYAMRYVAGQPAERILPPGSFASIGQALP-TWGTVKYRRAYSDPGVEHIQTATR*MVVGIKELRQRCTSGVIAGSADNARVSTVCDR*L
SCRRSGTRFRAATTSFWRNDPFGGTSSVLLPVT*TRTHFASGEVGTGDGLSIT*HPPVDGG*YTRRQLQSGRGCL**AVTSYWRSDSSPQRPG
HYGGRFQCLEP*KDERVISLCAGNVAAPGAFYR*SAPSGVWSPARFCFLPWSERQ*SFCTGYARFRSQSATR*IQSRQA**I
>NC_000913.3_cds_NP_414746.1_200_1 [gene=yafE] [protein=putative S-adenosyl-L-methionine-
dependent methyltransferase] [protein_id=NP_414746.1] [location=231926..232549]
MSGLPQGRPTFGAAQNVSAVVAYDLSAHMLDVV-STSCRSPATEKYHHPPGICRKSAICR*RI*YCYQPLFCPSLA*CWCSTARSE*DIETWR
*ADCDGRNVSGSPSARHLVTDGRSITRYLSRTKLRQR*VVDVNQ*SQSDS**FNYR*VTAGIFFMGRENAYARSVSRRYSHLPTERIDRGENV
FCLAE*WLFHQ*YHHGRCT*SGI
>NC_000913.3_cds_NP_414747.1_201_1 [gene=mltD] [protein=putative membrane-bound lytic murein
transglycosylase D] [protein_id=NP_414747.1] [location=complement(232597..233955)]
MKAKAILLASVLLVGCQSTGNVQQHAQSLSAAG-TRGSSKVYKSGTMDGRWDVYRARW*LVGFHWRRAKDGNSGK*PDSRTETEIFTQ*ELSP
RCNFTGRAVYVLDSRAS*KT*HAYGTGTTTHSGERF*SSRNVWRQCRRHLADHSEHGAQLWFETDPQL*RASRCCCFNNCRAEYDAASEQNV*
RRLASDRSGL*QRRRSGHEGN*NEQSAWEIHGLLVVTVAAGNEAVRA*NAGIE*YSQKQQALWRTSANDR*KPCSGACAPEQPG*NGEGSRYG
GDFRQQAEDIQRWRKRLHAGRKWPAVRDGAKEACRSTA*ISGFRRNCCCTVDAGCRQYAA*QPCLHRTLWRHAFKYRFTSRRKHQRFAAVEQT
ARI*AEARPKFDDWCRQ*RTATGKQQR*HYVSCAQRRFAFKHC*TPRREHQRCDALEQRHCESATRR*ADVVCEKQQHARFL
>NC_000913.3_cds_NP_414748.1_202_1 [gene=gloB] [protein=hydroxyacylglutathione hydrolase]
[protein_id=NP_414748.1] [location=complement(234027..234782)]
MNLNSIPAFDDNYIWVLNDEAGRCLIVDPGDAE-TSIKRHCRQ*LATGGHISHPPSPRSRWRRKRTGGKVSTNCGVWSTRDTR*GNNTGSQRW
RNCLRFGA*I*CNCYAGSHFRTYLLLQ*TLSILRRHTVFWWVWSVV*RDSITNVSIT*KVKCVT*RYIGMLCS*IYLIKYEVCFEYSSARFVH
K*LLS*S*GVTGKKSNNTTRNSEK*AAN*CFFKNGRY*FN*CN**RNIIATT*RAFCMVKVKER*VL
>NC_000913.3_cds_NP_414749.2_203_1 [gene=yafS] [protein=putative S-adenosyl-L-methionine-
dependent methyltransferase] [protein_id=NP_414749.2] [location=234816..235538]
MKPARVPQTVVAPDCWGDLPWGKLYRKALERQL-KPVVH*NVWFSSA*DWQFKRRNQLRSVRGFSSSECFCARNARPGTGGPTSSSFCR*IR*
CLSTGTYIAVVHRSASFIA*SRSGID**WLAGH*WLQSHQFYGITQTCAGIAQNLAL*QPDVYSDAAAGLALFVEF*SATRQPFPRSPVEQTR
RKTIECAYSCAWLLTTYCCPETDYSFNAKSDETE*KQATNSPGGWSHPAMS*TTGL
>NC_000913.3_cds_NP_414750.1_204_1 [gene=rnhA] [protein=ribonuclease HI, degrades RNA of DNA-
RNA hybrids] [protein_id=NP_414750.1] [location=complement(235535..236002)]
```

MLKQVEIFTDGSCLGNPGPGGYGAILRYRGREK-NL*RWLHPHHQQPYGVDGRYCRAGGVKRTLRSHFEYRQPVCPPGYHPVDP*LEKTWLEN
RRQKTSKKCRSLATS*CCIGAASNQMGMG*RPCRTPGKRTL**TGSCRGDESHTGRYRLPS*SL
>NC_000913.3_cds_NP_414751.1_205_1 [gene=dnaQ] [protein=DNA polymerase III epsilon subunit]
[protein_id=NP_414751.1] [location=236067..236798]
MSTAITRQIVLDTETTGMNQIGAHYEGHKIIEI-RCR*SGEPSPDGQ*LPCLSQTRSAGGSGSLWRTWYCR*IFAR*AHVCRSSR*VHGLYSR
RGVGDP*RSVRYRLYGLRVFVA*ARYSEDQYFL*GHR*PCGGEENVSR*AQQPRCVMCSLRNR*Q*TNAARGITRCPDPCGSLSGDDRWSNVD
GFCDGRRDTTATR*SNNSAHCTSGK*VTRCFCDR*RDCSS*SPSRSGAEERRKLPLASI
>NC_000913.3_cds_NP_414752.1_206_1 [gene=yafT] [protein=lipoprotein] [protein_id=NP_414752.1]
[location=237335..238120]
MNSKKLCCICVLFSLLAGCASESSIDEKKKKAQ-SHTK*Y**KHSPATDRQRFIR**NHSGRIRRGYSSCA*WR*VPRSP*FSCNTCSIRQPR
TGNHYAGGDAKILYCFHILWYPGQAKASDL*QKQR*K*ERGCCQC*EYELDAGTAFCGCKRTSESDYCLPGYVADRKI*LCAEINSMVRL*K*
QTH*RYLPALPGTFHAGGCGNR*VGYLVAGEL*I*SASTIARQE*SQYD*YDRAANHAT*AKDL*SDGKRFG*PLSI
>NC_000913.3_cds_YP_001165307.1_207_1 [gene=ykfM] [protein=lethality reduction protein,
putative inner membrane protein] [protein_id=YP_001165307.1]
[location=complement(238257..238736)]
MRLHVKLKEFLSMFFMAILFFPAFNASLFFTGV-KTSIFNNKMLHGNIL*LENVDSVLRFHVVFLFKYSCNFTYDNKIILNQKNEGSQFRHRH
NNTANSYIFTNSYRNSTFNSSFCHRICQY*LSPLR**YGNFPRSNLY*KWHEM**WIYFSQGRFSS*I
>NC_000913.3_cds_NP_414754.1_210_1 [gene=yafV] [protein=putative NAD(P)-binding C-N hydrolase
family amidase] [protein_id=NP_414754.1] [location=complement(239419..240189)]
VPGLKITLLQQPLVWMDGPANLRHFDRQLEGIT-RARCDRSTGDVYQRLCHGSGSFVASTR*RSELDDS*GAAVQCADCRQCCITNGVWFG*P
LFAG*AGRHGTFL**ASSVPHGR*ASTL*SGQCASDCGMARLAYFAAGVLRLTFSCVVAQSQRL*PRPVRRQLACSALSALAGIADGPRD*ES
GVCGGMQSRRQRWQRLPLSR*QPGD*SARRDYRYCRRASGNAH*CGAVDGSIAGI*RKVSGMAGCG*V*VVV
>NC_000913.3_cds_NP_414755.1_211_1 [gene=ivy] [protein=inhibitor of c-type lysozyme,
periplasmic] [protein_id=NP_414755.1] [location=240343..240816]
MGRISSGGMMFKAITTVAALVIATSAMAQDDLT-N*QPCKGRNHQSCI*SDGTRA*AACLGDERRYLYSRTNRNVGR*DVSGDERVQTA*LWL
ATYRCDVVREI*SDDGAVLDY**ENVARETHLAECERCAFD*W*NGVVRGVDRQPGKPSGWL*F*I
>NC_000913.3_cds_NP_414756.2_212_1 [gene=fadE] [protein=acyl coenzyme A dehydrogenase]
[protein_id=NP_414756.2] [location=complement(240859..243303)]
MMILSILATVVLLGALFYHRVSLFISSLILLAW-NSRPRRCWSVVGVGTGASGHYPRAI*LCAYA*VDDFRAGISRFP*GDAADVAH*ERSD*
CGHHLVGGRLVPGQAGLEKAA*LSAAAPDRRRASVSRRPGRRSLPDGE*FPDHP*AGGSAAGVVGVP*RASFLRDDHQKRVRRAGVLGLCPVS
RAAKTLRRERDPGDYRRRAKLIRPGRTVATLRH*RAERSLSAASGAWSGDPLLCTDQPGSGFRCGRDSGHRDCLHGRMAGPAGAGDASDLEQT
LHYAGTDCDRAWAGV*TLRPGKITRRCRRFRHYLCADPNHHAGRGNWSSPLPAERTVPERTDAR*RCLRADRLHHRRAENGRARLADAGGVPL
GRPRHHPAFQLNRRREIGSAGNRRVCSHSPSVQNLYW*DGRD*RAAGAYCR*CLRDGCCGIADYLRHYARRKTCRAVGYR*VSLYPPRAAVDY
*CDGYYRR*RHYARAKQLPGACLPGRTDCHHR*RG*HSDPQHDDLRTRSDSLPSVRAGRDGSGEEQ*RQRVR*TVVQTYRSRR*QQSSQLLAG
PDARFNQQHANRRCH*TLLSAPEPPERQPRPAF*CLDGSAGRQPETSRAHLGPSGGYFKPALPRLCRAEAL*RRRP**SRPAAGALGRTRCAV
SG*TGDG*FTAKLPEPRGCRAAECGDLPDRTSLSGTF*QAGS*SGEDFTSAERHPFPHWSRSVPDAERA*SGWLAGRGAGGCDCRRPNSSADL
*RAG*KPAVYPSG*TGAQRAGEGAD**R*SRYSGES*RKPSAQY*R**L*SGRAGDEAGKVAGESAES*SRV
>NC_000913.3_cds_NP_414757.1_213_1 [gene=gmhA] [protein=D-sedoheptulose 7-phosphate
isomerase] [protein_id=NP_414757.1] [location=243543..244121]
MYQDLIRNELNEAAETLANFLKDDANIHAIQRA-SGPVSRQL*SRWQSAFLRQRRFPLRRYALCRRVDRSLP*KPSGLPGDCYF*R*SYFLRR
**FRFQ*YFLPLR*SGRSRRRCTAGDLHLR*LCKRDQSDRSGA*EGNESDHPDR*RRRQNGWHGGYRNSRTALWLCRPHSGDSH*SDPYPDPV
D*KRDG*V
>NC_000913.3_cds_NP_414758.1_214_1 [gene=yafJ] [protein=type 2 glutamine amidotransferase
family protein] [protein_id=NP_414758.1] [location=244327..245094]
MCELLGMSANVPTDICFSFTGLVQRGGGTGPHK-RWLGHYLLRR*RLSHI*RSTTQL*FPHRQTCPGLPDKILFGGGSYSPG*SGRGGAGKYS
PIYPRVMGA*LDLCP*RTTDGLQITGNRQLPPGRRNRQRKSLLLAPA*INAALPAHTGQHGGGI*IYRLTGG*TAAEGRFQHAAFGRALCNGV
LLD*FTLDHPPRAVWRGNVAGSGCGNRLQLADHTE*CGHGDCNTAADGQ*NLAKDYARRMALILPRGACSL
>NC_000913.3_cds_NP_414759.1_215_1 [gene=yafK] [protein=L,D-transpeptidase-related protein]
[protein_id=NP_414759.1] [location=complement(245065..245805)]
MRKIALILAMLLIPCVSFAGLLGSSSSTTPVSK-RV*AAVDGIPCLHPDLQGRTYARSLRQNGRAISTARQL*NL*IFRRLRAKTASGRFQKP
GRVL*RPA*SVKTRQPLLQSD*YWFPQCL*PCAWL*REIPDDPRRLCFHRLLRNDQSGY**DIPVRYWCAGVWSAERASEYLPVPHDRRQYEA
P*IFQL*GLLGATEAGLRLL*ANP*ATNRFCGQWSLRGQQAVKPRSCAATTGIKLHAPRGKI
>NC_000913.3_cds_NP_414760.1_216_1 [gene=yafQ] [protein=mRNA interferase toxin of toxin-
antitoxin pair YafQ/DinJ] [protein_id=NP_414760.1] [location=complement(245961..246239)]
MIQRDIEYSGQYSKDVKLAQKRHKDMNKLKYLM-NASYQ*YFTASSCL*RPPAARFMERLSRCSCRTGLDPDLQTYR*TFTI*ENWNSRGALW
V
>NC_000913.3_cds_NP_414761.1_217_1 [gene=dinJ] [protein=antitoxin of YafQ-DinJ toxin-
antitoxin system] [protein_id=NP_414761.1] [location=complement(246242..246502)]
MAANAFVRARIDEDLKNQAADVLAGMGLTISDL-SSHNPHKGRA*KGIAV*FTRA*SINHSINQKQRSWH*CS*GQRRR*FI**IRNL
>NC_000913.3_cds_NP_414762.1_218_1 [gene=yafL] [protein=putative lipoprotein and C40 family
peptidase] [protein_id=NP_414762.1] [location=246712..247461]
MSLPSIPSFVLSGLLLICLPFSSFASATTSHIS-IQLRRPPADAKPCAFIKTVPNSSEKAGQLYCGRQCRKQKGATPAQPGADKTASRMVSCS
AQGE*QTLAGAGGKQPLFKQRPSA*HYRSGDSPPGAAAWQALRLGRYAA**RL*L*RVGFLCLQQDP*G*APAHGQ*DVPLSPGNDCGEQRPA
PGRFAVFPYPQPRDSRSYGRVFGRWAIYRVATYRRNHSDKPISRTFLAGPFFGRAQDFDGRDDFV
>NC_000913.3_cds_NP_414763.1_219_1 [gene=rayT] [protein=RAYT REP element-mobilizing
transposase; TnpA(REP)] [protein_id=NP_414763.1] [location=247637..248134]
MSEYRRYYIKGGTWFFTVNLRNRRSQLLTTQYQ-NAPSRHY*S*ARQAF*NQRLGRFARAYALYLDIT*RR**FFLALAGN*KAIYPCLWIEK
YLATTFLGARHPQYQRL*ASC*LYLYKSSKAWLGKASE*LAILNVPSRCRARVISHRLGGGRNGF*CRGAYHFI

>NC_000913.3_cds_NP_414766.1_222_1 [gene=dinB] [protein=DNA polymerase IV]
[protein_id=NP_414766.1] [location=250898..251953]
MRKIIHVDMDCFFAAVEMRDNPALRDIPIAIGG-KPRTSGGDQHRQLSRA*IWRT*RYADRDGAQIMPTSHLASGAL*RLQRSLKSYP*NLLA
LHLAH*TVVTG*GLSRCHR*RPLPRFCDPHRPGNPPDNLQRAATDGVCGRGTSKVSRQNRLRHE*TQRPVCDYAGRSSGIFTNLTAGKNPRRR
QSLSGKTGSDGAADLR*CTKV*SGDAA*TLWQIWPHFVGA*SGD*RTRC*QRTVAKIRRRGTHDGGRYSSLV*M*SDYRAAVSGT*TPSGKGK
T*FTDCSPGGEIKVRRFSANHPGARLAAAE*S*SNRHRA*NLG*TPRRARCASGGAACDVA*PANGKTTGAGIM
>NC_000913.3_cds_NP_414767.1_223_1 [gene=yafN] [protein=antitoxin of the YafO-YafN toxin-
antitoxin system] [protein_id=NP_414767.1] [location=252005..252298]
MHRILAEKSVNITELRKNPAKYFIDQPVAVLSN-KSPRRISLKCQRIRSVNGHAC*TRGEKAHKGALPSKCCKIRGNYTPR*TIS**YDG**F
Q*L*GI
>NC_000913.3_cds_NP_414768.1_224_1 [gene=yafO] [protein=mRNA interferase toxin of the YafO-
YafN toxin-antitoxin system] [protein_id=NP_414768.1] [location=252301..252699]
MRVFKTKLIRLQLTAEELDALTADFISYKRDGV-IARYIWSRCTLRRLLYLAINQI*ASCSYSSGK*E*SISATVAPIQQNE*RSAFGILSGG
V**ASMVAHCHSET*TS*TGSR*QPNA*NWENGRSVSHAFL
>NC_000913.3_cds_NP_414769.1_225_1 [gene=yafP] [protein=GNAT family putative N-
acetyltransferase] [protein_id=NP_414769.1] [location=252709..253161]
MNNIQIRNYQPGDFQQLCAIFIRAVTMTASQHY-ITTTNFRLGAD*RISLEGETREITSVGCDH*CTTGWFYFPH*TLYRYVIC*P*IHPPWG
CQRFVKTFD*V*IRTYGGRKHNRKTLF*TLWFSDS*AAAR*MPGSVVY*FLYAI*TATL
>NC_000913.3_cds_NP_414772.1_228_1 [gene=pepD] [protein=aminoacyl-histidine dipeptidase
(peptidase D)] [protein_id=NP_414772.1] [location=complement(254259..255716)]
VSELSQLSPQPLWDIFAKICSIPHPSYHEEQLA-RIHCWLGKRERFPCRTRSGR*YPDS*TCYRRYGKS*TGRLTGPPRYGAAEK*RHRA*LH
ERSYPALY*WRMG*SARHHAGCG*RHWYGLCAGGSG*RKRGSRPAGSAADHDRRSRYGRCVRLTGQLVAG*YSD*HRLRRRR*NLHGLCGGYR
LHLQPAFRS*SGSSWF*NLQVNLKRSERRSLRRGNPRWAG*CQQTAGALPGGSCGRTGSAPYRFQRRHTA*RHPA*SLCDHCCRS**SRRPEI
SGEYLSGDPEKRAGRKREKSGLVAGLCSER*SCPDCEISRYLYSSAERHPERCDS*LRCSQRCG*NLPERRCGDHD*Q*RRNSLPDPFTDRQR
*RLRGEHAGFAG*TGWRENRSERRISWLAAGR*FSGDASGT*NLSAPVQQDAEHPDYPRGPGMWSVQKTVSGNGHGFYRANYHRSTLSG*ASS
HRKRRSLLDTAD*TAERNSGEV
>NC_000913.3_cds_NP_414773.1_229_1 [gene=gpt] [protein=xanthine phosphoribosyltransferase;
xanthine-guanine phosphoribosyltransferase] [protein_id=NP_414773.1]
[location=255977..256435]
MSEKYIVTWDMLQIHARKLASRLMPSEQWKGII-SRKPWRSGTGCVTGA*TGYSSCRYRLYFQLRSRQPARA*SAETRRRRWRRLHRY**PGG
YRWYCGCDS*NVSKSALCHHLRKTGWSSAG**LCC*YPARYLD*TAVGYGRRIRPANLRSL
>NC_000913.3_cds_NP_414774.1_230_1 [gene=frsA] [protein=fermentation-respiration switch
protein; PTS Enzyme IIA(Glc)-binding protein; pNP-butyrate esterase activity]
[protein_id=NP_414774.1] [location=256527..257771]
MTQANLSETLFKPRFKHPETSTLVRRFNHGAQP-TCAVGP*W*NHPSLVSHD*PSDVDLARH*PTRNPRRPGTYCDERCRTYRR*FIRYGDWL
PWRQLDL*VGHPGDGVATKSLCGRRSATQWSSLAACGYVVQHCRLSSSERR*PGRASAGFVKPRL*RGRSASTGHDAADGVYRTRRCAHHRLF
AYAERRWPVPDGINVWWSGCDADGLLQPV*TLFCAARHCDADY*YAVGGLFFKMEAHPGLQPVASARLKGAA*RTVGGSHSRRGLWFPFRR*R
CRASGIP*IAASESGCLSWSGSSYPVE*F*VPATGAGNVS*RSGQSFGDA*CFR*SVARGAESLFIKSARIAGTSLPNANVIRLLEERSVQPG
RGLTLNHLIIC*R*IIRDPI*PGVSEF*QRSSGNHRLDRKTLVL
>NC_000913.3_cds_NP_414776.1_232_1 [gene=phoE] [protein=outer membrane phosphoporin protein
E] [protein_id=NP_414776.1] [location=complement(259045..260100)]
MKKSTLALVVMGIVASASVQAAEIYNKDGNKLD-SLWQS*SHALYE**RQ*RWRPELYPFWFQRRNTN*RSTDWLWSLGSRVCR**SRE*YCT
AKNASRFCRVEI*RFGFFRLWS*PGGVV*RGSLDRYVPGIWWRFLGADRQLYDQTRQRSGDVSEHRLLRRYRWPELNPAISREKRKPRR*KAK
RRWLRHVIDI*LWRQRFRH*WGLYQLRSHQRAEPAKPWHRQACRSMGNRSEIRCQ*YLSGNFLF*NTQNDANNWRLCQ*DTEL*SGRSIPV*L
WSASIAGLCLIERERY*RYR**RSGQLYRRRCYVLFQQKYVSVC*L*NQPTG*R*QIEY****YCRGWHDVSVL
>NC_000913.3_cds_NP_414777.1_233_1 [gene=proB] [protein=gamma-glutamate kinase]
[protein_id=NP_414777.1] [location=260388..261491]
MSDSQTLVVKLGTSVLTGGSRRLNRAHIVELVR-TVRAVTCRRASDCYCDVGRDRRRT*APGLPGTASDHRLETTAGGGRAESTDSTVGTAVF
DLWHSRRANAADPC*YGRP*TLPERPRHPASVAR*QYRSGNQ*ERCCRYGRD*GRR*R*PFCAGGDSCGCR*TVAADRSKRFVYR*PAQQSAG
RTD*RCLRH**RTARDCR*QRFRPRNWRHEYQIAGR*RGLPCGYRHHYCRGQQAGRYW*CDGRHFRRYAVPCPGDSA*KP*TLDFRCAAGG*N
HGR*RGNCRHSGTRQLPVAERH*KRDWQFLAW*SHPHLQPRRPRYRPRRQSLQQRCITPYCRTPLARN*CNTGI*IRPGCRSP**HDYPL
>NC_000913.3_cds_NP_414778.1_234_1 [gene=proA] [protein=gamma-glutamylphosphate reductase]
[protein_id=NP_414778.1] [location=261503..262756]
MLEQMGIAAKQASYKLAQLSSREKNRVLEKIAD-RTGSTKRNHPQR*RPGCC*RASQWP*RSDA*PSGTDARTAERHCRRCTSGVQPRRSGGA
GNRWRRTGQRPAS*ASSRTAGGYWRDL*SAPERDG*CRFAVPENR*CGDPARWQRNVSH*RCNGGGDSGRPEILRLTGGCRAGD**S*PCAGQ
*NAAYG*IHRHADPAWWRWFA*TVP*TVDNPGDHRWYRRMPYLR**KCRDR*SIKSDRQRENSASEHM*YG*NVAGE*KHRR*LPARIKQTNG
GKRRDITRRCSCTGAVAGRPCEGGCC*SRRV*R*VSVIRFERQNRQRS*RCHRPYS*TRHTTLRCDPDPRYAQRPAFC*RSGFVRCLR*RLYA
FYRRRPVWSGCGSGGKHTKTPRAWPNGAGSTDHLQVDRHW*LHHSCV
>NC_000913.3_cds_NP_414779.1_236_1 [gene=ykfI] [protein=CP4-6 prophage; toxin of the YkfI-
YafW toxin-antitoxin system] [protein_id=NP_414779.1] [location=complement(263328..263669)]
MKTLPAITQRAVKPCLSPVAVWQMLLTRLLEQH-IWSDNKRHAILQ*GCD*GTHRCRYHPSRCREFSGRKIRAGSYRQEGI*LAGTISLSPGC
RHSASAAGNWLVAAKP*QRSTM
>NC_000913.3_cds_NP_414780.1_237_1 [gene=yafW] [protein=CP4-6 prophage; antitoxin of the
YkfI-YafW toxin-antitoxin system] [protein_id=NP_414780.1]
[location=complement(263690..264007)]
MSNPTRGLQREITLRLGARLVQEGNRLHYLADR-SQHHRQVQ*HRMPEAG*NIPALYPPDGIDADHR*TQPPPCPLRYPVPQRFNLRSRHPWQ
LRLRIHRHLPHSAL

>NC_000913.3_cds_YP_588435.1_238_1 [gene=ykfH] [protein=uncharacterized protein] [protein_id=YP_588435.1] [location=complement(264026..264247)]
MKIISKRRAMTIYRQHPESRIFRYCTGKYQWHG-KRLSLHRQGRSGYRRSPRGIRRTPPGPQWALYLPDEHHPEL
>NC_000913.3_cds_NP_414781.1_239_1 [gene=ykfG] [protein=CP4-6 prophage; RadC-like JAB domain protein] [protein_id=NP_414781.1] [location=complement(264256..264732)]
MKQLSFLPGEMTPQDRRLIQRALRALDRHLHEP-RRSLHLYPRRT*MAATAYGRA*AGRVPGVVSGQPESVDCP*NALHRHD*PHRGASPGGG
QTCSALQRGGGDTRA*PSFRRDDT*PGRQNPHAATGSGASAGGYPCP*PSDCRWQANLFVRRTRSAL
>NC_000913.3_cds_NP_414782.1_240_1 [gene=yafX] [protein=CP4-6 prophage; uncharacterized protein] [protein_id=NP_414782.1] [location=complement(264748..265206)]
MTTQTQHDLAPANQPEFELTVTPVPDEQRIDFW-TTVLWRYPAVATPGAAYLRLDGPLL*GLQRWYLVVLHPQQWRRIYVPRA*QR*DMASV*
LPER*RCPNECRSSRYCCLPDCV*PPCLPYRM*RDDRTLLPPAGVCHATSRGSRHSAYYRL
>NC_000913.3_cds_NP_414783.1_241_1 [gene=ykfF] [protein=CP4-6 prophage; uncharacterized protein] [protein_id=NP_414783.1] [location=complement(265304..265543)]
MTQSVLLPPGPFTRRQAQAVTTTYSNITLEDDQ-RQSLPSGGS*Y*RPDGLAGMEL*AGCR*RS*PLYPHLRHPYRHGHPL
>NC_000913.3_cds_NP_414784.1_242_1 [gene=ykfB] [protein=CP4-6 prophage; uncharacterized protein] [protein_id=NP_414784.1] [location=complement(265620..266087)]
MTILSLSRFMLAGVLLASFNASAIPGFWQQGYG-TGQYGIQRDRSQRKDVYHQLHREPGPEWFLSAFSLSYPCR*QDGQFAR*RHYYHRSDGS
PAVHYSVQPGLA*RR*RLV*LHQQYL*GRAVRRLRQ*PQSRDLHCGPEER*ESSVHSRRLQQRL
>NC_000913.3_cds_NP_414785.4_243_1 [gene=yafY] [protein=lipoprotein, inner membrane; degP regulator; CP4-6 prophage] [protein_id=NP_414785.4] [location=complement(266110..266553)]
MKRKTLPLLALVATTLFLIACDDRSDDLKAISK-I*GPHPTAFQ*CGQPPG*CQRRMVTG*LLIRSHLAGFTYPPVARWLRGWQLLLPRGYAG
KNRPATDECAVYCR*HQTGIPGGDGPVYQRKIL*VRP*RQTDGAATDTLEP*QSGI
>NC_000913.3_cds_NP_414786.4_246_1 [gene=yafZ] [protein=CP4-6 prophage; conserved protein] [protein_id=NP_414786.4] [location=complement(267184..268005)]
MTRLASRFGAANLIRRDRPLTREELFRVVPSVF-K*GQTRVP**ALYLYTHHLPARQPTARRLPAILCLSDPRA*PGSS*TYKAYAASAAGRA
DHR*TGAGNYSTQLSRWNQFVSDVAGTISCGLSERARLR*VVWRGAGATQGGRGESGD*RRV*GAGDF*PGGGETGCHAVVAVATPGTAGTGK
SRPHIPLW*RPPAGD*IADPLPSPLAG*EQ*PVDHVPAYSGEPD*GRAQWP*CQRRTVTYPCRSRYRRGRET*PGTVGDGGSPAHATAV
>NC_000913.3_cds_NP_414787.4_247_1 [gene=ykfA] [protein=CP4-6 prophage; putative GTP-binding protein] [protein_id=NP_414787.4] [location=complement(268097..268960)]
MNNSEGLKSFQQSLADLPQWVSERLLQQINQLT-KLRASDRHYG*NRCWEEQSVQCPVCRRYITGQRCGGLYT*ATALSPASRRPLYNADGSA
RRGRKWRSRYRVCCAVPRTASSPRPGTVAD*GR*PGADGG*TFLSSGDWRGIPA*GAVCYQPVG*GRTYQRWGTVVHGAEAEYQPQNLPAA*A
VPARPSGVCCVGPSTMGA*GDGRADD*VPAT*GHQPGGVATPSFLSHDGSPGTGS*RFW*NRRGRTR*HQCLSPDTRPGAGRHSGRAHHGGVS
GPRRLGFLLL
>NC_000913.3_cds_NP_414788.1_248_1 [gene=perR] [protein=CP4-6 prophage; putative DNA-binding transcriptional regulator] [protein_id=NP_414788.1] [location=complement(269289..270182)]
MKLLAKAPLNLLRAFEAAGRTGAFALAASELEL-ITQCDQPCHPQTGKLA*CTPFSAQYARNYADERR*NTA*AHTAGI*RITAGVGISDG**
IQAFAPSYCTEFCPSMAFTTSW*VHT*EPKHRSATFSQHRICTF*TG*F*SRYSLR*TPPITL*EDPACC*RTYATVFSPTG*AAKETRGSLC
VDIDSVRCAVVPVERMV*GE*DDATQ*LWPSI*P*LYGDCCRS*RLGSCA*IKTTG*T*NR*R*TGMPTCKQYQRNTLHRSLFGFPPAPTHAF
CA*CVQNLATE*TQSG*NSL
>NC_000913.3_cds_NP_414790.1_250_1 [gene=insI1] [protein=IS30 transposase] [protein_id=NP_414790.1] [location=270603..271754]
MRRTFTAEEKASVFELWKNGTGFSEIANILGSK-TRNDLHYVKGYWRHKTP*A*AGCSSPDTV*ARGDTSWFVSQNEHSCDSYCAESQSFDDL
T*SSA*SGQTLLQSC*C**PSQQNGEKAKTVLTGSKFTIAKACSGKAGDEMVSRANIRMVKANKTTSKNAANIT*DNL*NAVLS*P*SATPPE
YTASATVA*PSPWQASYPQRRKRYD*HSERNTNSRTFPKYR*QTLSGALGGRFSLRYKKLSYSHTCRPKITLYDHR*TQGQRFCLSKSGSYRQ
IPEFTVRTQKITDMGQRNGTGQTSRIYCQHRR*SLLLRSSESLAAGNK*EYKWANSAVLS*KDMSCPIYST*TRSGCCSAKQQTEKDTEVQNT
ERDN*KGCCIDRL
>NC_000913.3_cds_NP_414793.1_252_1 [gene=insH1] [protein=IS5 transposase and trans-activator] [protein_id=NP_414793.1] [location=complement(274101..275117)]
MFVIWSHRTGFIMSHQLTFADSEFSSKRRQTRK-RDFLVPHGADSAMAKHGGSHRAVLPQGW*WPATLSAGNHATHSLHAALVQPERWRDGRC
SVRNRLHASVCPVIPG*RLAGPHHHHEFPPPAGAASTGPPIVQDHQSLAGRSRRHDDSRHLGRCHHH*GTQLDQEQRAATRSGDASDQERQSV
ALWHEGPHWCRCQEWPDPQPGHHRGQRA*PQSAG*SAAWRGAICLSRCRLPRGATARGAGRGGCGLADRRAPRQGKNLETASTQEQNGHQHRI
HESQHPGQGGAPISHHQATVRLRESQIQGVAEKR*PTGDVIHAGQPVSGGPNDTSVGEISL
>NC_000913.3_cds_NP_414794.4_253_1 [gene=mmuP] [protein=CP4-6 prophage; putative S-methylmethionine transporter] [protein_id=NP_414794.4] [location=275325..276728]
MQTTQQNAPLKRTMKTRHLIMLSLGGVIGTGLF-IQYRVHHFHHWSGGNAAGLSDWCAGGLAGYAVSGRAVGRDAGDRSVSRLCRALSWSGYR
VYRGLALLADLDRGAGFELYRRWILYAVLVSTGAGMGLVRGVLRDYFWSECYLHALFCRRGVLVLAGQSGHYHRLYHPRWGGDFRLYSDAGWL
ARAGAE*YHGRRLVPARWLTDFDDYGGSELCFFGYRAYRHCRR*NGKPAQSYPGSDSYYHRATDYFLYRHRVCAGSADPDAAGGRGEKPVCAG
I*ESRDPVRR*YF*LRDPDGYSFCSELRVICLRAHAVVVVE*TYATGLFCASNEKRRATDGAVGQYARWCAGAVFQRGGPGHGICCAVGNLRV
CGGSGVAEYLRLAFCFSSPSSATR*GIE*ITLSRAVVSAGASIRFCAVPGGLCWAGIRSSAENCVVVRVTVCCVVLWCLFPYSTPKRKTGART
CRRI
>NC_000913.3_cds_NP_414795.1_254_1 [gene=mmuM] [protein=CP4-6 prophage; S-methylmethionine:homocysteine methyltransferase] [protein_id=NP_414795.1] [location=276715..277647]
MSQNNPLRALLDKQDILLLDGAMATELEARGCN-ISRQPVVSQSAGRKPGAYPRSAS*LLPGGGAMRDHCQLSGDAGGLRRARSG*SAVESAD
WQKRGAGA*SP*SVSGREPAGGNASGGGIRRALRRVSGGWL*IPWRLSL*R*GISGVSSPARGSLAGCRGRSAGLRNPAEFFRD*GVGRAVDR
ISACAGVVLIYPARQRTPERRYAAA*RGCVAGGLSAGGGARH*LYCAGKHHRCVAAFTRFNGAAAGGVSELGRALRCREQNLASSR*TLRAAG
GLSAAVAGRWRTVDWRVLSHHACGYRRVKSAKL

>NC_000913.3_cds_NP_414796.2_255_1 [gene=afuC] [protein=CP4-6 prophage; putative ferric transporter subunit] [protein_id=NP_414796.2] [location=complement(277756..278802)]
MTQKNFVELRNVTKRFGSNTVIDNINLTIPQGQ-NGDAARPVRLRQNHYFAPGCRAGKTERRANFH*WRRRHPSLYSAARYLYGVSVLCPVPA
YVAGRECRLWPENARRTARRAESPRQRGVGDGGSGRIRRPLCRSDLRRAAAARGAGPRADPQAESAAV**AVE*PRRQPASQHARQDPRVAKA
V*YHLAVRHPRSERSLCGF*YCAGDEQGTHHADRLTAGSLPPARLPLYGELYGRCQPVPGNLQRRIR*YLRLSSAAPAALWYTG*RDGRCAPG
SDHAQRSRRREPALRDPPCRLYGAAV*SDGGMARAGDIIAGQRYASATGRRRAVLS*NPSVRHVCSGGCGI
>NC_000913.3_cds_NP_414798.1_257_1 [gene=insB1] [protein=IS1 transposase B] [protein_id=NP_414798.1] [location=complement(279178..279681)]
MPGNRPHYGRWPQHDFPPFKKLRPQSVTSRIQP-RQ*RHRLRGNGRTVGIRRG*IAPALAVLRV*QAPEDGCCARIR*THYGDAGASYEPAVT
L*RGDMDDGWLAAV*IPPEGKAARNQQAIYAAN*AA*PESEAAPGTAGTEVAVVLKIGGAA*QSHRALSEHKTLSI
>NC_000913.3_cds_NP_414799.1_258_1 [gene=insA] [protein=IS1 repressor TnpA] [protein_id=NP_414799.1] [location=complement(279600..279875)]
VASVSISCPSCSATDGVVRNGKSTAGHQRYLCS-TLP*NMATAVHLHRFSTRYAPENH*YGHEWRWMPGNRPHYGRWPQHDFPPFKKLRPQSV
>NC_000913.3_cds_NP_414801.1_262_1 [gene=yagA] [protein=CP4-6 prophage; putative DNA-binding transcriptional regulator] [protein_id=NP_414801.1] [location=complement(280829..281983)]
MESLMPWDARDTMSLRTEFVLFASQDGANIRSL-MPSLRHFTCHRLQVAPALGSGRCRRSSGPPAHSAPFPEPLI*RHHGPAAYGP*PS*TLG
SPQD*ALARGPGAHHARLQHRP*PDGPPWPAAGRFTGHSRHGPVRTRRAEPPLADGF*GPLSFWRWTLPSAHPAGRPLPFFPVPGALYR*TAR
DRAAAAGQRV*ALRPAGPDDHG*RLTVGRHHRHLDGAGAVADAPGYPGGALPALSSADAGEAGAFSPQPEGGSAAGKMVRRQR*TAARLRPLA
DGL*P*TPARGAGYGGTGLAVSAVSAAVQRQHNAPGIR*RGDGQESGYQRKAERERGKSERRQGVQGRTGRAEGDAGRRQLRGVVVQHESGGD
RPEEKVDHHG*RML
>NC_000913.3_cds_NP_414802.2_263_1 [gene=yagE] [protein=2-keto-3-deoxy gluconate (KDG) aldolase; CP4-6 prophage] [protein_id=NP_414802.2] [location=282278..283186]
MPQSALFTGIIPPVSTIFTADGQLDKPGTAALI-RRSDQSRR*RPVLPGQRWRVLPARRRRA*SHCPLCYRSCRSSRAGADRHRRHQRPGNHR
TQPARAAGGRGRHRGDQPLLLESVGSEPDPLFRAGGRQRHAAGDAL*LPGADRAGSDSGAGENPRRLAQQYYRHQRHHRLRRPPAQHDPYRQR
CPSALHRALRLRRSSVQYPAARRRRGDIGERQLCPAGVGESSESLARRGRGESGRVSSDLAANSADVSAGYAVCERD*RGDRALRSSCLHARA
AARLAAGRAAQGAAENPAATAQALL
>NC_000913.3_cds_NP_414803.1_264_1 [gene=yagF] [protein=CP4-6 prophage; dehydratase family protein] [protein_id=NP_414803.1] [location=283201..285168]
MTIEKIFTPQDDAFYAVITHAAGPQGALPLTPQ-NADGISQRQPVRHDAERRDGLGRQQAHRQRGADYRHSGRHPRRRRTPNRAGLPHRALGD
RHADAGGGEGDHPQWRDPVRGLRQRSVRRALAGHARYVRFPAVPQRRGDRVSPPDPLPADAAGGDRRSDLR*RAARHHDCAGRDARPADYSGA
GRGDAAADRRGRRGQGADHRRAFRQPRTLPAGGRRTGLSRLRLAGRRVSVPRHGGHLAGGRGGAGSGAAALRAGAVRAGGVAGDRPPVGARGQ
RAG*PRHHHAGYPLR*SHRKRDGDPRGVRRLHQFTAAHSGHRPRGGLHDPGR*ALDAHQP*SAASGERAAQRPGLSPDRARLPRGRRAGGDAP
PARPRPAASGRHDRDRPDGGREP*MVAGVRAPGALPPVPARAGRRRAG*RDPAAGEGKSERADLDGLLPDGQHRSGRFGDQGHGDRPVGGGRR
WRIPPHRPGAGVCLGSAGDQGDQAGRDCAGRYHGGDRRRAVRHRHGRDLPAHLRAKAYLVGQDGVAHHRCALLGRVDGRLLRPRVAGGAGGRA
DWQAAR*RHHRDCRGSSDVNWQRELHRHRGQPADAGRGRARAGAAADAPGPARPRLFAGRHPAVGGTAVGERRHLERLYL*HR*NYRGN*RR*
KSARNL
>NC_000913.3_cds_NP_414804.1_265_1 [gene=yagG] [protein=CP4-6 prophage; putative sugar transporter] [protein_id=NP_414804.1] [location=285395..286777]
MTQLTMKDKIGYGLGDTACGFVWQATMFLLAYF-IHRRLRPVGGDYGHAVFGLPRARRRHRPADGAAGRPHPHAARPVPPVPAVGGHPVRHRL
RADLLHAGLLRTGQDHLRLRDLHSPDPGLHLR*RAVLRHAGRHHRRPERASRPAVLALLPGGGGLARYQRHRAAAGEHHRQRGRAGGLLRRHV
RAGAERRGAALRLLLHDQRALHL*GAAGLVGGERP*AAAGQQPVAHHVRVQDDGDLLQRGARRGDALLREIRDGSPGVGDPVFTLRQPRHHVR
LALLLTPAGPLRPRHRLQVDHRRLLADQPADFRHPGGAHRAHFCPQHPVPVRL*YHHAAAVADGF*RGGLRGEPQRSPPRRAGVLHLPVQPED
WPGDWRGGGGLDPGVRQLFRQQQRAAG*GAHHHQNSVLRGAGGALRGHVHHAVALQAHRCPRGGHQPAAD*APRGAGRGRSRRRDSRIPL
>NC_000913.3_cds_NP_414805.1_266_1 [gene=yagH] [protein=CP4-6 prophage; putative xylosidase/arabinosidase] [protein_id=NP_414805.1] [location=286789..288399]
MEITNPILTGFNPDPSLCRQGEDYYIATSTFEW-IPGRAHLPLP*PEKLVAGQHPVGPRVDAGHEGQPGLRRHLGAVPELRRR*ILAALHRRE
DCRLAVEKRPQLPRHRALHRGAMERANPDGQRRV*PVPVPRRRWPQILYLPPVGAAPPQQPAQHHRVTGV*PADRHALARAQNAVYRHAALLH
RRRAPVSPRGMVLPDGRRGRHQLRARRRGAAFQKYRRAVRAAPGRNDDDQLAPAGEPAAEERPRLAAADAYG*MVHGLPHQPPAAPARRAAAG
LRRTRLLPAGARDRHRPH*MARRLAVRGRRQARAADRERPASSRAACSRSGQLAGRFRRQFA*PGAADPAHSVRRHPRLAHRAPGLLTALWQR
LAQFDLHPIDRGAPLAALRLPGRNADGVLAGALPAERGADLLLQQQKLELLLCGLRGGTG*NHQSYPARPQRAVVAAARAAHSGAGTCGERLA
AGGRGYAGLPLQLLV*WRDVAHRAGDV*GVEAVGRLHRRARLLHRRVCGPALRGHQRRRLLRGLRLLHLRAGL
>NC_000913.3_cds_NP_414806.1_267_1 [gene=yagI] [protein=CP4-6 prophage; putative DNA-binding transcriptional regulator] [protein_id=NP_414806.1] [location=complement(288404..289162)]
MPIIQSVERALQILDLFNEQATELKITDISKLM-RAEQEYPPLAAKNPAASRLYRSEPGERQVSPRHEAGRARPFCRGLHRYSAEGKRLADGA
VPADRADHPSGDPGRA*RGLYRED*RQAGRHRLFTHRPPPAGARHRHRQGVDCLAGRGRAERPAGGLSVHYLYARHPRVSRSLNERPGADPRA
RLRPGQRRERAGRALRGGAGVEPRVPRHRRPEPVDADLPRGRRGAG*FPRAASAGRARALARAGLPGL
>NC_000913.3_cds_NP_414807.1_268_1 [gene=argF] [protein=ornithine carbamoyltransferase 2, chain F; CP4-6 prophage] [protein_id=NP_414807.1] [location=complement(289301..290305)]
MSDLYKKHFLKLLDFTPAQFTSLLTLAAQLKAD-KKKWQGSTEAYR*KHRAHLRKRLDSYPLLFRSCRI*PGRARYLFRAERQPDWA*RVN*G
HRAGSRADV*RHSVSRSRPGSGRNAGAVCGRAGVERADQRVPPDPAAGGPDDHAGAPAGQGV*RDDAGLRGRCAQQHGQLDAGSGGADRAGSA
PVGPESLLAGREPGGGVQRAGGEARRENYSDGRRGGRR*GRGLYLYRRVGVDGRGQREVGRADCAAARVSGERADDGADRQPEREVPALSAGV
P*RPDYARQADGEGVRSARRDGGDGRGV*VGGEHRVRPGGKPDAYD*GGDDGNAWGV
>NC_000913.3_cds_NP_414808.1_270_1 [gene=insB1] [protein=IS1 transposase B] [protein_id=NP_414808.1] [location=complement(290649..291152)]
MPGNRPHYGRWPQHDFPPFKKLRPQSVTSRIQP-RQ*RHRLRGNGRTVGIRRG*IAPALAVLRV*QAPEDGCCARIR*THYGDAGASYEPAVT
L*RGDMDDGWLAAV*IPPEGKAARNQQAIYAAN*AA*PESEAAPGTAGTEVAVVLKIGGAA*QSHRALSEHKTLSI
>NC_000913.3_cds_NP_414809.1_271_1 [gene=insA] [protein=IS1 repressor TnpA] [protein_id=NP_414809.1] [location=complement(291071..291346)]

```
VASVSISCPSCSATDGVVRNGKSTAGHQRYLCS-TLP*NMATAVHLHRFSTRYAPENH*YGHEWRWMPGNRPHYGRWPQHDFPPFKKLRPQSV
>NC_000913.3_cds_NP_414811.1_273_1 [gene=yagK] [protein=CP4-6 prophage; conserved protein]
[protein_id=NP_414811.1] [location=complement(292322..292948)]
MKTYQGTHGVHILEYQSKINKLLCYLTNRYPRL-NCCTCRSPLSQNS**R*QYLLLP*PGTWGDIPDA*IPQS*AGG*PYP*GA*G*THLPLP
FIYYMGERVF*IR*MPLSHLPVVQQRRLLPPRGLRSGR*P*RDDHRSLVQRPETGERRSPWSGPFS*KL*VCARHQ*Y*LPAELPGAVNPTGL
PDQS*KQSVW*RRP*LRL*PNRTV
>NC_000913.3_cds_NP_414812.1_274_1 [gene=yagL] [protein=CP4-6 prophage; DNA-binding protein]
[protein_id=NP_414812.1] [location=complement(293220..293918)]
MRGKILLYQLKYRWQSLSIFGCFLCKMTLFRYQ-KNNL*HRSASDAFIFLHHMQ*RTARKHYRSSLTC*DMPKI*YST*TCCDRAGRY*RGCF
RATFTQTIDSS*NEPAGHACHSRSKLSWQNCGGLTEYFIFLPSKRNVYLQLPSSFKDRAFC*KLLVFFDCSSGYD*HSQSEIN*KPISTC*EK
IRTKGGKQIPT*YHNT*ERWIYSG*DCKEIEYQFIDCEAALE*RNHRL
>NC_000913.3_cds_NP_414813.1_275_1 [gene=yagM] [protein=CP4-6 prophage; uncharacterized
protein] [protein_id=NP_414813.1] [location=complement(293945..294799)]
MSNSVTNFEMSSVLPGKKPCQGKNNESQVVQTT-THKKTLSHVQKSIFIRSN*SLCQTNK*ISLFRNSGSCGFGYPYIRKMQSS*LVNK*NKK
*PVKVLYKRKHQSKPR*NRSNSGRVLFVNLENEHHESIKNPHCRLLSTER***IHGER*KNSYT*KAILAKGKLRYGKSHLHLQSKTF*CKAS
KCLRIFKHYSYS*NNL*GLLL*CRAGSTLVNIPIDYIRDK*SS*KKGDCYALSGCLLD*YLPCQ*NGGYAASAPQNRCFQPC*CTG*HHYKPI
LTREQNL
>NC_000913.3_cds_NP_414814.1_276_1 [gene=yagN] [protein=CP4-6 prophage; uncharacterized
protein] [protein_id=NP_414814.1] [location=complement(295139..295579)]
MATPATVSIEPTLAAIRARWCINSSKTTQSFND-TCVHGRGCRVSQRNILSSSQVCRMRQTENFTS*TKNAKCY*LSRASDVM*KSGIQIASQ
YG*IS*KCLIIRFR*W**PRPRARNVSFCLHYYPY*FNNA*SFLCQYLNH*GGKM
>NC_000913.3_cds_NP_414815.1_277_1 [gene=intF] [protein=CP4-6 prophage; putative phage
integrase] [protein_id=NP_414815.1] [location=complement(295696..297096)]
MFIPSIYLHQQLHYCKTAILNWSRKMALSRQKF-NLRKTSQIHLTGREKTNFSLGCRCNNPGMPSN*RSKSLCIPKRICGENPSHDYWQH*RL
ED**CESRGKTVTNIDRYRDRSTNC*GCKNRRSRIPAGRIT*NKSDFLRRLGRLSSRIENRYQCKN*TPIFYSIHCRSH*LVQSWRRK*KKRP
RPDFGWTIG*FAQPAVIGANPRLHSSVAEYRKAK*TYRHCSRLSPTTCFHQME*LSEKISRDHSWRSGTRLQRKKNGSRVSE*S**LPAKGTT
KKLV*CRA*PQ*SYCIGLSPSTFAHWCSA*RNCVASLVRRRFQMVKHAN*RQDRR*TYHPSHSLCF*IVKCTSAIPKF*RK*GGLGFQK*Q*K
WQNY*AAFSAQQSISAG*VTTYQPSRFTS*FWYFGRVG*SSHWYCCSNYGTQTQRSCRKTLSPSSVRSVTKMAREN*DMDLK*SRYYHKKQR*
YAL
>NC_000913.3_cds_NP_414817.1_280_1 [gene=paoD] [protein=moco insertion factor for PaoABC
aldehyde oxidoreductase] [protein_id=NP_414817.1] [location=complement(297770..298726)]
MSYPLFDKDEHWHKPEQAFLTDDHRTILRFAVE-SANVR*RSGAGDAGGDTRRRGAPARGADGGARRWSLLRFCLWRLRGGRCRF*SAGDDGL
RPRSRNSLWRRFAVV*HRSALRRWDHADAP*TTLGTASARRAEPPGTEKTGGAALRSASTIAGVPAHANPDGLESQWL*GGVQAMRQADDLRT
FS*GAGNRESCSSHRL*QPYLRSFSGLSQRSDRYRYGGHFAVP*SQPGAASVAGRARSKTLLSRRIGQLSNPHVTSAKAPRAGMVQGGNNANP
GTRRDIPQSPGCAYSGTLRAGRSRLCTSPSGGGFMPAPVVL
>NC_000913.3_cds_NP_414818.1_281_1 [gene=paoC] [protein=PaoABC aldehyde oxidoreductase, Moco-
containing subunit] [protein_id=NP_414818.1] [location=complement(298736..300934)]
MKFDKPAGENPIDQLKVVGRPHDRIDGPLKTTG-NGTLRLRMA*RSPQRRLWLYRRFRHCQRTPHRP*YGRRAKSAGRTGCHYRQ*RRGTRQR
RQKHRQAVRRPHY*ALSSGHCAGSGRDLRTGASGGLAGAGTLSP**RSLLPGGRKTGRQSAAGRHARQKRR*L*RGFHLRCGED*CYLHDPGP
EPYGDGAACLDGRLGWK*AYPLDLKSDD*LVPHRSGKNAESSRGECAYYLPVYRRRVWRQAVPEKRCAAGGPRRPSGETSG*SDAPPPLYSQ*
HHAPPRHPSALAYRCRPERENHRYLT*KLVWKPARRHAGNGGTAKRITLRRGESSYRPAARHA*FAGRERHACARRSPRSDGARNRDRRTGGK
SGHRSRRVSHPE*HSG*PRRPDALLLSPSAYRVLAHRSG*IWLEAAQRHTRTGARRGVASRPRCCGGLSQ*SAGKIGCSGSPRTKRHRYRRNG
HDRHWHRQLHHSGPDGSGNAWRTAGAGCGSPRRFQFPGFCGFWWTMGREYLHLRRLRRLYEASRNDCLGSRV*S*AVAVCRRQDYQRYPKRHA
T*SHRRRQTDSGREH*IRNTEQRVPAVDLCRAFCGGRRA*RDGRSSGPAYARCVCCRTHPESENCAQPGHWRNDYGHGRGTDGGAGGG*PFGL
LR*SRYGGV*GAGSCGYPKTGGDFPG*YRPHILPDEGQRCR*AGPVRRERGYRQRGV*RHRYSGTGLSHHSG*AAR*AAGCGL
>NC_000913.3_cds_NP_414819.1_282_1 [gene=paoB] [protein=PaoABC aldehyde oxidoreductase, FAD-
containing subunit] [protein_id=NP_414819.1] [location=complement(300931..301887)]
MKAFTYERVNTPAEAALSAQRVPGAKFIAGGTN-TAGPDEAGN*NAHPPYRCERPRAR*D*SDRRGWAAHRRTGTEHRPGGSRARAS*LRGTL
PRPARWRVWSVT*SGNHRR*SAPAHALPLFLRHQSALQ*APARERLRGA*RL*PSARGGRRKRSLHCHPSERYGGRNAVAGCGGGNHHAGGKD
SQYHTG*FLSPSGKNAAH*NRPASR*AYRCGDVTSTARRKTYLP*GARSRLLRLCPGIGRGDYSA*RQRARRAGRSST*ALAH*GCGCSAIPG
GAGRI*HAVRQRPSHR*KHL*TPVGEANACLRTG*SEGTGM
>NC_000913.3_cds_NP_414820.1_283_1 [gene=paoA] [protein=PaoABC aldehyde oxidoreductase, 2Fe-
2S subunit] [protein_id=NP_414820.1] [location=complement(301884..302573)]
MSNQGEYPEDNRVGKHEPHDLSLTRRDLIKVSA-SNSGDRRGLSSFYAGGKRSGSYTRARDNAPDTEGERQNRAA*GGYPNHATGHFA*KSAF
DRYQERLRSRTVRSLYRAGQWSQA*CLPDACSHASGGRDHHH*RPGFAR*SSPHAGGLYQA*WLPVRLLHLRANLLISSGAKRDSGRHSQSRH
GRFGFRSRNNCR*DP*TYERQHLSLWCIR*HPCRH*RCCGGDKIM
>NC_000913.3_cds_NP_414821.1_284_1 [gene=yagU] [protein=DUF1440 family inner membrane acid
resistance protein] [protein_id=NP_414821.1] [location=302991..303605]
MNIFEQTPPNRRRYGLAAFIGLIAGVVSAFVKW-RG*SSIAAT*PGGYV*CSVWPGIINQGCRPN*LLA*FSQSTVYFSSRLVGADRSQCGCL
YLCRACL*LGWCYAHYLFDSVCCRLLCGR*SISKN*TLAGLTGRCFSPTFCSYDFIPSHGTDATSV*SPVV*ECF*NFWTFSLVLVY*NYSQR
FTKQNYS*ARP*DPFRLKQI
>NC_000913.3_cds_NP_414822.1_285_1 [gene=ykgJ] [protein=UPF0153 cysteine cluster protein]
[protein_id=NP_414822.1] [location=complement(303853..304182)]
MSNLNPCMTCGACCAFFRVSFYWAEADDAGGTI-TRQAH*TNIPFSPMHERYQSEKPPMYCPCRNPGQKCLLHDI*KSIVHMQRIRHVW*ERS
RQ*GLQSCKG*IRADTVI
>NC_000913.3_cds_NP_414823.2_286_1 [gene=ecpE] [protein=ECP production pilus chaperone]
[protein_id=NP_414823.2] [location=complement(304495..305205)]
```

MFRRRGVTLTKALLTAVCMLAAPLTQAISVGNL-NIFAAVRD*LCQQTCSE*QQKRTDIPYCHQCY**PGQQ*IAHPTGGW*TAFRPPPAGVA
GW*ERVF*ILLSWSTG*PRALLPGLISRGPHS*PYKTQPNRRRGQHGAGGGDGYHSGSTTTSGSV*MVLRQGDRNGK*HWQHMV*ATD*ARM*
FDRRGRRCLVSTSGRRGSSA*VTSAGESLSGL*RQIH*D*RFLSG*APFGGL
>NC_000913.3_cds_NP_414824.1_287_1 [gene=ecpD] [protein=polymerized tip adhesin of ECP fibers] [protein_id=NP_414824.1] [location=complement(305174..306817)]
MRVNLLITMIIFALIWPVTALRAAVSKTTWADA-TGTRVCVCRKQLRRQLFRHSWRGAGSAPDRCQPLDRFKIHWFRNHLSAKPRLH**RLQH
RPLYQLEV*YVAGKFTSFISFNWLALHQLVRWV*YDHQSYPAANHRRQWILWRDGDQRRREVDARHVVRRVLPVYATNARRQQLYNDHQCLPD
LCEL*RQQRRTL*GSGLRQLVCSQRHPYESSKSTVDKYPLAGGSIYQQRRSTDSGRRERRLPDANHRQPFRIKL*DG*LYPANKRTQQHLNPY
IPGDRQLVVSLGRRGVRYAVQSEWQFMETGEQYRLLLHLQRDEERRLDLCFLLEQLL*ADGEPRDQRYQHQRSIQLSLSEHHITGVWLV*IFY
LQHADYQTP*FQHQYYLR*IYSDTVAGGICWQRRVGTRFRLYRNHQR*NSCRRSADQGDRTRAGDWRALLLCLQLR*R*GESTVPGDTFLYYP
QRSYKNLRCRVR**LAGYDRCAVVDHTVD*YLWRSGADG*DHSQIFDSNG*RHFAAYGR**RLVWRSQRFRRDSCSGDVA*H*L
>NC_000913.3_cds_NP_414825.1_288_1 [gene=ecpC] [protein=ECP production outer membrane protein] [protein_id=NP_414825.1] [location=complement(306807..309332)]
MPLRRFSPGLKAQFAFGMVFLFVQPDASAADIS-SAANRWGDYSAGLQSGASGRHERPALYSSRR*PGSPGRSANRQRFYLAGRWTATHPENT
AGRE*R*RQCQRTNSTAADGSGQRPVQ*GPYHPPD*QRAAGSQLAPTAAAASGQARSAGHRTTLT*RRHRAVQC*HPQQ*SEL*LGRL*QPVA
*RREQHIQLSVAE*RYCTARTSCGARRLAVRDR*RSTGQ*II*SDV*TRFCRSPICRWNARHLELAVLRADDRHFSREDLRPFLGKPGQLHHL
RQQPVSHASDRLFTGGGRSTSHP*WAATKRSELHYGQS*SGYPGSTVRDLRCGS*GDR*RSRDQQTHPAGQ*AV*PGARRRCTTGVAGMGR*L
SYGSLVGKREKDATS*RELASRCLDLRLIEYA*LGGNGIWIR*SGGG*NPSDAAAWGGDQR*PAKYAGQ*QLMEQHRQHQRHSTGRL*FAVG*
SGKNPHWQSIAT*RCRQPCNRRHTQPELTVVEAGHIQHQLQ**PPLQQPLLHGRLLSKCLQRYLWFAWPAGRYSAL*QRRQQRQYRKIYRSRS
LATTGQLV*RRDDSSKRLHHGKPVSTQTV**RNHSHCWCQSVTCHLRRYR**QNPQRRGVCTIRRSLRQRNAERQ*RGGRLRQYQLDRQWQRR
LAG*KHCCQRAD*WQRWGDIQHRAGGRRSDQRQNQRADFPA*RQA*LSPALSLWKI*GGVTEQQKLTRQLRYRQRPQKSSDSLSRQCRCH*AR
GEADGYRLRSYPCGRRHTAG*RTD*QPYRPNPNR*KRRVCHGRG*EIPHYRFSLQWQ*NLRSGSGTQPGARCRLGR*CGLQRPLIVGGGDADR
RRE*EL
>NC_000913.3_cds_NP_414826.1_289_1 [gene=ecpB] [protein=ECP production pilus chaperone] [protein_id=NP_414826.1] [location=complement(309358..310026)]
MKKHLLPLALLFSGISPAQALDVGDISSFMNSD-KQHAEQNDQKQYRQWSPYQYPSRTALFTA*RRAGYLNGQAG*VATHSRQLAATRPSQRS
DPLLL*GTRR*KRALLPHCLV*SGPQ*CAAR*CQPQRCGHCFRPHRHHSGRRPSSGELPLSVRQRLPDKYRKCDAADPRLRTLPESRQR*GV*
RELLPDAGQVASFYPRGHCG*QRTGCTLAG**VHSREI
>NC_000913.3_cds_NP_414827.1_290_1 [gene=ecpA] [protein=ECP pilin] [protein_id=NP_414827.1] [location=complement(310084..310671)]
MKKKVLAIALVTVFTGMGVAQAADVTAQAVATW-IGNSQKRHHQ*AGCDATR*PGVPVCRRH*RF*LTERSI*RGYRG*LNGYRL*TDLTSYH
QHINPVGYLRFHTECGRGL*RRGSRKNWRYRDDRYRQRRTGRQP*PAG*RLQCQQSYHRTGWFHLLHHQRYHQWYHRSNRLQHSTGRHLERRR
*RTVRRDLDQL
>NC_000913.3_cds_NP_414828.1_291_1 [gene=ecpR] [protein=putative transcriptional regulator for the ecp operon] [protein_id=NP_414828.1] [location=complement(310746..311336)]
VTWQSDYSRDYEVKNHMECQNRSDKYIWSPHDA-ILL*RTI*TDCGYRQINLSIVGEN*KRFRVYQSQYGFFI*IYKP***MVIRGKGETGRI
DCGQKVRGLSKLLVLQ*QY*GRGIRWTES*Y*KRTGLCD*WQVPEKRY*ER*NHGPGNGNYPHDGPGNAT*IDCQN*KL*CEDSVYPSA*C*G
QAVLKNI*VGSV
>NC_000913.3_cds_NP_414829.1_292_1 [gene=ykgL] [protein=uncharacterized protein] [protein_id=NP_414829.1] [location=312112..312339]
MQAAPQREETEWRVQSKRGLMPAYRGEAGQQVN-N*DNGIFGEKRQAVGLK*TGRIYPQEN*CWCYKHTNINPI*LL
>NC_000913.3_cds_YP_588437.1_293_1 [gene=ykgO] [protein=RpmJ-like protein] [protein_id=YP_588437.1] [location=complement(312374..312514)]
MKVLNSLRTAKERHPDCQIVKRKGRLYVICKSN-TTF*GRSGS*EKTL
>NC_000913.3_cds_NP_414830.1_294_1 [gene=ykgM] [protein=50S ribosomal protein L31 type B; alternative zinc-limitation L31 protein] [protein_id=NP_414830.1] [location=complement(312514..312777)]
MKPNIHPEYRTVVFHDTSVDEYFKIGSTIKTDR-RD*AGWRNVSIRDN*CLF*IAPVLYREAENSGIRRKCCTIHPTFWSFC*HEKGGV
>NC_000913.3_cds_YP_002791238.1_295_1 [gene=ykgR] [protein=uncharacterized protein] [protein_id=YP_002791238.1] [location=complement(313141..313242)]
MKENKVQQISHKLINIVVFVAIVEYAYLFLHFY-I
>NC_000913.3_cds_NP_414832.2_298_1 [gene=insE1] [protein=IS3 transposase A] [protein_id=NP_414832.2] [location=315291..315590]
MTKTVSTSKKPRKQHSPEFRSEALKLAERIGVT-SRSP*TQPV*ITTLQLAQ*TAKSADVF*T*TGDVYRDCTSQTPAGRTG*RAGYPPKGRD
ILREAPEM
>NC_000913.3_cds_NP_414833.1_299_1 [gene=insF1] [protein=IS3 transposase B] [protein_id=NP_414833.1] [location=315587..316453]
MKYVFIEKHQAEFSIKAMCRVLRVARSGWYTWC-TAADKDKHASAVPPTLRQRCPRGFYPVKTALRCPTPDG*TACSGLPL*RKNRGGKPAPS
GTEGKGLPEVQPGQLPRTRPACVRKSVGAGFLRQWPEPEVGRRHHVLTYR*RLAVSGSGH*PVVTCRYWLVNVATHDGATGLRCPADGAVAA*
EAPERYRSHGPWRPVLFSRLSGATEAA*SAWKYERKRLLLR*CLRGKLLSFAESGMYPWRTLYQPGNNAGNGV*LYRM*LQSVAAAQLVWRPQ
SGTI*KQEPRL
>NC_000913.3_cds_NP_414835.2_302_1 [gene=rclC] [protein=reactive chlorine species (RCS) stress resistance inner membrane protein] [protein_id=NP_414835.2] [location=complement(317726..318319)]
MEKYLHLLSRGDKIGLTLIRLSIAIVFMWIGLL-KVCPLRGRQHYTIRRKQSTNVVLL*TPGRL*TVSDSRRRIQTRSKGMANGQ*YLWFFQR
SWRRGGDYCSAGFG*SCQSLVRFIGRTDGIYHAAGNTLIFNHHPGGMGTRIG*RSSWFPLFIRCWSPGIERYSDAGRCSHDNGRFGAGNS*TT
Q**IQFNVKN*IL

>NC_000913.3_cds_NP_414837.2_303_1 [gene=rclB] [protein=reactive chlorine species (RCS) stress resistance periplasmic protein] [protein_id=NP_414837.2] [location=complement(318331..318567)]
MFKKSVLFATLLSGVMAFSTNADDKIILKHISV-IVSISITDSSGGYHC*YSQKI*CFILESHIDAN***FNRNSSIV*I
>NC_000913.3_cds_NP_414838.2_304_1 [gene=rclA] [protein=reactive chlorine stress species (RCS) resistance protein; pyridine nucleotide-dependent disulfide oxidoreductase family] [protein_id=NP_414838.2] [location=complement(318676..320001)]
MNKYQAVIIGFGKAGKTLAVTLAKAGWRVALIE-TIKCNVWRDLY*YRLHPNQNIGS*RTAAHRFCPCHTA*K*SG*FFT**EFS*SCGYAQY
RRDRRPGGVYQ*S*PACSSA*GKSGNSWRENFY*YRCTNRGSANSWNYHHARSI*QHRIT*SKRIAWAFRYFGRRIYWR*VRLYVR*FWQQGN
HFRSSFAVFASGRSGYC**YRDDFTRSGRRYYPQCPCGANQSP*KSSASA*RARPTGGGCTVNSFRSSTGYRFVTSRKCRYRSKRARGNCR*Q
AITYHRRQYLGDGRCYRRAAIYLHITG*LPHCT**VTG*RQT*Y**SEKCALFRIYDTAPVQGWYDRRTSQREWC*YSGGDIACSCNSACQSD
E*YSWGIKSDC***NPTYVRGITAVC*LPRDDQYSENGDGCRAAL*HITRSDIYSSVDERITQ*SIFISQI
>NC_000913.3_cds_NP_414839.1_305_1 [gene=rclR] [protein=reactive chlorine species (RCS)-specific activator of the rcl genes] [protein_id=NP_414839.1] [location=320227..321081]
MDALSRLLMLNAPQGTIDKNCVLGSDWQLPHGA-RGIIGYSLACVNARSGEAGNADGGDFYITPGKCGPATTKFSSSPESCR**IDLYCLRHS
SVATFGALFFNVFAGNAVFSTG*PQRGI*LAEGGDPVFTTGIQIGNAGSGCTV*PDLRYILYPRGA*VDCTG*Y*EKHSQFASASTSWCGNTA
NAGNARTRLDRRIAGQHRSHVPGKFCPAFP*CFRNHAAGCINKVASTNSGPDVFPGNAPCCGDR*VSRLCQ*IIFSQGVCPRVWLYPGRISGK
GQTACTL
>NC_000913.3_cds_NP_414840.1_306_1 [gene=ykgE] [protein=cysteine-rich LutA family protein; putative electron transport chain YkgEFG component] [protein_id=NP_414840.1] [location=321608..322327]
VNVNFFVTCIGDALKSRMARDSVLLLEKLGCRV-KFPGETGMLRSACDQ*RLYQRSDSRDEKSDRRTGG*RRSHYFTGWLLHLCRKKLPDVSG
G*T*MGITCRKGCRAYAGSHLFYC**IRGSRCRCQFARESGVSPIL*PGP*AGSEGRATYAAEKCAWTGAVDLC*TGYLLRIWRHVLGQNGRN
IRRDGERKGCAPDGSPP*VFNWC*RELPAKHQWAITTGRAESQSDAYC*SVDEPL
>NC_000913.3_cds_NP_414841.1_307_1 [gene=ykgF] [protein=ferridoxin-like LutB family protein; putative electron transport chain YkgEFG component] [protein_id=NP_414841.1] [location=322338..323765]
MSIKTSNTDFKTRIRQQIEDPIMRKAVANAQQR-NWGKSAKNGR*IGALGGVARSGRPDT*SCSE*SRRLSVPALRKSDAKRRSRLFCKNQRR
RYPLHFTGCPTQKCPEGGEI*IDGDRRDWCQSCVAGCWHSGD*NRSG*IYSPAGSRSAISCCGPGNS*RSPSDPSSATRTSGL*GAGNA*SDD
LIHPAKNPRRFPQC*NRYYRL*FRGGRDRFGMPGDQ*R*CANVYHAA*NAYCSDGNGAYCPHVCRGRCIDHHAGAQCRWCTFDGIQHLADRTA
RSWAR*WS*RVSSGYCR*RAF*GAGL*ISGCAALYSLRGLYEYLSGISPYWRSWIWLYLSRANWCGDFSATWRL*RF*RFTLRLLFMHSL*QR
VSGAYSAVKTDFASSSGDG*KRDHRKSRATGDKNVRLCQ*SSRIVESRDDGRCSCGKLVYQWRQNTTQIWRD*RLDGSTRSS*S*RREFP*LV
*ETSGAGEKEWI
>NC_000913.3_cds_NP_414842.4_308_1 [gene=ykgG] [protein=LutC family protein; putative electron transport chain YkgEFG component] [protein_id=NP_414842.4] [location=323758..324453]
MDNRGEFLNNVAQALGRPLRLEPQAEDAPLNNY-S*RAAYPT*PTAAL*RVYSVCQRCYVDAL*ADQRGEGGRSCNTSV*RAGRSVGRD*R*H
EAGGIGD*RTFAAGMQCRCLGSGERCREYLAGRAG*SGCCVC*IWFNRIGRRGSFFRRRARAFIEPAPGIFSFYPA*KHYPAACSATRRKIAS
ESAGR*TNAFLH*HH*RPQFNGGY*AYQSRRSSWPGESGVSDY*GLL
>NC_000913.3_cds_NP_414844.1_309_1 [gene=ykgH] [protein=putative inner membrane protein] [protein_id=NP_414844.1] [location=complement(324696..325364)]
MREQIKQDIDLIEILFYLKKKIRVILFIMAICM-SYGAVVSVYQ*RQYKSDLQPKNKPDNARYTC*L**Q**FCLSDYND*RCYSANYYIFSH
QPRCQEQRNKAGMVRR*ESFTNC*RGNISRAGLYYQMVCVRIS*WQASSR*DTNAFSN*Q*ALYKNDIPDQELVIVSER*WLCNYKLTRNKK*
IPCCHLPGSGIFSKHCNFCNVLPCQKNGR*IPTKLWAV
>NC_000913.3_cds_NP_414845.1_310_1 [gene=betA] [protein=choline dehydrogenase, a flavoprotein] [protein_id=NP_414845.1] [location=complement(325577..327247)]
LQFDYIIIGAGSAGNVLATRLTEDPNTSVLLLE-SGRPGLSL*LPHPDARCPGIPATG*TLQLGL*NGT*TVYE*PPHGVRTR*RSGWIVADQ
RHVLHPWQCAGSR*LGARTRSGELELPRLPALLPQGRDSRYG*KRLSRR*WPGERHYLQTRRQSAV*SDD*SGRAGGLPAHGRSQRLSAGRFW
SDGSHRHAAGPSRQHRAWLSRSGQIAS*PDHSYSRYDRSHHF*RQTRGGRRMAGRRQHHPNPRNGQQRSAVMCRRDCLTADPATLRRRQR*TA
GGV*YSAGA*ITRRRRKSSGSSGDVSAI*VQRTGFPLPCPAVVEPAENRCGVAVWRHWRWCQQPL*SRWIYSQP*GICVAEYSVPFPASSD*L
*RLECSERARFPVPRRLNALAKPWACAD*IPRPAPASGDSV*LHVARAGLAGVPRRNSHHPRDHASTRAGSVSWPRNQPRCRMPDG*TAR*VR
A*PRRNRLPSVRYLQNGLRRDVRG*RRRPRTRVRRPACGGCVDYAADYHREFERHDNYDWRENSGYDSWTGSAAEEHGGIFCGKWDAGESEKM
>NC_000913.3_cds_NP_414846.1_311_1 [gene=betB] [protein=betaine aldehyde dehydrogenase, NAD-dependent] [protein_id=NP_414846.1] [location=complement(327261..328733)]
MSRMAEQQLYIHGGYTSATSGRTFETINPANGN-SAGDRAGRRARGCRSRREKRPAGAKNLGVDDRHGALAYSASGR*YSA*TQ*RTRKTGNP
RHRKSIFGNLNRRYRYRCGRAGVLRRADPGAGRQPDPVA*NVLCVYPPRTAGRSGRDWRMELPDPDCPVEIRPGAGGRQRNDFQTERSYPAYR
VKAG*NLQRSGPAGRRI*RVAGRGRGDRAISDRASGHCQSVIYRRCRQRQKSDG*LGGLFPERSDHGTGR*ITADRFR*CGSRSRRRYRHDGK
LLQLRSGVYQWHPRLRSGEMQSRI*AENSGAR*AHSRGRRFRSAN*LRPAGQLPASR*RAALYRQRQRGRRARTVRRRCTERRWLR*RRMGCT
DSVHRLQRRYDHRA*RDLRASDVHSDLRVGRRSHSPR*RYRLRPGGGHRDSGPEPRASRHSSAGSGYLLDQHLGRIPGRDARWRLQTLRHWSR
ERRDDAPELHPGEVHPG*DG*IPVHIL
>NC_000913.3_cds_NP_414847.3_312_1 [gene=betI] [protein=choline-inducible betIBA-betT divergent operon transcriptional repressor] [protein_id=NP_414847.3] [location=complement(328747..329334)]
MPKLGMQSIRRRQLIDATLEAINEVGMHDATIA-TDRPPCRRFYGDHQPLFQGQKWSAGSNHARYHQSAA*RGFESITCTSAGQCRAAITGDC
WRKLR*NAGEQCGDESLAGVLGQQYASADALSFTAGQQPPLAVESGERVSSRIAARTGTGSGLRPGRAD*WIMAARGSERQTAG*NPR*FPDP
PLYHSASTHRL
>NC_000913.3_cds_NP_414848.1_313_1 [gene=betT] [protein=choline transporter of high affinity] [protein_id=NP_414848.1] [location=329463..331496]

MTDLSHSREKDKINPVVFYTSAGLILLFSLTTI-TVSRLLGPVDWPHAGLGF*NLRLVLSAGGNALYCLCGLYRLFAFWFGEARARTIQTGIQ
PAELGGDAVCCRDRYRPDVLLRSRTGNAVYAAAGRRGTDD*GRASGDGLDAVSLRLNRLVDVCADGHGARIL*LSL*FAAHHPLGAVPDLR*T
D*RADRSLSGYCSGDRHYLRYCHYARYRCGAA*LWLERTV*YSRFDGGESGTDRLVGDNRHDLCHLRCR*GHSRVIGA*CRAGAGIDPVRIVY
GRHFVPA*CTGAECWRLCESLYGHDAQQFCLRPSG*VDE*LDALLLGMVGGMVAVCRLVPGAYLAWAYHSPVRAGHVDYSVYLHAVMALGVRQ
*RAV*NHPRRRGICRGSDGPSGARLLQPAGAVSGVYL*RLRRHHYWPAVLCDLGGLRGAGAGEFHLAA*RYQQRRPRLAARLLVGGDWPADAR
HADD*RDIRAAKHHGDYGAAVQLCDLLRDGGVV*ISEGRRLPP*KCQPRYRTATAGASGSPELEKTSLAPDELSGHALH*TDDGDGLLPGNGR
SGAGVAVARRVRGAKKPATGRGTAVGSSGFVGAYGRRAKLCLSDLAAAIFGAGLYLPRTQR*IDLLPAGNLPVRRQPGQRPDGLQQRAGDHRY
S*PVRAAP*LYSSPS*SAGP*RDVPGRV
>NC_000913.3_cds_NP_414849.1_314_1 [gene=yahA] [protein=c-di-GMP-specific phosphodiesterase]
[protein_id=NP_414849.1] [location=332371..333459]
MNSCDFRVFLQEFGTTVHLSLPGSVSEKERLLL-KAADAGNVCNRNITVQKSQCKDNFTSKETAL*ETGDSERYYFLARYFLSVQSGDHIRHG
E**SQIY***SLSPYRHA*SHQSGVGKPRIQTVDPTGFLRADWRTDGL*GACPLGTSTNGNYPTGSVYSSGGVIRSYCHNDPPTDETDCGYSD
AGKTFAAGQFPYWHQRLGGLFFGSGI*KRVSEPG**IR*R*NQAGSRANGT*PYSGNARSQSDI*QPSSAQHYLCAG*LWYGLCDLSLLAGVP
GRFY*DR*VICANGECRRNLRSYCGQYCRTSA*AWSEYRGGRGRNPGAGGFNDR*RRSLFAGLFVLSASTG**IYL*MGNESRWL
>NC_000913.3_cds_NP_414850.1_315_1 [gene=yahB] [protein=putative DNA-bindng transcriptional
regulator] [protein_id=NP_414850.1] [location=complement(333501..334433)]
MNSIFTEENLLAFTTAARFGSFSKAAEELGLTT-IRH*LHH*AYGDGAGCGAVHSQYPQH*VNGVRALFFPQSYRPAE*FLCHQTQNRYYFAG
HRSACAHLY*SAALYAQTHRTLVTGAEKAVSYLPDYRHHRSV*RRLGCDY**SGQYRHRRTRYTTGRRRY*LH*NWRDSLGFCYRPGSPVSLC
AGTHRRKPTASLP*YYGGGHRAYD**KSGLVAARAGVNSGARFQHQMSVPDFR*RNWFFAGLHGP*GDDAIPAGYPTNP*SAPGFAHVTGDSA
FCYGSGDAVD*KTICAKWHINGDLSGFTASGEL
>NC_000913.3_cds_NP_414851.1_316_1 [gene=yahC] [protein=putative inner membrane protein]
[protein_id=NP_414851.1] [location=complement(334525..335022)]
MNGLTATGVTVGICAGLWQLVSSHVGLSQGWEL-IRNHWLRRLLQFLCRRRR*IGVYQKSGG*LFRDGVGVFRRADCWLAGLCKWTVRVLGER
NYYRTLLGGSGLARAFLVTVLYSRWLSRHDAVFCQRDELDGDVTGFSGG*LRGGNFRIWWTKIKRSDD*TRRVL
>NC_000913.3_cds_NP_414852.1_317_1 [gene=yahD] [protein=ankyrin repeat protein]
[protein_id=NP_414852.1] [location=335280..335885]
MSIKNLPADYLLAAQQGDIDKVKTCLALGVDIN-NLRSSGENGNYAGKFISAICLRSGIN*CRSGY**ARSYLFKSFFN*LSER*FNATTNYF
TG*TRS*LRNPFWRCRPDACL*KRPFKYCKRAFGAYGD*R*PDQPCRLDAATGSDCA**WWY*TAGDCAVIAGTRCQPASDR*IWQNATGTGA
GTGL*RDCAVTDCRRCI
>NC_000913.3_cds_NP_414853.1_318_1 [gene=yahE] [protein=DUF2877 family protein]
[protein_id=NP_414853.1] [location=335925..336788]
VWALTADADFLAQRGQGQVEQVFARAVNIALPA-TPAVADAAL*RVRQCAKQLSVGTHSL**SVPAW**GSV*RSRYYGWSTSSYRDESLSAL
AVPNLANDRCEFSPYRLATVARHYSSAPGGK*NPV*LSRR*SVLSGVK*RITY*TTGSYSGRKR*TKYRLSGRQYDGVRDWPYAISRRLFNRS
GAYFIYSRASGGKIQRGILSRSATRQK*YHIIKCHNAGSRITTTLPGKYSSFYSQHYL*HPWERNSGNRKN*TYWLQFRLRHAVWHGRWLCAE
PNLRRELCQL
>NC_000913.3_cds_NP_414854.1_319_1 [gene=yahF] [protein=putative NAD(P)-binding succinyl-CoA
synthase] [protein_id=NP_414854.1] [location=336778..338325]
MSVKIVIKPNTYFDSVSLMSISTRANKLDGVEQ-SICGDGDRNE*RRAEEFRTADAGAGAGEKRRPDDCHQW*IGCGQRAVTGGD*RTVQHQS
AKRLARGALRHYWQRQKAYPGK*PGGDFGQRSVCRSRSASGAAKRSQRDAVFR*RLS*R*TGAQATGPRKRAADDGARLWHGDYQRRGALFW*
RRASRQHRYCWRIRHRQSGVERPHS*IWRRRFATDWHRRARPERENRRPDDARRHRDAGKRSAN*NHCAYLQTACACGGPQSAGTCARLPQAG
GRLLPRSWRNASG*AGATVCPRHQRGSAKSGDALRRETGKSRPAYA*PAVDCGCACASATAAEIHSWPVLRRHAVRRNHVRGDGKTWRCLQQH
SARSGIPPERYQPQHQTHLPRLWR*RLHQWQAAPDD*PHQPHQSLDRRGARSRSGGDRDGFCARIWIA*RSGRLHHRDDQRSESDRRCRRTRV
DHSRLCAGYRS*YAIVRTTKPDAA*CRSDSGEQQHQYRIAGA*IYLQRGGSL
>NC_000913.3_cds_NP_414855.1_320_1 [gene=yahG] [protein=DUF1116 family protein]
[protein_id=NP_414855.1] [location=338325..339743]
MSQSLFSQPLNVINVGIAMFSDDLKKQHVEVTQ-TRLDAAGAGQYAGGAGAG*HCRFAAGGQNRRR*PAGAGAYYPVASGADWF*SGD*RGAG
HDGENHSSRRAADHLGKNVRRDERRGHRSAGVRRTGERSRRGG*TGGFRGDHLLAVSRARLRGIDGGCYLGLDVYAHRKKQNLRQHRLYQHER
ADGEDFAYGR*RPERD*PPELDA*CAGTNTARRDENYRRNRSALNAGAGAAYGR*VP*PQ*RRDDTADSGADAGDYSGGLFRRATARSV*VCR
QQRLLLRPDVDGDV*SGDGCGAWHRIQHRGHHHGA*RRRVRPAGQRPAGAMVYRPGAAGDRPDVCRL*AGRFGAGYRRQRHHRNLRYWRICYG
DRARYRRAGGRHGGRSY*FLPSDARNHPR*KPQRHHSAARFYGRAVGNRHHPRG*QRHSAGDQHRHRP*RCGRRHDWRGHCASTFCLLRESHS
WLVRTLRRL
>NC_000913.3_cds_NP_414857.1_321_1 [gene=yahI] [protein=carbamate kinase-like protein]
[protein_id=NP_414857.1] [location=340165..341115]
MKELVVVAIGGNSIIKDNASQSIEHQAEAVKAV-SRYGAGNAGFRLRHCADPRQRAAGRAGFTPCGDCPQARRAALNAAGELCGRYAGRHRLS
DPTGAE*PAGASRREESRHRGDSGGSG*KRSRFCPSHQAHRRIL**QPA*RITKGKP*LVFC*RCRAGLSPRGRLAGTETYCRSTCH*SADPT
RFCRNWRGRRWNSGSAY*RGRLPKRGRGYRQRSLYRAAGP*NSRRHSCDHHWRRKSVYSLWQTAAAGARSGGYCHHDPLYAGRAFPARQHVAK
NHRQPDIFRTRRQRSDYHHAGMPACGAARRNGHSYY*NV
>NC_000913.3_cds_NP_414858.1_322_1 [gene=yahJ] [protein=putative metallo-dependent hydrolase
domain deaminase] [protein_id=NP_414858.1] [location=341125..342507]
MKESNSRREFLSQSGKMVTAAALFGTSVPLAHA-SGSWHPKLRSEQHHENH*PALLSR*RAAGNRF*LRKWRGGADPHGAPDRGDSGRQNCRP
AREQAASGRHAAAL*RWR*ADAAHHPRHAYSSRQNLLRRAVALAQSSGRHHHPGHDQTRAENAAGTATVHSGAGRKTD*FIAVERHHHCPQPL
QYRTGFRPEKSAKFAGGAGATSGGL*V*NRRLPAARFAAVEI*TFNA*SDAGGGALRRRPGPDQC*WRDGKIPRHHVPDCAGLRQRRRYSPAR
NHSGRRGSHQLYG*NGRENATAEGQADHQSRLCAGNAQRATGR*TGEPDGGATNFYRLDGADWHAAYAAQTVARQRRKSDDWH*QRYRPLVAL
WSGRHAGKSQSVRAALYSS*RTEPLPLAVFSHWRCIAAE*KRRACMAKSAG*RQLCAGGRLLFRRGGGAYLAENRNVP*RATGVGECGRL
>NC_000913.3_cds_NP_414859.1_323_1 [gene=yahK] [protein=broad specificity NADPH-dependent
aldehyde reductase, Zn-containing] [protein_id=NP_414859.1] [location=342884..343933]
MKIKAVGAYSAKQPLEPMDITRREPGPNDVKIE-NRLLWRLPFRSPPGPFRVGGDGLPLRAGS*NCGACGSRW*SGRKICAGRSGRCRLHCRQ
L*TLRRV*RRVGKLL*SHDRHL*LADAGRTGPYSGRLLTTDRRS*AICSAYSSPARAAGGGGSFVVCRDHHVFAATSLAGRAG*KSGRGRHRR

SGTYGD*AGPRDGGTCGGIYHF*GKTRSGKSPGGR*SC*LTQCR*DGGSSEEFRFHFEYSSCAT*SRRFYHLAEA*WHHDAGWCACDTA*IAG
SFQPDHETPCDSRFYDWRHSRNSGDARFLRRTWHRG*YRDDSGRSN**SL*ANAAR*CEISFCYR*SHTNRL
>NC_000913.3_cds_NP_414860.1_324_1 [gene=yahL] [protein=uncharacterized protein]
[protein_id=NP_414860.1] [location=344176..344991]
MISLKAPHNNLMPYTQQSILNTVKNNQLPEDIK-KLPGFLCRYIQGFD*TIL*LSL*LS**FSR*RQTHSSHGCRTRL*VVRR*RTHHKCAV*
*FPWIL*LDGLP*SPG*VCFSHT*KSKRDSLGI*PG*CVS*Y*SKRAGWTLYRA*EISAVYRFSANRLQMYSS*IPYAFAENFP*RERVYACG
KMASRNVVYSGDRRAH*AGACAWIIITLKKYLPCFS*ITGDVIALCSGRKNIQR*CAIK*MDI**SAGIR*QILSCK*AR*NPLHSL
>NC_000913.3_cds_NP_414861.2_325_1 [gene=yahM] [protein=uncharacterized protein]
[protein_id=NP_414861.2] [location=345404..345649]
MAVQLFKTLLNQIPLLSSLQSGTLPLFGYSGWG-KTNEKSPHRGEWAEMGS*RLK*TYRK*GPRITGAIIADGRDSANQVDYV
>NC_000913.3_cds_NP_414862.1_326_1 [gene=yahN] [protein=amino acid exporter for proline, lysine, glutamate, homoserine] [protein_id=NP_414862.1] [location=complement(345666..346337)]
MMQLVHLFMDEITMDPLHAVYLTVGLFVITFFN-TGSQSLCGSTNQPGFRSTRRGADRAGRGAGRCILFRVGFVWSCNANYAV*GDFFAYQNR
RRRLSLMVCVVQHAPPVNTANEHTTTTD*RPLVCLFSPRINYRSL*PANRFIFYQYFLSNIKCRNTNMGTFNGLGGDCARINYLASFS*SGVF
FARCASCLWAYATRCQSGYWCNYWCIRATPDLRRGDAAV
>NC_000913.3_cds_NP_414863.1_327_1 [gene=yahO] [protein=periplasmic protein, function unknown, YhcN family] [protein_id=NP_414863.1] [location=346484..346759]
MKIISKMLVGALALAVTNVYAAELMTKAEFEKV-RIAV*KNR*YFNQQ*NVDCRCKRRFDQKSG*KRG*CVGTDLRSN*Q*DPRHGKYL*EEV
>NC_000913.3_cds_NP_414864.1_328_1 [gene=prpR] [protein=propionate catabolism operon regulatory protein] [protein_id=NP_414864.1] [location=complement(346857..348443)]
MAHPPRLNDDKPVIWTVSVTRLFELFRDISLEF-RSPGEHYPYSAWL*KSSDLHPQETGKRTL*RHHRGWL*RRVPEKPPVSASYFD*TERLR
CVTGTGKSRKTHLFYRRCHLSGNHSSAGGVSKNL*FAPRPT*LHYRGRRTRAD*RAKS*RHRSGGRRRADYRFGRRSRNDRNFYLFRRHRAPG
VQRCAGYDAHVVTP*HSRCHPQRPAYSLRAGRYARSITTDGTSTADYFAVCPLQCGGVD*GGNGDGQRAGGPGDSSGIFCPPRCATGQKVASV
CCRQLRGDCRIAAGSRTVWL*GRGVYRLATRRSRRAVRNCPRRYAVSG*DWRNAATFADPAAAGAGRKRGHPRRRASACSGRCTGH*RHSLQS
GRRYAARTFSP*SVLSAEYFASAIATTARAGGGYSSAGGKLFESVSGGALRPIFCCITPGVTGK*NCAAALRLARQYS*TAQYDGTTGAVFKC
GTDAGFNAAVYATATAGTGARVGENSRSTLTDTTTGTGEI*WR*NSSGELFRHQPDDVLAAAEKL
>NC_000913.3_cds_NP_414865.1_329_1 [gene=prpB] [protein=2-methylisocitrate lyase]
[protein_id=NP_414865.1] [location=348682..349572]
MSLHSPGKAFRAALTKENPLQIVGTINANHALL-SAACRISGNLSFWRWRGGRFAGAARSRYFYP**CADRHSPYHRRLFAAAAGGCGYRFWF
FGL*RGAHREIDD*SRCGRIAY*RSGWCETLRSSSE*SDRLERRDGGSDPRGGGCENRS*FCDHGAHRCSGGRGAGCGDRACAGLC*SGCRDV
VPGGDYRTRHVPPVCRCGAGADPRQHHRIWCHAAVYHRRITQRPCRNGAVPTFSVPRHEPRR*TCLQRPAPGRHAEKRHRHHADPQRAVRKHQ
LLPVRREARQPVCP*PGEI
>NC_000913.3_cds_NP_414867.1_330_1 [gene=prpC] [protein=2-methylcitrate synthase]
[protein_id=NP_414867.1] [location=350012..351181]
MSDTTILQNSTHVIKPKKSVALSGVPAGNTALC-NRG*KWQ*PALPRLRYS*SGETLRI*RSGASADPRQTADP*RTRRLQNETESPARFTG*
RAYRAGSLTGGVAPDGCYAHRCFRARLHAARKRGAYRLWRAGYCRQTAGVA*LDSPLLVSLQPQRRTHPTGNR*RLHRRSLPASAARRKAIAK
LGKGDAYLAGAVRRTRV*RLHLYQSGDCGHRL*YVFRDYWRDWRTARAKTRRGE*SVAGDPATLRNAGRSRSRYPQARGKQRSGHWFWSSGLH
HR*PAPPGD*TCGEAALAGRRLAEDVQHRRSPGNGDVGEQKDVPQSRLVLCCFLQHDGRSHRDVHTTVCYRPRHRLGGAHYRTTSGQQNYPSF
RQLCWTGRPPVCRAG*APV
>NC_000913.3_cds_NP_414868.1_331_1 [gene=prpD] [protein=2-methylcitrate dehydratase]
[protein_id=NP_414868.1] [location=351215..352666]
MSAQINNIRPEFDREIVDIVDYVMNYEISSKVA-IRHRTLLPARHARLRSGSSRIPGL*KTAGANCSRHRRTQRRARPRNSVPARPRPGGI*H
RRDDPLARFQRYLAGGGVGPSFRQPRRHSGNGGLAFAQRGRQRQSAVDHETGADRNDQSP*NSGLHRAGKLL*PRRPRPRSVSESGFHRRGRR
NARPDPRGNSQRRFAGVGGRSVAAHLSPCAEHRHA*ILGGGRCHFPRGTSGTDGENGRNGLPVSPDCAGVGLLRRLL*R*IVPLPAPVRFLRY
GKCAVQNLLPGGVPLPDGS*SSDDAL*TDAGSRQNGGGYRKSDHSHPRSLYSHHRQKRAAQ*PGRPRSLHSVHGGDPAAIRALNGGRLRGQRC
AR*TH*RPAREDQLL*RSGIYR*LPRPGKTRHRQCHYP*VHRRHTI*RSGGGVPHWSCSPPSGWYSETGR*IQNQSRAPVPDSPTAAHSGGFS
RQSSPGTDAGQ*VSRPVRHL
>NC_000913.3_cds_NP_414869.1_332_1 [gene=prpE] [protein=propionate--CoA ligase]
[protein_id=NP_414869.1] [location=352706..354592]
MSFSEFYQRSINEPEQFWAEQARRIDWQTPFTQ-NARSQQSAVCPLVL*RPNQLVPQRHRPLAGETARGAGADCRLFGNRRRAHLYLSSAA*R
SERGGLNVAFIGCAARRSGAGVYADDCRSAYYSAGLRAHWRYSLGGVWWICLAQRGGAN**R*TGADCLG*CRSARWQNHSL*KIARRCDKSG
AAPATPCFAGGSRAGENGARQRAGCRFRVVAPSTHRRAGTGGVAGIQRNLLHSLHFRHDRQT*RRAA*RRRICGGAGDLDGHHFWRQSGQRVL
LRIGYRLGGGAFVYRLRAAAGGDGDYRLRRIADLAGLRRVVDNRREISG*PDVLSADRHSRAEKIPYR*NSQTRSLVAGSALSGWRTAGRADR
QLGEQYAGCAGHRQLLADRIRLADYGDCSRSGRQADASGKPRCADVWL*RAVA**SHRRTVWRQRERDAGGGRAAAAGVYSDHLGRRRPLCED
LLVAVFPPGVRHL*LGHP*R*RLSLYSRAH*RCN*RCRASAGDARD*REYLQPSGRCRSGGGWGERCAERAGGGGVCHSERERQSGRS*CGAL
AREGDYGAGGQPDWQLWPPGARLVCLAIAKNAIRKNAAPHDPGDLRRTRSWRSDDH**SCVVGSDPPGDGRV
>NC_000913.3_cds_NP_414870.1_333_1 [gene=codB] [protein=cytosine transporter]
[protein_id=NP_414870.1] [location=354922..356181]
VSQDNNFSQGPVPQSARKGVLALTFVMLGLTFF-IRQYVDRRHSRNRS*LS*FLPRSSHR*SSPRYLHFISRLHWRKNRPDHSSSCSLLVWC*
RLMAAFTATGRNSGWLVWRRCGDVCHSGG*GNRAGY*FADCRFRFTDDRHRLFWHFGADGSFGDCGSGYRLPGRLFRVAGC*RHGRPGRIKSG
RSRTTVRFQCRAGAGCGVIYQCGYAHR*LCPVWSQCQTGGAGGDGGLFPRQLVDVYFRCSGRCGTGHGGYL*CDDCSGPAAACDCGAGAEYLD
HQR*RTLCVGFRFRQHYRDVEQNPFGNQRYYRYGLRIMAV*QFCRLVDLPFGSYSSSGWRDHRRLSDEPSPL*ALCDHAYDECQLGGDSGGRL
GDCCRPLVTGNCSGQRGIRWRAELSDP*PDFES*NDSSNDACGG*QCRI
>NC_000913.3_cds_NP_414871.1_334_1 [gene=codA] [protein=cytosine/isoguanine deaminase]
[protein_id=NP_414871.1] [location=356171..357454]
VSNNALQTIINARLPGEEGLWQIHLQDGKISAI-RCAIRRDAHN*KQPGCRTRFSYTAVCGATYSPGHHANRRTTELESVRHAV*RH*TLGRA
QSVINP*RCETTRMANAEMADCQRHSACAYPCRCFGCNANCAESNAGSEAGSRAVD*SANRRLPSGRDFVVSQR*SVAGRGVTLRGRCSGGDS
AF*IYP*IRRGVAA*NLRPGAKIRPSHRRSL**DR*RAVALCRNRCCPGAP*RHGRASHRQPHHGNALL*RGVYLTPVPLAENVRY*LCRQPA

GQYSSARTFRYVSKTSRHHAR*RDAGVRH*RLLWSR*CLRSVVSAGNGEYAASAAYGAACLPVDGLRAD*RWPEFNHPPQRKDVEFAGLRHCR
RKQRQPDYPAG*KWV*CAAPSGSGTLFGTWRQGDCQHTTGTNHRISGAARSHRLQTL
>NC_000913.3_cds_NP_414872.3_335_1 [gene=cynR] [protein=transcriptional activator of cyn operon; autorepressor] [protein_id=NP_414872.3] [location=complement(357791..358690)]
MLSRHINYFLAVAEHGSFTRAASALHVSQPALS-TADSPVRGEFRRAAV*P*RANDSSH*CRRSLATVRQPGVTGTGGG*TGDS*CCRSDARI
AAYRRHPHLYELLYRPLNGGFLCALSQHHAPATGNVAGENRGYALPRRVGRWDCLRACAFAGAGGNSFTDRKFSVSRGATSSAGRP*TGGVES
LA**KTGPAQRGICHQRAN*PLLRESGATSTGGH*GELN*RGSGADSPHFPFHIVTSSDCHTT*RA*SYFSCPATTGENGGFVAAEK*LADSR
REGIFAHGVG*MRGCWRK*ITV
>NC_000913.3_cds_NP_414873.1_336_1 [gene=cynT] [protein=carbonic anhydrase] [protein_id=NP_414873.1] [location=358799..359458]
VKEIIDGFLKFQREAFPKREALFKQLATQQSPR-NTFYLLLRQPSGP*AGDAT*AWRSVRYSQRGQYRPFLRAGTRWRFCFGGVCRRCASGI*
HCDLWSFQLWRDDRHCQLSVHGPYACRLPLAALCRFSPRR**GAPAFRFTVKSCGDGT*KRHCSVG*FANSSIGAPGARRGADRPARLGLRH*
KRQHRSF*RRNPPVCATGR*SSRLCHTATPTDRSV
>NC_000913.3_cds_NP_414874.1_337_1 [gene=cynS] [protein=cyanate aminohydrolase] [protein_id=NP_414874.1] [location=359489..359959]
MIQSQINRNIRLDLADAILLSKAKKDLSFAEIA-RRHRSGRSLCNRGFAGSAGASCRRRPPGRGEAGSRRRLHSTVADDSTAWLH**PYSN*P
NDVSFL*NVAGVRYNPESVGS*EIWRWHY*RD*LQTRR*ESGGPGRWRTCGHHLRW*ISADQTVL
>NC_000913.3_cds_NP_414875.1_338_1 [gene=cynX] [protein=putative cyanate transporter] [protein_id=NP_414875.1] [location=359992..361146]
MLLVLVLIGLNMRPLLTSVGPLLPQLRQASGMS-I*RGCPVDRSAGGYHGRAGAGRKLASSACQRTSQCRHQSVADCRRCIDA*ALPAKCAAA
*QRTAWWGGDRHHSGGDAFGD*TAVSAAHATGDGAVVRGSDGRRWAWCRHNALVSST*RNLVSNTRLVGAACRCCALCLVVAKRPRGRLFPQD
NNHSGSRGIHSPRVDAGCLLRSD*RRLRQPDCLVTRFLY*DWCQRAVQRFLTGIDDAWASRRSFADACYGSPSGSAQTVNAGAGVTTGGVLRL
YLAADAIAGIVGDGVWVRSGRRVSALFAAGARSLCATGYCWQAGGVYAGNRFYHRRACPVVFWRAA*YQRQLPDGLGISCAVRRWADDHNPAF
CTSTFSAAVGQRGM
>NC_000913.3_cds_NP_414876.1_339_1 [gene=lacA] [protein=thiogalactoside acetyltransferase] [protein_id=NP_414876.1] [location=complement(361249..361860)]
LNMPMTERIRAGKLFTDMCEGLPEKRLRGKTLM-I*V*SLASIRS*KKRKPD*RNVCHGRGKRLGRTACLFLLRFQHPYRPQFLCKFQFNHCR
*LHGNNR**RTDCTQRYSFRYGTPCTP*IEKKRRDVLFSDNDWQ*RLDRKSCGY*SRRHHRG*FCYWRG*YRHKRHSTKRRGGWRSLSGYSRN
KRPG*ALLFQRL*S*IVSL
>NC_000913.3_cds_NP_414877.1_340_1 [gene=lacY] [protein=lactose permease] [protein_id=NP_414877.1] [location=complement(361926..363179)]
MYYLKNTNFWMFGLFFFFYFFIMGAYFPFFPIW-TT*HQPYQQK*YGYYFCRYFSVLAIIPTAVWSAF*QTRAAQIPAVDYYRHVSDVCAVLY
FYLRATVTIQHFSRIDCWWYLSRLLF*RRCASSRGIY*ESQPSQ*FRIWSRADVWLCWLGAVCLDCRHHVHHQ*SVCFLAGLWLCTHPRRFTL
FRQNGCALFCHGCQCGRCQPFGI*P*AGTGTVQTAKTVVFVTVCYWRFLHLRCF*PTVC*FLYFVLCYR*TGYAGIWLRNDNGRIT*RLDYVL
CATDH*SHRWEKRPAAGWHYYVCTYYWLIVRHLSAGSGYSENAAYV*STVPAGGLL*IYYQPV*SAFFSDDLSGLFLLL*ATGDDFYVCTGGQ
YV*KHRFPGRLSGAGSGGAGLHLNFRVHA*RPRPAFPAASSGE*SRL
>NC_000913.3_cds_NP_414878.1_341_1 [gene=lacZ] [protein=beta-D-galactosidase] [protein_id=NP_414878.1] [location=complement(363231..366305)]
MTMITDSLAVVLQRRDWENPGVTQLNRLAAHPP-IRQLA**RRGPHRSPFPTVAQPEWRMALCLVSGTRSGAGKLAGVRSS*GRYCRRPLKLA
DARLRCAHLHQRDLSHYGQSAVCSHGESDGLLLAHI*C**KLATGRPDANYF*WR*LGVSSVVQRALGRLRPGQSFAV*I*PERIFTRRRKPP
RGDGAALE*RQLSGRSGYVADERHFP*RLVAA*TDYTNQRFPCCHSL***FQPRCTGG*SSDVRRVA*LPTGNSFFMAG*NAGRQRHRAFRR*
NYR*AWWLCRSRHTTSERRKPETVERRNPESLSCGG*TAHRRRHAD*SRSLRCRFPRGAD*KWSAAAERQAVADSRR*PSRASSSAWSGHG*A
DDGAGYPADEAEQL*RRALFALSEPSAVVHAVRPLRPVCGG*SQY*NPRHGANESSDR*SALATGDERTRNANGAARS*SPECDHLVAGE*IR
PRR*SRRAVSLDQICRSFPPGAV*RRRSRHHGHRYYLPDVRARG*RPALPGCAEMVHQKMAFATWRDAPADPLRIRPRDG*QSWRFR*ILAGV
SSVSPFTGRLRLGLGGSVAD*I**KRQPVVGLRR*FWRYAERSPVLYERSGLCRPHAASSADGSKTPAAVFPVPFIRANHRSDQRIPVPS*R*
RAPALDGGAGW*AAGKR*SASGCRSTR*TVD*TA*TTAAGERRATLAHSTRSATERDRMVRSRAHQRLAAVASGGKPQCDAPRRVPRHPASDH
QRNGFLHRAG**ALAI*PPVRLSFTDVDWR*KTTADAAARSVHPCTAG*RHWRK*SDPH*P*RLGRTLEGGGPLPGRSSVVAVHGRYTC*CGA
DYDRSRVAASGENLIYQPENLPD*W*WSNGDYR*C*SGERYTASGADWPELPAGAGSRAGKLARIRAARKLSRPPYCRLF*PLGSAIVRHVYP
VRLPERKRSALRDARIELWPTPVARRLPVQHQPLQSTATDGNQPSPSAARGRRHMAEYRRFPYGDWWRRLLEPVSIGGIPAERRSLPLPVGLV
SKI
>NC_000913.3_cds_NP_414879.3_342_1 [gene=lacI] [protein=lactose-inducible lac operon transcriptional repressor] [protein_id=NP_414879.3] [location=complement(366428..367510)]
VKPVTLYDVAEYAGVSYQTVSRVVNQASHVSAK-NAGKSGSGDGGAELHSQPRGTTTGGQTVVADWRCHLQSGPARAVANCRGD*ISRRSTGC
QRGGVDGRTKRRRSL*SGGAQSSRATRQWADH*LSAG*PGCHCCGSCLH*CSGVIS*CL*PDTHQQYYFLP*RRYATGRGASGRIGSPANRAV
SGPIKFCLGASASGWLA*ISHSQSNSADSGTGRRLECHVRFSTNHANAE*GHRSHCDAGCQRSDGAGRNARHYRVRAARWCGYLGSGIRRYRR
QLMLYPAVNHHQTGFSPAGANQRGPLAATLSGPGGEGQSAVARLTGEKKNHPGAQYANRLSPRVGRFINAAGTTGFPTGKRAV
>NC_000913.3_cds_NP_414880.2_343_1 [gene=mhpR] [protein=mhp operon transcriptional activator] [protein_id=NP_414880.2] [location=complement(367587..368420)]
MQNNEQTEYKTVRGLTRGLMLLNMLNKLDGGAS-SRAAGGTQRPASHHCAATAGDAAGRGICPP*PLR**FSTDHQSAAIKRRIS*RTVDFCT
GGPTAGRSVARSGMADRCVHAGC*CNGGTRNHSPFQPLILSPGNGRATFAASENRLGPDLAGLLPGTRPQGINRNVSLPPR**LSTGTGTVKA
GSHSGARAQRGLRTELPRLGSGGEDRLYRRTAAQ*TTGDWLSESGVYGERNDH*TGSGKASSGATTGSKTDRRRG*IAGYSGGRKAKRHAFTL
>NC_000913.3_cds_NP_414881.1_344_1 [gene=mhpA] [protein=3-(3-hydroxyphenyl)propionate hydroxylase] [protein_id=NP_414881.1] [location=368611..370275]
MAIQHPDIQPAVNHSVQVAIAGAGPVGLMMANY-TRPDGH*RAGGGETR*VDRLPACDWY**RGAAHHAVGRPGR*CSAAHYAVARDAFSHPE
RPLFC*YSANDR*IWLAAP*RLYSAAGRCGDAGRGVAFSECALLVFPRAGGLQSAR*RSDLAPENGRRAAGNSQSPVAGSL*RWSKFCPSHSE
CAV*R*NCAKSVDCGRYRQRSVKYAAYLFVLRSGAPVCFCRAASCGTSL*IYGDAGRNRRAAA*AAKYAQAVKQSAA*SGQC*IDSPACLHPQ
RATGATFPY*SRTAGGRCRAHHAGMAGAGL*QWYARRL*PRMETGVGYPGESPRCAARYLSTRTSRSRQSDD*PVRDGGQRAGSAETLAGYVT

*RRFLAVELSAASKTLLPRNALQADAAILRRCADA*GRSEALSGRQDVYSAESHAGKRRRDAAR*RDRRELRGNWLGMQSTVGDERRANPAVA
RVGHTLHSGGAGSANSYRTG*PRRRTTRGRYARSPA*LVRATQCFAGGDAPGSLCCRHRHSANPGQDPE*TGVGDDADPP*CRRFCRKGSL
>NC_000913.3_cds_NP_414882.1_345_1 [gene=mhpB] [protein=2,3-dihydroxyphenylpropionate 1,2-dioxygenase] [protein_id=NP_414882.1] [location=370277..371221]
MHAYLHCLSHSPLVGYVDPAQEVLDEVNGVIAS-SPRAYCGILP*TGGAVCARSLQRLFL*RDATVLFRRWSDGNW*FRQCGRRAARACGAGG
GLCACRHEERDRSCRFLLYAGGPRVRPAAGVPARWAG*GASSACVHQRCRHAAARFPAYPHVG*SHWTFHQHSQ*TRAVPGFRWAFPSAAGAR
TGESRCPYARPSVGERERFTRQ*ARIASATGD*RR*EVC*GSENAASAQPDLG*PVHDFAGAGTHTGTGCRQ*RRAFRHCRKVDT*NQNLGRR
FCRYFCVWQLA*RRALLPPNPGVDCRIWLVKRQNREL
>NC_000913.3_cds_NP_414883.5_346_1 [gene=mhpC] [protein=2-hydroxy-6-ketonona-2,4-dienedioic acid hydrolase] [protein_id=NP_414883.5] [location=371239..372105]
MSYQPQTEAATSRFLNVEEAGKTLRIHFNDCGQ-RRRNRCPAAWFRPGCYWLGELQPQYRSAGRGGLSGDPAGLSGLGQERFGR**WFAIGS*
CTNPEKRGGSTGYRQNPPAGQLDGGP*FCGVHP*MAGARRQTGADGRRYGRHEFVYADANRRY*ATESALSSADYRKPEADDGYLRF*YQRFD
RRPV*SAPE*YAVAPRSPGKLR*EPGS*SETVPGFWPTSGGNQSANPDCLGAQRPLCADGCGSASAVRHCRF*TAYLPRLWSLGAVGTCRRFQ
STGAEFPRTPL
>NC_000913.3_cds_NP_414884.2_347_1 [gene=mhpD] [protein=2-keto-4-pentenoate hydratase] [protein_id=NP_414884.2] [location=372115..372924]
MTKHTLEQLAADLRRAAEQGEAIAPLRDLIGID-KR*SGLRHSAHKCAT*RCAGASRGRA*SGPDTSESATTTGR*STGFWDVICRHVLWR*R
NHSFFPCSATPH*SGDRTGVEPRFARNRYHLRRIV*RH*MGTSGAGSGGEPHSRLVDSVCRYRGR*RLLWGVCHRRSGATSGGVRPEKLRHED
DA**RRGF*RARQRMPGTSA*CGRLAGTQNGQSG*TAAHRRYHSYRGIRSDGGGECGRSF*SPY*RHRFSCCDIFKRSPKRKSVM
>NC_000913.3_cds_NP_414885.1_348_1 [gene=mhpF] [protein=acetaldehyde-CoA dehydrogenase II, NAD-binding] [protein_id=NP_414885.1] [location=372921..373871]
MSKRKVAIIGSGNIGTDLMIKILRHGQHLEMAV-NGWH*SSVRRSGARQTYGRRHHP*RGDRTDEHA*IC*YRHCI*CDQRRCSCEKRCRFTR
SETGYSLN*PDACCHRPLLRAGG*PRGERRSTERQHGHLRRPGHHSNGGGSFTRGACSLRRNYRFYRQ*ICRTWHACQYR*IYGNHFPSH*SG
GRRGKREGDYCA*PSRATVDDA*HGVCIERRSFTR*YRSLNQ*NG*GGAGLRTGLSPETARAV*SYPAG*TGQFTGRGAILRTENSGLAGSRR
RSALSACLCGQPRHYDFQCAGDSGKNGPVTGAQGRRSGM
>NC_000913.3_cds_NP_414886.1_349_1 [gene=mhpE] [protein=4-hyroxy-2-oxovalerate/4-hydroxy-2-oxopentanoic acid aldolase, class I] [protein_id=NP_414886.1] [location=373868..374881]
MNGKKLYISDVTLRDGMHAIRHQYSLENVRQIA-KSTGRCPRGFD*SGPRRRFARFQL*LWFRRT*RP*MD*SGGGCGEARQNRDVVAARNRH
YSRSEKCLAGWRAGGSCGNALYRS*CFRPAYSVCPRARNGHRWFSDDEPYDHAGESRQAGKADGRLRCDLYLCGGFWRCDEHERYP*PFPRPE
SRAETRNANWHARSP*PESWRGELYRGGGRGLRPNRRQPRGNGRGRR*RTAGSVYCRRG*TGLAAWDRSLCVNGCRRRPGASVAGSTGTSRSR
NAGAGIRWCLLELPASL*NGGGALWLKCGGYSR*AGQTPDGWRPGGYDR*RGAGSAQQQI
>NC_000913.3_cds_NP_414887.2_350_1 [gene=mhpT] [protein=3-hydroxyphenylpropionic transporter] [protein_id=NP_414887.2] [location=375459..376670]
MSTRTPSSSSSRLMLTIGLCFLVALMEGLDLQA-SWHCGGWHRPGFRTR*NANGLDI*RRNTRFATRRVGWRNAGGPLWSQAHFDWLSCAVWF
VLTGNGDCLGFPLTGLCAADDRCRAGGGVAESYRPDV*SRGSTFSWDGSEPDVLRCSHWRGAGGDTGFRGGKLSMANGVLGRWCGAVDSGAAI
NALAAGVGGFRWRKTVCATTACLICARNGNRDAAAVVVLFLHSAGGLHVDQLATATFGGARIPAIAGGRGDVCPANGGGKRDVNVGRIDG*AA
SSNHVATDL*RHVSFAAGAWNGVVI*RYVAGGICRGVVCDRWAKRFVCPGTVVLQFADPRNRCGNSRGGRASGGYERSVTGRENAGIRHWHGR
RNGRFCTGYSCCWVGGVYFDEPEITNTAVRRCL
>NC_000913.3_cds_NP_414888.4_351_1 [gene=yaiL] [protein=DUF2058 family protein] [protein_id=NP_414888.4] [location=376772..377311]
MAKLTLQEQLLKAGLVTSKKAAKVERTAKKSRV-TGA*SSGGGRRK*KGTA*A**TA*RTAKTSGVGERI*SSGEAAY*NEPNHHCQWRYWF*
LH*RQSD*EDFCR*AHTGAVD*WSSGDCPLVGR*Q*RR*IRHYSRQRCR*NCSARCQQYSVAQRAQRRRAG*RRPVCRLQSA**FDVV
>NC_000913.3_cds_NP_414889.1_352_1 [gene=frmB] [protein=S-formylglutathione hydrolase] [protein_id=NP_414889.1] [location=complement(377535..378368)]
MELIEKHVSFGGWQNMYRHYSQSLKCEMNVGVY-TPTKSRE*KIAGAVLAFRPDLQRAEFHY*IGDAALRG*AQHYCCCAGHQSARQSCRRC*
PLRSRARCRVLPERDASAVE*TLQNV*LYPQRAAGFSDASFSGNGQKVYLWSFYGRAGRAGAGVT*PR*ICQRLGVFAHCLPIASAVGTASLC
CISC*K*RCLVGLRPGESYFTRSTRCGNHG*SGVE**FLRRTAADSKS*KDLPGDEYQDVNPLSRGL*SQLLFCLQFYWRAYCLPRQ*TEYAL
>NC_000913.3_cds_NP_414890.1_353_1 [gene=frmA] [protein=alcohol dehydrogenase class III; glutathione-dependent formaldehyde dehydrogenase] [protein_id=NP_414890.1] [location=complement(378462..379571)]
MKSRAAVAFAPGKPLEIVEIDVAPPKKGEVLIK-SHPYRRLPYRRIYPLRR*PGRCIPGGSRSRRGRRCG*SR*RRNQRQTWRPCDPALHRGV
RRV*VLSFWQN*PLCCGSRNPG*RPDARRHHPFFLQRAAALSLHGVLYIQ*IHRSRGSVSGKN*SRSKP*TRLPAGLWRDHRYWRGTQHS*SP
AR*FCCRVWSWRDWSGSGSGRASGESGSDYRYRYQPEEIRSGASLRCYRLH*PE*LRQTDKRCPVGYQQMGYRPYL*MHR*RQRDACGAGKCA
PRLGSVGDHRGRGCRSGNLHPSIPVGHWSRMERFRVWRRERSFPVTGHG*RCDER*YRSGTVCHAYHEPG*N**RLRPDA*RQIHSNRNSLL
>NC_000913.3_cds_NP_414891.4_354_1 [gene=frmR] [protein=regulator protein that represses frmRAB operon] [protein_id=NP_414891.4] [location=complement(379606..379881)]
MPSTPEEKKKVLTRVRRIRGQIDALERSLEGDA-RMPCHTPTDRCRSGRG*WADGRSA*KPYPGNV*PK*LLQPRSQPIR*RHY*TGSCLS*I
>NC_000913.3_cds_NP_414892.1_355_1 [gene=yaiO] [protein=outer membrane protein] [protein_id=NP_414892.1] [location=complement(380069..380842)]
MIKRTLLAAAIFSALPAYAGLTSITAGYDFTDY-IWRSWQP*FSVC*TGGES*KRNAAF*SFPGAS*L*N*TFQCHSRSGCGLV*ME*LADNP
NGYCLCG*YAGLCPPGFSSGY*PGPVTKNAFHDRLSLH*ILR*CRSRCLARRRITLYWPGDHQLPLYPL*LQRCRW*L*QYDFRASE*PARHW
LYATMAKPRNRCLHL*LDARNTLRQHEERQSATYSTAD*AT*SRADGR*SVVRHPNR*F*RSATCSPSDLEIL
>NC_000913.3_cds_NP_414894.2_357_1 [gene=insC1] [protein=IS2 repressor TnpA] [protein_id=NP_414894.2] [location=381351..381716]
MIDVLGPEKRRRRTTQEKIAIVQQSFEPGMTVS-TRCPATWCSSQPVISLA*AIPGRKSYCCRRRRTGCSCL*TCCRHEAD*RTPAPARQEND
GK*TPQRSR*IWTGKKVDSARALIARGWGV
>NC_000913.3_cds_NP_414895.1_358_1 [gene=insD1] [protein=IS2 transposase TnpB] [protein_id=NP_414895.1] [location=381674..382579]

VDSARALIARGWGVSLVSRCLRVSRAQLHVILR-TNR*LDGWPPQSSH**YGCASPYTPCYRRAANVWLSSGMGAASQTGRT*WYACDQCQTC
LPDHAPECAVA*AKTCCTAIETGTYRQSGRERKQSAMVL*RVRVLL**RRETACHVRAGLL*S*GTALGGHYRRLQQ*NSTGRHAGSGGTPLR
QRSSVVSSGVADG*WFMLPG**NTPVRPDVGT*TEEHGGAESGE*RNSRELRENDKA*LHQYHAQTRRVNGSKEPCRGVRAL*RMASA*CAGL
SLATGISAAAGL*WVK**QMSGNI
>NC_000913.3_cds_NP_414897.1_359_1 [gene=yaiP] [protein=putative family 2 glycosyltransferase] [protein_id=NP_414897.1] [location=complement(382739..383935)]
MKTWIFICMSIAMLLWFLSTLRRKPSQKKGCID-SHYTCV*RRPVSGAVTG*STA*PLFLPGNLR*RRLHGQYRSGHGGSQTQMGRPLCCRHA
KKYR*RWCADEWPQLRHLRPGFFK*CRHLCSARSRRNGLYAGRN*ARCRCRRRHSLYCVERRGSVTAHPRDRKVADDCYEAHATAAPGWRTVY
YQRCLRDVPY*CIA*VRFLGSY*SRRP*SHLDIGGKRLPYSAGESLHRIPTGMQQPA*GVASLAALDCGIRGLYAPA*KTFI*PLRYLQYISY
AVGCALWRWDLSHYLV**IHHHRAAWSGVGNVSAYLGRRSLCYWCF*RLVSSLLVVGAFSAAFRCVCIISLCHLDYLWTYCLFYWTRTSARQT
HPLFRTGGSVNRLFPTFCHRN*KTI*SL
>NC_000913.3_cds_NP_414898.4_360_1 [gene=yaiS] [protein=putative PIG-L family deacetylase] [protein_id=NP_414898.4] [location=complement(384059..384616)]
VDKVLDSALLSSANKRKGILAIGAHPDDIELGC-RRIACSSCAKRNLYRSRGDDYRQLWHRWNNRSP*RIAQRPKDIRVPPNYSS*FC*HPRS
FTAQ*YDFRPGRHH*KSNSF*C*NHAGIYHA*CRPPSGSSCCLSSFNGCLPNYSANSRLRNPKYLAFIYASGF*IR*RRIFHSQTCCIKKT*K
L
>NC_000913.3_cds_NP_414899.2_361_1 [gene=tauA] [protein=taurine ABC transporter periplasmic binding protein] [protein_id=NP_414899.2] [location=385232..386194]
MAISSRNTLLAALAFIAFQAQAVNVTVAYQTSA-RTGESGSGRQHLC*RKRSNRGLA*V*QRSQHRAGAGFRRRANRQPRFQPVSGCSQPTGA
D*SLLAGVKTG*LRSAGGKENYQQTGRSDWQTHRRTVYLHHPLQPAGGTETLGH*TRASGDCEPAAARDYRCLAAGRY*WCLCLGTGG*RPGK
RRQGVDRF*TGRAVGRANAGRLGGAQRFCRETS*GRESVR*KRHRCSATVHC*PRRVAETAGKHQQTGAFKRRA*R*RSGAGEGEYLSDAAAT
NGRTDRTGEQSDHRHRAVFERAGQGPGCSE*LQPVRYLALRAI
>NC_000913.3_cds_NP_414900.1_362_1 [gene=tauB] [protein=taurine ABC transporter ATPase] [protein_id=NP_414900.1] [location=386207..386974]
MLQISHLYADYGGKPALEDINLTLESGELLVVL-RAVRLR*NHPAESDCRFCALSAWQHSTGG*AY*GTGSRAWRSFSE*RATTVAQCTGQRG
VRPAIGRYRENAATGNRAPDAEKSGAGRRRKTLHLAAFRWSTSAGGDCSCAGGESPAVITRRTVWCAGRLHPRPDANPAAETLAGDGQAGAVD
YPRYRRSGVYGD*TGSAFIRPWPCAGAAAAQLCSPLCCGRVEPQHQVRSTIHRHARICFKPRI*ATGGVLM
>NC_000913.3_cds_NP_414901.1_363_1 [gene=tauC] [protein=taurine ABC transporter permease] [protein_id=NP_414901.1] [location=386971..387798]
MSVLINEKLHSRRLKWRWPLSRQVTLSIGTLAV-ITHRMVDGGDAATD*PAIFAAAATGTGKTTHHCRTARLYGRHAVAASGSQSDAHYAGAI
CSGVVRYSGRDRDGT*PYGTRHSGSDNRALSSGAAAGLFAADGDLVWYW*NLEDLTDLFSDFCTGGDVGAGGGEKRAAGSHSCRPVAGCQPCA
GAVVCHFARCAAGNPHRITYWSGGGLVYAGGGGADCRDARFRIYGSVSG*ISRN*RGAGGDRGDCDYRLSFRTGSARVTAPPDALAWRSTM
>NC_000913.3_cds_NP_414902.1_364_1 [gene=tauD] [protein=taurine dioxygenase, 2-oxoglutarate-dependent] [protein_id=NP_414902.1] [location=387795..388646]
MSERLSITPLGPYIGAQISGADLTRPLSDNQFE-TALPCGAAPSGGVSTRSSYYAAAATRAGPAFWRIAYSPCLPACRRG*RDHRAGYP*R*S
AR*RQLAYRCDIY*NATRRGDSGS*RVTFDRR*YALDQRYCGL*GALCSLPPAAEWAACGA*FP*IVPGIQIPQNRGGTSTLARGGREKPAVA
TSGGANASGER*TGAVCE*RLYYANC*CERERERSLVKFFVCPYHQTGVSGALALATK*YCDLG*PRDPALCQCRLPATATDNASGDDPWG*T
VLSGGV
>NC_000913.3_cds_NP_414903.4_365_1 [gene=hemB] [protein=5-aminolevulinate dehydratase (porphobilinogen synthase)] [protein_id=NP_414903.4] [location=complement(388753..389727)]
MTDLIQRPRRLRKSPALRAMFEETTLSLNDLVL-TDLC*RRN*RLQSR*SHARRDAHSRETSGTRN*THRQRRYSFRDDFWHLSPYR*NRQRC
LAGRWTGGAYVAHLQADRARNDRYVRHLLL*IHFSRSLRCAVRAWRRQRRDSGKFRQASRGCSCCRCRLHRPFRRDGRPGTGDSSGAGRCGI*
RYGDYVVFDQVRLLLLWPVP*SCRKRIKRRPQKLSDEPNEPS*GDS*ITAG*SPGRRLPDG*TCWSVPRHRA*AA*TY*IADWRVSGER*VCD
D*VRRAGGCYR*RESRARKLRFD*ACGCGSDFQLLCAGFG*EEDSAL
>NC_000913.3_cds_NP_414906.1_367_1 [gene=insF1] [protein=IS3 transposase B] [protein_id=NP_414906.1] [location=complement(391739..392605)]
MKYVFIEKHQAEFSIKAMCRVLRVARSGWYTWC-TAADKDKHASAVPPTLRQRCPRGFYPVKTALRCPTPDG*TACSGLPL*RKNRGGKPAPS
GTEGKGLPEVQPGQLPRTRPACVRKSVGAGFLRQWPEPEVGRRHHVLTYR*RLAVSGSGH*PVVTCRYWLVNVATHDGATGLRCPADGAVAA*
EAPERYRSHGPWRPVLFSRLSGATEAA*SAWKYERKRLLLR*CLRGKLLSFAESGMYPWRTLYQPGNNAGNGV*LYRM*LQSVAAAQLVWRPQ
SGTI*KQEPRL
>NC_000913.3_cds_NP_414907.2_368_1 [gene=insE1] [protein=IS3 transposase A] [protein_id=NP_414907.2] [location=complement(392602..392901)]
MTKTVSTSKKPRKQHSPEFRSEALKLAERIGVT-SRSP*TQPV*ITTLQLAQ*TAKSADVF*T*TGDVYRDCTSQTPAGRTG*RAGYPPKGRD
ILREAPEM
>NC_000913.3_cds_NP_414909.2_369_1 [gene=yaiV] [protein=putative transcriptional regulator] [protein_id=NP_414909.2] [location=394506..395129]
MLSVVKPLQEFGKLDKCLSRYGTRFEFNNEKQV-NIFQ*CQ*RRYFRYFRGSYLSA*RRKRTYRYYPGSLYYGAG*WFNEKRYTIQINIRRKL
YGISSTSQTNHYAY*TKSTLARRFLLVSLAK*NSGITRRAAHWA*FLRTNPRNIIIND*LE*RIAITYWCDELYPSTYTHIAFCRRRSSRCFA
*RRLYRNE*RQTGRYQPFAFRVL
>NC_000913.3_cds_NP_414910.1_370_1 [gene=ampH] [protein=D-alanyl-D-alanine-carboxypeptidase/endopeptidase; penicillin-binding protein; weak beta-lactamase] [protein_id=NP_414910.1] [location=complement(395130..396287)]
LKRSLLFSAVLCAASLTSVHAAQPITEPEFASD-NCRSLCRSYFLRQRCHGDGSGCYRR*PARLSQLWRNATW**RSPAAGFGRAYCFPHQAN
DQ*DAGEIARPGDREVKRSAK*ICSARRTRANLQWDAHYAGQSGNPYQRPAP*TARWRGTSSGVCLANARATLEIPFYGEAESRARQSGGVL*
PCVRPAGRCTGECVRQALYPVV*RADYPSIGDERHHLHPLTGSVPPFNGC*ARCQSVQ*HAGGNWQRRRLFHAWRYDALDAAVFVI*FLSAQ*
PGRSHADVDLSARAVYESDWHGCPRQSGCAWFRLGIYGPERGSSGDYSEDRRWWRFHYLYGDDPAEEYWRVCGGHSFAVDAL*KYERWHQ*PG
DRAKRE*TVGYPRVL

>NC_000913.3_cds_NP_414911.1_371_1 [gene=sbmA] [protein=peptide antibiotic transporter] [protein_id=NP_414911.1] [location=396639..397859]
MFKSFFPKPGTFFLSAFVWALIAVIFWQAGGGD-MGGAYHRRFRADPD*RRAFLVVGFPDFLRLLHCLRRTFCIVLVYLQPASLAILVNTRYCTDHLRHLVFGGSRGRRQRLVCAVL*SDSNRAKFAA*SHHRTILPRSGRLSGDCADRCGDQCAEQLLCQSLRVPLAYSDERILHGELATTASYRRGRTACAGRHHAFCFNAGEYGRQFYQRHHDVDRLPAGAGNALRACAGAADYRAHSVWSGDCRNRLVADGDRIAGSGRDQTAGAGV*KPACRGCLP*RAGLW*RRCHARDAAYGTRAV*RRTEKLFPPLFSLYVFQHRPHSLFAGR*RFRFVLAVSVNCCRYDYARPDDADYQRFWSGSRCFPVPD*LMDHTG*VDVYLQTSAQL*T*AGW*QNSGSNPYLEL
>NC_000913.3_cds_NP_414912.1_372_1 [gene=yaiW] [protein=microcin Bac7 uptake protein; outer membrane surface-exposed lipoprotein] [protein_id=NP_414912.1] [location=397872..398966]
MSRVNPLSSLSLLAVLVLAGCSSQAPQPLKKGE-KSDRCSERRAAEDACERERPRCLGERSRHHL*KSGAGTDAGKRLLGAGGGAAGVKLSGRSGRSGLKQDRLARD*PSC*TDAHSCFSGSYRAKNQIAKRQKL*RTVRFGAYRKAIERDF*RSDQHGANGADAVWFAQSGAHRWADAGQHCFCRTAYQRVSVENGRYSPSGSLQSPRRVVVWYLPFTELSRQL*RTDIPFC*F*RWLVRQP*CRVSERGQ*GQRREAGTGR*FNSLRQ*RTRENRTGNAQTGCKTGDERQRNSPSVRER*QLLF*GDGAVQESLSTCRNENG*ITPPRNVAWHSTGKPENHPQPDYGLVCEARRRTAGALYETV
>NC_000913.3_cds_NP_414913.1_373_1 [gene=yaiY] [protein=DUF2755 family inner membrane protein] [protein_id=NP_414913.1] [location=complement(399025..399333)]
MADFTLSKSLFSGKYRNASSTPGNIAYALFVLF-MLLGWGAIAEPVSACARRL*AFNAGPGNRSPTGGNWFRCRHHFRADPVFSRLPHFCSGGAMAALATSPPV
>NC_000913.3_cds_NP_414914.2_374_1 [gene=yaiZ] [protein=DUF2754 family putative inner membrane protein] [protein_id=NP_414914.2] [location=399593..399805]
MNLPVKIRRDWHYYAFAIGLIFILNGVVGLLGF-RSKRLADLCRRSGDVGD*FLAGGVDYSSSR*RN*KRPI
>NC_000913.3_cds_NP_414915.1_375_1 [gene=ddlA] [protein=D-alanine-D-alanine ligase A] [protein_id=NP_414915.1] [location=complement(399829..400923)]
MEKLRVGIVFGGKSAEHEVSLQSAKNIVDAIDK-KSLRRCAAGH**TRAMARQRCQQLSAKCRRSCPYCVAPFGDQPCAGAR*T*ASAYRRAKRSAVADGGCHFPDCPRYAGRRWFLAGNAAGRQFTVCRF*CSGFSSLYGQRCHQTSATRCRAEHCAIYYPDARQSSQH*FCRSGV*TGVTTVCKTG*SGLFCWCQQSNQ*RTVRNCRRSGVRVRS*SDR*ARDQRS*DRMRSSGQRQSASQHLWRDRTHQRFLCLRHQVH*RRWRESGSSGSHCARNQR*DPGDCRSGLSNVGMRRHGACRRVFNPRERSGDQRDQHPAWLH*YQYVSEAVASQRSGLHRSDHTFD*TGAGASRCG*RIENHNV
>NC_000913.3_cds_NP_414916.1_376_1 [gene=iraP] [protein=anti-RssB factor, RpoS stabilzer during Pi starvation; anti-adapter protein] [protein_id=NP_414916.1] [location=401386..401646]
MKNLIAELLFKLAQKEEESKELCAQVEALEIIV-NCNASQYGAK*PTAVD*SGRGGAVRGKARCQHS*RRYGAAARLRKEVIEASSSV
>NC_000913.3_cds_NP_414917.2_377_1 [gene=phoA] [protein=bacterial alkaline phosphatase] [protein_id=NP_414917.2] [location=401747..403162]
VKQSTIALALLPLLFTPVTKARTPEMPVLENRA-SSGRYYCTRRCSPFNG*SDCRSA*FS*R*TCKKYYFADWRWDGGLGNYCRT*LCRRCGRLF*RYRCLTAYRAIHSLCAE*KNRQTGLRHRLGCISNRLVNRCQNL*RRAGRRYSRKRSPNDSGNGKSRRSGDR*RFYRRVAGCHARCAGGTCDLAQMLRSERDQ*KMSG*RSGKRRKRIDYRTAA*RSCRRYAWRRRKNLC*NGNRW*MAGKNAA*TGTGAWLSVGERCCLTEFGDGSESAKTPAWPVC*RQYASALARTESNVPWQYR*ARSHLYAKSAT**QCTNPGADDRQSH*IVE*K*ERLFPAS*RCVNR*TGSCCESLWANWRDGRSR*SRTTGAGIR*KGG*HAGHSHR*SRPRQPDCCAGYQSSGPHPGAKYQRWRSDGDELRELRRGFTRTYRQSVAYCGVWPACRQCCWTDRPDRSLLHHESRSGAEI
>NC_000913.3_cds_NP_414918.4_378_1 [gene=psiF] [protein=PsiF family protein] [protein_id=NP_414918.4] [location=403281..403601]
MKITLLVTLLFGLVFLTTVGAAERTLTPQQQRM-NLL*SAGDGAGVERGCS*DLHE*LPEEQQVCAWRKKFDATAAKDARMQ*SSNTTISER**S**VYECLPQESRL
>NC_000913.3_cds_NP_414919.1_379_1 [gene=yaiC] [protein=diguanylate cyclase, cellulose regualtor] [protein_id=NP_414919.1] [location=403703..404818]
MFPKIMNDENFFKKAAAHGEEPPLTPQNEHQRS-RAALRPSRQTTPCGWPGWHVLTDCFNAGFTPAAGLVVAGVGRLGVRLAAFSLADSEQGRRSA*PGNLQLKNRCSISGNVGRRNGRKRAAFHRDVDDYVSEFDGGRRPPSVCRGSGVDGGFLPCHPRADGHYRVVQ*CAAGMVALPSHYCHLSSAVWLGQLPDGNQTGGT*TQVAGHEYPRRHDGRV*PTSLGNYVTQ*I**LSAA*SRCNVTDYRYRPFQEHQRYLGP*CGR*SDCGAYPTVTNYPAR*RCDWSVWRR*VCSNHVRYAS*ERHYRHVTGA*RAKYITFAEYATGNFTD*CGGCAAEPTNESLS*VVEIGRFGALQSKESRT*PHRSGGL
>NC_000913.3_cds_NP_414920.1_380_1 [gene=proC] [protein=pyrroline-5-carboxylate reductase, NAD(P)-binding] [protein_id=NP_414920.1] [location=complement(404835..405644)]
MEKKIGFIGCGNMGKAILGGLIASGQVLPGQIW-SIHPLPG*SRRPA*PVRHQRRRIGARSGANRRHHFCCR*TWHHD*SA*RNHLQPE*RLSGRFYCCRCHARPACPRAGP*PENYPRHAEHSRTG*CRDDLRNAKRAGNPRRYR*CAEYFPLLWRSGSNC*ADDPPGGRCERFFASLRIYVYRSDGRRRRAGRDATRPGV*ICRSGGNGFRKNGAGNGRTSGGTERYGLLTGRHHH*SGTRTGRERLPCCSDRSDDEVYGKIRKTQQIL
>NC_000913.3_cds_NP_414921.2_381_1 [gene=yaiI] [protein=UPF0178 family protein] [protein_id=NP_414921.2] [location=405764..406222]
MTIWVDADACPNVIKEILYRAAERMQMPLVLVA-KPEFTRAAIAIYSYAARRGRFRRCR*RNCPAV*SGRFGDHRRYTFGC*SHRERRCGA*SARRTLHASDHS*APDDARFYGYLTCQWDPDRRTR*PFTT*PPGLCRGAGEVVAGSAT*SWL
>NC_000913.3_cds_NP_414922.1_382_1 [gene=aroL] [protein=shikimate kinase II] [protein_id=NP_414922.1] [location=406405..406929]
MTQPLFLIGPRGCGKTTVGMALADSLNRRFVDT-RSVVAITAQYDGRGDRRKGRVGGISRQRNGGAGSGNCAIHRYRYRRRHYSDGI*SSLHAK*RDRGLFVCASISPG*PTASCTGRRFTANLNGKTAERRSSGSAGRTRCAISRSCAYYHRRNKRTQPGDF*NSQRPGTDDQLL
>NC_000913.3_cds_NP_414923.1_383_1 [gene=yaiA] [protein=OxyR-regulated conserved protein] [protein_id=NP_414923.1] [location=406979..407170]
MPTKPPYPREAYIVTIEKGKPGQTVTWYQLRAD-TS*TRLVDQ*TSDRSGSDGCEKTL*GP*QRV
>NC_000913.3_cds_NP_414924.1_384_1 [gene=aroM] [protein=AroM family protein] [protein_id=NP_414924.1] [location=407428..408105]

MSASLAILTIGIVPMQEVLPLLTEYIDEDNISH-T*PAGEVKS*RSDGGVRARSRRRHHSHIIK*QPAGPCFASQSGA*PARCG*SAR*SGL*
RHFINEYSKH**YDCA*YDLS*AVANIASTGFLYC*RSSGGGYRSG*GDAARSGAKMANFAEIAGIFIG*PHS*FRTKNH*CRERITGKRG*C
HHAGLFRISPTSSRFTAKTARCSCLAV*RIDCTAGCGITGV
>NC_000913.3_cds_NP_414925.1_385_1 [gene=yaiE] [protein=UPF0345 family protein]
[protein_id=NP_414925.1] [location=408177..408461]
MLQSNEYFSGKVKSIGFSSSSTGRASVGVMVEG-RIHLQHR*AGRDDGNQWRAECVTA*RDRLAGV*SRFGV*CSRSQ*VSSASCRTHLLSVP
LSV
>NC_000913.3_cds_NP_414927.1_387_1 [gene=rdgC] [protein=nucleoid-associated ssDNA and dsDNA
binding protein; competitive inhibitor of RecA function] [protein_id=NP_414927.1]
[location=complement(409108..410019)]
MLWFKNLMVYRLSREISLRAEEMEKQLASMAFT-TMRQPGHGEDGLGSSDGIAQRCVNARCQWSNCYLRAQRRKNPPVSGD*TGAGSENRQTG
SGTGA*AEENRKRFAERRSAALSAAACFQPF*PDNDVDRHG*RFDYGGLRQCQKSGRYAGITA*KPGVVTGCTIEHGKPD*TDADRMGSLR*C
GTGLPAA**SRAEIVAGRWRRDPREETRSDQRRDHQSH*SRKSGD*TGARLAAAHSVCDVRRWFAQASEVLRRAARSKRRYRP*RFRPAF*CR
FHPDDW*TGSVNSKPD*RIRWRSTTL
>NC_000913.3_cds_NP_414928.2_388_1 [gene=mak] [protein=manno(fructo)kinase]
[protein_id=NP_414928.2] [location=410144..411052]
VRIGIDLGGTKTEVIALGDAGEQLYRHRLPTPR-R*LPADY*NDRHVG*YGGAGDGAARNGRYGHSWLNFALHRCGEECQFNLAQRSAIR*RL
KREVAAGSAAGK*R*LSGGFRSSRWRGSGSADGICRDYRHGMRRGRGIQWAGAYRRQWHGR*VGTQSATVDGRRRTALSRGSPLLLR*TRLY*
NLYFGHGIRDGLSSFERTCAERQ*NYPPG*RKRSGSGTGIASLRAAAGKIAGTCREYSRSGCDCPGGRDEQCRPFISNGWAVD*TICLRRRM*
NAGA*GEAR*FQRRTRRCVVMATRV
>NC_000913.3_cds_NP_414930.3_389_1 [gene=araJ] [protein=L-arabinose-inducible putative
transporter, MFS family] [protein_id=NP_414930.3] [location=complement(411297..412481)]
MKKVILSLALGTFGLGMAEFGIMGVLTELAHNV-RNFDSCRRAYDLVLCTGGGGRCANHRTLFQPLLTQTYLVVSGGVVRHWQRHVHALFVLP
DARHWSAGIRLSAWRIFWRRSDRVIKNYQTRKSHRRRGGDGFRDDSRQFAGHSAGNVFKSGI*LALHLFIDRCF*YCGDGIGLFLGARYSRRG
ERKSARTISLFAQPGPVVNFRRHDVWQRRCVCLVQLRKAIHDVYFRFFGNGDDLYYDVSWARDGAGKYAKWQDFRTLFTTAHCSSD*LYNCTG
TADALFLRRHENNVAYFCFYLLRGIICPFSTATNIVTTKRQRRRVIRCRRWANSV*PR*RRRRILRRYDADAGAGI*LRGAACRPAFVCCDVV
VAAVWSL*APASGGYSGAGETTGV
>NC_000913.3_cds_NP_414931.1_390_1 [gene=sbcC] [protein=exonuclease, dsDNA, ATP-dependent]
[protein_id=NP_414931.1] [location=complement(412607..415753)]
MKILSLRLKNLNSLKGEWKIDFTREPFASNGLF-SYYRPNRCGENHPAGRHLSGAVSRNSASL*RFTIAK*SHDPRYRRMSGGGGV*SER*SV
PCILEPESGA*PTRR*FAGATRRAGALRRRQNSRRQSER*AGTDSDVNRAGLRALHPFDAAFAGAICCLPECQTQRTRGIARGVNRH*NLRAN
LGDGF*AAQIGPHRAGEAASAGQRRHVAHAGTSAIADSEFAGTY*RRKTVNYRAAARTTIAKLVNASGRIAARSQPPSAGLATGVSRRRKSAT
STGGA*SGTTGTKSSSTLGTHRRTQRGAGAYSPAD*RSKYSLTEHNGASREHSPPRGEAVSRITAAATKPEYLVTGTRPLPSVEQRTGGLACA
VLPTNQRSRASAAMAATVNPC*AKT*CACGDHVDVNRR*SCYRPGATC*ATPTASAPGRAAWTDCSPTKTSGAVTGRYPECHARTDAT*RRT*
RNAPAL*RKDAATCRCENHLRAGSAHQNAGSSTCTVTGGSALPTLWFHQPPGGRGVSGAGAWR*SVSITGAGKRS*KAR*RRCDATWATGRHN
KAASA**KRSAKPPTR*ASTYSTMASRHGQPQYHLAATGRYSTVAGCTR*ARTPAAVTQPTA*ITRADCRA*SANYPVSTAN*TTPATTFNDI
DGLCTDIATGR*RRELVGDTSARSAELAATPERINRAAKPYSAADADSGNVAAK**TPALRRNCGIGKLAAGT*TMSRITQPAADVTATGCSG
GAKSAKSPGAV*HRATGQRL*RSAGVPCGANG*TNTNAAGTAQAESGKPAPSGANSGHSDSRNAGTASTTPT*RRVGSHCDGGADSARVSANS
PKVA*KHHESRRDSPAAEAGCR*PSATTNLNAANCSNDAAG*GLGISEFANRFQRGR*IPQVCPGADAG*FSPSR*SATYPAARALSVTAQSQ
RGAGSRGC*YLAGRCGTRYPYPFRRRKFPR*SGAGAGAFGSGQP*NTY*LAVP**RFWHAG*RNAGYRP*CAGCPERQWQNHRCD*PRRSDER
AYSGADQSEKDQRPGLQQTGKYVCSEI
>NC_000913.3_cds_NP_414932.1_391_1 [gene=sbcD] [protein=exonuclease, dsDNA, ATP-dependent]
[protein_id=NP_414932.1] [location=complement(415750..416952)]
MRILHTSDWHLGQNFYSKSREAEHQAFLDWLLE-NSTNPSGGCDYCCR*CFRYRLAAQLRPHVIQPFCCQFTANWLSSGGTGRKP*LGRHAE*
IARYHGVPQYYRGRQRRTCAANLASSRRDARRSAVPHSVFTSA*HYYQPGGA*RY*KTAAFTGSDYRLLPTTLCRCLQTARRSASAHHRHGTF
NDRGGQ*K*RRA*HLYWHAGRVSGTKLSTSRLHRARAYSPRTDYWRHGTCSLLRLPHSTEF**MR*E*ICPSGDIFKRQIRERGKPERTGNAT
HGSAERRSGVDYRTAGTVARCIAGATCLAGYRNHY**VSA*YSAQNPGINRIIACRSIAGTSES*TARACVSQPTA*NPQRTQRRRGVQSPSG
TGRTG*IAAATSAASFHHDVAYPRRRTRSM
>NC_000913.3_cds_NP_414933.1_392_1 [gene=phoB] [protein=response regulator in two-component
regulatory system with PhoR] [protein_id=NP_414933.1] [location=417142..417831]
MARRILVVEDEAPIREMVCFVLEQNGFQPVEAE-RL*QCCESTE*TLAGFNSPRLDVTWRLRYPVHQTPQARVDDPGYSSGDVDRQRGRRRSR
ARP*NRRG*LYHQAVFAEGAGGANQSGNAPYFANGGGRGD*DAGIKSRPDISPSDGGRRAAGDGADRI*TAALFYDAS*ARVQPRAAVKPRLG
N*RVCGRPHGRCPHSSPA*STGARRA*PHGADRARYRISFFNPLL
>NC_000913.3_cds_NP_414934.1_393_1 [gene=phoR] [protein=sensory histidine kinase in two-
component regulatory system with PhoB] [protein_id=NP_414934.1] [location=417889..419184]
VLERLSWKRLVLELLLCCLPAFILGAFFGYLPW-IFAGIGNRTAYLAFLEFIAPFMVAVGGSQYDPATGAW*LGTATIRLTPDAAAK*KTPP*
TGQSD*TLS*RRGVAARRGGADHGRGRYFLV*RSGATNSWFALAGR*RAEHP*PTALPGVYAISENA*FFSPAQSGAQHRAASGNSRHALYPQ
TVADGGA*CHANASTGRGAA*LFCQREP*VTYAIDRVTGLPGDDE*AAAGRRGTRKSVAHHARADPADGRTGEAIADAVENRSRTDAFAQ*KG
*CADDAARC*ARGSDSESEKTDIYL*DR*RPQGVWQRRSATQCDFEPGL*RRESYAGRHAYHRTLAASAARCRI*R*R*RTGHCTGAYSAPDR
AFLSR**SAFPANRR*RIRVSDRETCCESSRKSPEY*EYSRKRNTFQFCYPGTFNCQKQRL
>NC_000913.3_cds_NP_414935.1_394_1 [gene=brnQ] [protein=branched-chain amino acid transport
system 2 carrier protein; LIV-II transport system for Ile, Leu, and Val]
[protein_id=NP_414935.1] [location=419591..420910]
MTHQLRSRDIIALGFMTFALFVGAGNIIFPPMV-RLTGRRTRLDCGIRLPHYCRWPAGINGSGAGKSWRRC*QPQHANR*SRWRTAGNGLLPG
GGAAFRYAAYSYRFL*SGDCAADG*FRAAAVYLQPGLFRYRYSGFALSGQAAGYRGQLPCAAENYRAGHPVCCRYCLAGGFYQHGD*GLSKRC
VF*RLR*RLSDHGYAGRNGVWYRYC*RGAFSWRYRSASADPLYRLGWPDGGCWSDSAVPGAVPSGVRQRVAGRSVCKRRCYSACLRSAHLWRR
R*LPAGGVNLHRLPGNGSWPDLCLCRILCPVRTALLSYAGVYPRRLLDGGF*PRLKPADPDLRTGADRYLSAVYRTGCIKFYTLMVA*FVPRD
CSADVYQPAFWYSRRDQSICIQRYLTVLGAAFTAGRTRSGVVNANSGDGGSGHYLGSRGRSSGDLQRSL

>NC_000913.3_cds_NP_414936.1_395_1 [gene=proY] [protein=proline-specific permease] [protein_id=NP_414936.1] [location=420986..422359]
MESKNKLKRGLSTRHIRFMALGSAIGTGLFYGS-SRRHQNGRSERVVGLYYRWYRGVYHYACAGGNVGT*PGRQLFLALCAGKPRPAGRLHYR
LDLLL*NPYCRHRRCDRFWYLYGCLVPDGAALDLGTERGADHLRRKPDEREGIR*AGILVLVL*SRHHHHHDCRRFRHHHLGDWQRRATDRYS
*PVEQRRLLQ*RLAWHGNVVANGDVCLRWDRNYRDYRR*SERS*EIDTACD*LRADAYSGVLRRYAVRHYVYLPVESGWHCR*PVRADVPAYG
HYLCRQHS*LCCADCFAVGN*Q*CIWRRPYAPRYGRAGQRAENFQQNVASRYSVGYGAGDDYRAAVCGVSELHHAGKRLPGDRFAGNLRHGVG
VDYDPAVANCLPSPFAARRS*GAEI*SAGWGSNDHRRADFPALYYRVDWLSPGYAYLAVCRFRVDCCAVDWLDV*TPPRSSAG*KPV
>NC_000913.3_cds_NP_414937.2_396_1 [gene=malZ] [protein=maltodextrin glucosidase] [protein_id=NP_414937.2] [location=422518..424332]
MLNAWHLPVPPFVKQSKDQLLITLWLTGEDPPQ-THYAAYRTR*RRNVSTDA*AAQSAAAWRHRMACGD*SLQRTTPAALQFQTAVARSPALV
YTAGLQPNAAGTTGAVCRRCTGYRPTMGCGSDFLSDLP*SFCA*SSS*S*TGSCLLPSCSRTRDHLA*LG*TGHGAGGRINVLWRRSGRDKRK
TAVSEKAWRDSAVSQSGV*SSQRT*IRYRGLSPCRSAVWR*WGVAAFATQYAAAGNAAGAGRRV*PQWRFPCLV*QA*SWHGWCLSQPRIALA
RLVLV***WHGARLAWLCQLAEAGLSVGKSGE*NLSRGRQYCPPLAESAVEYGRLAAGCGAYAGGGGWGAQ*YAARCRDHRSGERNPAGSVYC
RRTFWRCTAMVTGRCGRCRHELSWLHIPVVGISCQYRYLLRSAAN*CPNLYGLDG*LPRRAFSSTTITYV*SARQPRYCAI*NAARSGYCAPA
AGGGLAVHLAWCTVHLLR**SRTGWQKRSVLP*TVPLAGGKAGYGVIRAVPANDCAA*EKSGATSWRLSGAVCGR*RGGICPRAESATCTGGN
QPWRGL*SGATRVTVSQCRAMAMQRRAWATD*RDSGFACHFGYGMDEL
>NC_000913.3_cds_NP_414938.1_397_1 [gene=acpH] [protein=acyl carrier protein (ACP) phosphodiesterase; ACP hydrolyase] [protein_id=NP_414938.1] [location=complement(424337..424918)]
MNFLAHLHLAHLAESSLSGNLLADFVRGNPEES-ISARRRGWHSYASTYRRID*QSAGSPRSTGVVS**NAPRCAYYAGCHVGSLSFPPLVAA
VAGLSATGICLLCPRASDDDFAGLTATFYQSEQLLVVRAVAGALSRYGFHPERVKRHGKPPPTSGCPA*LLVRFRRSL*RPRNPLLAVLSADD
GAGVTQGVI
>NC_000913.3_cds_NP_414939.1_398_1 [gene=queA] [protein=S-adenosylmethionine:tRNA ribosyltransferase-isomerase] [protein_id=NP_414939.1] [location=425011..426081]
MRVTDFSFELPESLIAHYPMPERSSCRLLSLDG-TDGRADARYFHRFT**AQPRRSSGF**YPRDPGAPVWA*SQRRQD*SAG*TDARRQTHS
CAYSRLESAKTWRRTAAGR*RKY*RNNDRAPRRTV*SRI***TLGAGYSQQHRPYAAAAVYRPSGRRR*PRTLSNRL*RKTGRGCSPDRRSAF
*RAFAGKIARQRRGDGVCDVARWCGHLPAGARRHH*RSHHALGIR*STAGCGRRGTGGESAR*PGDCGWHHFSTFAGKRGSGSEKRSH*TVLR
RYPNLYLSGLPVQSGRCAGDELPLARVDADYAGFGLCRLSTHHERL*SSGRREISLF*LR*CDVYHVQSAGN**ARRGV
>NC_000913.3_cds_NP_414940.1_399_1 [gene=tgt] [protein=tRNA-guanine transglycosylase] [protein_id=NP_414940.1] [location=426137..427264]
MKFELDTTDGRARRGRLVFDRGVVETPCFMPVG-NLRHRKRDDAGRS*SHWRANYPRQHLPPVAAPGPGNHETARRSARFYAVEGADPHRLRR
LPGLQPWRYS*NHRTGRALP*PDQRRSDFPRS*KINGDSVRSWFGYRHDL**VYAVSC*LGLRKTLHGDVSALGEA*P*AF*QSRKQKCAVWY
HPGQRLRRFT*YFC*RSGRYRF*WLRCRRSGCG*AESRYAPHSGACMPANSGRQTALPDGRW*TRRPG*RRTSWYRYV*LRNANPQRPKWSFV
RDRWRGENPQCEV*ERYWPTRS*V*LLHLSQLFTRLLASS*PLQRNIRRATQHHS*PSLLPAFDGGFTQGY*RG*IRELRN*FLPASGARSTT
FER*L
>NC_000913.3_cds_NP_414941.1_400_1 [gene=yajC] [protein=SecYEG protein translocase auxillary subunit] [protein_id=NP_414941.1] [location=427287..427619]
MSFFISDAVAATGAPAQGSPMSLILMLVVFGLI-ILFHDPASTAEAHQRTQKADGLHCQR**SSDERWPGWSRNQSSGKRLHCYRAE*HH*SS
Y*T*LRSCRPAERHHEGAV
>NC_000913.3_cds_NP_414942.1_401_1 [gene=secD] [protein=SecYEG protein translocase auxillary subunit] [protein_id=NP_414942.1] [location=427647..429494]
VLNRYPLWKYVMLIVVIVIGLLYALPNLFGEDP-SCSDHWCARSRRQ*ANADPGPENVTRRKNNC*VCGTGRGRYSCALRLH*HPVARS*SIN
GRYG*QIRRGA*PCPGNAALAGSYSR*ADEARP*PAWRRSLPDGSGYGHRAWQTPGTKYR*PTQ*PARKGHPVYHCS*RKQLRPEHHFPRC*S
S**SHCVSEQAPSGPGD*QPGQQPAACGNERCSSE*SA*ICGAAEH*YPA*PCKPTWRGGAGGSASGC*PYRC*TARYSGHCACERDSGCDGN
AGIPSGKHQR*PGRCGIRSRTGRL*SETDPRRSASCAVQTRNSDR*PYHRLHFQPGRIQPTAG*HLAR*RWW*HHV*LH*GQHRQTDGNPVCG
VQRQR*ERCKWSCGSGETGRGD*HRQHPVSSG*QLPYHRHQQPERSPSAVTAAACRCVDRADSDC*RTYHWPNPGYAEH*TGAGGLPCRSAGF
YSVHDHLL*EVWSDCDQCSDCQLDLNRRHYVAVARRNAEYARYRGYRLNPCGGGRCERTDQRTY*RRVEQRTYCSTGN**RLSWRIQFYLRCE
HHHAD*SHHPVRSGYRGN*RVRDYYRYRCGDVDVYRDCRYACHRKPAIWRQARQEAVNL
>NC_000913.3_cds_NP_414943.1_402_1 [gene=secF] [protein=SecYEG protein translocase auxillary subunit] [protein_id=NP_414943.1] [location=429505..430476]
VAQEYTVEQLNHGRKVYDFMRWDYWAFGISGLL-INRCYRYYGRARL*LGAGFHRWYGY*NYARKTG*N*RNA*CIAKSRF*RADAAKLW*QP
*HHGPYAAC*RRNRRSGVGQPGSEGD*RIHQSECSSEAY*VRRSERGGRPCANRCDGVDGSAAVYPRVRRFPL*VATGGRGGYCAGARRYHYA
GYFVVIPYRD*PDHCGIVDVGYRLLA*RQYRGIGPYS*KLPQDPSRYALRNL*RVLDPDAAPYLDHIRYYLDGYPDAVPLRWSGTGRLLADHA
YRCFHRYCIFHLCGICVGSETGYEARTHVAAESGKRRGGSAVNSAV
>NC_000913.3_cds_NP_414944.1_403_1 [gene=yajD] [protein=HNH nuclease family protein] [protein_id=NP_414944.1] [location=430605..430952]
MAIIPKNYARLESGYREKALKIYPWVCGRCSRE-ICLFQPA*TYRSPH*SRPYQ*PGRWQ*LGIVVSLLPRS*AFEIYRSGSVWYDRYRRGRC
AERCR*SEVQPIR*PESDDEQEEV
>NC_000913.3_cds_NP_414945.1_404_1 [gene=tsx] [protein=nucleoside channel, receptor of phage T6 and colicin K] [protein_id=NP_414945.1] [location=complement(431129..432013)]
MKKTLLAAGAVLALSSSFTVNAAENDKPQYLSD-MVAPER*RCRKLSHPFRTADPQRYLP*VRSIR*KRLVRLLWLCGCAGILRR*LRC*RYL
EPRFSAVYGNRTTFLHRQADQY*P*LRSVQRVVLREQLHLRHGS**RWSPEHLVHGSGYRYRHWPADEPVHERLCEIPVAELWRSERKRVGRL
PFQN*ILCADYRSVGRSAELHRLHQLRLGFRFRG*QR*RNQRY*DPY**LYRFQPYSGSELRSLALLCRSSLLARRWSVERRCRTELRQRQLQ
RSLYRLGWLPGSRLQLL
>NC_000913.3_cds_NP_414946.4_405_1 [gene=yajI] [protein=putative lipoprotein] [protein_id=NP_414946.4] [location=complement(432312..432851)]
MNTNVFRLLLLGSLFSLSACVQQSEVRQMKHSV-KHAEPGDDATQSRNSQNHSAKQAECKIQQWGLPSAWSENTGKTGKPDRYFTYVAGEYYA
*CRWHHPYTTYSG*VE*SLARIQRNC*IWANSGNNRQLSGNQCAESID*CPCQRPRPQRC*YSVTVKGDFCGSVRLCTYPRHSTCYAV

>NC_000913.3_cds_NP_414947.1_406_1 [gene=nrdR] [protein=Nrd regulon repressor]
[protein_id=NP_414947.1] [location=433002..433451]
MHCPFCFAVDTKVIDSRLVGEGSSVRRRRQCLV-M**TFHHL*SGGAGYAACCKKQRRA*TV**REIA*RNAAGAGKTSGEFR*RRNGNQSY*
IAAARHR*ARSAEQDDWQSGDGAIEKAR*SRLYPFCLCLPQFRRYQRIWRRDRAPGGL
>NC_000913.3_cds_NP_414948.1_407_1 [gene=ribD] [protein=fused
diaminohydroxyphosphoribosylaminopyrimidine deaminase and 5-amino-6-(5-phosphoribosylamino)
uracil reductase] [protein_id=NP_414948.1] [location=433455..434558]
VQDEYYMARALKLAQRGRFTTHPNPNVGCVIVK-RWRNCR*RLPPTCG*TTCRSTRVAYGG*KSQRCDRLCHTRTL*PSWSYATVL*RTHRRW
RSARGCLDARS*PAGRWAWTLPSATGWH*RQPRPDDE*SRAIE*RLSQADAHRLSLYSVKTWRIA*WSHGDGERRKPVDHFAPGAA*CTTTAR
AKSCHFNQQRHGAGG*SCLNGALV*TG*TNSGALSATKSPSADTYCD**PKSRDAGTSHCAAARRNLVRAYAGRFS*VAGNGAYLADSRA*RS
SGSGCTDDATG*TAN*QHLGGSGANARWRIAAGGFSR*ADCLYRT*TIRQRRPRIMHAARA*EISRRPPI*IQRDTSCRPGCLPAFSGCM
>NC_000913.3_cds_NP_414949.1_408_1 [gene=ribE] [protein=riboflavin synthase beta chain]
[protein_id=NP_414949.1] [location=434647..435117]
MNIIEANVATPDARVAITIARFNNFINDSLLEG-SN*RTETYRSGKR*KHYRCLGAWCL*AAAGGGCTG*NR*IRRGDCAGYGYSWWHCPL*I
CRWWCKQRPGACCPGQRNSGCFWGSDH*KH*TSDRTCWHQSWQQRCRSCTDRA*ND*CIESHQGL
>NC_000913.3_cds_NP_414950.1_409_1 [gene=nusB] [protein=transcription antitermination
protein] [protein_id=NP_414950.1] [location=435137..435556]
VKPAARRRARECAVQALYSWQLSQNDIADVEYQ-IPG*TGCKRR*RPVLP*AAGRGGD*YRIPRRTDEAIPVPPAGRTGTGRKSSTAHCAVRT
V*T*RCAIQSGH*RSDRTGEIVRRRRQP*VRQRRTR*SSTCDSP*QKV
>NC_000913.3_cds_NP_414951.1_410_1 [gene=thiL] [protein=thiamine monophosphate kinase]
[protein_id=NP_414951.1] [location=435634..436611]
MACGEFSLIARYFDRVRSSRLDVELGIGDDCAL-TQYPRETDPGDQH*YAGGG*PFPP*YRSC*SGL*STGGEPKRSGSDGGRSGLADAGINL
TGRRRSVA*VLQRQFV*SSQLLRYATHWRRYHAWAIINDVGYPRLCSDGTSLNALWGETG*LDLCDRYTGR*RRRAGDFAKPFAGCRC*RCGL
LDQTSSPSIAAYFTGAGTARSGKFSHRSL*RFDFRSRAYRESQRLRRTY*PGIAAVF*CAFSPC*TGTGAALGALWR*RLRVVFHCAGTEPWR
AGCGSRTPGRTVYLYRANDRRYRRALFYS*RRTCYIRLERI*PFCHAI
>NC_000913.3_cds_NP_414952.1_411_1 [gene=pgpA] [protein=phosphatidylglycerophosphatase A]
[protein_id=NP_414952.1] [location=436589..437107]
MTILPRHKDVAKSRLKMSNPWHLLAVGFGSGLS-TDRSWDDGLAGSDSVLVSDDLFALAALLAGGDAGDLYRRLSLSSNGERHGCARSWQHCL
GRIYWYVDHAHGAADQ*LAVGCRRVCDFPYSGYVEAVADPLV*SQCAWRHGDHDRRYCRRGDFRRHPVFYRSSLAAGYSVV
>NC_000913.3_cds_NP_414953.2_412_1 [gene=yajO] [protein=2-carboxybenzaldehyde reductase]
[protein_id=NP_414953.2] [location=complement(437161..438135)]
MQYNPLGKTDLRVSRLCLGCMTFGEPDRGNHAW-NTAGRKQPSHN*TCTGRRHKFL*YRQQLF*RQQRRDRRSRTAGFRPS*RRGRCDQSVPS
RW*FTGRIIPCANFALYRRQPATSRHGLCRYPANSSLGLQHADRRDAGSPQRRGKSRESALYRRVINARFAVCSGTGTPKTARLGAVCQYAGS
LQSDLS*RRARDATTVLSGGRGGNSMEPAGKGPSDASVGRNYRTTGV**GGEKSL*RKR*K*RADRRAVNRRQ*RTGGDTSTSCAGLVVE*TG
HCRTDYRNFARRTA**AIERGGYHFEAGTDCRTGNAV*TASCRRI*I
>NC_000913.3_cds_NP_414954.1_413_1 [gene=dxs] [protein=1-deoxyxylulose-5-phosphate synthase,
thiamine triphosphate-binding, FAD-requiring] [protein_id=NP_414954.1]
[location=complement(438315..440177)]
MSFDIAKYPTLALVDSTQELRLLPKESLPKLCD-RTAPLFTRQREPFQRALRLRAGHGRTDRGAALCLQHPV*PIDLGCGASGLSA*NFDRTP
RQNRHHPSERRSAPVPVARRKRI*RIKRRAFINLHQCRNWYCGCCRKRRQKSPHRLCHWRWRDYRRHGV*SDESRGRYPS*YAGDSQRQ*NVD
FRKCRRAQQPSGTAAFR*ALLFTARRREKSFLWRAAN*RAAQTHRRTY*RHGSAWHVV*RAGL*LHRPGGRSRCAGAYHHAKEHARPERPAVP
AYHDQKRSWL*TGRKRPDHFPRRA*I*SLQRLFAEK*RRFAELFKNLWRLVVRNGSERQQADGDYSGDA*RFRHGRVFT*IPGSLLRRGNCRA
TRGDLCCGSGDWWVQTHCRDLLHFPATRL*SGAA*RGDSKASGPVRHRPRGHCWC*RSNPSGCF*SLLPALHTGNGHYDPER*KRMSPDALYR
LSL*RWPVSGALPAWQRGRRGTDAAGKTTNWQRHCEASWRETGDP*LWYADARSGESRRIAERHAGRYAFCETA**SVNSGNGRQP*SAGHRR
RKRHYGRRRQRRERSADGPS*TSTRAEHWPAGLLYSARNSGRNARRTRPRCRWYGSQNQGLAGI
>NC_000913.3_cds_NP_414955.1_414_1 [gene=ispA] [protein=geranyltranstransferase]
[protein_id=NP_414955.1] [location=complement(440202..441101)]
MDFPQQLEACVKQANQALSRFIAPLPFQNTPVV-RNHAVWRIIRW*APATFPGLCHRSYVRR*HKHAGRTRCRR*VYPRLLINS**FTGNG**
RSASRFANLPCEVWRSKRDSRWRRFTNAGVLDFKRCRYAGSVGPRQNFDDF*TGERQWYCRNVRWSGIRFRRGRQTRTSGRA*AYSSS*NRRI
DSRRRSPWCIKRRR*RTSCSAGTRQVCREHRPCLPGSG*HPGCGGRYCNVGKTPGCRPATW*KYLPCTSGS*ASPEESPGSDRRCPSVAETTG
*TVTRYLGTGSASGLHHPA**I
>NC_000913.3_cds_NP_414956.1_415_1 [gene=xseB] [protein=exonuclease VII small subunit]
[protein_id=NP_414956.1] [location=complement(441101..441343)]
MPKKNEAPASFEKALSELEQIVTRLESGDLPLE-RGAERVRTRRAAGTSGAGQITTSRTARTNSAV*Q*RRLSNPFYTGQ*V
>NC_000913.3_cds_NP_414957.1_416_1 [gene=thiI] [protein=tRNA s(4)U8 sulfurtransferase]
[protein_id=NP_414957.1] [location=441549..442997]
MKFIIKLFPEITIKSQSVRLRFIKILTGNIRNV-IKAL**DARCRPPLG*HRSSRKR*KPASGYSRRSDPYSGYPPYSRSRRRAVYRHARYFR
ESVGSVSRSAGRQNLLRTREAPWQT*F*LD*CGTLRRRRFKSAY*IRAREADQSGCDCPSGSGRRSSPAD*RPLRRYWRFPDRHPGRCAVAHF
RWFRLRCFQLYVDASRLPRALLLL*PRRRGA*NWRASGGALSVEPLWQLPPRAFCRY*FRTGRRGNSRENRRRSDGRYPQTYDGACRI*SG*T
LRRTGAGHRRSARPGVQPDADQPAPD**RLRHADPASADLLRQRAHHQPGPPDWHRRLCSHDAGILWCDLQKPDGESS*IED*SGRREVRLQH
SR*SG*GSE*R*YPRNRPADRAGSGGSGNRQWLRPERRDPRYPFYR*TGR*ATESRRD*CGFSAVL*TEHQIWRSRPEQNLAAVV*ARGDEPS
AGALSARAGL*QCEGISPV
>NC_000913.3_cds_NP_414958.4_417_1 [gene=yajL] [protein=oxidative-stress-resistance
chaperone] [protein_id=NP_414958.4] [location=complement(443051..443641)]
MSASALVCLAPGSEETEAVTTIDLLVRGGIKVT-NCQRRQRW*PGDYLLARREAAGGCAAGRSG*WRI*RDRAAWWH*RRGVFSR*HSAG*NR
*TVPPFRAYRRGYLRRASHRAGAARYLPDW*YDRLPDAERQNSRRTMAGQARRLGCTGKIADQPGAGYSYRLWSENYRPVGWA*KSP*SGITT
GDGGRDL*LLRV

>NC_000913.3_cds_NP_414959.1_418_1 [gene=panE] [protein=2-dehydropantoate reductase, NADPH-specific] [protein_id=NP_414959.1] [location=complement(443604..444515)]
MKITVLGCGALGQLWLTALCKQGHEVQGWLRVP-TTLL*RESG*DRWFDI*RIADRQRSRFSRHQRSAPGDAESMAGFRCRQKPRVHTACNYA
NTVNSQRHGHHRRVAKHSAAITDGHHHPCSPPRRQCHYSCGKRYHAYWPGTATGRGLQLSGGYFANRVA*RCLA*QYSRRAVAQAGSQLRD*S
TDCHLELPER*ITSSSARNYADMRRSRGGDRTRRASYFSRRFA*LRDAGD*CHSGKYLVDVAGYPRAAPH*NRLYQWFSLTPRPRAWDCRTGK
HPPV*NGKKKGE*I*AHRHWFASPLV
>NC_000913.3_cds_NP_414960.2_419_1 [gene=yajQ] [protein=phage Phi6 host factor, ATP/GTP binding protein] [protein_id=NP_414960.2] [location=444683..445174]
MPSFDIVSEVDLQEARNAVDNASREVESRFDFR-KR*SLI*AERRQQNHQSVERVRFPGQSVAGYSACQAAEARH*RQFAGCTGEYRS*R*NL
VCGSETETGH*ERDPEENRQDDQRQQTESAGANSGR*NPRNGQIS**FAGCHGDGTWWRSRSAVPVQKLPRL
>NC_000913.3_cds_NP_414961.4_420_1 [gene=yajR] [protein=putative transporter] [protein_id=NP_414961.4] [location=complement(445302..446666)]
MNDYKMTPGERRATWGLGTVFSLRMLGMFMVLP-SSDHVRHGSARCQRSINRYCHWYLWSDSGRFSDSVWPAFRPHWSQTINCRWAGGVCRR*
RYRCAFRLHLGNYSGPGATRLWSNCRRRYGAAFRSHARTKPHQSDGVYRRELWHYLCHCDGAWPDHHSQTWAARAVLDDRYSGNDRHCVDHLG
CAQQ*HSRT*S*VRNGERQFQ*SAGGTAAAETQLWHYVSAYFADVDVCCPARTTG*CRVPGG*TLEGLSGDNANRLWLGRAFHYLR*S*AQNE
ASLCLLRRVDRGCGNCVVERANAVLATGGRRAAFLCGV*FDGSPPALTYQ*RVASRLQRYGDGCLLHQPVSWRGDWRFAGRLD*RHV*RSGGI
SRWRNAGRSVADSRQYHERTAVCQQFAH*NPGEHCRKRGVKSAFARN*RHQRSVDCRRRTFSLCENRQQSDESL*DRTGNSSGI
>NC_000913.3_cds_NP_414962.1_421_1 [gene=cyoE] [protein=protoheme IX farnesyltransferase] [protein_id=NP_414962.1] [location=complement(446815..447705)]
MMFKQYLQVTKPGIIFGNLISVIGGFLLASKGS-N*LSPVYLHAGWGVTGCGVGLCV*QLHRQGYRQKDGKDEESGAGERPDLSCCLAGVRHV
AGYCWLYAAVVWRESAGLLAGGDGLCGLCRRL*PVHETPLCLRHVDWFALRRCAAGDRLLCGNR*VR*RRSDPAGYLQPVADASLLCHRHFPL
*GLPGGKHSGIASGKRHFGGEESHHAVYHRLCRCHADALSWRLRWV*ISGGRRGG*RLVVRYGSARL*SC**QNLGAQAVRLLYHRHHCPLGD
DVR*FYGTGLAYAAGCCVV
>NC_000913.3_cds_NP_414963.1_422_1 [gene=cyoD] [protein=cytochrome o ubiquinol oxidase subunit IV] [protein_id=NP_414963.1] [location=complement(447717..448046)]
MSHSTDHSGASHGSVKTYMTGFILSIILTVIPF-MDGDDRSCLSGRNSGNNPGNGSGTGSGASGVLPAHEYQIR*RLEHDGVCLHRANHRYPG
CRLHLDYVEPQLQHDDAL
>NC_000913.3_cds_NP_414964.1_423_1 [gene=cyoC] [protein=cytochrome o ubiquinol oxidase subunit III] [protein_id=NP_414964.1] [location=complement(448046..448660)]
MATDTLTHATAHAHEHGHHDAGGTKIFGFWIYL-NERLHSVLYLVCYLCRSGERHRRRPDR*GHFRTAVRSG*NFLAVVQLHHLRHGGYRHVQ
KQQKPGYLLAGVDLVVWCRIYRDGNL*IPSPDC*RHGSGSQRLPVSVLCAGRHARSARHFWSYLDGGADGANRPSRPDQH*PYPHHVPEPVLA
LPGCGLDLCVHCCLSDGGDV
>NC_000913.3_cds_NP_414965.1_424_1 [gene=cyoB] [protein=cytochrome o ubiquinol oxidase subunit I] [protein_id=NP_414965.1] [location=complement(448650..450641)]
MFGKLSLDAVPFHEPIVMVTIAGIILGGLALVG-TDHLLR*VDLPVERVADLRRP*TPRYHVYHRGDCDVAAWFC*RHYDA*PAGSCLGGRSG
LPATSPLRSDLYRARRDYDLLRGDAFRYRSDEPGGSAADRRA*RCVPVPQQLKLLVYRCWCDSG*RFSRRGRICADRLAGLSTAIGNRVQSGS
RCRLLDMESPAIRYRYDAYRYQLLRYHSEDARTGHDHVQDASIYLGITVRERTDYCFLPNSDGYRRVVDPGSLSGHPFLYQRYGWQHDDVHQP
DLGLGPPGSLHPDPACFRCVLRNCGNLLA*TSVWLYLAGMGNRLYHRAVVHRLAAPLLYDGCGRERKRLLWYHHNDYRHPDRGEDLQLAVHHV
SGPHRVPFCDAVDHRFYRHLLGGRDDWRAAGRTGRGLRSA*QPVPDCALP*RDHRRRGLRLLRRDDLLVA*SVRFQTERNLG*TRVLVLDHRL
LRCLYATVCAGLHGHDPSFEPAD*PAVPHHADDCSQRCSTDCAGYSLPRYSDVRFYSRPRPEP*PDWRPVGWPYAGVGNLFPASVL*LCRSAA
RSRT*CILGNERERRSV*KA*PL*RNSYAEKQRCRYRHCSFLHHLRFRHDLAYLVAGDCWLRRHDHHLDREKLRRGRGLLRAGGRNRKTGKPA
FR*DY*GRAEKWQL
>NC_000913.3_cds_NP_414966.1_425_1 [gene=cyoA] [protein=cytochrome o ubiquinol oxidase subunit II] [protein_id=NP_414966.1] [location=complement(450663..451610)]
MRLRKYNKSLGWLSLFAGTVLLSGCNSALLDPK-RTDWSGATFTDTDGIWPDVDCRYSRNLDGCWFRLEVPCEQ*RC*VQPELVTLQ*SGSCG
LDGTYLNHHLPCSTDLENHSRS*A*QAAGTRREAHYHRSGFHGLEMVLHLPGTGHCYRE*NRFPGEHSGVLQSDLQLRDELLLHSASG*PDLC
HGRYADSPASDRQRTRHL*RYLRQLQRPGLLRHEVQSYCNTGSRRIRPVGRKSEAVAEHHV*HGCVRKTGRA*RIQPGGIFLQRETRLVCRCN
*QVYGSR*EHGHDPARR*AQRTRRYGRHGHEPRGIRPL
>NC_000913.3_cds_NP_414967.1_426_1 [gene=ampG] [protein=muropeptide transporter] [protein_id=NP_414967.1] [location=complement(452070..453545)]
MSSQYLRIFQQPRSAILLILGFASGLPLALTSG-NLTGLDDGREYRSQNHWFLLSGRPGLRF*IPLVTADGPLHASIFWAAARLAARHANPVI
SRHCGDGFSRTRHPTPLDGGAGSGDRFLLCFPGYSLRCVENRCASGRRTWCGRGNQRAGLPFRDAGFRRPSPVAGR*MAGLAGHVLVNGGTVD
PLYYRDVACTRTNRHHSCAQNAGTSGCCTSARFLWSQ*CLAYFASYRAV*AGRRIRHEPDNHVFDSRRRV*CG*SRRG*QNAWLTSDHCWRIV
RWDFDAAPVTVPGTADFRHFTRCV*RRLLAAVDY**ASLQHGRSRLFRKPLWRDGHISLCRAVNDAM**VIFRYSICPALSAFCCRASLCRPR
GGLVC*STRLVDILSILRRRCRTRAYFAAGLPPDA*IYTSK*QLYLPYRISGRLCLCHVDTGGGRQPVGRVVTAIDDGRAGFDALLFPACSAG
SRGFSRPFWRRAWWFAGLSGATKNASDV
>NC_000913.3_cds_NP_414968.4_427_1 [gene=yajG] [protein=putative lipoprotein] [protein_id=NP_414968.4] [location=complement(453589..454167)]
MFKKILFPLVALFMLAGCAKPPTTIEVSPTITL-TTAGSKPDGRHRKH*WCRSAYRSGAGKSHPR*SNRYPDRLPRSAFPAARSAGKTDDRAW
LHGWSEWPG*SANHR*PTVC*RVPGQRALQHRDQSGYRHHRYRAEWQQNDQKLSCQLQR*RCVPGLQQKYRRCG*QCADRYHR*YVSGHQHPR
IHQAERAL
>NC_000913.3_cds_NP_414969.4_428_1 [gene=bolA] [protein=stationary-phase morphogene, transcriptional repressor for mreB; also regulator for dacA, dacC, and ampC] [protein_id=NP_414969.4] [location=454472..454789]
MMIRERIEEKLRAAFQPVFLEVVDESYRHNVPA-RL*KPF*SCAGQRSFYG*TFSESSSNDLQYFSGGTLYYRSCAGSAYLHY*GVGRVAGHR
LCLSSLSWSRKHRV
>NC_000913.3_cds_NP_414970.1_429_1 [gene=tig] [protein=peptidyl-prolyl cis/trans isomerase (trigger factor)] [protein_id=NP_414970.1] [location=455133..456431]

MQVSVETTQGLGRRVTITIAADSIETAVKSELV-KRCEKSTY*RLPQRQSANEYRCSALWRVCTPGRSG*PDEP*LH*RHH*RKNQSGWRTDL
CSGRIQAG*RLHLLCRV*SLSGS*TAGSGSDRS*KTDR*SDRR*R*RHAGYSA*TAGDLERKRRC*SRRPRNHRLHRFCRRRRVRRR*SV*F
RTGDGPGSYDPGL*RRYQRPQSWRRVHHRRDLPGRIPRRKPER*SSEIRYQPEES*RA*TAGTDCRIHQTFRR*RWFRRRSAR*SA*KHGARA
EERHP*PR*VSGDRRSGKS*RHRRTGCADRQRNRRSASPGCTAFRWQRKTSSGTAARTVRRTG*TPRSCWPAAGRSYPHQRAES*RRARERPD
RRDGFCVRRSERSYRVLQQKQRTDGQHAQCCSGRTGC*SCTGESESD*KRNHFQRADEPAGV
>NC_000913.3_cds_NP_414971.1_430_1 [gene=clpP] [protein=proteolytic subunit of ClpA-ClpP and ClpX-ClpP ATP-dependent serine proteases] [protein_id=NP_414971.1] [location=456677..457300]
MSYSGERDNFAPHMALVPMVIEQTSRGERSFDI-IFSST*GTRHFSDWPG*RPHG*PDCGADAVPGSGKPRKRYLSVH*LPRRGDHCRDVYL*
HHAVYQA*CQHHLYGPGGLDGRFLADRRGKR*TFLPAEFARDDSPTVGRLPGPGDRYRNSCP*NSES*RAHE*TYGASYGSIIRTD*T*YRAR
SLPFRP*SGGIRSGRFDSDPS*L
>NC_000913.3_cds_NP_414972.1_431_1 [gene=clpX] [protein=ATPase and specificity subunit of ClpX-ClpP ATP-dependent serine protease] [protein_id=NP_414972.1] [location=457426..458700]
MTDKRKDGSGKLLYCSFCGKSQHEVRKLIAGPS-SVYLRRMC*FM*RHHSRRD*RSCTAS*TQCATDAA*NSQPPGRLRYRPGTGEKSAGGRG
IQPLQTSAQRRYQQWRRVGQK*HSADRSDRFR*NAAG*NAGAPAGCSVHHGRRDYTDRSRLCG*RR*KHHSEAVAEMRLRCPESTAWYCLHR*
NRQDFS*VRQPVHYPRRFR*RRTAGTVETDRRYGSCCSTARWA*TSAAGILAG*YL*DPVYLWRCVCRSG*SDFPPCRNRLRHWFWRDGKSEV
RQSKRRRAAGAG*TGRSDQVWSYP*VYWSSAGCRNVE*TERRSSDSDPQRAEKRPDQAVSGAV*SGRRGSGIP*RGAGCYR*ESDGA*NRCPW
PAFHRRSRTARYHVRSAVHGRRRKSGYRRVGN*WSKQTVADLWQAGSATGIW*I
>NC_000913.3_cds_NP_414973.1_432_1 [gene=lon] [protein=DNA-binding ATP-dependent protease La] [protein_id=NP_414973.1] [location=458888..461242]
MNPERSERIEIPVLPLRDVVVYPHMVIPLFVGR-RKIYPLSGSGDGP**KNYAGRAERSFNG*AGCKRSFHRRDRGLYIADAETA*RHRQSAG
RGVTARAYFCAL*QWRTLFCEGGVSGVADH**AGTGSAGAYCNQPVRRLHQAEQKNPTRSADVAE*HRRSGASGGYHCCTYAAETG*QTVCSG
DVRR*RTSGISDGNDGIGNRSAAG*ETHSQPR*KADGEIPA*VLSERANESYSERTR*NGRRAGRKRSPEAQNRRGEDAERGKRESGSRVAEA
ENDVSDVGRSDRSAWLYRLDGTGAVECA*QGQKRPASGAGNP*YRPLWSGARERSNP*VSCGSKPCQQNQGTDPLPGRAAGGR*NLSWSVHCQ
SHRA*ICPYGAGRRA**SGNPWSPPYLHRFYAG*TDPENGESGREKPAVPAR*DRQNVF*HAWRSGLCTA*SAGSRAERSVQRPLPGSGLRSQ
RRDVCRDVELHEHSGTAAGSYGSDSPLRLYRR*KTEHRQTSPAAEAD*T*CTEKR*ADRRR*RHYRHYSLLHP*GGRAWSGA*NLQTVSQSG*
AVTAR*VIKTYRN*RR*PA*LSRCSAFRLWSRG*RKPCRSGNRSGVDGSGR*LADH*NRMCSG*RQTDLYRFARRSDAGVHSGGVNGGSCACG
KTGDQP*FLRKT*HPRPRTGRCDAERWSECRYCYVHRAGFLPDR*PGSCRCGNDR*DHSAWSGTADRWFERKTPGSASRRD*NSANSVRK*TR
SGRDS*QRNCRSGHSSCEAH*GSSDSGAAK*TVWYAGCDCKI
>NC_000913.3_cds_NP_414974.1_433_1 [gene=hupB] [protein=HU, DNA-binding transcriptional regulator, beta subunit] [protein_id=NP_414974.1] [location=461451..461723]
VNKSQLIDKIAAGADISKAAAGRALDAIIASVT-RISERRG*CSTGRFWYFCR*RACCPYWPQPADR*RDHHRCC*STELPCR*STERRGKL
>NC_000913.3_cds_NP_414975.1_434_1 [gene=ppiD] [protein=periplasmic folding chaperone, has an inactive PPIase domain] [protein_id=NP_414975.1] [location=461915..463786]
MMDSLRTAANSLVLKIIFGIIIVSFILTGVSGY-TDWRRQ*LRRKSE*PGNQPWAIRERLQQRA*SHAATAGRSILRAGSERRLYENPASTGA
ESSDRRGAAGSVRT*AETGYQR*AG*TGDFRDPSLPG*RQI**QPL*RYPQPDGDDRRSVRPGAA*PAHYPTAD*RRCRYRFYAER*NRRAGG
TGRATTRGA*GDYRC*RAGGEAACDRTGDCQLLRTKQKQFHDAGTIPRELHQAGCRNDAATG*RCGYPELLRPASGSIHPAAAYPLQHHPDQN
*R*SESGT**AE*RR*FCCISQRKICRYYLCS*RRRYGLVRRCHYPGRTEKCWSERKRPTVWCHQIFGRFPDCTSGRHSASESEIVRRST*RH
CGESETRKSPRCVLRAAAESERCGKQRHRVSGRCRASCRR*SHSDGLVQQR*PAGRVELQAGCRRYL*RRSGR*KRRAGHQL*HHHRRRRPRI
RAAHQRAQTGSGETVGRCSGTS*GIGSAQQS*TTGESGC*ETAG*FESRQRCGSYAGCRSEIWRAENLKPFRS*PD*PGGVCTATASERQTEL
RYGDRYAR*CGSAGAG*SETRFNAGRSEKSDGAGYHPEQRTNRL*SSDE*PA*RGENQNWRCAGTAI
>NC_000913.3_cds_NP_414976.1_435_1 [gene=ybaV] [protein=putative competence-suppressing periplasmic helix-hairpin-helix DNA-binding protein] [protein_id=NP_414976.1] [location=463937..464308]
MKHGIKALLITLSLACAGMSHSALAAASVAKPT-SGRNQSGSSCSTK*SSSTGESQ*RRRHPGQH**CQRGRASPRDEWRWPEESAGDCQLSR
RVRSV*NCGGSKAGAGDGQFAGGT*SGGINPV
>NC_000913.3_cds_NP_414977.1_436_1 [gene=fadM] [protein=long-chain acyl-CoA thioesterase III] [protein_id=NP_414977.1] [location=464402..464800]
MQTQIKVRGYHLDVYQHVNNARYLEFLEEARWD-RVGK*RQFSVDDGP*HRLRRGQYQY*LSSPSGIK*PVNYYQSVAAIKR*KRHLKPGHYT
GAGRAGGSGCAYYVCLY*S*NAESISSGRGIARKAGADG*V
>NC_000913.3_cds_NP_414978.1_437_1 [gene=queC] [protein=7-cyano-7-deazaguanine (preQ0) synthase; queuosine biosynthesis] [protein_id=NP_414978.1] [location=complement(464852..465547)]
MKRAVVVFSGGQDSTTCLVQALQQYDEVHCVTF-RLRSAASRRNRRGTRTGAETGGTRA*GAGCHPAQRAGGQ*PDA*QHSGA*L*T*SRWYP
EYVCPRA*YFVPDAGGNICVSGKSRSRNYWRLRNGFLRLPGLPR*VCESTKPCRQFGHGERYSF*NAADVD**SGNLGAGRLLRQTGFSP*RN
VDLL*RL*RRRLRSLCGM*FTRQRFESLSGR*TDGDGSDEAENRVEV
>NC_000913.3_cds_NP_414979.1_438_1 [gene=ybaE] [protein=putative ABC transporter periplasmic binding protein] [protein_id=NP_414979.1] [location=complement(465612..467312)]
MRLLNRLNQYQRLWQPSAGKPQTVTVSELAERC-ILQRTPCSYAVASGTGGGMAGVAGAVRTRKARTITLSGHAGIATQCDDGTGTGNRKAAR
CAGAGATGPR*AAHSVTAVYGRTMAKRYTHVAYSLLSPARTATTRLFARPCRAASRRADIFRPDPLR**YSAPDWRFSASLGNLY*RVTLGLL
SSFNPTLA*RRCSKSLTLTPAIIDAVTTASTGSIIY*REAY*SHPSAVSDLLFTSP*LLACAPAGELLQPSGASAIPTDRHGSFSLNTIHSRA
GAPGKP*LLPFTSSAA*SG*VLDNSAAFRKRFGNQLSASRANHHRQTGGAATGQPGQ*RHQFRFLLFDVAQKSPTLPLAGAKSDLHYSSIRFI
TNVRSRRKPDHRQSCITARLDYSALAGTG*SQTTENLDAGLSPTDRTSYHGRTPTGDTGSGRL*THNYFS*RKKLGRHDPTGTRRPHDGRQIN
WRSTGIYSGAMAALRSAVATCFRRSSIRTSTIDTGCRANNA**RKPI*CPESGF*PVNDRCDADAAVQLSLSH*CPSRRERCATDTARLV*IY
RSLASRAIAM
>NC_000913.3_cds_NP_414980.2_439_1 [gene=cof] [protein=thiamine pyrimidine pyrophosphate hydrolase; HMP-PP phosphatase] [protein_id=NP_414980.2] [location=467412..468230]

MARLAAFDMDGTLLMPDHHLGEKTLSTLARLRE-TRHYPHFCHGASCAGDAAYSRGAIAGCVFDYRQRNARAFSGR*TFTS**FTCGCRGAGA
VSAMGYPSQHAYLQ*RRLVYRERDPCVVAGICL*RFSLSDNRCQKNATRQRHQDLLLWRSRRSYTLADPAIRSIRRACTFVFFRHGLPRSAAG
GLQ*RRCIDGADPTFRFIVARLHGLW*CDERSRNVSQRR*RIYYGQCDAATARGAPAFTGDWTLPKSGCLSLFDALAGLSTSTLFPRI
>NC_000913.3_cds_NP_414981.4_440_1 [gene=ybaO] [protein=putative DNA-binding transcriptional
regulator] [protein_id=NP_414981.4] [location=468383..468841]
MLDKIDRKLLALLQQDCTLSLQALAEAVNLTTT-TLLEAPETAGGRRYPYRQSRPAGSGKNRPRPDRFCADKNATSQQRMVLPLCHGGYRNAR
SAGVLAHGW*IRLSDARPGCRHETLRRVL*ASGKQRAGAVGRHFQLRDGTD*IHHFFTHRI
>NC_000913.3_cds_NP_414982.1_441_1 [gene=mdlA] [protein=putative multidrug ABC transporter
ATPase] [protein_id=NP_414982.1] [location=468871..470643]
VRLFAQLSWYFRREWRRYLGAVALLVIIAMLQL-SSAKSGWYCCRWRDRTTLYYRADPDVDRHHGADCRCGLSPALRLAGIAVWCVLSTGC*T
A*RLLPSAKPAAS*VLPASSHR*PHGSCDQ*RRSRRVCRRRRGADAGGFTGDGLRCVDYDVYAN*LAVDLIFPVADASDGDHD*AQRRCFA*T
L*AGTGGVFQS**PHPGKPHQYPHDQSLWSGRSPVGVICRGCRRYRQKKHAGGAY*CSFRPDHLYRDWYGELAGDWRR*LDGGAGQFNAGPAH
QFYDVFRSDDLANAGAGMDV*HCGTW*CCVQPYSRDAGGSAGGERW*RTRAGRAWRTGCKYSPVHVSAD*PSCAGKRQFRPETRSDAGYLRAD
WFRQKYPVVAHSASFRRQRGGYSLS*YSSDEVTTR*LA*PPGGS*PDAIPFF*HCGE*HRAGLPECHPARD*ACRAVSQRT*RYFASTARLRY
RGGRARCDAFRRAKTAYLHCSCVISQRGNPHP**CAFGGGRTH*APDPA*PASVGAGKNGNHQCPSPFCTDGSQ*NYCDAARTYRPAWQS*CA
GTTKRLVSRYVSLSTTGGGARRRSGKSRGGRRCV
>NC_000913.3_cds_NP_414983.1_442_1 [gene=mdlB] [protein=putative multidrug ABC transporter
ATPase] [protein_id=NP_414983.1] [location=470636..472417]
MRSFSQLWPTLKRLLAYGSPWRKPLGIAVLMMW-SCGGGRSQWAAAYQLFYRQYGSEK*PAVESGCRAGCGVCWAATVCRRATLRAVAAV*SG
GSRRSATVAYRRDGCCVTPAIKRV*YPTRRAGDFPRH**H*SDPRSLRYRSGNCPAQCRAGGRDAGGDVQPRLANGTGGDNDFPGGAGGNGDI
PALQHADCPSCARLFGGYQRRL*RNHQWHERYPAVSSAGAIWRTYGGGQSFTLYGEDANPAPRRFSAASAAESVFIAHSLWLVDAVWLLRQRH
H*SGRAVCVYQLSWAT*RTINRTDHATGDAATGCCCW*ARV*TDGRTAPAIWQ**SPVTEWHHRSR*RVICLSR*QSGAKEH*SLCAFAQFCG
AGRAYRQWQKHPRQFIDGLLPANGR*DSP*WSSIKFAKSQRAAPGRGNGAARSGGAGGYLPRQRDAGAGYLRRTRLAGAGNRATGGAGA*HER
RYLHAAGRAGE*SLSWAKATAGTGARAGRDAANPDP**GNRQH*LRY*TGDSTCSGGGA*TYHAGSDCSPLIDHC*CRHHSGASSWASRGAGH
SPATAGGPGTLLADVSTATCGRRAGSQRA*RGIIERL
>NC_000913.3_cds_NP_414984.1_443_1 [gene=glnK] [protein=nitrogen assimilation regulatory
protein for GlnL, GlnE, and AmtB] [protein_id=NP_414984.1] [location=472598..472936]
MKLVTVIIKPFKLEDVREALSSIGIQGLTVTEV-KRFRASERACRAVPGGGIQRQFPAKSKN*CGDC**PTR*SDRYRQ*GGLHRKNWRRQNL
RR*IATRHSYSYRRSRRSGAV
>NC_000913.3_cds_NP_414985.1_444_1 [gene=amtB] [protein=ammonium transporter]
[protein_id=NP_414985.1] [location=472966..474252]
MKIATIKTGLASLAMLPGLVMAAPAVADKADNA-IYDDLYCAGAVYDYSGDCPVLRWVDSRQKRAVDADAGDGDICTGLYSLGGLRLLAGVW*
GQQLLRQH*LVDAEKHRTDGGDGQHLSVYPRGVSGIVCLHYRRLDSWGAGGTNPLLSCVDFRGGMADALLHSDCAYGVGRWFAGFSRCAGFRG
WHRGAH*RRNRRSGGRVSDRKTRGLR*RGV*TAQPADGLHRDCHSLYRLVWL*RRVSGHGE*NRGTGICEYCGRNGGGNSWLDLR*MGAAW*A
FTAGGVFWRDCRSGRRDASLRLHWGWRRVDYRRGSWSGGLVGRYHAQTLAAGG*SLRCLRCARRLWHCRLYHDRDFCRQLAGRRGLR*RCDDG
PSVAGTAGKHRHYDRLVRCCGIYRLQIGGSDGWSACTGRAGARRAGCQQPRRECL*RV
>NC_000913.3_cds_NP_414986.1_445_1 [gene=tesB] [protein=acyl-CoA thioesterase 2]
[protein_id=NP_414986.1] [location=complement(474301..475161)]
MSQALKNLLTLLNLEKIEEGLFRGQSEDLGLRQ-SVWRPGRGSGLVCCKRDRP*RAAGTFVSQLLSSPWR**EADYL*CRNAA*R*QLQRPPG
CCYSKRQTDFLYDCLFPGTRSGFRTSKNNAVRASA*WPPFGNANRPIAGAPAAASAER*IHLRSSAGSPSGGVS*PTERSRRRTTSSGVDPRK
W*RAG*PARSSVSARLRF*S*LPAGSSTAARHRFSRTGDSDCHH*PFHVVPSPV*FE*MAAV*RGEHLGVQRTWLCAR*VLYPRRRTGCLDRS
GRGDA*SQL
>NC_000913.3_cds_NP_414987.3_446_1 [gene=ybaY] [protein=outer membrane lipoprotein]
[protein_id=NP_414987.3] [location=475379..475951]
MKLVHMASGLAVAIALAACADKSADIQTPAPAA-KYVYFSNTTTSYPATECLRYRLDPSESRTAA*CCADRDTF*RVVSRCTVKSAGAESGAY
*R*TVTIQLCSVI*PGRCSAERAYSVECGDYRE*QTGIYHRYRSAGDQPGRN*SRPDIGAGTANRRAGSGQRWRNDYRTFDFTNSGESVFGSS
SSYAIL
>NC_000913.3_cds_NP_414988.1_447_1 [gene=ybaZ] [protein=excision repair protein,
alkyltransferase-like protein ATL] [protein_id=NP_414988.1]
[location=complement(475982..476371)]
MLVSCAMRLHSGVFPDYAEKLPQEEKMEKEDSF-TPTRLANRRRYSRRLCHHLR*CGETGGIAPRRAPGGRCVKASP*RQHLTLAPGG*SPRH
NFANRTGFTASATGITGRRCDGIGKRANRLAALSLELL
>NC_000913.3_cds_NP_414989.1_448_1 [gene=ybaA] [protein=DUF1428 family protein]
[protein_id=NP_414989.1] [location=476672..477025]
MKYVDGFVVAVPADKKDAYREMAAKAAPLFKEF-RRASYCRMLGQRCTGWQSDRFSYGGESGRE*RGCL*LD*IPFKRGPRRC*SKDDVGPTD
ERVRRVHAV*RQANDLWRIRVNHRRI
>NC_000913.3_cds_NP_414990.2_449_1 [gene=ylaB] [protein=putative membrane-anchored cyclic-di-
GMP phosphodiesterase] [protein_id=NP_414990.2] [location=complement(477067..478617)]
VRTRHLVGLISGVLILSVLLPVGLSIWLAHQQV-RNIVY*RAGYLFLPRRYSSQ*GGDTRERCAAGAGKMARRCL*RSPSHGNASGILQLSLY
SGSGLYR*QRSPVFVSGA*KSARYLPRAR*NFERWLSCLVNIA*RFRHYPLHGRHGNGTLCRHDRPRFLY*CHSL*LMAN*CRHYWQCP*RCH
NQQR*NCSGNYYQATKNTR*AYRK*WNHLRYPALTGDEYFDHHMGFNENVAERLASASLYLVTARVGDWPAGSDVCAAYFAPYSVTASSAAGC
YRKS*YLRALSADCLLSQWQNCRC*GTGALAADRR*LVVTR*FYSAGTANGPF*AIDATDYKKRL*RYGRLAASASTAAYFDQS*IPRAHLGK
NPAIAA*HDQSLSG*SQTDRA*TH*TRVCRSENQRPDNFSLPGGGP*NLS**FWYGVFKFKLFTGSGCRHSEDR*IFR*CAGI*KCHAAYHRN
GKNTETENGSGGNRNQ*TRRVVTPAWRALRSGLALQQGITERRFLTLGRATFV
>NC_000913.3_cds_NP_414991.4_450_1 [gene=ylaC] [protein=DUF1449 family inner membrane
protein] [protein_id=NP_414991.4] [location=complement(478781..479251)]
MTEIQRLLTETIESLNTREKRDNKPRFSISFIR-KTSGAVYRYVRCFFCHPGGDVAVRNAVRLCLATGCIIYPA*WFLLFRCLPTLPL*RYRR
AGFPRLL*RRMVQHALCTCRAG*SHLELSACRGCS*GTTAKNDRP*R*TVFLRYFYPRSRRINIL

>NC_000913.3_cds_NP_414992.1_451_1 [gene=maa] [protein=maltose O-acetyltransferase] [protein_id=NP_414992.1] [location=complement(479367..479918)]
MSTEKEKMIAGELYRSADETLSRDRLRARQLIH-TIQSFPGGRAHITPANSR*SIRSGDRGLY*ANVSL*LWL*HFSR**FFRQLRLRDA*CL
PYSHR**LYVGTRRSYLHGNTSHRPCST**RC*TGETRHHR**RLDWRTRGH*PWCDHW**RRGSLRCSCHKRCPGQRCRGR*SSQNN*KIV
>NC_000913.3_cds_NP_414993.1_452_1 [gene=hha] [protein=modulator of gene expression, with H-NS] [protein_id=NP_414993.1] [location=complement(480090..480308)]
MSEKPLTKTDYLMRLRRCQTIDTLERVIEKNKY-RIIR**TGGILLSRRSPPRRIDHE*TVRQDPFLSMEIYSL
>NC_000913.3_cds_NP_414994.1_453_1 [gene=tomB] [protein=Hha toxicity attenuator; conjugation-related protein] [protein_id=NP_414994.1] [location=complement(480334..480708)]
MDEYSPKRHDIAQLKFLCETLYHDCLANLEESN-TWLGKRPNLGDQPPVE*TD*AYCDLRT*LQN*V**RQ*AH*ADRRISG*HLYVVQ*LWY
*YAGSSEMAEVR*STIPLFCQCDERESCEFILL
>NC_000913.3_cds_NP_414995.1_454_1 [gene=acrB] [protein=multidrug efflux system protein] [protein_id=NP_414995.1] [location=complement(481254..484403)]
MPNFFIDRPIFAWVIAIIIMLAGGLAILKLPVA-TISYDCTAGSNDLRLLPRR*CENSAGHGDTGYRTEYERYR*PDVHVL*Q*LHGYRADHP
DL*VWY*CGYRAGSGAEQTAAGDAVAAARSSAARGER*EIIQQLPDGCRRYQHRWHHDAGGYLRLRGGEYERCHQPYVGRG*CSVVRFTVRDA
YLDEPE*AEQIPANAG*CHYRHQSAERPGCGGSARWYAAGERPTA*RLYYCSDASDLY*RVRQNPAESESGWFPRAAA*RRED*AGW*ELRHH
RRV*RPTGFRSGDQAGDRCKRAGYRCGNPC*TGEDGTVLPVGSENCLPIRHHAVRENLYSRSG*NAGRSDHPRVPGYVSVPAELPRDVDSDHC
RTGGIARDLCRPCRLWLLDKHANNVRDGARHRPVGG*RHRCGRKR*ACYGGRRFAAKRSYP*VDGADSGRSGRYRDGTVGGIRTDGLLWRFYW
CYLSSVLYYHCFSNGAVGTGGVDPDSSSLCHHAETDCQRRSRGR*KRLLRLV*PHVREEHAPLHRQRRRYSAQYGALPGAVSDHRGRHGLSVR
ASAKLLLAR*GPGRVYDHGSAASRCNAGTYTESAQ*GNALLSDQRKEQR*VGVRR*RLRLCGTWSEYRYCVRFLEGLGRSSGRRKQS*SDYHA
CNTRFLANQRCDGFRL*PARNRGTGYCNRL*L*AD*PGWPWSRKTDSGA*PVACRSSEAP*YVDQRTSKRSGRYPAV*D*YRPGKSAGAGCFY
QRH*HHSGRCMGRQLCERLYRPRSCEESLCHVRSEIPYAAG*YRRLVCSCC*WSDGAILGVLLFSLGVRFAASGTLQRPAIHGNLRPGGTG*K
YR*SNGADGTTGEQTAYRCWL*LDGDVLSGTSLRQPGTFTVRDFVDCRVPVSGGAVRELVDSVLRYAGRSAGGYRCVAGCHLPWPDQ*RLLPG
RPAHNHWVVGEERDPYRRIRQRLDG*RR*RSD*SDA*CGADAFTSDPDDLAGVYPRRYAAGYQYWCWFRRAERSRYRCNGRDGDRNGTGNLLR
SGILCGGSPPL*PQE*RYRAQPYCRSSL
>NC_000913.3_cds_NP_414996.1_455_1 [gene=acrA] [protein=multidrug efflux system] [protein_id=NP_414996.1] [location=complement(484426..485619)]
MNKNRGFTPLAVVLMLSGSLALTGCDDKQAQQG-RPADARRWRSNSQN*TSADHNRASGSHQCLPDRRSSSSS*RDYPEA*FQRR*RHRSRCL
SLSD*SCDLSGDIRQCER*SGESPGCSQYRAIDGESLSETARYSVHQ*ARVRSGSG*CATGECCGNCGESCR*NCADQSGLHQSHLSD*RSHW
*VERDGRRIGTERSGDCAGNRAAT*SDLR*CDPVQQRLPAPETGTGEWHAETRERQSQSVTDHQ*RH*VPAGRYAGIL*RYR*SDHWVYHPTR
YLPEPGSHSAAGYVRARTSGRRA*SKRYFSPATGRNPYAAWRCHRTGSWRG*QSGNPSDRCKPGYWR*VAGDRRSESRRSRSNKWAAESASWC
PGKSTRSYR***PASRKRCSA*TVQVL
>NC_000913.3_cds_NP_414997.1_456_1 [gene=acrR] [protein=transcriptional repressor] [protein_id=NP_414997.1] [location=485761..486408]
MARKTKQEAQETRQHILDVALRLFSQQGVSSTS-TGRDCKSSWRYARCNLLAF*RQVGFVQ*DLGTVRIQYW*TRA*VSGKIPWRSTLSIKRD
INSCS*IHGDRRTASIIDGDYIPQMRICRRNGCCATGTT*SLSGKL*PYRTNVKTLY*SENVACGFNDASRSNYYARLYFRPDGKLALCPAIF
*S*KRSPRLRCHLTGDVSPVPHAS*SCH*RI
>NC_000913.3_cds_NP_414998.1_457_1 [gene=mscK] [protein=mechanosensitive channel protein, intermediate conductance, K+ regulated] [protein_id=NP_414998.1] [location=486536..489898]
MTMFQYYKRSRHFVFSAFIAFVFVLLCQNTAFA-TGVIEW*SADKSGPAGAT*LTK*TKRSFCSGQTGAAGSDRYISHPR*NRSHKRRDSSAT
AKSR*SAGKNAPGDRGVNST*RCR*RRNAQNSEHAVVAPAGNSRCPGAGRFAKRTKRSGVL*QPAGFVTDAARTRAKCDV*RFAAAATNSQS
SGWD*CRRDSLTSQPESVNAGPAGVAECGD*PAA*KPGREHRLAGYLAKAT*LRDGEQRSSGAPVTTVARSGKQQAPDFNRKNGAGSRLPG*S
RAYSG*SAGEAGTGN*PAVKSASDYRD*KR*SVDAAKH*SQKLAGAGAAIGTQY*RADCRPEGQPAVVSYPLPATTNAALGG*TGKHDQPHRG
FASRTV*S*PAA*CTLPERCVRQQTGRRSHQRSQQRSSRCVIASG*YASRIAGSTQQTVG*PADDGH*PANQPAAVNECVEKPEIHPDSANLL
GEQ*PSNGLGLDQSVPAKPER*I*VDENHGELAKSLARRFYRFPRWFAAAVDCRADPLASGLAESVSTKTGFRCGFPA*RQPAQHTKSDPYRP
DPCAAGVPDYSRGWPDSVDHAAQHQRTAMVVQQKTGDILAGVWPVLEGTGEKRRCRTSLRHAGTADQPLASANCPHQSRIAAYPFLVCGGRTF
PAASDG*CAGASDDFLQPAADCLPGMADVPRKLA**RVAHHATGHHYRAVDNPDCADGADCYRLLLHYAASGRTLD*NRLSGDHLEPAVPDGT
AWLKRSGAAYRLASCAGASAESGERGRRRC*TAGRTHHCTGAS*PADAAYYHVADVCAVRCHVLGNLVRFDHRVQLSRQHHALALQRH*SWRC
GGEKRHHGQSVVCDYRLNGGLGVDSQPAWFTGSAGALATEYAPGRVVCHYYHP*LHHYCCWCDDGVRIAGRLLG*TPVAGRSIIRRSWFWFTR
NFR*LRLRFDHSIRTSGAYWRYGNHW*LLGDGK*DPYSCDNDYRFRSQRSDHPEQSVCYRASDQLVVD*HYYASGDPSRRGLWLRSGKSA*SV
TEGGD*APKGDARTNAGSLLYGIWCQHVGS*AASVCA*TA*P*SYCR*AEPYYRSAVP*KRHQHCL*PA*SASAQREGR*GDGSKTRLQRR*P
DASGRV
>NC_000913.3_cds_NP_414999.1_458_1 [gene=ybaM] [protein=DUF2496 family protein] [protein_id=NP_414999.1] [location=complement(490110..490271)]
MSLENAPDDVKLAVDLIVLLEENQIPASTVLRA-TGYCKA*L*KEINAR**GGKV
>NC_000913.3_cds_NP_415000.1_459_1 [gene=priC] [protein=primosomal replication protein N**] [protein_id=NP_415000.1] [location=complement(490285..490812)]
VKTALLLEKLEGQLATLRQRCAPVSQFATLSAR-IRQASFSDSCDNTTGLSRRGGR*SGCASSCS*AATAAASGLAGGTSGGTTGSHRA*SLR
LVIARVGGQCTTENCPLAA*TYSASGF*AAAT*DGCRTQSPSGAGDRSRGTANAAS*SGSL*SAPGTLPPCAGKNRKQVSAFNPL
>NC_000913.3_cds_NP_415001.1_460_1 [gene=ybaN] [protein=DUF454 family inner membrane protein] [protein_id=NP_415001.1] [location=490882..491259]
MQRIILIIIGWLAVVLGTLGVVLPVLPTTPFIL-TGGLVLCPFFPALSRLVAVPLMVWQLSTFLAETSCDAARRQTAGDFAYFAHVCHFSVVR
PDAMGAHHVAGNSRLFAFLYVANSGD**KARKAL
>NC_000913.3_cds_NP_415002.1_461_1 [gene=apt] [protein=adenine phosphoribosyltransferase] [protein_id=NP_415002.1] [location=491412..491963]
MTATAQQLEYLKNSIKSIQDYPKPGILFRDVTS-ITGRPESLRSQHRLAG*ALQKCGHYQSCRHRSAWLLVWRSGSSGSGRWLCTGP*TGQTA
A*NHQ*NLRPGIRHRSAGDPR*CHQTGRQSSGGGRPAGNRRHYRSDR*TDPSSGW*SG*RCVHYQPVRSRRRTASRKTGHYQLQPCPVPGPL

>NC_000913.3_cds_NP_415003.1_462_1 [gene=dnaX] [protein=DNA polymerase III/DNA elongation factor III, tau and gamma subunits] [protein_id=NP_415003.1] [location=492092..494023]
MSYQVLARKWRPQTFADVVGQEHVLTALANGLS-IRAYSSCLSFFRHPWRRKNLYRPTAGEGAKLRNRHYRDAVRRVR*LS*NRAGALCRSD*
NRRRLAHQS*RYPRPAG*RPVRSGAWSFQSLSDRRSAYAVAPQL*RTVKNP*RAAGAR*VSAGDDRSTEIAGDDFVTLSAISSQGAGCRANSP
SA*AHPQRRTYRSRAAGAAIAGTRR*RQPARCLKSDRPGDCQR*RPGFNPGGQCDAGYA*RRSGAVAG*SDGRGQRRARNGAD**SRCPWYRV
GSVAGGNARPVASYCDGTTFACCTWQRHGRHRAADA*TGAHHTADGYSALLSDAVDWSQRITVCAGPSHGR*DDAAARAGIPSAYAAA*ARSA
TTVLCTRRANGSNDANPGAAATAISAAAGTDCTAPGNHQPGAGGAPAVAARAGSNQSKKE*TGSRYPRAAGE*RCAGKTGFGHRSRSGASGAI
GAGKSASQKRSVSLEGDHSGDAAKRSGRHAEGAEKSAGT*KNAGTGGEASGRSH*ARPVGGTGEPTFATKTGRTGGVKCLERGERQRSMSAFA
LLSAAFEQPRCTAKTG*SVEHVKRFNG*TDYR*R**SRGAYAAGVASGDIRRKTCAGARVHYCG**YSDPASVLRCGAG*RKYPPHL
>NC_000913.3_cds_NP_415004.1_463_1 [gene=ybaB] [protein=DNA-binding protein, putative nucleoid-associated protein] [protein_id=NP_415004.1] [location=494076..494405]
MFGKGGLGNLMKQAQQMQEKMQKMQEEIAQLEV-NRRIWRRSGKSDHQRCTQLPSRRDRPEPAGRRQRDAGRPGGCSIQRRSTSY*RNAERKN
GLCILRNAAAAWL*DAVL
>NC_000913.3_cds_NP_415005.1_464_1 [gene=recR] [protein=gap repair protein] [protein_id=NP_415005.1] [location=494405..495010]
MQTSPLLTQLMEALRCLPGVGPKSAQRMAFTLL-TARS*RRDASGAGAHPGDVGNRPLRRLPHFHRTGSL*HLFESASSGKRSNLRGGESGGH
LRH*ADGAVFRSLFCVDGASVTAGRHRSG*YRA*SSGTASGRGKNH*SDPRHQPHG*R*SYR*LHCRALRAI*RGSQPNRSWRSGWRRAGNGR
RHHVVTLPCRAS*DSFL
>NC_000913.3_cds_NP_415006.1_465_1 [gene=htpG] [protein=protein refolding molecular co-chaperone Hsp90, Hsp70-dependent; heat-shock protein; ATPase] [protein_id=NP_415006.1] [location=495120..496994]
MKGQETRGFQSEVKQLLHLMIHSLYSNKEIFLR-RAYL*RLRCGGQAAFPCAL*PGPVRR*WRTTRSCLFR*RQAYADHLR*RRGDDPRRSD*
PSGDYR*IRYQIIPRIPGF*PGERQPADRSVWCWFLLCVYRGRQSDRAYSRGRRKTRKWRLLGIGWRR*IHRCRHHQRRSWY*NHPASA*RRR
RVPR*LARAFHHQQILRPYRAAGRDRKTRRERRRNRYLLGENQQSAGAVDS*QVGNHR*RVQRVLQTHRPRL**SADLEPQPC*R*AGVHQPA
VHPVPGSVGYVEPRS*TRPETVCSACVHHGRRRTVHAELSALRAWSD*LQRSAAERFP*NPPGQHGNA*PAQCADQACAANAGKTGERRRGKI
PDLLATVWPGTERRSGGRFR*PGSDRQTAAFCFYPYRFFCADRISGRLRFPHERRAGENLLHHRRQLCGSEEQPAPGTAA*ERHRSSAAFRPH
R*VDDELSD*VRR*TVPVGV*S*RVA*KTG*RS**ERERSGESTDSVHRPCESPARRARERCPSDSPSDRYASDRFDRRGRNEHSDGETVRCG
GPESARSEIHLRTEPGSRTGETCGRY*R*SEVQRVGRTAAGSGAAGRTRHAGRSEPVYSSYEPAAGFL
>NC_000913.3_cds_NP_415007.1_466_1 [gene=adk] [protein=adenylate kinase] [protein_id=NP_415007.1] [location=497175..497819]
MRIILLGAPGAGKGTQAQFIMEKYGIPQISTGD-NAACCGQIWLRAG*TSKRHYGCWQTGHRRTGDRAG*RAHCSGRLP*WFPVGRLPAYHSA
GRRDERSGHQC*LRSGIRRTGRTDR*PYRRSPRSCAVWSCLSR*IQSAESRRQRRRYR*RTDYP*R*SGRDRT*TSG*IPSDDSTADRLLLQR
SRSG*YQIRES*RHQAGC*SSR*SGKNPRL
>NC_000913.3_cds_NP_415008.1_467_1 [gene=hemH] [protein=ferrochelatase] [protein_id=NP_415008.1] [location=498055..499017]
MRQTKTGILLANLGTPDAPTPEAVKRYLKQFLS-RQTRG*YLTVVMVAIAARRDFAAALAACGEAVCLCLDGRWLAADGLQPPATAGAGTTFT
GDARSAGNELRLAITGKRRR*TPGRACRSYCGAAALSAILLFYGRCGMG*TGTHSGAQT*HSGDIVYT*LRR*PRLH*CTGEQRTRFFCQTWR
TGSATALLSWHSPALCR*RR*LPATLPHNDS*TGFRIGDGTGKSDDDLSVALWSGTLADALYRRNAENARRKRRRSYSGDVPGLCCGLSGDAG
RDCRAKP*GLPRCRREKI*IYSGA*CHAGTYRNDG*SCCRVSL
>NC_000913.3_cds_NP_415009.1_468_1 [gene=aes] [protein=acetyl esterase] [protein_id=NP_415009.1] [location=complement(499014..499973)]
MKPENKLPVLDLISAEMKTVVNTLQPDLPPWPA-NGNDC*ATTVLHA*APILECGRSRNGNQSLHGSNKIWAGGNTSLLSAAR*PSDAILFAW
RRFYSRQSRYPRSHHAPAGKLQPMYGDWY*LHPFT*SAFSASDRGNCGCLLLFPPAGGGLSNQYVPHWLCR*FRRCHAGARQCVVVA**TDRL
R*SCGRFAVVWALRITGFRDSSSVGRCLGWLNATGFADVRRGIFKQRRGPRVAVLLSV***SHSRSSALFYCRGGVRSAAG*QPSALPDVSGA
SAAL*VQTLPRHAARLFALFTDDENRRRGSSRRRSVLYRSAL
>NC_000913.3_cds_NP_415010.1_469_1 [gene=gsk] [protein=inosine/guanosine kinase] [protein_id=NP_415010.1] [location=500125..501429]
MKFPGKRKSKHYFPVNARDPLLQQFQPENETSA-SLGSGYRSNAGRY*SESG**IY*ALWIKRRAFTGD*G*CSRSALSGTKTEKPDYPSVCG
WHHW*HHAQLLGARGRPFGAAGRHVQQY*NWQLCLSLPV*HFQPYRS*LSTRRGWPDWSLLYADWRVRGTYLCYQSRPHEPAAG*KHSGRCDC
RSLGTGSHLISGALQAG*THAGSNHESH*VREEI*RTGGADAGHQVCHCRESAVVAAIPQRSRLYPCDERR*SRSVDRRKRSVVGI*QGAGLG
RSGAVHRRANRLVYGGLYRRRSET*NPASAAAGRYSGIQPV*V*PRHAPQGLPESAACIFAHCAVHGRAGKNHEH*WSGGWRIGSVAA*HYRQ
QLPS*QRTKLQQT*IHLVNLFIVSAGV*IC*PCELSGTEPAFTSFNARLAGA*RQPGRVLLGSL
>NC_000913.3_cds_NP_415011.1_470_1 [gene=ybaL] [protein=inner membrane putative NAD(P)-binding transporter] [protein_id=NP_415011.1] [location=complement(501562..503238)]
MHHATPLITTIVGGLVLAFILGMLANKLRISPL-SGISVSGCAGRTIHSGLCCRYQACPGTG*TGRHSVDVWRRFALFAEGFDGGKGHRHSRC
DRPDSRGDAAGYGALCRAGLVVNDRYRVRFMSFHRQYRGVTART*RTAIN*QSAWANRHRLVDCGRPGNGSDAGVAARSGRNDGTGRCGLCHS
CSRYGDHHRQSDRIYRHYDAGRSPSGAVDYGTQRGNRFSRAVYPVGAGAGVRGCLWCGRAV*CLLCTRCVLCRDGTERV*TESPCRPRYAAIA
RRVCGAVFCLRRDVV*SVNSDSATAGSAGDAGDYSVW*VVSRIFPGATVWSLPTYGINHRRQPGADW*VRVYPGGTGNGIEFTAAGRTKPGTG
RGDPVDYAQPGTVRTTGEISGEDRNAGRADAGRGNRRREADPSGYLQPCATGGLRSCRQPAGGEIARL*YSAGGD*DVTNPC**AARARGPRS
IGQCGERRNYATGASGMCKMADPDDSQRL*SG*DCGICPREKSGY*DYCPRPL*R*SGVYHRTWCESGSDGRA*NRPYHAGTAGNATGG*GGD
GV
>NC_000913.3_cds_NP_415012.1_471_1 [gene=fsr] [protein=putative fosmidomycin efflux system protein] [protein_id=NP_415012.1] [location=complement(503476..504696)]
MAMSEQPQPVAGAAASTTKARTSFGILGAISLS-TSAERHDPIADSGDLSAASVRIFSDIYADWHDNPHLPARLFATATSGRLLDR*ISDAMV
VANWHVLYLKRSGAACAGGQFWRSSAGGGAGRYRFIGLSSGIFSRGPYGFRRAAWPGAIYLSGRRQLWQFPGTLAGGGDYRALWQRQRCLVCA
CGTAGDRGVGANQPLVLGTAPNE*RKTQSDDYQSTAAQ*SCTGGQHSVNPHFLEIFLYGEHQQLLHLLSDAKIRIIYPECSASSVCLPVCRCG
RYGDRRACRG*NWAEICDLGLYPRRCAVYADFTLRQPALDGGFNGDYWIYPRFGILCHSGLRSGAASRTYRYGFWTLFRFCFWHGRSGSGSSG
AYRRSHQHRVSL*NLCFPATIGDVDHIPA**PA*RL

>NC_000913.3_cds_NP_415013.1_472_1 [gene=ushA] [protein=bifunctional UDP-sugar hydrolase/5*-nucleotidase] [protein_id=NP_415013.1] [location=504914..506566]
MKLLQRGVALALLTTFTLASETALAYEQDKTYK-NYSSAYQ*SSWAFLAQ*IWRIWSGGAKNAGGWYPQRGCG*RR*RAATFRWRH*HWRARV
*LTGCRT*FSRYESGGL*RDGDR*S*I**SAHRITPAGKVGQVPVAFREYLPEKYWRAPV*TVGAV*ASGSENCRYWADNR*HSKNW*PGILH
*YRIS*ARR*SEAGDSGAATDRKARHYYRGDPYGALR*W*ARL*RTGRCGDGTRAACRIAGDDRRWSLARSGLHGGRKQKTGRLRAGYAMQTR
STKRHLDCAGA*VGQIRGTG*F*VS*WRNENG*LPADSGEPEEESDLGRREKRARALHS*NR*KPANDLAVITVPEQRQSAAGSENRRNQWSS
GRRS*QSAFCTDQYGAVDSGSPNGSHWCRLCGDERRRNS*FYRSRRYQL*KRAESAAIRQCGGVCRHDR*RGD*LPDRRRADEARFRCLPAIC
QR*LCGERRQTERP*NQRRTGRSGENLPYGDIKLQCHRR*WISAP**QTGLCEYRLY*CRSAESVYPEKLAAGCECL*TER*GELAV
>NC_000913.3_cds_NP_415014.1_473_1 [gene=ybaK] [protein=Cys-tRNA(Pro)/Cys-tRNA(Cys) deacylase] [protein_id=NP_415014.1] [location=complement(506603..507082)]
MTPAVKLLEKNKISFQIHTYEHDPAETNFGDEV-SQKIRFESGSGLQNAAGGSER*YETPCRGRYAGRRSTGS*KSSKSAGCQES*DGRSDGR
AAFDGIPGWGD*PTGAEKTSANDYRRPRTRICHYLCFRRQARTGYRTGGRRSGKDPRCQIC*YRPPRL
>NC_000913.3_cds_NP_415015.1_474_1 [gene=ybaP] [protein=TraB family protein] [protein_id=NP_415015.1] [location=complement(507286..508080)]
MDLLYRVKTLWAALRGNHYTWPAIDITLPGNRH-ISSDWQYSYG*PRYGSPAHPFAQKAQKRRCADRRGGCFHQRYAFC*FACLRGAGRAH*R
GATPKPAAH*PGDGHFSLTLFYPTAVANRDGSSGDAGTKTGAAGRIRYRLPAIAGGEATT*TRD*TGRG*KPDCHVAPAP*QRTGAAGRYADP
LAYQRTVAATNDELVAECTAAK**YNAAQYVQSVAVRCADASAKSRLAG*ITRHAAGAICGRGRCTTPVWRREFAANVAL
>NC_000913.3_cds_NP_415016.4_475_1 [gene=ybaQ] [protein=putative DNA-binding transcriptional regulator] [protein_id=NP_415016.4] [location=508218..508559]
MKQATRKPTTPGDILLYEYLEPLDLKINELAEL-TACSS**RQCTDQ*QS*THY*DGISSGESF*YHSRFLAKPPGGG*SLGS*KQHAHPGRI
GTD*NSG*IFGTP*RACKKGRV
>NC_000913.3_cds_NP_415017.1_476_1 [gene=copA] [protein=copper transporter] [protein_id=NP_415017.1] [location=complement(508875..511379)]
MSQTIDLTLDGLSCGHCVKRVKESLEQRPDVEQ-SGCVYH*SARYRDCQCRTADRNHQTSGL*RICKPPKG*TAGGVINPVGSTDSGF*GASG
SDRR*R*QPAVAAERHELRQLCHPRTKCAAKRTGRHSGTGKPGGAYCAGDGQCLPTRFSAGGGKSGLRRGSD*R*R*TPRAPARNRRRYDEAL
PLAGNCRTGGGYPGDGLGDDRR*HDGHR*QPQPVVGYRPDNPGSDGFRRRPFLPQCMEKPAERCGDDGYAGGAGYWRGVALFDERQPVAAVVP
DGSATSLLRSQRDDYRSDQSRPYAGSARTPAFF*GAGKVTRFNPADGTPGY*RR*KKRASGRSAARYVAAPDDRRSRAGRWRDYPGRSMAG*S
DADGRTNPAAKRRRR*RPCRDSGTGRQCAVSCQCGWQPYYAVTNHSHGAPGPEQQARNRSAGG*NLSRICAGSGGYCACQCGNLVFLWSGTAD
CLYPGDCHHGTDYCLSVCAGAGDADVDYFRRRAGG*VWRAGAGR*RAATRQYTRHCSVR*NRDAD*REAAGCRSENIC*C**SAGIASGGGTG
ARFQPSAGTSDPR*SR*YAATAGQRFPHIARAGRER*S*RSCVIAGQSGAVK*ATGWYQSYRSGDYCSGIARGNACAAGG*RESGSPAGSTRS
VA***RGGAATPA*SGISSGDVDRG*PNHRQCDRQRSRD**GDRRGAAGR*SRSDQTSAK*RTSGGNGGRRH*RRASAGSGGCRHCDGWRQ*C
CH*NRGDYPDAP*PDGRCGCARYFPRNAAQHEAEPARCVYLQQYRYSGRRRYFVAVHWNTA*PGSCRSGNGALVDYRSE*RQPVAAV*TEGIX
